# SIRT2 mediates integrated stress response by deacetylating and stabilizing 4E-BP1 to suppress translation

**DOI:** 10.1038/s44319-026-00803-7

**Published:** 2026-05-18

**Authors:** Yanlin Zi, Jiaqi Zhao, Miao Wang, Dan Hou, Richard A Cerione, Hening Lin

**Affiliations:** 1 Department of Chemistry and Chemical Biology, Cornell University, Ithaca, NY USA; 2 Department of Medicine and Department of Chemistry, The University of Chicago, Chicago, IL USA; 3 Howard Hughes Medical Institute; Department of Medicine and Department of Chemistry, The University of Chicago, Chicago, IL USA

**Keywords:** Metabolism, Translation & Protein Quality

## Abstract

The ability to adapt to nutrient stress, such as amino acid limitation, is crucial for cell survival. The mTORC1 complex and integrated stress response (ISR) are two mechanisms that sense the availability of amino acids and regulate protein synthesis. Here, we reveal a new SIRT2-mediated pathway, downstream of the ISR, that is activated under amino acids limitation to suppress global translation. Under amino acid deprivation, SIRT2 protein level is upregulated translationally by its upstream open reading frame (uORF). SIRT2 in turn suppresses translation, which helps cells to survive amino acid limitation. We identify eukaryotic translation initiation factor 4E (eIF4E) binding protein 1 (4E-BP1), which binds to eIF4E and inhibits translation, as a substrate of SIRT2. SIRT2 deacetylates 4E-BP1 at lysine 69 and stabilizes 4E-BP1 by protecting it from proteasomal degradation, leading to suppression of global translation. Our study uncovers a role for SIRT2 in regulating translation and identifies a new regulatory mechanism of 4E-BP1 in cells.

## Synopsis

Amino acid limitation activates the ISR to increase SIRT2 translation via a 5′ uORF. SIRT2 deacetylates 4E-BP1 at K69, protects it from proteasomal degradation, which suppresses protein synthesis, and increases cell survival in this condition.

- ISR induces SIRT2 translation via a SIRT2 uORF during amino acid deprivation.
- SIRT2 deacetylates 4E-BP1 at K69, which reduces its proteasomal degradation and reinforces eIF4E inhibition.
- SIRT2 promotes cell survival under amino acid limitation.

## Abstract (web summary)

Amino acid limitation activates the ISR to increase SIRT2 translation via a 5′ uORF. SIRT2 deacetylates 4E-BP1 at K69, protects it from proteasomal degradation, which suppresses protein synthesis, and increases cell survival in this condition.

## Graphical abstract

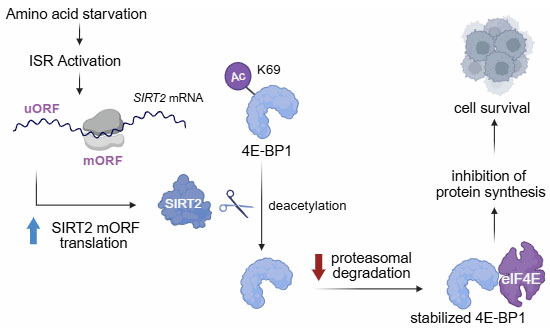

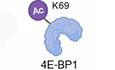

## Introduction

Stress response pathways are crucial for cell survival, and they are often heavily relied on by cancer cells, which are constantly subjected to various stress conditions (Hanahan and Weinberg, 2011). Cancer cells must adapt to nutrient and oxygen limitations, proteotoxic stress, and DNA damage. Therefore, stress response pathways have become part of cancer cells’ non-oncogene addiction (Luo et al, 2009). By targeting proteins involved in this form of non-oncogene addiction, such as stress response proteins, tumors can be selectively targeted while sparing healthy cells. If the intricacies of the stress response pathways can be better understood, it would aid the design and development of drugs targeting diseases, including cancer.

The integrated stress response (ISR) pathway is an elaborated signaling pathway that enables cells to respond and handle stresses from amino acid deprivation, viral infection, heme deprivation, and ER stress (Pakos-Zebrucka et al, 2016). When cells sense one of these four types of stresses, an ISR regulator (GCN2 for amino acid stress, PKR for viral infection, HRI for heme stress, and PERK for ER stress) is activated and triggers the phosphorylation of eukaryotic translation initiation factor 2α (eIF2α) on Ser51. This phosphorylation of eIF2α attenuates global cap-dependent mRNA translation but initiates the preferential translation of certain mRNAs that contain upstream open reading frames (uORF), such as activating transcription factor 4 (ATF4), as a mechanism promoting cell survival and recovery (Harding et al, 2000).

Like other stress adaptation pathways, defects in ISR have also been shown to suppress the development of cancer and other diseases. Inhibition of eIF2α Ser51 phosphorylation was reported to trigger cytotoxicity in aggressive metastatic prostate cancer (Nguyen et al, 2018). ATF4, one of the few well-studied uORF-regulated proteins in ISR, was reported to be overexpressed in solid tumors and critical for maintaining metabolic homeostasis in tumor cells (Ye et al, 2010). This underlies the importance of identifying more proteins directly regulated by ISR and understanding the stress responses of cancer cells.

mTORC1 regulates several critically important cellular processes. It affects cell growth by regulating mRNA translation, metabolism, and protein turnover (Saxton and Sabatini, 2017). Like the ISR pathway, mTORC1 also senses growth factors and specific amino acids, such as leucine and arginine (Wolfson et al, 2016; Wolfson and Sabatini, 2017), as signaling cues to promote cell growth and survival. Thus, when amino acids are abundant, mTORC1 activity increases. mTORC1 then promotes protein synthesis through phosphorylation of ribosomal S6 kinase (S6K), which induces translation (Ma et al, 2008; Dorrello et al, 2006), and eukaryotic translation initiation factor 4E (eIF4E)-binding protein 1 (4E-BP1), which negates the inhibitory effects it would otherwise have on translation (Gingras et al, 1999; Brunn et al, 1997).

Sirtuins are NAD^+^ dependent deacetylases. Within the sirtuin family, there are seven members, SIRT1-7, in humans (North and Verdin, 2004). Although they are highly conserved, each sirtuin has its own localization and cellular function (Cantó et al, 2013). In this study, we primarily focus on SIRT2, which was discovered over two decades ago (Gottlieb and Esposito, 1989), but whose complete function remains incompletely understood. In addition to exhibiting strong deacetylase activity, SIRT2 has also been reported to remove long-chain fatty acyl groups (Wang et al, 2022; Teng et al, 2015; Kosciuk et al, 2020; Jing et al, 2017; Spiegelman et al, 2019). It has been shown to play important roles in regulating gene expression (Zhang et al, 2013, 2016), signal transduction (Schartner et al, 2018; Wang and Tong, 2009; Jing et al, 2007), metabolic disorders (Rothgiesser et al, 2010; Lee et al, 2014), and various diseases, including cancer (Jing et al, 2016), diabetes (Watanabe et al, 2018), inflammatory bowel disease (IBD) (Hou et al, 2024), and neurodegenerative diseases (Outeiro et al, 2007). Eukaryotic translation initiation factor 5A (eIF5A) has been reported to be a deacetylation substrate of SIRT2, but whether and how SIRT2 regulates translation is not well known (Ishfaq et al, 2012).

In this study, we demonstrate that SIRT2 is translationally upregulated in response to amino acid stress via ISR and that SIRT2 negatively regulates global protein synthesis. Moreover, we discovered that 4E-BP1 is a substrate of SIRT2. SIRT2 catalyzes the deacetylation of 4E-BP1 at lysine 69 (K69), which is then protected from proteasomal degradation, thereby contributing to translational suppression.

## Results

### SIRT2 protein level is upregulated under amino acid limitation

**Figure 1:**
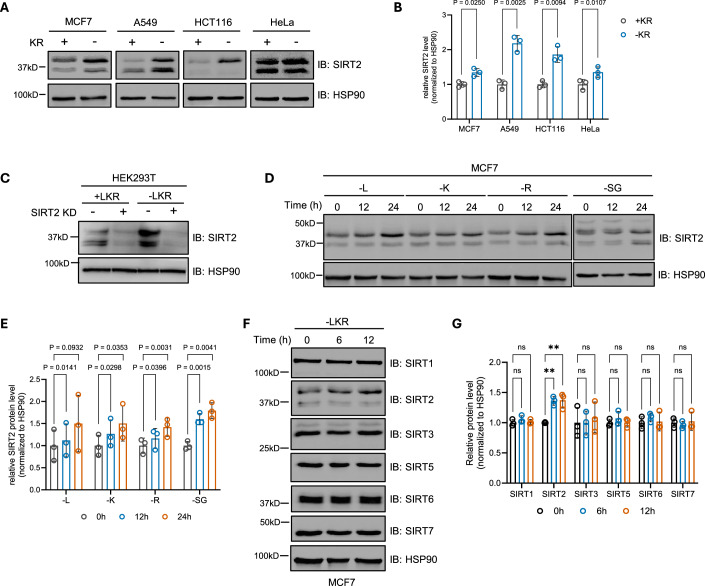
SIRT2 is upregulated under amino acid limitation. (**A**) SIRT2 level is upregulated in multiple cell lines when starved with lysine and arginine (KR) for 24 h. SIRT2 levels in MCF7, A549, HCT116, and HeLa cells with or without lysine and arginine starvation were measured by western blot. (**B**) Quantification of SIRT2 protein levels in (**A**). *P* = 0.0250 (MCF7), *P* = 0.0025 (A549), *P* = 0.0094 (HCT116), *P* = 0.0107 (HeLa). *P* values are determined using paired *t* test. (**C**) SIRT2 level is upregulated in HEK293T cells when subjected to leucine, lysine, and arginine (LKR) starvation for 24 h. Western blot was used to determine SIRT2 levels. SIRT2 knockdown (KD) HEK293T cells were negative controls. (**D**) Immunoblots for SIRT2 levels in MCF7 cells starved of leucine (L), lysine (K), arginine (R), and serine and glycine (SG) for 12 and 24 h. (**E**) Quantification of SIRT2 protein levels in (**D**). *P* = 0.0141 (-L, 12 h), *P* = 0.0932 (-L, 24 h); *P* = 0.0298 (-K, 12 h), *P* = 0.0353 (-K, 24 h); *P* = 0.0396 (-R, 12 h), *P* = 0.0031 (-R, 24 h); *P* = 0.0015 (-SG, 12 h), *P* = 0.0041 (-SG, 24 h). *P* values are determined using paired *t* test. (**F**) Only SIRT2 level increases under leucine, lysine, and arginine (LKR) depletion. SIRT1/3/5/6/7 protein level is not affected by LKR depletion. MCF7 cells were starved of LKR for 6 and 12 h. Western blot was used to determine the sirtuin protein levels. (**G**) Quantification of sirtuin protein levels in (**F**). *P* = 0.8588 (SIRT1, 6 h), *P* = 0.9896 (SIRT1, 12 h); *P* = 0.0037 (SIRT2, 6 h), *P* = 0.0028 (SIRT2, 12 h); *P* = 0.9987 (SIRT3, 6 h), *P* = 0.6884 (SIRT3, 12 h); *P* = 0.7691 (SIRT5, 6 h), *P* = 0.9995 (SIRT5, 12 h); *P* = 0.6788 (SIRT6, 6 h), *P* = 0.9800 (SIRT6, 12 h); *P* = 0.9392 (SIRT7, 6 h), *P* = 0.9705 (SIRT7, 12 h). *P* values are determined using two-way ANOVA. Representative data from three biologically independent experiments. Data with error bars are mean ± SD, **P < 0.01; ns, not significant. Source data are available online for this figure.

To investigate whether SIRT2 is regulated in response to amino acid limitation, we removed certain amino acids from the cell culture medium. Depletion of lysine and arginine (KR) led to consistent upregulation of SIRT2 protein levels across various cancer cell lines (Fig. 1A,B, different cell lines used in the study are listed in Appendix Table S1). Similarly, HEK293T cells upregulated SIRT2 when starved with leucine, lysine, and arginine (LKR) (Fig. 1C). We then tested additional amino acid depletion conditions. SIRT2 upregulation was observed under all conditions examined, including deprivation of branched-chain amino acids (leucine) and charged amino acids (lysine or arginine) individually, as well as non-essential amino acids (serine and glycine) (Fig. 1D,E). Collectively, these findings, observed across multiple cell types and deprivation settings, indicate that the increased SIRT2 protein level in response to amino acid deprivation is a general phenomenon.

To gain a deeper understanding of the specificity of SIRT2 upregulation, we assessed the response of several other sirtuins to amino acid deprivation. Of those examined (SIRT1/2/3/5/6/7), only the SIRT2 protein level elevated with LKR depletion (Fig. 1F,G), highlighting a unique role for SIRT2 in response to amino acid stress.

### Upregulation of SIRT2 is controlled translationally by its uORFs

**Figure 2:**
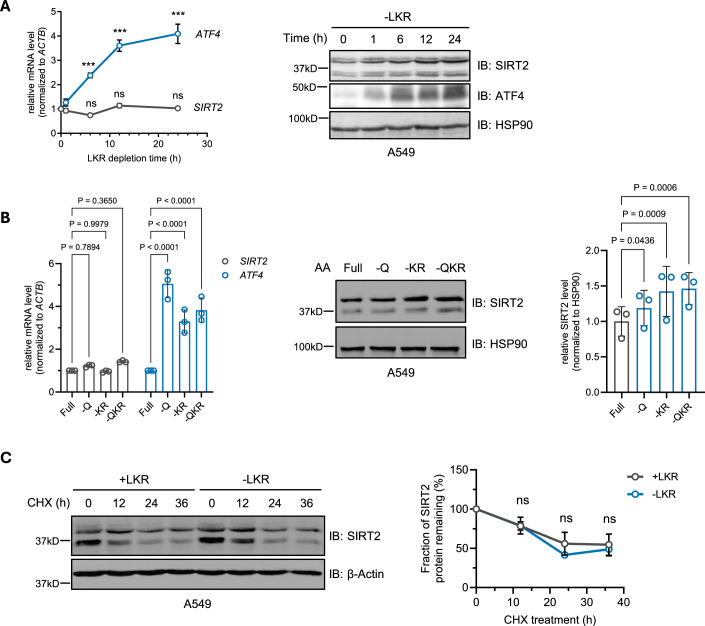
Increased SIRT2 protein level is not due to changes in transcription or protein stability. (**A**) RT-qPCR analysis of SIRT2 and ATF4 mRNA levels under leucine, lysine, and arginine (LKR) depletion in A549 cells (left). Immunoblotting (right) analysis of SIRT2 and ATF4 protein levels under the same conditions. *P* = 0.9756 (*SIRT2*, 1 h), *P* = 0.2596 (*SIRT2*, 6 h), *P* = 0.8519 (*SIRT2*, 12 h), *P* = 0.9995 (*SIRT2*, 24 h); *P* = 0.2359 (*ATF4*, 1 h), *P* = 1.53 × 10^−8^ (*ATF4*, 6 h), *P* = 2.46 × 10^−13^ (*ATF4*, 12 h), *P* = 1.11 × 10^−14^ (*ATF4*, 24 h). *P* values are determined using two-way ANOVA. (**B**) SIRT2 mRNA levels remain the same under different combinations of amino acids depletion. A549 cells were treated with starvation of glutamine (Q), lysine and arginine (KR), or a combination of all three (QKR) for 24 h. RT-qPCR analysis (left) and immunoblotting (right) were used to assess mRNA and protein levels, respectively. RT-qPCR: *P* = 0.7894 (*SIRT2*, -Q), *P* = 0.9979 (*SIRT2*, -KR), *P* = 0.3650 (*SIRT2*, -QKR); *P* = 2.32 × 10^−7^ (*ATF4*, -Q), *P* = 2.59 × 10^−4^ (*ATF4*, -KR), *P* = 2.46 × 10^−5^ (*ATF4*, -QKR). *P* values are determined using two-way ANOVA. Immunoblotting: *P* = 0.0436 (-Q), *P* = 0.0009 (-KR), *P* = 0.0006 (-QKR). *P* values are determined using paired *t* test. (**C**) LKR depletion does not affect the stability of SIRT2. Cycloheximide (CHX) chase experiment was utilized. A549 cells were treated with 100 μg/ml CHX for 0, 12, 24, or 36 h. For the western blot quantification (right), SIRT2 protein level from the sample without CHX is set to 100%. *P* = 0.9999 (12 h), *P* = 0.2861 (24 h), *P* = 0.8613 (36 h). *P* values are determined using one-way ANOVA. Representative data from three biologically independent experiments. Data with error bars are mean ± SD, ***P < 0.001; ns, not significant. Source data are available online for this figure.

We next aimed to elucidate the mechanism by which SIRT2 is upregulated under amino acid stress. Amino acid deprivation is one of the stresses that activate the ISR. In the ISR, upregulation of ATF4 is the best-understood mechanism, where ATF4 activates the transcription of its target gene, including itself, to overcome amino acid stress (Pakos-Zebrucka et al, 2016). Therefore, we first asked whether SIRT2 upregulation is through transcription, potentially serving as a target gene of ATF4. However, RT-qPCR analysis revealed no significant changes in SIRT2 mRNA levels following LKR depletion, whereas ATF4 mRNA showed a strong time-dependent induction (Fig. 2A). Similarly, when deprived of glutamine (Q), lysine and arginine (KR), or all three combined (QKR), the relative SIRT2 mRNA levels remained comparable to the sample without any amino acid deprivation, even though SIRT2 protein levels had increased significantly (Fig. 2B). These results suggest that the upregulation of SIRT2 protein levels is not due to increased transcription.

Altering protein stability is another regulatory mechanism when cells encounter stress conditions. To determine if the increased SIRT2 level results from greater protein stability, we treated A549 cells with the protein synthesis inhibitor cycloheximide (CHX) and monitored SIRT2 protein levels to assess SIRT2 degradation rates in the presence or absence of LKR deprivation. Immunoblotting analysis indicated comparable SIRT2 stability between the amino acid-starved and replete conditions (Fig. 2C). Therefore, the protein stability difference does not account for the increased SIRT2 protein level under amino acid depletion.

After ruling out regulation at transcriptional and protein stability levels, it became highly possible that increased SIRT2 translation is responsible for the upregulated SIRT2 protein level upon amino acid deprivation. Under conditions of ISR, eIF2α is phosphorylated on Ser51 and becomes inactive. This inactivation leads to a decreased concentration of the ternary complex, allowing scanning ribosomes to skip the inhibitory uORF of specific mRNAs, such as ATF4. As a result, there is increased translation of the main open reading frame (mORF) (Pakos-Zebrucka et al, 2016). We hypothesized that the translation of SIRT2 is regulated through uORFs similarly to that of ATF4.

To test our hypothesis, we first examined whether amino acid deprivation activates the ISR under the same conditions that increase SIRT2 protein levels. Amino acid limitation is known to activate the ISR primarily through the kinase GCN2, which phosphorylates eIF2α at Ser51. Consistent with activation of this pathway, phosphorylation of eIF2α at Ser51 was strongly induced across all cell lines tested (Appendix Fig. S1). To further determine whether ISR signaling is required for SIRT2 upregulation, we treated cells with ISRIB, a small molecule that restores eIF2B activity and functionally antagonizes the effects of eIF2α phosphorylation. ISRIB treatment diminished the amino acid limitation-induced increase in SIRT2 protein levels (Appendix Fig. S2). Together, these results support a model in which amino acid limitation activates the ISR pathway, leading to enhanced translation of SIRT2 mRNA.

**Figure 3:**
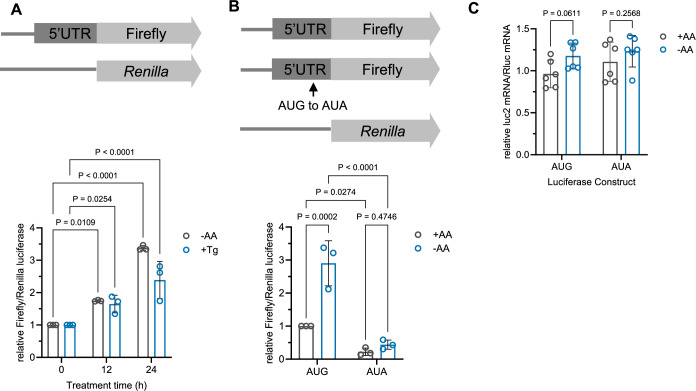
Upregulation of SIRT2 translation is mediated by its uORFs. (**A**) Top: luciferase constructs designed to examine the role of SIRT2 5’UTR region in translation. Bottom: the ratio of firefly luciferase activity over Renilla luciferase activity in HeLa cells with leucine, lysine, and arginine (LKR) depletion or 1 µM thapsigargin (Tg) treatment. *P* = 0.0109 (-AA, 12 h), *P* = 2.91 × 10^−7^ (-AA, 24 h); *P* = 0.0254 ( + Tg, 12 h), *P* = 7.60 × 10^−5^ ( + Tg, 24 h). (**B**) Top: Luciferase constructs designed to examine the role of uORF (133–234) in SIRT2 translation. AUG at 5’UTR position 133 was mutated to AUA. Bottom: The ratio of firefly luciferase activity over Renilla luciferase activity in HeLa cells starved of LKR. *P* = 0.0002 (AUG, +AA vs -AA), *P* = 0.4746 (AUA, +AA vs -AA), *P* = 0.0274 ( + AA, AUG vs AUA), *P* = 2.75 × 10^−5^ (-AA, AUG vs AUA). (**C**) RT-qPCR analysis of mRNA levels of luciferase constructs. *P* = 0.0611 (AUG), *P* = 0.2568 (AUG). Representative data from three biologically independent experiments. Data with error bars are mean ± SD. *P* values are determined using two-way ANOVA. Source data are available online for this figure.

We then utilized the luciferase reporter system, a well-established tool in the study of ISR-mediated translational changes (Palam et al, 2011; Vattem and Wek, 2004). The 5’UTR from the human SIRT2 variant 1 (NM_012237.3) was inserted into the pGL3 vector driven by the SV40 promoter. The start codon of the SIRT2 uORF was fused in frame with firefly luciferase. For an internal control, we used a Renilla luciferase reporter construct which was under the same promoter (Fig. 3A). The two constructs were co-transfected into HeLa cells, and the SIRT2 5’UTR-regulated translation efficiency was measured by the ratio of firefly luciferase activity over Renilla luciferase activity. In the presence of SIRT2 5’UTR, the translation efficiency increased after amino acid depletion or ER stress induction in a time-dependent manner (Fig. 3A). This result strongly suggests that the upregulation of SIRT2 is through translational regulation, likely via the uORFs in the 5’UTR region of the SIRT2 mRNA.

The most well-studied case of the uORF regulating translation is yeast Gcn4, and both inhibitory and activating uORFs have been established (Hinnebusch, 2014). When the inhibitory uORF (uORF4) is lost, ribosomes can scan to the main ORF, resulting in increased protein synthesis. When the activating uORF (uORF1) is lost, the ribosome dissociates more readily from mRNA, resulting in a non-inducible Gcn4 translation. We predicted potential upstream open reading frames in the 5’UTR of SIRT2 mRNA based on previous reports of ribosome profiling analysis (Ingolia et al, 2011) and performed mutagenesis to remove the corresponding start or stop codons. We identified one uORF (133–234) (Appendix Figs. S3 and S4) that, when mutated to the non-Kozak sequence, dramatically decreased translation and the ability of amino acid deprivation to increase translation (Fig. 3B), despite comparable luciferase mRNA levels (Fig. 3C). These results suggest that the uORF (133–234) in the SIRT2 5’UTR serves a stimulatory role, similar to uORF1 in Gcn4. Thus, we concluded that the translational upregulation of SIRT2 under amino acid depletion is regulated by the uORF in the 5’ UTR. We then analyzed publicly available ribosome profiling datasets using the GWIPS-viz genome browser. Consistent with our observations, we found that cross multiple independent studies and cell types, ribosome footprint signal is consistently detected over this uORF (133–234) region (Appendix Figs. S5 and S6), indicating that this uORF is ribosome-engaged and could thus potentially regulate the translation of the main SIRT2 ORF.

### Knocking down SIRT2 promotes protein synthesis and downregulates 4E-BP1 protein level

**Figure 4:**
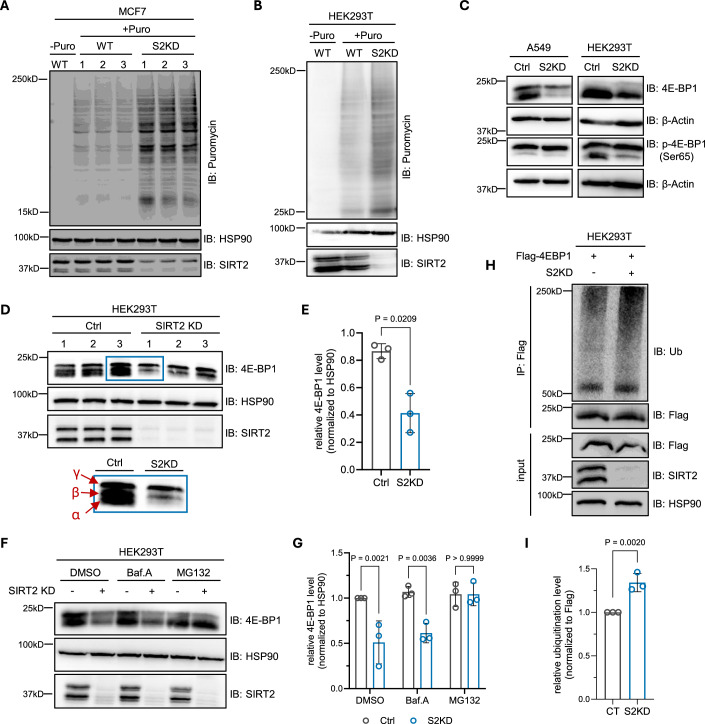
Knocking down SIRT2 promotes protein synthesis and downregulates 4E-BP1 through proteasome degradation. (**A**, **B**) SIRT2 knockdown MCF7 cells (**A**) and HEK293T cells (**B**) have upregulated protein synthesis. Wild-type (WT) and SIRT2 knockdown (S2KD) cells were treated with 10 μg/ml puromycin for 10 min before collection. Protein synthesis levels were measured using western blot. (**C**) 4E-BP1 and p-4E-BP1 (Ser65) levels in SIRT2 knockdown A549 and HEK293T. Protein levels were measured using western blot. (**D**) SIRT2 knockdown HEK293T cells have significantly reduced α isoform of 4E-BP1. Endogenous protein levels in control and SIRT2 knockdown HEK293T cells were evaluated using western blot. α, β, and γ phosphor isoforms are indicated with red arrows. (**E**) Quantification of 4E-BP1 protein levels in (**D**), *P* = 0.0209. *P* value is determined using paired *t* test. (**F**) MG132 treatment could rescue 4E-BP1 level in SIRT2 knockdown HEK293T cells. Control and SIRT2 knockdown HEK293T cells were treated with DMSO (control), 100 nM Bafilomycin A1, or 40 μM MG132 for 5 h before collection. Protein levels were evaluated using western blot. (**G**) Quantification of 4E-BP1 protein levels in (**F**). *P* = 0.0021 (DMSO), *P* = 0.0036 (Baf.A), *P* > 0.9999 (MG132). *P* values are determined using one-way ANOVA. (**H**) Knockdown of SIRT2 increases the 4E-BP1 ubiquitination level. Flag-tagged 4E-BP1 was transfected into control and SIRT2 knockdown HEK293T cells. Cells were treated with 40 μM MG132 for 6 h before collection. Ubiquitination level was analyzed using western blot after Flag IP. (**I**) Quantification of 4E-BP1 ubiquitination levels in (**H**), *P* = 0.0020. *P* values is determined using one-sample *t* test. Representative data from at least three biologically independent experiments. Data with error bars are mean ± SD. Source data are available online for this figure.

After establishing that SIRT2 is directly upregulated through translation by ISR via 5’ uORF, we then sought to explore the role of SIRT2 in stress response. Since protein synthesis is crucial for cell survival and often regulated by environmental factors, such as amino acid abundance (Roux and Topisirovic, 2018), we proceeded to verify if SIRT2 has any effect on protein synthesis. A previously established puromycin labeling method was used to probe global protein synthesis (Schmidt et al, 2009). In this assay, the immunoblotting signal of puromycin is directly proportional to the level of global translation. Notably, we observed that when SIRT2 was knocked out in MCF7 cells, there was a significant increase in puromycin labeling, indicating that lower levels of SIRT2 promote translation (Fig. 4A). Similar results were also observed in HEK293T cells. Knocking down SIRT2 in HEK293T cells resulted in increased puromycin labeling (Fig. 4B). We further confirmed this finding in experiments where the SIRT2 protein level was elevated, or its enzymatic activity was inhibited. Inhibition of SIRT2 using the small molecule inhibitor TM (Jing et al, 2016) increased translation, while overexpression of SIRT2 decreased translation (Appendix Fig. S7). Thus, SIRT2 has an inhibitory effect on global translation, providing an explanation for why SIRT2 levels are upregulated in response to amino acid stress. When amino acids are limited, cells increase SIRT2 to decrease translation levels and maintain amino acid homeostasis.

To further study SIRT2’s role in translation, we set out to identify a specific target that is important for protein synthesis and is affected by SIRT2 directly. Translation can be regulated through S6K, whose phosphorylation by mTORC1 will stimulate protein synthesis (Ma et al, 2008; Dorrello et al, 2006), and 4E-BP1, which exerts an inhibitory effect on protein synthesis when it is not phosphorylated by binding to the eukaryotic translation initiation factor 4E (eIF4E) (Brunn et al, 1997; Gingras et al, 1999). We observed that when SIRT2 was knocked down in A549 and HEK293T cells, total 4E-BP1 protein level was significantly decreased, which could increase global translation (Fig. 4C). Since SIRT2 has no significant effect on endogenous S6K protein level (Appendix Fig. S8), we decided to further examine the relationship between SIRT2 and 4E-BP1.

### Knocking down SIRT2 promotes the proteasomal degradation of 4E-BP1

Based on immunoblot analysis, three 4E-BP1 isoforms can be identified: α, β, and γ. The α isoform is the least phosphorylated and migrates the furthest when examined by SDS-PAGE and Western blotting. The β isoform is phosphorylated at an intermediate level, and the γ isoform is hyperphosphorylated. It is the α and β isoforms of 4E-BP1 that bind to eIF4E to inhibit protein synthesis (Gingras et al, 1998, 1996; Beretta et al, 1996). When using a gradient gel to obtain better resolution for small proteins, we observed that when SIRT2 was knocked down in HEK293T cells, the total 4E-BP1 level decreased by roughly 50% compared to the control cells (Fig. 4D,E). The primary protein level difference was with the α isoform. When SIRT2 was knocked down, most of the 4E-BP1 α isoform was eliminated, while the γ isoform was less affected. This finding is consistent with the observation that knocking down SIRT2 promotes global protein synthesis, given that the 4E-BP1 α and β isoforms inhibit translation.

We then examined how SIRT2 downregulated 4E-BP1. Given that SIRT2 is reported to affect the degradation of its substrates (Karim et al, 2024; Jiang et al, 2011), we hypothesized that the downregulation of 4E-BP1 could also be due to increased degradation. To test this hypothesis, HEK293T cells were treated with DMSO, Bafilomycin A1 for lysosomal inhibition, or MG132 for proteasomal inhibition. While the DMSO and the Bafilomycin A1 treated groups still displayed lower endogenous 4E-BP1 protein levels in the SIRT2 knockdown cells, the difference in 4E-BP1 protein expression between control and SIRT2 knockdown cells was rescued in the MG132 treated group (Fig. 4F,G). This result shows that SIRT2 affects the degradation of 4E-BP1 through the proteasome. To further confirm this finding, ubiquitination of 4E-BP1 was analyzed given that this post-translational modification leads to proteasomal degradation (Ciechanover and Schwartz, 1998). As expected, 4E-BP1 in SIRT2 knockdown HEK293T cells showed a significantly higher level of ubiquitination compared to control cells, confirming that knocking down SIRT2 promotes the ubiquitination and degradation of 4E-BP1 (Fig. 4H,I).

### SIRT2 deacetylates 4E-BP1 at K69 to stabilize 4E-BP1

**Figure 5:**
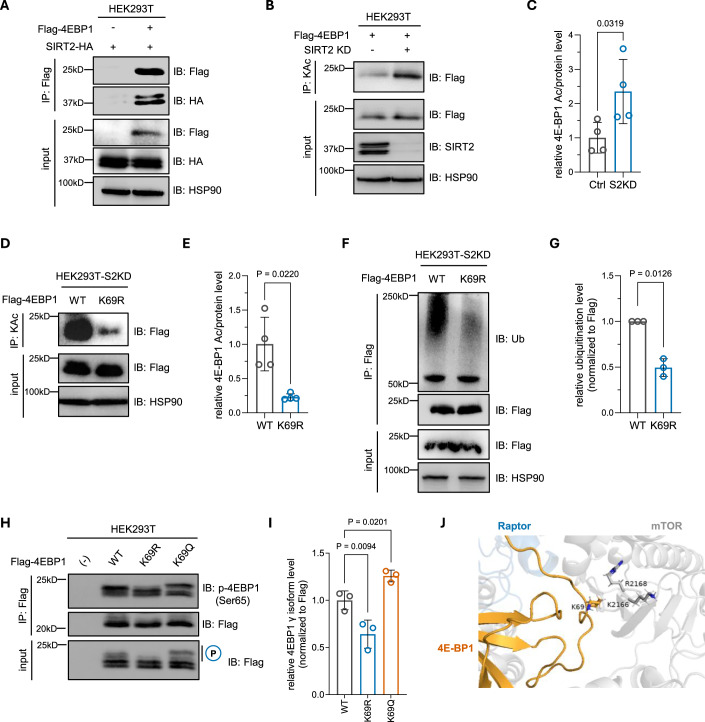
Deacetylation of 4E-BP1 at K69 by SIRT2 stabilizes 4E-BP1. (**A**) SIRT2 interacts with 4E-BP1. HEK293T cells were transfected with HA-tagged SIRT2 along with Flag-tagged empty vector or Flag-tagged 4E-BP1. Co-immunoprecipitated proteins were examined after Flag-IP. (**B**) 4E-BP1 is deacetylated by SIRT2. Flag-tagged 4E-BP1 was transfected into control and SIRT2 knockdown HEK293T cells. Cells were treated with 40 μM MG132 for 6 h before collection. Acetylation of 4E-BP1 was examined via acetyl lysine IP. (**C**) Quantification of relative 4E-BP1 acetylation levels between control and SIRT2 knockdown HEK293T cells. *P* = 0.0319. *P* value is determined using paired *t* test. (**D**) SIRT2 deacetylates 4E-BP1 at K69. Flag-tagged 4E-BP1 or 4E-BP1 K69R mutant was transfected into SIRT2 knockdown HEK293T cells. Cells were treated with 40 μM MG132 for 6 h before collection. Acetylation levels of 4E-BP1 WT and K69R mutant were assessed via acetyl lysine IP. (**E**) Quantification of 4E-BP1 acetylation levels in (**D**). *P* = 0.0220. *P* value is determined using paired *t* test. (**F**) Deacetylation of 4E-BP1 at K69 by SIRT2 decreases the ubiquitination level. Flag-tagged 4E-BP1 WT or K69R mutant was transfected into SIRT2 knockdown HEK293T cells. Cells were treated with 40 μM MG132 for 6 h before collection. Ubiquitination level of 4E-BP1 was analyzed after Flag IP. (**G**) Quantification of ubiquitination levels in (**F**). *P* = 0.0126. *P* value is determined using one-sample *t* test. (**H**) Phosphorylation levels of wild-type (WT) 4E-BP1, acetylation-deficient mutant (K69R), and acetylation-mimic mutant (K69Q) in HEK293T cells. (**I**) Quantification of hyperphosphorylation levels in (**H**). *P* = 0.0094 (K69R), *P* = 0.0201 (K69Q). *P* values are determined using paired *t* test. (**J**) Modeled structure of p-4E-BP1 (T37/T46) in complex with mTOR and Raptor. Positions of 4E-BP1 K69 and mTOR K2166/R2168 are highlighted in sticks. 4E-BP1 (brightorange), mTOR (gray), and Raptor (skyblue). Representative data from at least three biologically independent experiments. Data with error bars are mean ± SD. Source data are available online for this figure.

We noticed that SIRT2 interacts with 4E-BP1 through co-immunoprecipitation (Fig. 5A), which encouraged us to investigate whether 4E-BP1 is a SIRT2 substrate. We then examined if 4E-BP1 is acetylated and, if so, whether it can be deacetylated by SIRT2. Flag-tagged 4E-BP1 was transfected into control and SIRT2 knockdown HEK293T cells. Since SIRT2 affects 4E-BP1 proteasomal degradation, cells were treated with MG132 for 6 h before collection to ensure the inhibition of degradation. Acetylated proteins were enriched using anti-acetyl lysine beads, and the amount of 4EB-BP1 in the output sample were assessed via immunoblotting to analyze the acetylation level of 4E-BP1. Knocking down SIRT2 increased the acetylation level of 4E-BP1 (Fig. 5B). Quantification of four independent replicates showed that knocking down SIRT2 increases the acetylation of 4E-BP1 by approximately 2.5-fold (Fig. 5C). SIRT2 inhibitor TM also increased 4E-BP1 acetylation level in a dose-dependent manner (Appendix Fig. S9). Collectively, these data support that SIRT2 deacetylates 4E-BP1.

After verifying that 4E-BP1 is a substrate of SIRT2, we aimed to identify the deacetylate site of 4E-BP1 by SIRT2, which could further help to elucidate the function of the deacetylation. 4E-BP1 contains three lysine residues: K57, K69, and K105. Single lysine-to-arginine (K to R) mutants were prepared to determine the deacetylation site (Appendix Fig. S10). The K69R mutant had a significantly decreased acetylation level compared to wild-type 4E-BP1 (Fig. 5D,E), suggesting that K69 is the major site of deacetylation by SIRT2. Since we found that SIRT2 affects the ubiquitination level of 4E-BP1, we hypothesized that this effect could be through deacetylation at K69. Indeed, the K69R mutant showed a marked reduction in ubiquitination level compared to the wild-type 4E-BP1, indicating that deacetylation of 4E-BP1 at K69 inhibits the ubiquitination of 4E-BP1 (Fig. 5F,G). This result also agrees with the observation that there was more endogenous 4E-BP1 in control cells than in SIRT2 knockdown cells.

A previous study identified CUL3 as an E3 ligase of 4E-BP1 (Yanagiya et al, 2012). To understand the degradation of 4E-BP1 further, we sought to investigate whether acetylation affects the interaction between 4E-BP1 and CUL3. In HEK293T SIRT2KD cells, we found that WT 4E-BP1, but not K69R mutant, strongly co-immunoprecipitated with CUL3 (Appendix Fig. S11). Taken together, our data suggests that SIRT2 deacetylates 4E-BP1 at K69, and this deacetylation inhibits 4E-BP1 ubiquitination, preventing it from being degraded through the proteasome. This could be one mechanism contributing to SIRT2’s ability to suppress protein synthesis during amino acid limitation.

We also tested the potential crosstalk between 4E-BP1 acetylation and mTORC1-mediated phosphorylation. When we overexpressed wild-type (WT), acetylation-deficient (K69R), and acetylation-mimic (K69Q) 4E-BP1 in HEK293T cells, the acetylation-deficient mutant showed markedly reduced hyperphosphorylation at Ser65, whereas the acetylation-mimic mutant showed significantly increased hyperphosphorylation (Fig. 5H,I). On the contrary, when inhibited mTOR function with rapamycin, the acetylation level of 4E-BP1 remains the same (Appendix Fig. S12). These results suggest that acetylation at K69 may influence phosphorylation of 4E-BP1 by mTORC1. Consistent with this idea, a previously reported structure model of phosphorylated 4E-BP1 (T37/T46) in complex with mTORC1 (Böhm et al, 2021) places K69 at the interface between 4E-BP1 and mTOR (Fig. 5J; Appendix Fig. S13). In this model, K69 lies near positively charged mTOR residues K2166 and R2168. Because acetylation neutralizes the positive charge of lysine, modification at K69 may alter electrostatic interactions at this interface and influence subsequent phosphorylation events.

### SIRT2 helps cancer cell survive amino acid depletion

**Figure 6:**
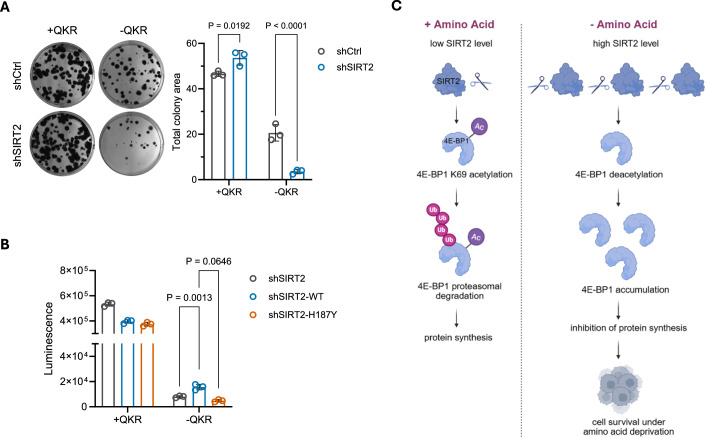
SIRT2 protects cancer cells from amino acid limitation induced stress. (**A**) SIRT2 protects A549 cells after QKR depletion in the clonogenic survival assay. Equal number of SIRT2 KD and control A549 cells were seeded and exposed to or not to lysine, arginine, and glutamine (QKR) depletion. The cells were then reseeded at a low confluency in complete medium and allowed to proliferate and form foci. The number and size of the foci formed were indications of cell survival under amino acid depletion. *P* = 0.0192 ( + QKR), *P* = 7.48 × 10^−5^ (-QKR). *P* values are determined using two-way ANOVA. (**B**) SIRT2 catalytic activity is necessary for its protective role in cancer cell survival after QKR depletion. SIRT2 KD A549 cells were rescued with SIRT2 WT or catalytically dead mutant H187Y and subjected to QKR depletion as in (**A**). The cells were then reseeded in complete medium and assessed for proliferation. *P* = 0.0013 (-QKR, shSIRT2 vs shSIRT2-WT), *P* = 0.0646 (-QKR, shSIRT2-WT vs shSIRT2-H187Y). *P* values are determined using paired *t* test. (**C**) Proposed working model. Under amino acid limitation, SIRT2 protein level is upregulated, and 4E-BP1 is more prone to deacetylation by SIRT2. SIRT2 deacetylates 4E-BP1 at K69, which inhibits its ubiquitination and proteasomal degradation. The accumulated 4E-BP1 binds to eIF4E, inhibiting protein synthesis. In contrast, when amino acids are abundant, less SIRT2 is present, and more 4E-BP1 is acetylated. Acetylated 4E-BP1 is more ubiquitinated, which leads to proteasomal degradation. Because of the decreased endogenous 4E-BP1, translation is upregulated. Representative results from three biologically independent replicates. Data with error bars are mean ± SD. Source data are available online for this figure.

Our data so far showed that under amino acid depletion, SIRT2 suppressed translation at least partially through deacetylation of 4E-BP1 K69 and inhibiting its ubiquitination and degradation. Suppression of global protein synthesis under amino acid limitation helps preserve cellular energy and resources (Roux and Topisirovic, 2018; Liu and Qian, 2014; Jobava et al, 2021). We next asked whether SIRT2’s role in translation suppression could provide a survival advantage to cells under amino acid depletion. Using a clonogenic survival assay, we found that control and SIRT2 knockdown cancer cells formed similar numbers of colonies under normal conditions, whereas under amino acid deprivation, control cells formed significantly larger and more colonies than SIRT2 knockdown cells (Fig. 6A). Moreover, cell survival could be rescued by overexpressing WT SIRT2, but not the catalytic dead mutant (Fig. 6B). These results suggest that SIRT2 helps cancer cells survive amino acid limitation by suppressing translation. We therefore propose a model in which SIRT2 is translationally upregulated under amino acid limitation and suppresses global protein synthesis through stabilization of 4E-BP1, which helps cells survive the amino acid limitation (Fig. 6C).

## Discussion

Our study shows that SIRT2 is upregulated translationally under amino acid stress via its 5’uORF. The upregulated SIRT2 then suppresses translation by deacetylating and stabilizing the unphosphorylated 4E-BP1. These findings further broaden our understanding of SIRT2 as a stress response protein. SIRT2 is known to play a crucial role in responding to oxidative stress (Wang et al, 2007, 2012; Singh et al, 2017), mitotic stress (Inoue et al, 2007), replication stress (Zhang et al, 2013), and Golgi stress (Wang et al, 2022). For example, SIRT2 is reported to be upregulated under oxidative stress and deacetylates the transcription factor FOXO3a, which in turn increases FOXO3a transactivation activity, reduces cellular ROS levels, and promotes cell death under severe oxidative stress (Wang et al, 2007). Although previous studies reported that SIRT2 levels are elevated under caloric restriction, the underlying mechanism remained unclear. Here, we provide mechanistic insight into how SIRT2 is upregulated when cells experience nutrient stress. We show that amino acid limitation increases SIRT2 translation through the integrated stress response (ISR) pathway. Translation is subject to complex regulations and is essential for cell survival and homeostasis. Our study provides new insight into how cells adapt to amino acid deprivation by identifying 4E-BP1 as a substrate of SIRT2 and revealing an additional layer of translational control mediated by protein acetylation.

The mechanism of SIRT2 upregulation resembles that of ATF4. Bioinformatics studies have shown that nearly half of the human transcriptome contains uORFs (Ingolia et al, 2011), yet relatively few transcripts are known to be directly regulated by uORFs. Our study identifies SIRT2 as a new transcript whose translation is regulated by a uORF in response to amino acid limitations, thereby expanding our understanding of uORF-mediated translational regulation. We propose that, like ATF4, SIRT2 is an important effector of the ISR pathway. Whereas ATF4 functions as a transcription factor that coordinates stress responses at the transcription level, SIRT2 functions as an enzyme that modifies multiple substrate proteins and coordinates the stress response at the post-translational level.

4E-BP1 has long been recognized as a key regulator of protein synthesis, and its phosphorylation has been widely studied. Phosphorylation of 4E-BP1 by mTORC1 causes 4E-BP1 to dissociate from eIF4E, thereby promoting translation (Gingras et al, 1999; Brunn et al, 1997). However, regulation of 4E-BP1 by acetylation has not been previously described. In this study, we identify K69 as an acetylation site on 4E-BP1, and found that this modification is removed by SIRT2. K69 acetylation promotes ubiquitination and proteasomal degradation of 4E-BP1, whereas SIRT2-mediated deacetylation stabilizes the protein and suppresses translation. Ubiquitination of 4E-BP1 has been reported to occur on K57, although how this process is regulated has not been fully understood (Yanagiya et al, 2012). Our findings suggest that under amino acid deprivation, increase SIRT2 levels promote deacetylation of 4E-BP1 at K69, which in turn reduces ubiquitination at K57 and stabilizes the protein. As a result, more endogenous 4E-BP1 becomes available to bind eIF4E and inhibits protein synthesis.

While we focus on 4E-BP1 as a SIRT2 substrate underlying the regulation of translation, SIRT2 likely suppresses translation through multiple mechanisms. In a related manuscript under consideration (Shrama et al, 2026), SIRT2 is shown to deacetylate RheB, a small GTPase that activates mTORC1, thereby limiting mTORC1 signaling (Shrama et al, 2026). In our system, SIRT2 knockdown increased S6K phosphorylation (Appendix Fig. S8), consistent with enhanced mTORC1 activity and suggesting that regulation of the RheB-mTOC1 pathway may also contribute to the translational effects of SIRT2. Similarly, in the related manuscript, SIRT2 knockout also decreased 4E-BP1 levels, consistent with our finding here.

Our findings suggest that SIRT2 regulates translation at two distinct levels. Upstream, SIRT2 suppresses mTORC1 signaling through deacetylation of Rheb, reducing phosphorylation of downstream targets such as 4E-BP1 and S6K. Downstream, SIRT2 directly stabilizes 4E-BP1 through deacetylation of K69, thereby increasing the abundance of this translational repressor. These mechanisms are therefore complementary. Inhibition of mTORC1 reduces phosphorylation of 4E-BP1, whereas stabilization of 4E-BP1 increases the total pool of the translational inhibitor available to bind eIF4E. Together, these regulatory layers would be expected to reinforce suppression of cap-dependent translation during nutrient stress.

Our data also suggest potential crosstalk between 4E-BP1 acetylation and mTORC1-mediated phosphorylation. We observed that the acetylation-deficient mutant showed markedly reduced hyperphosphorylation at Ser65, whereas the acetylation-mimic mutant showed significantly increased hyperphosphorylation (Fig. 5H,I).

More broadly, our results establish SIRT2 as an important stress-response protein. With its levels and activities upregulated by diverse stresses, SIRT2 plays a critical role in helping cells survive these stresses. Interestingly, SIRT2 inhibitors have been recognized to have broad anticancer activity while showing limited toxicity in normal cells (Jing et al, 2016). The stress-response role of SIRT2 may provide a logical explanation for the broad and cancer cell-selective toxicity of SIRT2 inhibitors, as cancer cells often experience higher levels of metabolic and proteotoxic stress. Indeed, our clonogenic survival assay showed that under amino acid deprivation control cells formed significantly larger and more colonies than SIRT2 knockdown cells (Fig. 6A). These results indicate that SIRT2 helps cancer cell survive nutrient stress.

## Methods

**Table:** Reagents and tools table

Reagent/resource	Reference or source	Identifier or catalog number
**Experimental models**		
HEK293T (*Homo sapiens*)	ATCC	CRL-3216
HEK293T-shCtrl (*Homo sapiens*)	This paper	
HEK293T-shSIRT2 (*Homo sapiens*)	This paper	
MCF7 (*Homo sapiens*)	ATCC	HTB-22
MCF7-shCtrl (*Homo sapiens*)	This paper	
MCF7-shSIRT2 (*Homo sapiens*)	This paper	
HeLa (*Homo sapiens*)	ATCC	CCL-2
A549 (*Homo sapiens*)	ATCC	CCL-185
A549-shCtrl (*Homo sapiens*)	This paper	
A549-shSIRT2 (*Homo sapiens*)	This paper	
A549-shSIRT2-SIRT2-WT (*Homo sapiens*)	This paper	
A549-shSIRT2-SIRT2-H187Y (*Homo sapiens*)	This paper	
HCT116 (*Homo sapiens*)	ATCC	CCL-247
**Recombinant DNA**		
pGL3-control Vector	Promega	E1741
pGL3-control-SIRT2-5’UTR	This paper	
pGL3-control-SIRT2-5’UTR (26-88) AUG to AUA	This paper	
pGL3-control-SIRT2-5’UTR (133–234) AUG to AUA	This paper	
PRL-Renilla Vector	Promega	E2231
pcDNA3.1-4E-BP1-C-(K)-DYK	Genscript	OHu15712
pcDNA3.1-4E-BP1-C-(K)-DYK K to R/Q mutants	Genscript	customized
pcDNA3-myc-CUL3	Addgene	19893
pcDNA-Flag-SIRT2-WT	This paper	
pcDNA-Flag-SIRT2-H187Y	This paper	
lentiCas9-Blast	Addgene	52962
pFUGW-SIRT2-WT	This paper	
pFUGW-SIRT2-H187Y	This paper	
**Antibodies**		
SIRT2 (D4O5O)	CST	12650S
SIRT1 (D739)	CST	2493S
SIRT3 (D22A3)	CST	5490S
SIRT5 (D8C3)	CST	8782S
SIRT6 (D8D12)	CST	12486S
HSP90 (C45G5)	CST	4877S
ATF4 (D4B8)	CST	11815S
p-S6K Thr389 (9205)	CST	9205S
S6K (9202)	CST	9202S
p-4E-BP1 Ser65 (9451)	CST	9451S
4E-BP1 (9452)	CST	9452S
Ubiquitin (E4I2J)	CST	3124S
HA-Tag (C29F4)	CST	3724S
p-eIF2α Ser51 (D9G8)	CST	3398 T
eIF2α (L57A5)	CST	2103 T
Myc-Tag (71D10)	CST	2278S
Anti-mouse IgG HRP-linked antibody	CST	7076S
Anti-rabbit IgG HRP-linked antibody	CST	7074S
SIRT7 (C-3)	Santa Cruz	sc-365344
β-Actin (C4)	Santa Cruz	sc-47778
Anti-Flag M2 antibody conjugated with horseradish peroxidase	Sigma	A8592-5X1MG
Puromycin (12D10)	Sigma	MABE343
**Oligonucleotides and other sequence-based reagents**		
RT-qPCR primers	This paper	Methods section
Primers for site-directed mutagenesis	This paper	Methods section
Primers for NEBuilder® HiFi DNA Assembly	This paper	Methods section
**Chemicals, enzymes and other reagents**		
Anti-FLAG affinity gel	Sigma	A2220
Thapsigargin	Sigma	586006-2MG
cycloheximide	Sigma	239763-5GM
ISRIB	Sigma	SML0843-5MG
Crystal violet	Sigma	C0775-25G
puromycin	Sigma	P9620-10ML
Dual-luciferase Reporter assay system	Promega	E1910
TM	Synthesized in the lab	Methods section
Polyethyleneimine	Polysciences	24765
FuGene6	Promega	E2691
CellTiter-Glo® 2.0 Cell Viability Assay	Promega	G9241
Acetyl-Lysine Affinity Beads	Cytoskeleton Inc	AAC04
MG132	MedChemExpress	HY-13259
Bafilomycin A1	MedChemExpress	HY-100558
RNeasy Mini Kit	Qiagen	74104
SuperScript VILO cDNA synthesis kit	ThermoFisher	11755050
PowerUp SYBR Green master mix	Appliedbiosystems	A25742
Polybrene	APExBIO	K2701
Pierce™ Protein A/G Magnetic Beads	ThermoFisher	88802
**Software**		
ImageJ	https://imagej.net/ij/	
PyMOL	https://pymol.org	
**Other**		

### Reagents, antibodies, and plasmids

Anti-FLAG affinity gel (A2220), Thapsigargin, cycloheximide, ISRIB, crystal violet, and puromycin were purchased from Sigma-Aldrich. Dual-Luciferase® Reporter Assay System (E1910) and CellTiter-Glo® 2.0 Cell Viability Assay (G9241) were purchased from Promega. TM is synthesized as previously described (Jing et al, 2016). Polyethyleneimine (PEI, 24765) was purchased from Polysciences. Acetyl-Lysine Affinity Beads (AAC04) was purchased from Cytoskeleton, Inc. MG132 and Bafilomycin A1 were purchased from MedChemExpress. Polybrene was purchased from APExBIO (K2701). Pierce™ protein A/G magnetic bead was purchased from Thermo Fisher (88802).The following antibodies were purchased from Cell Signaling Technology: SIRT2 (D4050), SIRT1 (D739), SIRT3 (D22A3), SIRT5 (D8C3), SIRT6 (D8D12), HSP90 (C45G5), ATF4 (D4B8), p-S6K Thr389 (9205), S6K (9202), p-4E-BP1 Ser65 (9451), 4E-BP1 (9452), Ubiquitin (E4I2J), HA-Tag (C29F4), p-eIF2α Ser51 (D9G8), eIF2α (L57A5), Myc-Tag (71D10). Anti-rabbit/mouse IgG HRP-linked Antibody. The following antibodies were from Santa Cruz: SIRT7 (C-3), β-actin (C-4). The following antibodies were from Sigma: anti-Flag M2 antibody conjugated with horseradish peroxidase (A8492), anti-puromycin (12D10).

pcDNA3-myc-CUL3 was a gift from Yue Xiong (Addgene plasmid # 19893), lentiCas9-Blast was a gift from Feng Zhang (Addgene plasmid # 52962). Luciferase constructs were purchased from Promega. The human 302 bp *Sirt2* 5’UTR was amplified by PCR from cDNA library and inserted between HindIII and NcoI in pGL3 vector using primers 5’-AGT CAG AAG CTT CAT TTT CCG GGC GCC-3’ and 5’-AGT CAG CCA TGG GCG CGG-3’. The start codon of *SIRT2* main ORF was fused in frame with the *Firefly luciferase*. To disrupt uORF (133–234), site-directed mutagenesis was carried out using 5’-GTC TGC GGC CGC AAT ATC TGC TGA GAG TTG T-3’ and 5’-TAT TGC GGC CGC AGA CGC GCT TTC GTA CAA C-3’. Flag tagged 4E-BP1 wild-type and KR or KQ mutant vectors were purchased from GenScript. GenScript cloned 4E-BP1 gene into pcDNA3.1 + /C-(K)-DYK vector and then performed mutagenesis to generate the KR and KQ mutants. SIRT2 wild-type expression vector with FLAG tag was made as previously described (Jing et al, 2017). The SIRT2 shRNA resistant mutants were generated through site-directed mutagenesis using the primer pair 5’-tgaaaaatatcATCTTCCCTACCCAGAGG-3’ and 5’-agattatcgtaGAGGCCGGTGGATGG-3’. pFUGW-SIRT2-WT and H187Y lentiviral expression vectors were generated by inserting SIRT2-WT or H187Y shRNA resistant mutant into lentiCas9-Blast vector and replacing the Cas9 gene using NEBuilder^®^ HiFi DNA Assembly Master Mix (NEB, E2621S) with the primer pair 5’-gccagaacacaggaccggttatggcagagccagacccc-3’ and 5’- gagagaagtttgttgcgccgcccttatcgtcgtcatccttgtaatc-3’.

### Immunoprecipitation and immunoblotting

Plasmids encoding Flag or HA tagged proteins were transfected into HEK293 T control and SIRT2 knockdown cells using PEI. For the transfection process, 6 μg of plasmids were first added to 1 mL of DMEM media, then 18 μl of PEI was added to the mixture. The mixture was incubated for 15 min at room temperature before adding to the cells. An empty plasmid was used as a negative control. Cells were collected the day after the transfection and were washed with PBS prior to collecting via centrifugation of 3000 rpm for 5 min at 4 °C. For experiments other than 4E-BP1 and CUL3 co-immunoprecipitation, samples were processed as follows: for each sample, 1 ml of 1% NP40 lysis buffer with final pH 7.4 (150 mM Tris-HCl, pH 8, 150 mM NaCl, 10% glycerol, 1% NP40) was used to lyse the cells at 4 °C for 1 h. 100 μl lysis buffer was used for six-well plate samples. After spinning down the samples with 13,000 rpm for 10 min at 4 °C, the supernatant was collected and normalized after using the Bradford reagent to determine the protein concentration. As input, 40 μl of each normalized sample were collected, mixed with 8 μL of 6× loading dye, boiled at 95 °C for 5 min. For checking endogenous protein, the samples were then proceeded to standard western blot procedure. For the immunoprecipitation experiments, the rest of the normalized sample were incubated with 20 μl of HA, Flag, or acetyl lysine beads at 4 °C overnight. The affinity beads were then washed with IP washing buffer (25 mM Tris, pH 8.0, 150 mM NaCl, 0.2% NP40) for three times and were dried with a 1 ml syringe. Depending on the experiment, 50 μl of 1× loading dye or 35 μl of 2x loading dye were added to the dried beads, and each sample was boiled at 95 °C for 5 min. 4E-BP1 and CUL3 co-immunoprecipitation was followed previous report (Yanagiya et al, 2012) with modifications: Cells were transfected as above. Twenty-four hours post-transfection, cells were treated with 20 mM MG132 for 3 h, collected, and frozen and thawed in lysis buffer (50 mM Tris-HCl, 100 mM KCl, pH 7.5 supplemented with 1 mM PMSF and a protease inhibitor cocktail). Cells were lysed by three freeze-thaw cycles and centrifuged for 10 min at 17,000×*g* to remove debris. Cell lysates were incubated with anti-Myc antibody at 4 C for 3 h, Pierce™ protein A/G magnetic beads were then added and incubated for 1 h at 4 C. Beads were washed four times with lysis buffer and proteins were eluted with 2X loading dye.

Standard western blot analysis was used to analyze the samples. Western blots were performed as previously described (Spiegelman et al, 2018). The proteins were detected using enzyme-linked fluorescence (ECL, Pierce Biotechnology Inc., Clarity Max, Bio-Rad; Immobilon Western Chemiluminescent HRP Substrate, Millipore) on a ChemiDoc (Bio-Rad).

### Cell culture

HEK293T, MCF7, A549, and HeLa cells were grown in DMEM media (Invitrogen) supplemented with 10% (vol/vol) fetal bovine serum (FBS; Invitrogen, Carlsbad, CA). HCT116 cells were cultured in McCoy’s 5 A with 10% FBS. For amino acids depletion experiments, DMEM and McCoy’s 5 A depleted indicated amino acids supplemented with the indicated percent of dialyzed FBS (Invitrogen), while the control groups were cultured in full media also with dialyzed FBS. SIRT2 control and stable knockdown HEK293T, A549, and MCF7 cells were generated as previously described (Spiegelman et al, 2018). A549-shSIRT2-SIRT2-WT and H187Y overexpression cell lines were generated via lentivirus transduction followed by puromycin selection.

### RT-PCR analysis of mRNA levels

Total mRNA was extracted using RNeasy Mini Kit (Qiagen, CA, USA) according to the manufacturer’s instructions and then reverse transcribed to cDNA library using SuperScript Vilo cDNA Synthesis Kit (Thermo Fisher). Real-time PCR were performed on QuantStudio™ 7 Flex Real-Time PCR System using SYBR™ Green PCR Master Mix (Applied Biosystems) and primers for *SIRT2 (all isoforms)*: 5’-TGC GGA ACT TAT TCT CCC AGA-3’, 5’- GAG AGC GAA AGT CGG GGA T-3’; *ATF4*: 5’-GTT CTC CAG CGA CAA GGC TA -3’, 5’-ATC CTG CTT GCT GTT GTT GG-3’; *ACTB* (internal control): 5’-CAT GTA CGT TGC TAT CCA GGC -3’, 5’-CTC CTT AAT GTC ACG CAC GAT-3’.

### Luciferase assay

Firefly and Renilla constructs were co-transfected at 10:1 ratio using FuGene 6 into HeLa cells in full DMEM media. After 12 h the cells were exposed to indicated stress and then collected and analyzed using Dual-Luciferase Reporter Assay System according to manufacturer’s instructions.

### Clonogenic survival assay

One million shCtrl and shSIRT2 cells were seeded in 10-cm plates and exposed to lysine, arginine, and glutamine (KRQ) depletion for seven days. To avoid over-confluency, shCtrl and shSIRT2 cells from control groups were subcultured with the same split ratio. After stress treatment, cells from all groups were subcultured into 6-well plates in complete media with the same split ratio. Proper split ratio was chosen so that there were less than 200 cells in each well. Cells were allowed to recover and grow in six-well plates in normal condition for one week and fixed with methanol and stained with crystal violet.

### CellTiter-Glo® 2.0 cell viability assay

Half million shSIRT2, shSIRT2-SIRT2-WT, shSIRT2-SIRT2-H187Y cells were seeded in six-well plates and exposed to lysine, arginine, and glutamine (KRQ) depletion for 7 days. To avoid over-confluency, cells from the control group (+ QKR) were subcultured with the same split ratio. After stress treatment, cells from all groups were subcultured into opaque 96-well plates in complete media with the same split ratio. Cells were allowed to recover and grow for three days. Cell proliferation was assessed using CellTiter-Glo® 2.0 according to the manufacturer’s instructions.

### Data analysis

Prism (GraphPad Software) was used for data analysis. The paired *t* test, one-sample *t* test, one-way ANOVA, or two-way ANOVA was used to determine statistical significance between two groups. Experimental values are shown as mean ± SD. *P < 0.05, **P < 0.01, ***P < 0.001. ImageJ was used to quantify immunoblotting signals and calculated total colony area. PyMOL was utilized to visualize protein structures.

## Supplementary information

Appendix

Peer Review File

Source data Fig. 1

Source data Fig. 2

Source data Fig. 3

Source data Fig. 4

Source data Fig. 5

Source data Fig. 6

Appendix Figure Source Data

## Data Availability

This study includes no data deposited in external repositories. The source data of this paper are collected in the following database record: biostudies:S-SCDT-10_1038-S44319-026-00803-7.

## References

[CR1] Beretta L, Gingras AC, Svitkin YV, Hall MN, Sonenberg N (1996) Rapamycin blocks the phosphorylation of 4E-BP1 and inhibits cap-dependent initiation of translation. EMBO J 15:658–664 8599949 PMC449984

[CR2] Böhm R, Imseng S, Jakob RP, Hall MN, Maier T, Hiller S (2021) The dynamic mechanism of 4E-BP1 recognition and phosphorylation by mTORC1. Mol Cell 81:2403–2416.e5 33852892 10.1016/j.molcel.2021.03.031

[CR3] Brunn GJ, Hudson CC, Sekulić A, Williams JM, Hosoi H, Houghton PJ, Lawrence JC, Abraham RT (1997) Phosphorylation of the translational repressor PHAS-I by the mammalian target of rapamycin. Science 277:99–101 9204908 10.1126/science.277.5322.99

[CR4] Cantó C, Sauve AA, Bai P (2013) Crosstalk between poly(ADP-ribose) polymerase and sirtuin enzymes. Mol Asp Med 34:1168–1201 10.1016/j.mam.2013.01.004 PMC3676863 23357756

[CR5] Ciechanover A, Schwartz AL (1998) The ubiquitin-proteasome pathway: the complexity and myriad functions of proteins death. Proc Natl Acad Sci USA 95:2727–2730 9501156 10.1073/pnas.95.6.2727 PMC34259

[CR6] Dorrello NV, Peschiaroli A, Guardavaccaro D, Colburn NH, Sherman NE, Pagano M (2006) S6K1- and ßTRCP-mediated degradation of PDCD4 promotes protein translation and cell growth. Science 314:467–471 17053147 10.1126/science.1130276

[CR7] Gingras A-C, Gygi SP, Raught B, Polakiewicz RD, Abraham RT, Hoekstra MF, Aebersold R, Sonenberg N (1999) Regulation of 4E-BP1 phosphorylation: a novel two-step mechanism. Genes Dev 13:1422–1437 10364159 10.1101/gad.13.11.1422 PMC316780

[CR8] Gingras A-C, Kennedy SG, O’Leary MA, Sonenberg N, Hay N (1998) 4E-BP1, a repressor of mRNA translation, is phosphorylated and inactivated by the Akt(PKB) signaling pathway. Genes Dev 12:502–513 9472019 10.1101/gad.12.4.502 PMC316523

[CR9] Gingras AC, Svitkin Y, Belsham GJ, Pause A, Sonenberg N (1996) Activation of the translational suppressor 4E-BP1 following infection with encephalomyocarditis virus and poliovirus. Proc Natl Acad Sci USA 93:5578–5583 8643618 10.1073/pnas.93.11.5578 PMC39289

[CR10] Gottlieb S, Esposito RE (1989) A new role for a yeast transcriptional silencer gene, SIR2, in regulation of recombination in ribosomal DNA. Cell 56:771–776 2647300 10.1016/0092-8674(89)90681-8

[CR11] Hanahan D, Weinberg RA (2011) Hallmarks of Cancer: The Next Generation. Cell 144:646–674 21376230 10.1016/j.cell.2011.02.013

[CR12] Harding HP, Novoa I, Zhang Y, Zeng H, Wek R, Schapira M, Ron D (2000) Regulated translation initiation controls stress-induced gene expression in mammalian cells. Mol Cell 6:1099–1108 11106749 10.1016/s1097-2765(00)00108-8

[CR13] Hinnebusch AG (2014) The scanning mechanism of eukaryotic translation initiation. Annu Rev Biochem 83:779–812 24499181 10.1146/annurev-biochem-060713-035802

[CR14] Hou D, Yu T, Lu X, Hong JY, Yang M, Zi Y, Ho TT, Lin H (2024) Sirt2 inhibition improves gut epithelial barrier integrity and protects mice from colitis. Proc Natl Acad Sci USA 121:e2319833121 38648480 10.1073/pnas.2319833121 PMC11066986

[CR15] Ingolia NT, Lareau LF, Weissman JS (2011) Ribosome profiling of mouse embryonic stem cells reveals the complexity and dynamics of mammalian proteomes. Cell 147:789–802 22056041 10.1016/j.cell.2011.10.002 PMC3225288

[CR16] Inoue T, Hiratsuka M, Osaki M, Yamada H, Kishimoto I, Yamaguchi S, Nakano S, Katoh M, Ito H, Oshimura M (2007) SIRT2, a tubulin deacetylase, acts to block the entry to chromosome condensation in response to mitotic stress. Oncogene 26:945–957 16909107 10.1038/sj.onc.1209857

[CR17] Ishfaq M, Maeta K, Maeda S, Natsume T, Ito A, Yoshida M (2012) Acetylation regulates subcellular localization of eukaryotic translation initiation factor 5A (eIF5A). FEBS Lett 586:3236–3241 22771473 10.1016/j.febslet.2012.06.042

[CR18] Jiang W, Wang S, Xiao M, Lin Y, Zhou L, Lei Q, Xiong Y, Guan K-L, Zhao S (2011) Acetylation regulates gluconeogenesis by promoting PEPCK1 degradation via recruiting the UBR5 ubiquitin ligase. Mol Cell 43:33–44 21726808 10.1016/j.molcel.2011.04.028 PMC3962309

[CR19] Jing E, Gesta S, Kahn CR (2007) SIRT2 regulates adipocyte differentiation through FoxO1 acetylation/deacetylation. Cell Metab 6:105–114 17681146 10.1016/j.cmet.2007.07.003 PMC2083635

[CR20] Jing H, Hu J, He B, Negrón Abril YL, Stupinski J, Weiser K, Carbonaro M, Chiang Y-L, Southard T, Giannakakou P et al (2016) A SIRT2-selective inhibitor promotes c-Myc oncoprotein degradation and exhibits broad anticancer activity. Cancer Cell 29:297–310 26977881 10.1016/j.ccell.2016.02.007 PMC4811675

[CR21] Jing H, Zhang X, Wisner SA, Chen X, Spiegelman NA, Linder ME, Lin H (2017) SIRT2 and lysine fatty acylation regulate the transforming activity of K-Ras4a. eLife 6:e32436 29239724 10.7554/eLife.32436 PMC5745086

[CR22] Jobava R, Mao Y, Guan B-J, Hu D, Krokowski D, Chen C-W, Shu XE, Chukwurah E, Wu J, Gao Z et al (2021) Adaptive translational pausing is a hallmark of the cellular response to severe environmental stress. Mol Cell 81:4191–4208.e8 34686314 10.1016/j.molcel.2021.09.029 PMC8559772

[CR23] Karim R, Teng W, Lin H (2024) SIRT2-mediated ACSS2 K271 deacetylation suppresses lipogenesis under nutrient stress. eLife 13:RP97019 10.7554/eLife.97019 PMC12058118 40331334

[CR24] Kosciuk T, Price IR, Zhang X, Zhu C, Johnson KN, Zhang S, Halaby SL, Komaniecki GP, Yang M, DeHart CJ et al (2020) NMT1 and NMT2 are lysine myristoyltransferases regulating the ARF6 GTPase cycle. Nat Commun 11:1067 32103017 10.1038/s41467-020-14893-x PMC7044312

[CR25] Lee AS, Jung YJ, Kim D, Nguyen-Thanh T, Kang KP, Lee S, Park SK, Kim W (2014) SIRT2 ameliorates lipopolysaccharide-induced inflammation in macrophages. Biochem Biophys Res Commun 450:1363–1369 25003320 10.1016/j.bbrc.2014.06.135

[CR26] Liu B, Qian S (2014) Translational reprogramming in cellular stress response. WIREs RNA 5:301–305 24375939 10.1002/wrna.1212 PMC3991730

[CR27] Luo J, Solimini NL, Elledge SJ (2009) Principles of cancer therapy: oncogene and non-oncogene addiction. Cell 136:823–837 19269363 10.1016/j.cell.2009.02.024 PMC2894612

[CR28] Ma XM, Yoon S-O, Richardson CJ, Jülich K, Blenis J (2008) SKAR links pre-mRNA splicing to mTOR/S6K1-mediated enhanced translation efficiency of spliced mRNAs. Cell 133:303–313 18423201 10.1016/j.cell.2008.02.031

[CR29] Nguyen HG, Conn CS, Kye Y, Xue L, Forester CM, Cowan JE, Hsieh AC, Cunningham JT, Truillet C, Tameire F et al (2018) Development of a stress response therapy targeting aggressive prostate cancer. Sci Transl Med 10:eaar2036 29720449 10.1126/scitranslmed.aar2036 PMC6045425

[CR30] North BJ, Verdin E (2004) Sirtuins: Sir2-related NAD-dependent protein deacetylases. Genome Biol 5:224 15128440 10.1186/gb-2004-5-5-224 PMC416462

[CR31] Outeiro TF, Kontopoulos E, Altmann SM, Kufareva I, Strathearn KE, Amore AM, Volk CB, Maxwell MM, Rochet J-C, McLean PJ et al (2007) Sirtuin 2 inhibitors rescue α-synuclein-mediated toxicity in models of Parkinson’s disease. Science 317:516–519 17588900 10.1126/science.1143780

[CR32] Pakos-Zebrucka K, Koryga I, Mnich K, Ljujic M, Samali A, Gorman AM (2016) The integrated stress response. EMBO Rep 17:1374–1395 27629041 10.15252/embr.201642195 PMC5048378

[CR33] Palam LR, Baird TD, Wek RC (2011) Phosphorylation of eIF2 facilitates ribosomal bypass of an inhibitory upstream ORF to enhance CHOP translation. J Biol Chem 286:10939–10949 21285359 10.1074/jbc.M110.216093 PMC3064149

[CR34] Rothgiesser KM, Erener S, Waibel S, Lüscher B, Hottiger MO (2010) SIRT2 regulates NF-κB-dependent gene expression through deacetylation of p65 Lys310. J Cell Sci 123:4251–4258 21081649 10.1242/jcs.073783

[CR35] Roux PP, Topisirovic I (2018) Signaling pathways involved in the regulation of mRNA translation. Mol Cell Biol 38:e00070–18 29610153 10.1128/MCB.00070-18 PMC5974435

[CR36] Saxton RA, Sabatini DM (2017) mTOR signaling in growth, metabolism, and disease. Cell 168:960–976 28283069 10.1016/j.cell.2017.02.004 PMC5394987

[CR37] Schartner E, Sabbir MG, Saleh A, Silva RV, Roy Chowdhury S, Smith DR, Fernyhough P (2018) High glucose concentration suppresses a SIRT2 regulated pathway that enhances neurite outgrowth in cultured adult sensory neurons. Exp Neurol 309:134–147 30102915 10.1016/j.expneurol.2018.08.001

[CR38] Schmidt EK, Clavarino G, Ceppi M, Pierre P (2009) SUnSET, a nonradioactive method to monitor protein synthesis. Nat Methods 6:275–277 19305406 10.1038/nmeth.1314

[CR39] Shrama A, Zi Y, Pandit AS, Jha K, Sinha VK, Nagesh D, Shivanaiah B, Ravi V, Ghosh S, Khan D et al (2026) Sirtuin 2 inhibits global protein synthesis via Rheb-GTPase degradation. EMBO Rep. 10.1038/s44319-026-00724-5 10.1038/s44319-026-00724-5 PMC13261059 41813942

[CR40] Singh P, Hanson PS, Morris CM (2017) Sirtuin-2 protects neural cells from oxidative stress and is elevated in neurodegeneration. Parkinson’s Dis 2017:1–17 10.1155/2017/2643587 PMC5467326 28634568

[CR41] Spiegelman NA, Price IR, Jing H, Wang M, Yang M, Cao J, Hong JY, Zhang X, Aramsangtienchai P, Sadhukhan S et al (2018) Direct comparison of SIRT2 inhibitors: potency, specificity, activity-dependent inhibition, and on-target anticancer activities. ChemMedChem 13:1890–1894 30058233 10.1002/cmdc.201800391 PMC6402572

[CR42] Spiegelman NA, Zhang X, Jing H, Cao J, Kotliar IB, Aramsangtienchai P, Wang M, Tong Z, Rosch KM, Lin H (2019) SIRT2 and lysine fatty acylation regulate the activity of RalB and cell migration. ACS Chem Biol 14:2014–2023 31433161 10.1021/acschembio.9b00492 PMC6893912

[CR43] Teng Y-B, Jing H, Aramsangtienchai P, He B, Khan S, Hu J, Lin H, Hao Q (2015) Efficient demyristoylase activity of SIRT2 revealed by kinetic and structural studies. Sci Rep 5:8529 25704306 10.1038/srep08529 PMC4894398

[CR44] Vattem KM, Wek RC (2004) Reinitiation involving upstream ORFs regulates *ATF4* mRNA translation in mammalian cells. Proc Natl Acad Sci USA 101:11269–11274 15277680 10.1073/pnas.0400541101 PMC509193

[CR45] Wang F, Chan C-H, Chen K, Guan X, Lin H-K, Tong Q (2012) Deacetylation of FOXO3 by SIRT1 or SIRT2 leads to Skp2-mediated FOXO3 ubiquitination and degradation. Oncogene 31:1546–1557 21841822 10.1038/onc.2011.347

[CR46] Wang F, Nguyen M, Qin FX, Tong Q (2007) SIRT2 deacetylates FOXO3a in response to oxidative stress and caloric restriction. Aging Cell 6:505–514 17521387 10.1111/j.1474-9726.2007.00304.x

[CR47] Wang F, Tong Q (2009) SIRT2 suppresses adipocyte differentiation by deacetylating FOXO1 and enhancing FOXO1’s repressive interaction with PPARγ. MBoC 20:801–808 19037106 10.1091/mbc.E08-06-0647 PMC2633403

[CR48] Wang M, Zhang Y, Komaniecki GP, Lu X, Cao J, Zhang M, Yu T, Hou D, Spiegelman NA, Yang M et al (2022) Golgi stress induces SIRT2 to counteract Shigella infection via defatty-acylation. Nat Commun 13:4494 35918380 10.1038/s41467-022-32227-x PMC9345896

[CR49] Watanabe H, Inaba Y, Kimura K, Matsumoto M, Kaneko S, Kasuga M, Inoue H (2018) Sirt2 facilitates hepatic glucose uptake by deacetylating glucokinase regulatory protein. Nat Commun 9:30 29296001 10.1038/s41467-017-02537-6 PMC5750207

[CR50] Wolfson RL, Chantranupong L, Saxton RA, Shen K, Scaria SM, Cantor JR, Sabatini DM (2016) Sestrin2 is a leucine sensor for the mTORC1 pathway. Science 351:43–48 26449471 10.1126/science.aab2674 PMC4698017

[CR51] Wolfson RL, Sabatini DM (2017) The dawn of the age of amino acid sensors for the mTORC1 pathway. Cell Metab 26:301–309 28768171 10.1016/j.cmet.2017.07.001 PMC5560103

[CR52] Yanagiya A, Suyama E, Adachi H, Svitkin YV, Aza-Blanc P, Imataka H, Mikami S, Martineau Y, Ronai ZA, Sonenberg N (2012) Translational homeostasis via the mRNA cap-binding protein, eIF4E. Mol Cell 46:847–858 22578813 10.1016/j.molcel.2012.04.004 PMC4085128

[CR53] Ye J, Kumanova M, Hart LS, Sloane K, Zhang H, De Panis DN, Bobrovnikova-Marjon E, Diehl JA, Ron D, Koumenis C (2010) The GCN2-ATF4 pathway is critical for tumour cell survival and proliferation in response to nutrient deprivation. EMBO J 29:2082–2096 20473272 10.1038/emboj.2010.81 PMC2892366

[CR54] Zhang H, Head PE, Daddacha W, Park S-H, Li X, Pan Y, Madden MZ, Duong DM, Xie M, Yu B et al (2016) ATRIP deacetylation by SIRT2 drives ATR checkpoint activation by promoting binding to RPA-ssDNA. Cell Rep 14:1435–1447 26854234 10.1016/j.celrep.2016.01.018 PMC4758896

[CR55] Zhang H, Park S-H, Pantazides BG, Karpiuk O, Warren MD, Hardy CW, Duong DM, Park S-J, Kim H-S, Vassilopoulos A et al (2013) SIRT2 directs the replication stress response through CDK9 deacetylation. Proc Natl Acad Sci USA 110:13546–13551 23898190 10.1073/pnas.1301463110 PMC3746840

