# Supplementary material for: SIRT2 mediates integrated stress response by deacetylating and stabilizing 4E-BP1 to suppress translation
Source: EMBO Rep. 2026 May 18;27(11):3035–49. doi: 10.1038/s44319-026-00803-7 (PMC13260904; doi:10.1038/s44319-026-00803-7)
Supplement: Supplementary file 1 — Appendix [file 44319_2026_803_MOESM1_ESM.pdf]

Appendix to

SIRT2 Mediates Integrated Stress Response by Deacetylating and Stabilizing 4E-BP1 to Suppress Translation

Table of Contents

Appendix Figure S1. Amino acid starvation conditions that increase SIRT2 also induce eIF2α phosphorylation........ 2

Appendix Figure S2. SIRT2 and ATF4 levels change upon ISRIB treatment with or without amino acid starvation........ 3

Appendix Figure S3. 5'UTR from the human Sirt2 variant 1........ 4

Appendix Figure S4. SIRT2 5'UTR luciferase reporter assay with uORF mutations........ 5

Appendix Figure S5. Ribosome footprint signal over the SIRT2 uORF region in multiple public ribosome profiling datasets........ 6

Appendix Figure S6. Representative GWIPS-viz initiation- and elongation-view tracks over the SIRT2 uORF........ 7

Appendix Figure S7. SIRT2 inhibition promotes protein translation, and SIRT2 overexpression downregulates protein translation........ 8

Appendix Figure S8. S6K levels in control and SIRT2 knockdown A549 and HEK293T cells........ 9

Appendix Figure S9. Acetylation IP of the endogenous 4EBP1 with SIRT2 inhibitor TM treatment........ 10

Appendix Figure S10. SIRT2 deacetylates 4EBP1 only at K69........ 11

Appendix Figure S11. 4E-BP1 WT binds more strongly to CUL3 than K69R........ 12

Appendix Figure S12. The effect of rapamycin on 4E-BP1 acetylation........ 13

Appendix Figure S13. Modeled structure of p-4E-BP1 (T37/T46) in complex with mTORC1........ 14

Appendix Table S1. Summary of cell lines used in this study........ 15

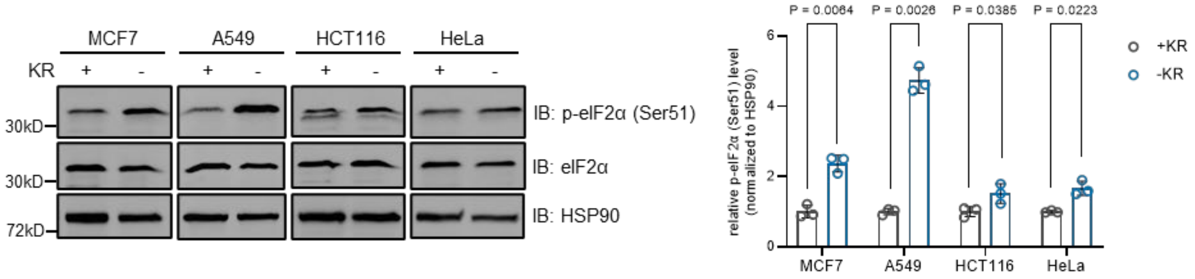

Appendix Figure S1. Amino acid starvation conditions that increase SIRT2 also induce eIF2α phosphorylation. MCF7, A549, HCT116, and HeLa cells were cultured with or without KR depletion for 24 hours. The amino acid limitation-induced p-eIF2α (Ser51) level increases were observed across all tested cell lines. Representative data from three biological replicates. *P* values are determined using 2way ANOVA. *P* = 0.0064 (MCF7), *P* = 0.0026 (A549), *P* = 0.0385 (HCT116), *P* = 0.0223 (HeLa).

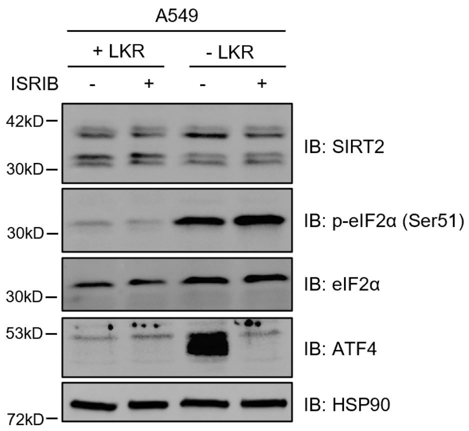

Appendix Figure S2. SIRT2 and ATF4 levels change upon ISRIB treatment with or without amino acid starvation. A549 cells were cultured with or without LKR depletion under 2 μM ISRIB for 24 hours. The amino acid limitation-induced SIRT2 protein level increases was diminished when treated with the ISR inhibitor ISRIB. Representative data from three biological replicates.

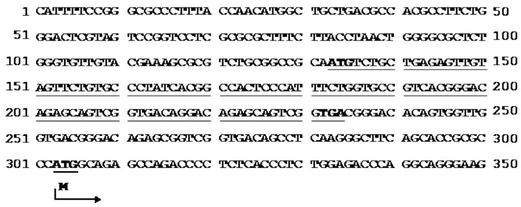

Appendix Figure S3. 5’UTR from the human SIRT2 variant 1.

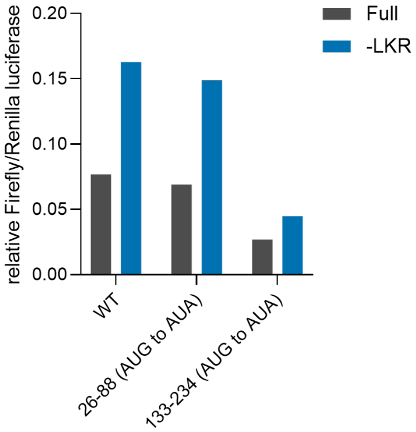

Appendix Figure S4. SIRT2 5'UTR luciferase reporter assay with uORF mutations. Firefly luciferase constructs containing either the WT SIRT2 5'UTR, a uORF 26-88 AUG mutant, or a uORF 133-234 AUG mutant were transfected into HeLa cells together with a Renilla luciferase control plasmid. The ratio of Firefly luciferase activity over Renilla luciferase activity was assessed with or without LKR (leucine, lysine, and arginine) starvation. Mutation of the 133-234 uORF, but not the 26-88 uORF, markedly reduced reporter activity.

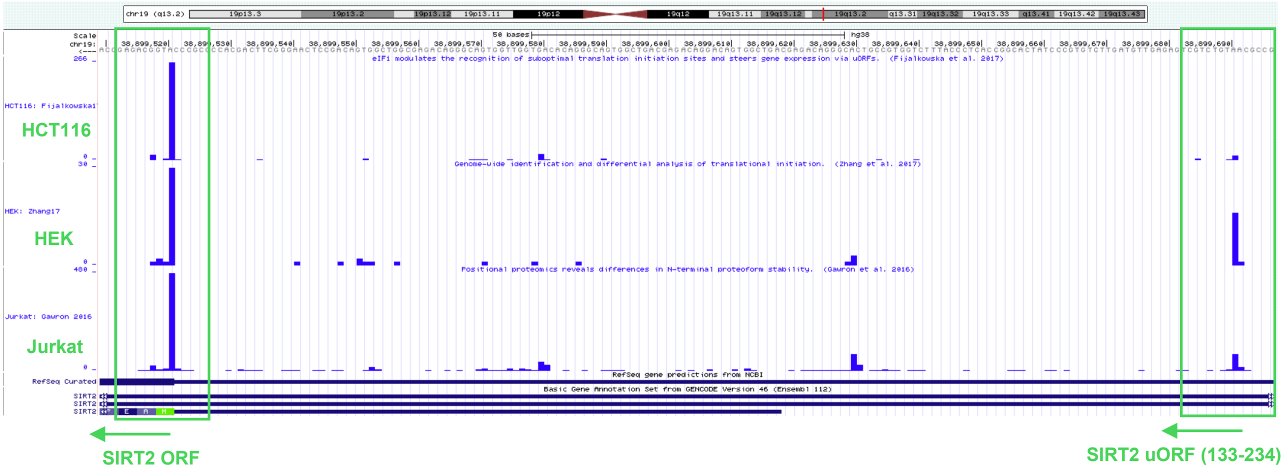

Appendix Figure S5. Ribosome footprint signal over the SIRT2 uORF region in multiple public ribosome profiling datasets. Ribosome profiling tracks from independent studies were visualized in the GWIPS-viz genome browser and aligned to the human SIRT2 transcript. Ribosome footprint coverage is detected over the annotated SIRT2 uORF (133–234), consistent with ribosome occupancy on this uORF. Datasets shown include HCT116 study (doi:10.1093/nar/gkx469), HEK study (doi:10.1038/s41467-017-01981-8), and Jurkat study (doi:10.15252/msb.20156662).

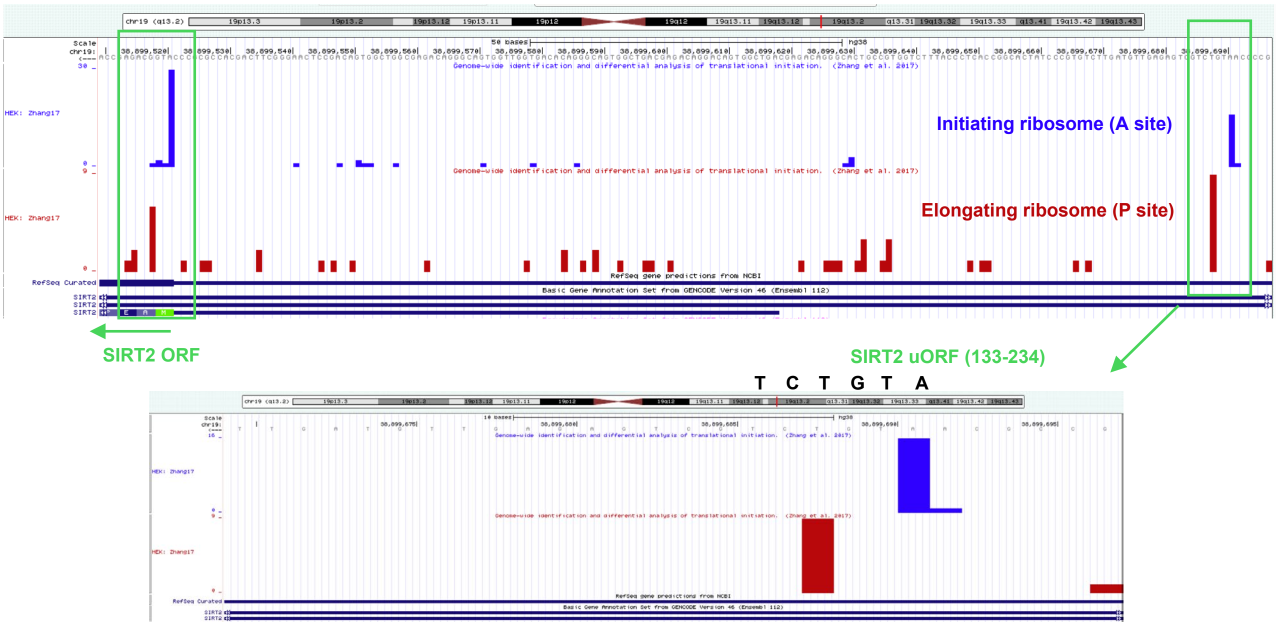

Appendix Figure S6. Representative GWIPS-viz initiation- and elongation-view tracks over the SIRT2 uORF. Shown is an example dataset ("HEK study" from Appendix Figure S12) visualized in the GWIPS-viz genome browser (Zhang et al., doi:10.1038/s41467-017-01981-8). The A-site (initiation-focused) and P-site (elongation) tracks display signal at/near the uORF (133–234) AUG and downstream within the uORF, respectively.

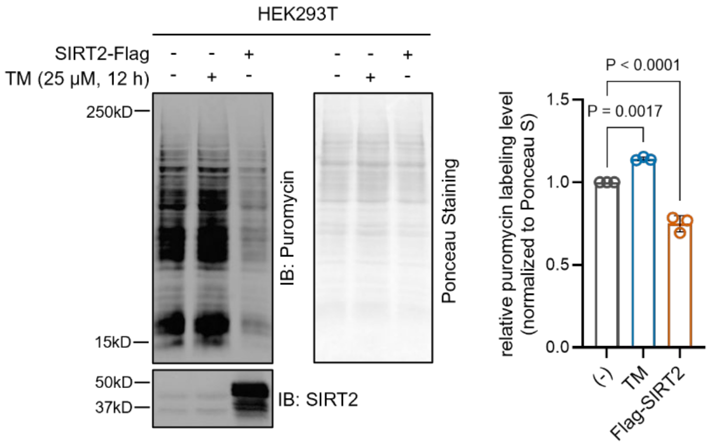

Appendix Figure S7. SIRT2 inhibition promotes protein translation, and SIRT2 overexpression downregulates protein translation. HEK293T cells were either treated with 25 μM of SIRT2 inhibitor TM for 12 hours or overexpressed with Flag-tagged SIRT2. Cells were treated with10 μg/ml puromycin for 10 minutes before collecting. Representative data from three biologically independent experiments. Data with error bars are mean ± s.d. *P* values are determined using one-way ANOVA. P = 0.0017 (TM), P = 9.62 x 10^{-5} (Flag-SIRT2).

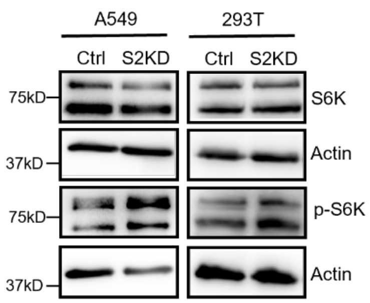

Appendix Figure S8. S6K levels in control and SIRT2 knockdown A549 and HEK293T cells. Protein levels were measured from whole cell lysate using western blot.

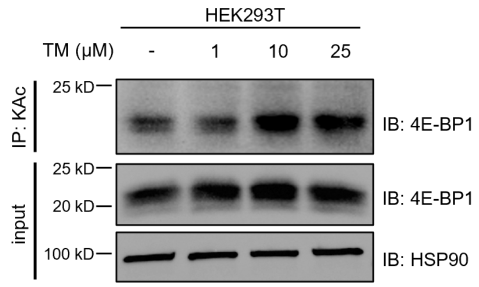

Appendix Figure S9. Acetylation IP of the endogenous 4EBP1 with SIRT2 inhibitor TM treatment. The acetylation levels of 4EBP1 increased upon TM treatment in a dose-dependent manner. Representative results from two biological replicates.

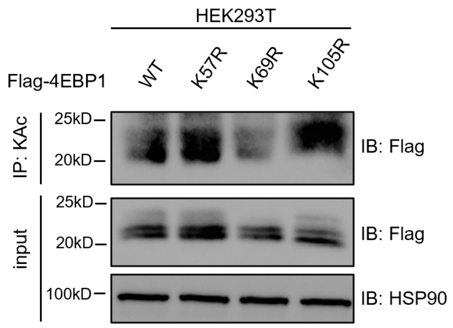

Appendix Figure S10. SIRT2 deacetylates 4EBP1 only at K69. SIRT2 knockdown HEK293T cells were transfected with Flag-tagged 4EBP1, Flag-tagged 4EBP1 K57R, K69R, or K105R mutant. Acetylated proteins were pulled down with acetyl lysine IP beads. Acetylation was detected using western blot.

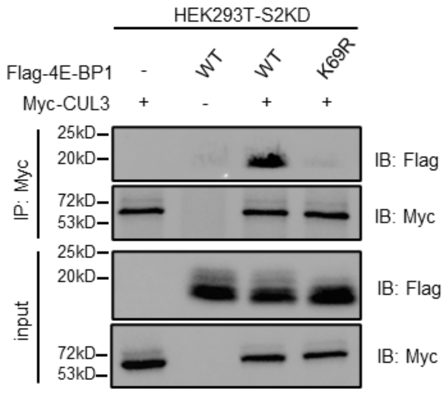

Appendix Figure S11. 4E-BP1 WT binds more strongly to CUL3 than K69R. HEK293T-SIRT2 KD cells were transfected with Flag-4E-BP1, Myc-CUL3, or both. Cells were treated with MG132 for 3 hours, collected and lysed via freeze-thaw cycles. Myc-CUL3 were then immunoprecipitated, and the interactions between 4E-BP1 and CUL3 were examined via western blotting. Representative data from three biological replicates.

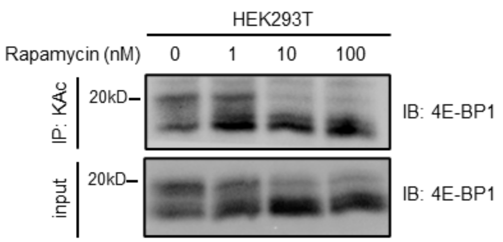

Appendix Figure S12. The effect of rapamycin on 4E-BP1 acetylation. HEK293T cells were treated with rapamycin at indicated concentrations and the acetylation levels of 4E-BP1 were assessed via acetyl-lysine IP. Rapamycin treatment did not significantly affect the acetylation of 4E-BP1. Representative results from two independent biological replicates.

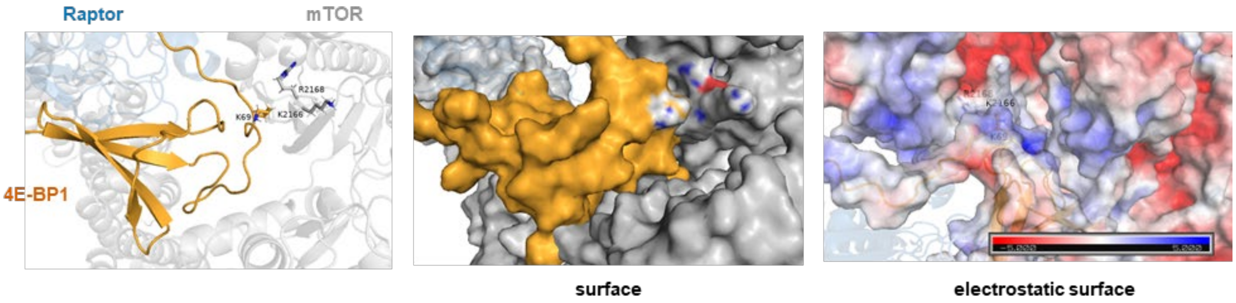

Appendix Figure S13. Modeled structure of p-4E-BP1 (T37/T46) in complex with mTORC1. Left: cartoon with K69 (4E-BP1), K2166/R2168 (mTOR) highlighted as sticks. Middle: protein surface. Right: Calculated electrostatic surface in PyMOL. 4E-BP1 (brightorange), mTOR (gray), and Raptor (skyblue).

Appendix Table S1. Summary of cell lines used in this study.

		HEK293T	A549	HeLa	MCF7	HCT116
Figure 1	1A/1B	■	■	■	■	■
	1C	■				
	1D/1E				■	
	1F/1G				■	
Figure 2	2A		■			
	2B		■			
	2C		■			
Figure 3	3A			■		
	3B			■		
	3C			■		
Figure 4	4A				■	
	4B	■				
	4C	■	■			
	4D/4E	■				
	4F/4G	■				
	4H/4I	■				
Figure 5	5A	■				
	5B/5C	■				
	5D/5E	■				
	5F/5G	■				
	5H/5I	■				
Appendix Figure S1		■	■	■	■	■
Appendix Figure S2			■			
Appendix Figure S3				■		
Appendix Figure S4		■				
Appendix Figure S5		■	■			
Appendix Figure S6		■				
Appendix Figure S7		■				
Appendix Figure S8		■				
Appendix Figure S9			■			
