# Supplementary figures and images for: SIRT2 mediates integrated stress response by deacetylating and stabilizing 4E-BP1 to suppress translation (part 1 of 2)
Source: EMBO Rep. 2026 May 18;27(11):3035–49. doi: 10.1038/s44319-026-00803-7 (PMC13260904; doi:10.1038/s44319-026-00803-7)

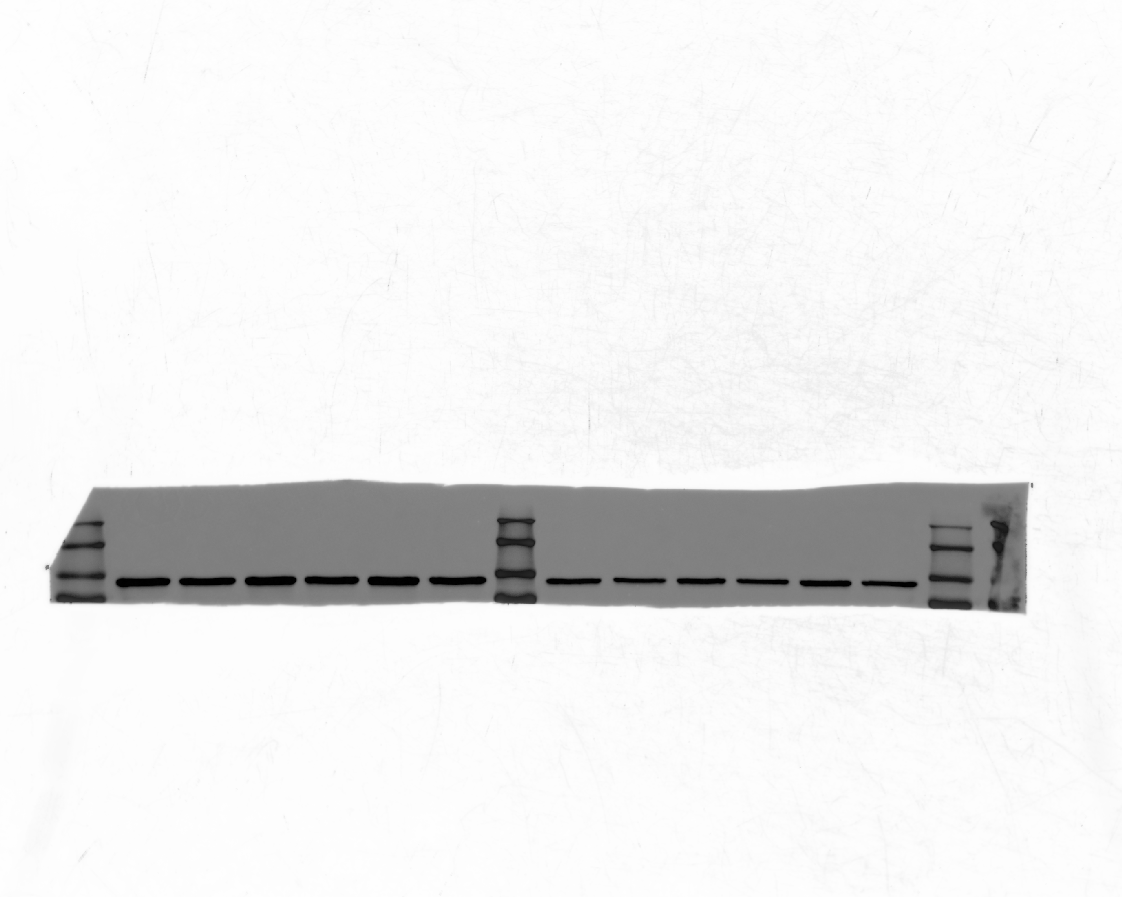

Supplement: Supplementary file 3 — Source data Fig. 1 [file 44319_2026_803_MOESM3_ESM.zip › Figure 1/1A/western A549 and HCT116 HSP90.tif]

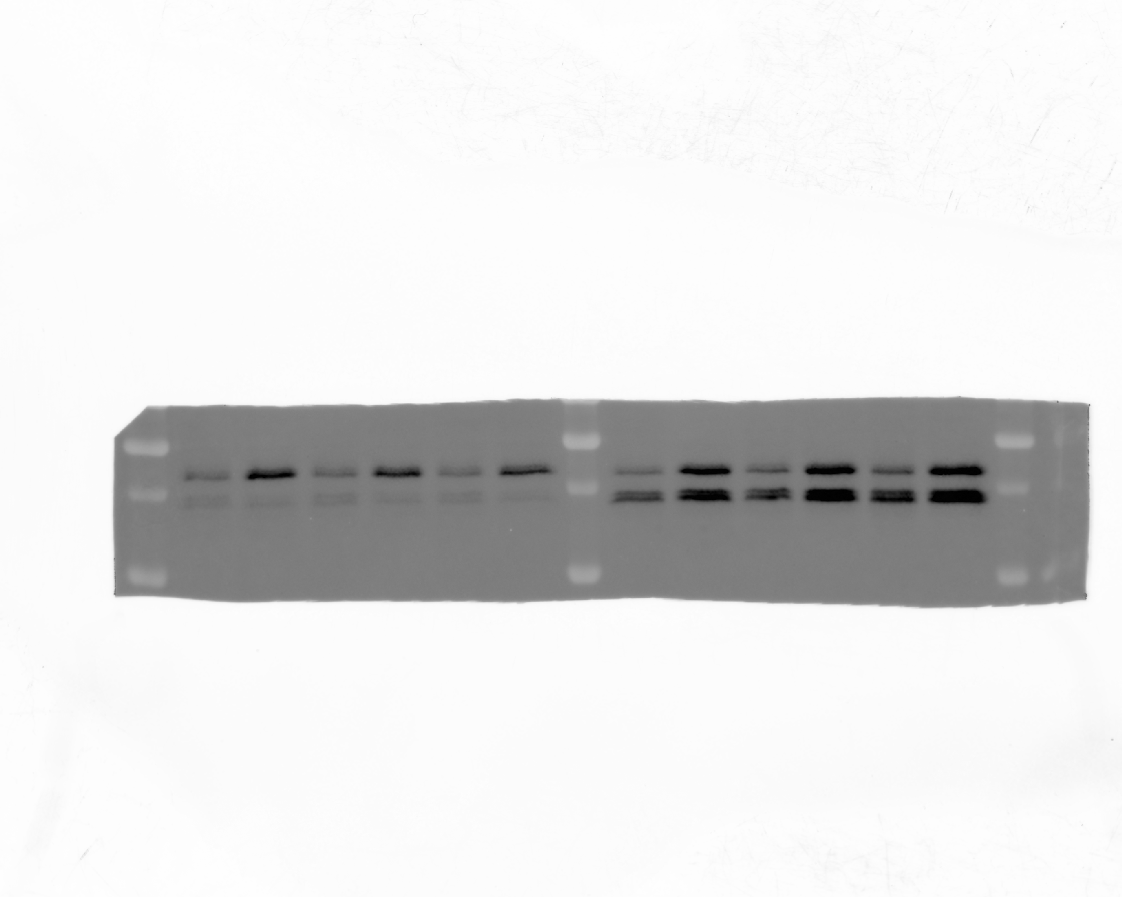

Supplement: Supplementary file 3 — Source data Fig. 1 [file 44319_2026_803_MOESM3_ESM.zip › Figure 1/1A/western A549 and HCT116 SIRT2.tif]

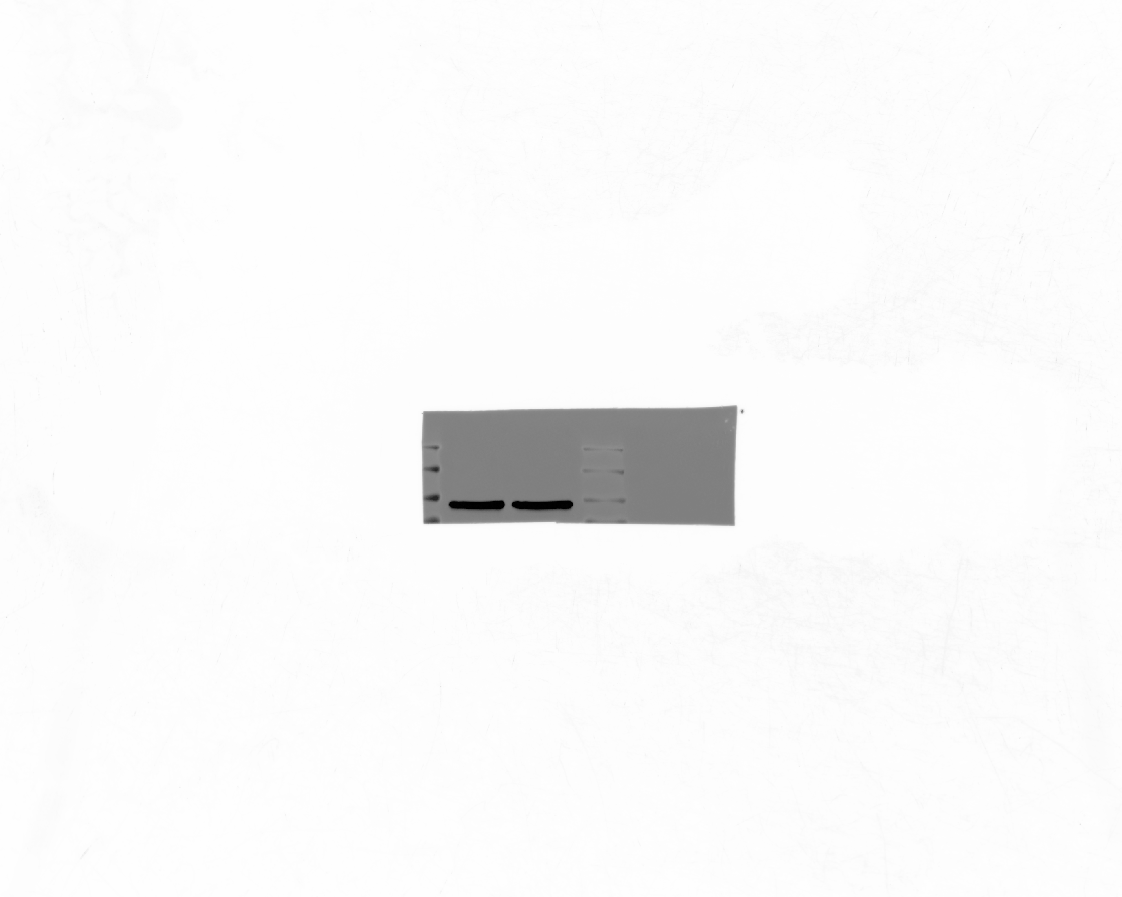

Supplement: Supplementary file 3 — Source data Fig. 1 [file 44319_2026_803_MOESM3_ESM.zip › Figure 1/1A/western HeLa HSP90.tif]

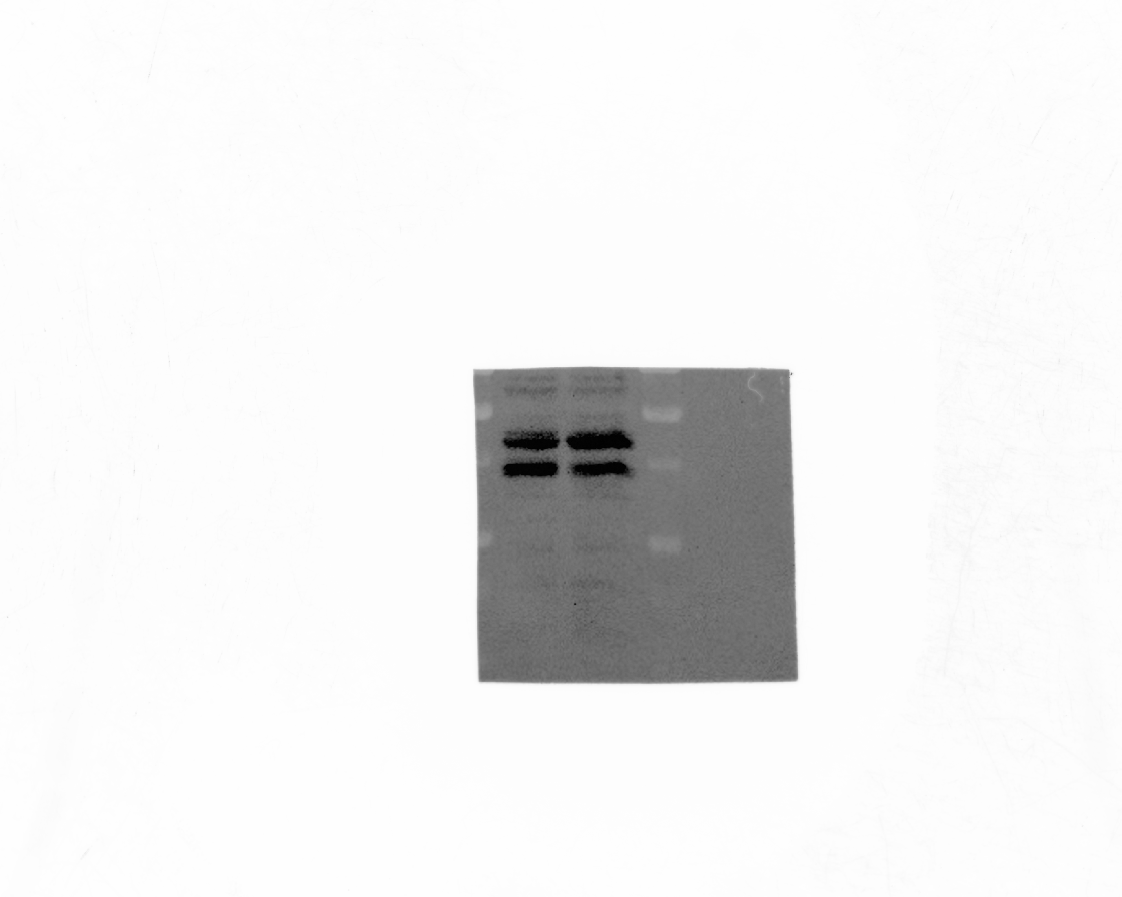

Supplement: Supplementary file 3 — Source data Fig. 1 [file 44319_2026_803_MOESM3_ESM.zip › Figure 1/1A/western HeLa SIRT2.tif]

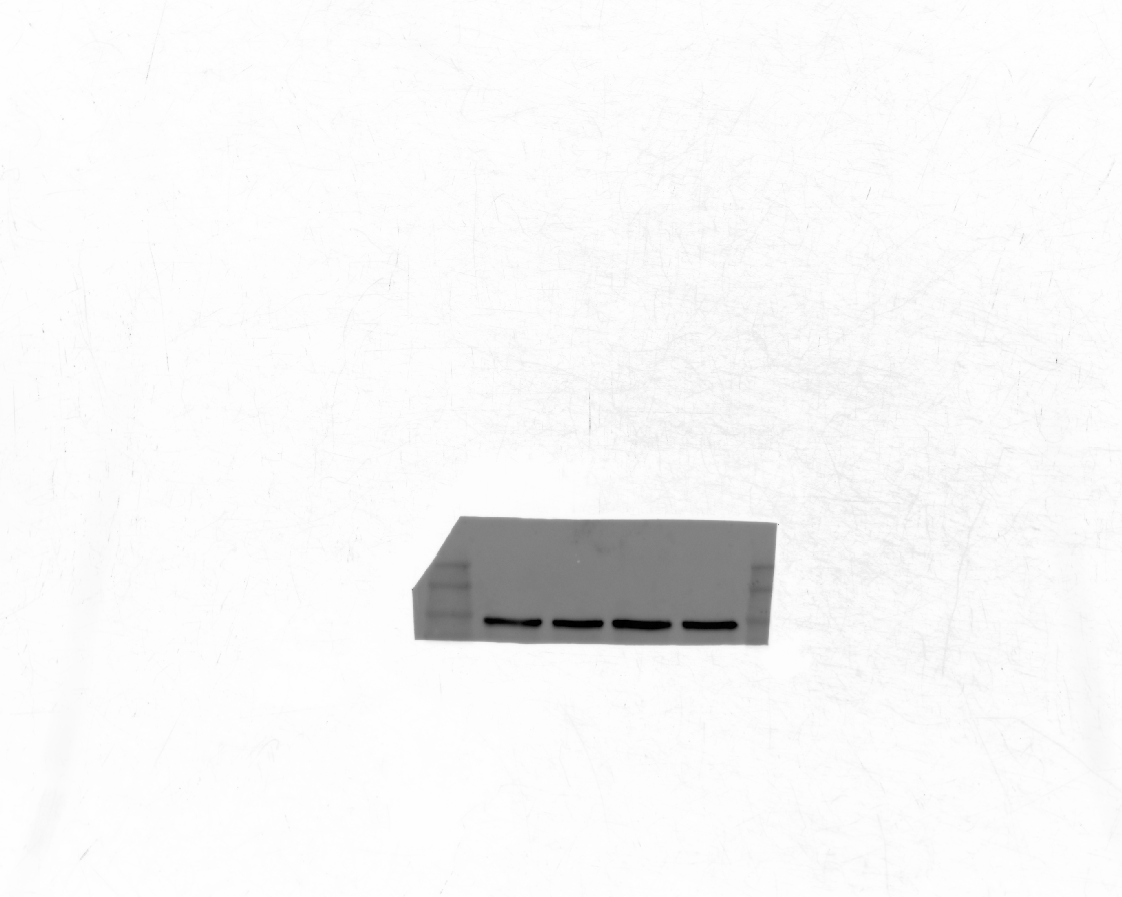

Supplement: Supplementary file 3 — Source data Fig. 1 [file 44319_2026_803_MOESM3_ESM.zip › Figure 1/1A/western MCF7 HSP90.tif]

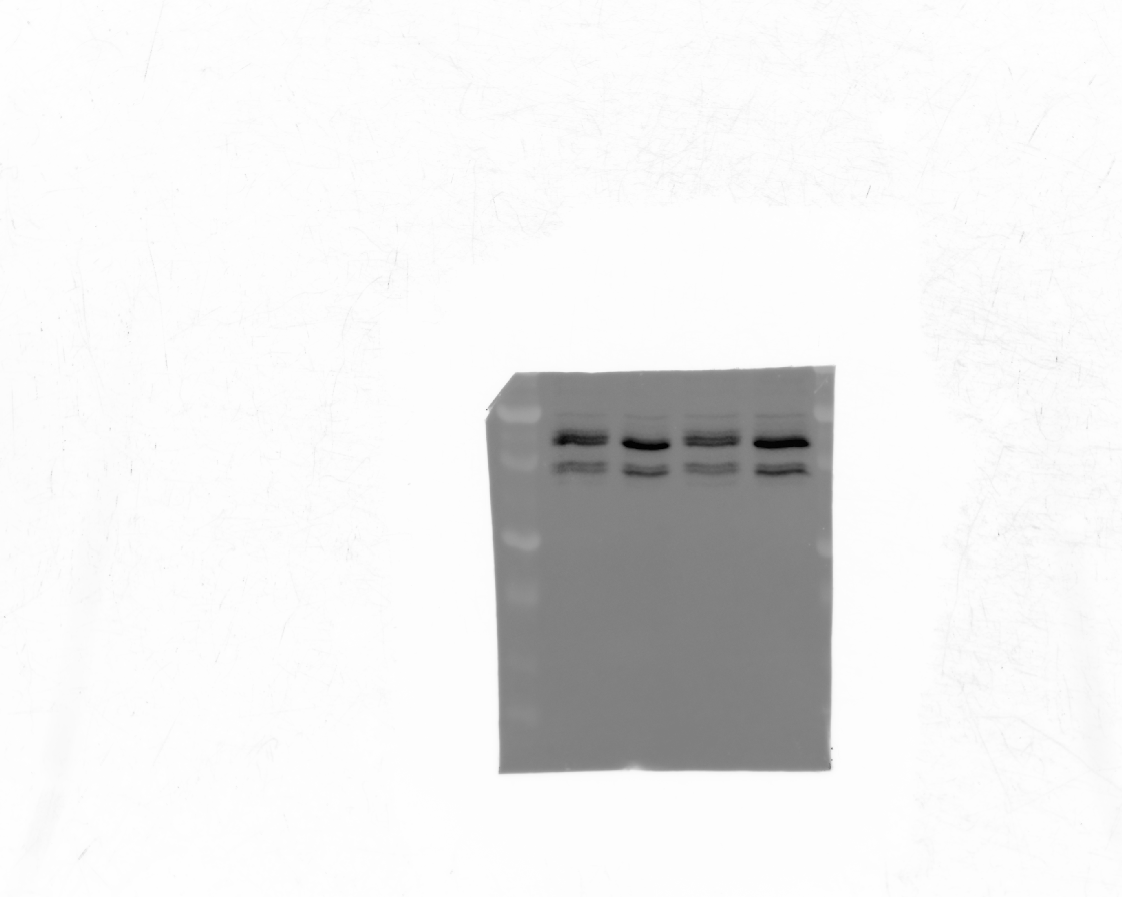

Supplement: Supplementary file 3 — Source data Fig. 1 [file 44319_2026_803_MOESM3_ESM.zip › Figure 1/1A/western MCF7 SIRT2.tif]

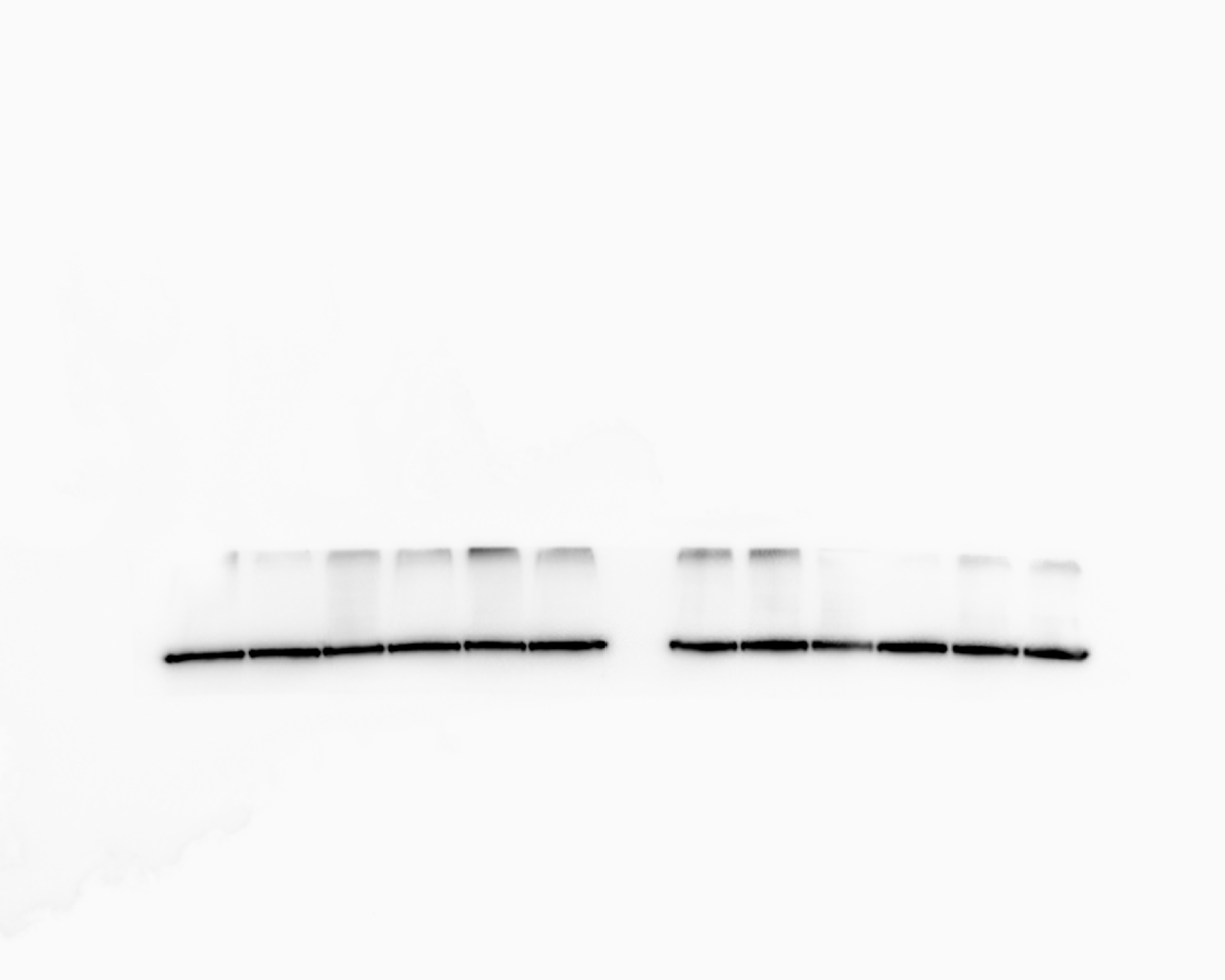

Supplement: Supplementary file 3 — Source data Fig. 1 [file 44319_2026_803_MOESM3_ESM.zip › Figure 1/1C/western HSP90.tif]

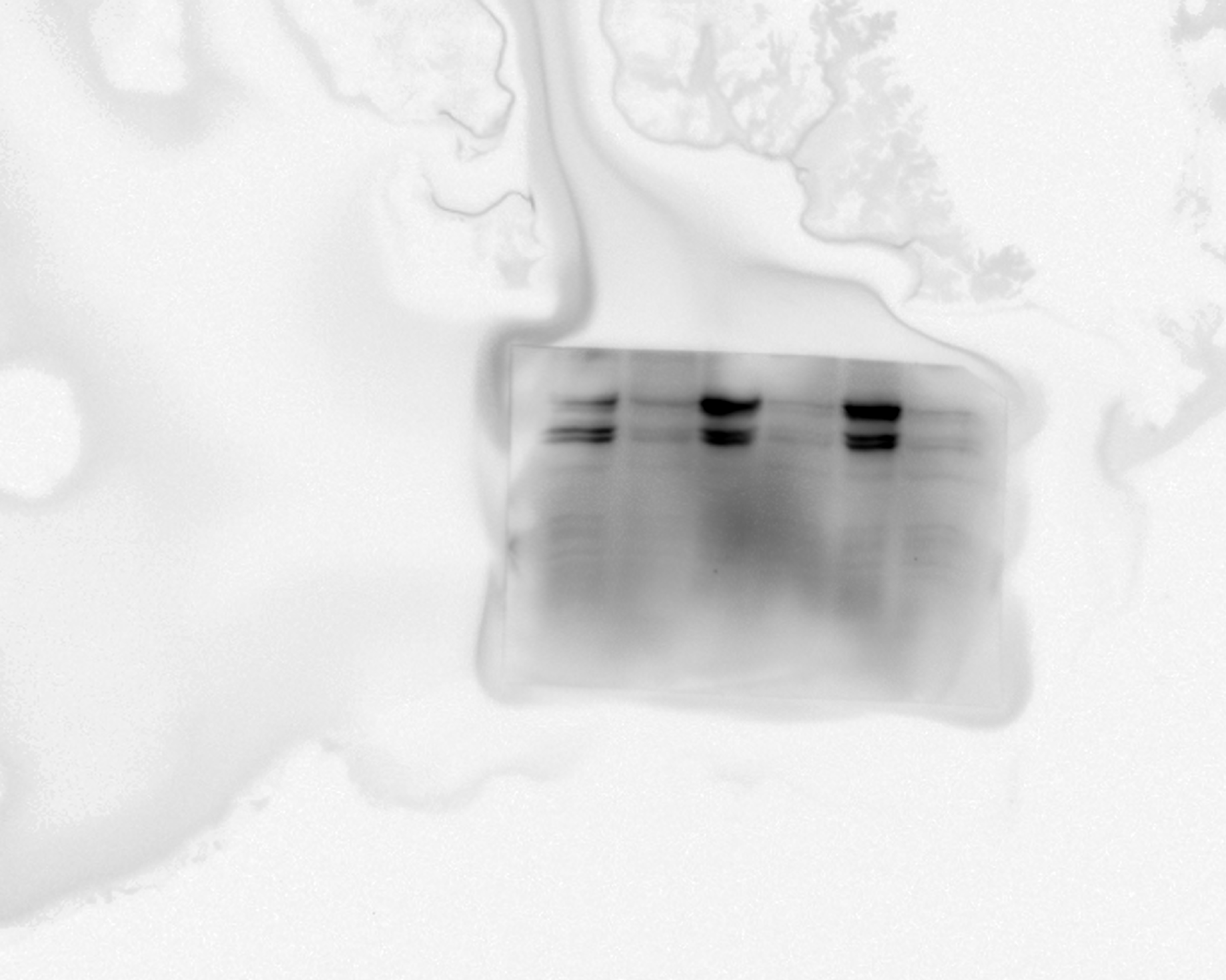

Supplement: Supplementary file 3 — Source data Fig. 1 [file 44319_2026_803_MOESM3_ESM.zip › Figure 1/1C/western SIRT2.tif]

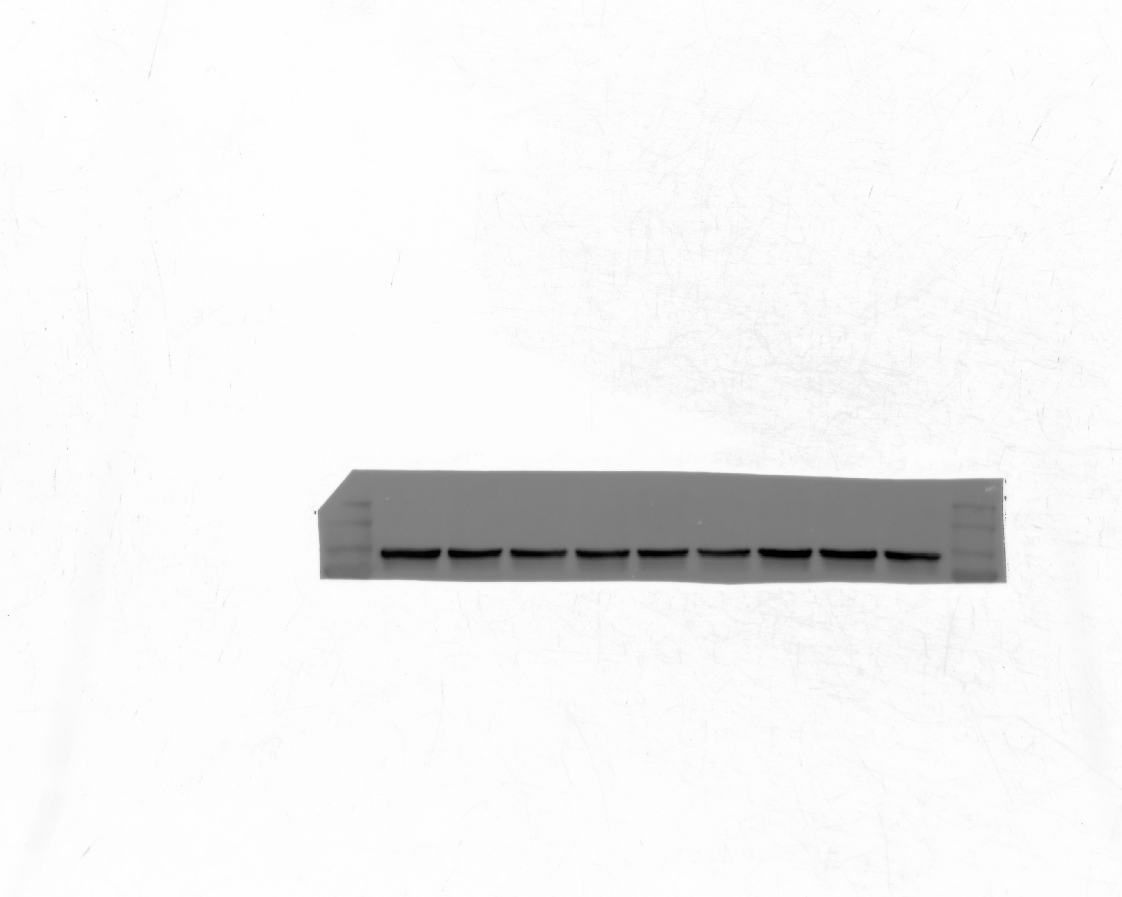

Supplement: Supplementary file 3 — Source data Fig. 1 [file 44319_2026_803_MOESM3_ESM.zip › Figure 1/1D/western -L-K-R HSP90.tif]

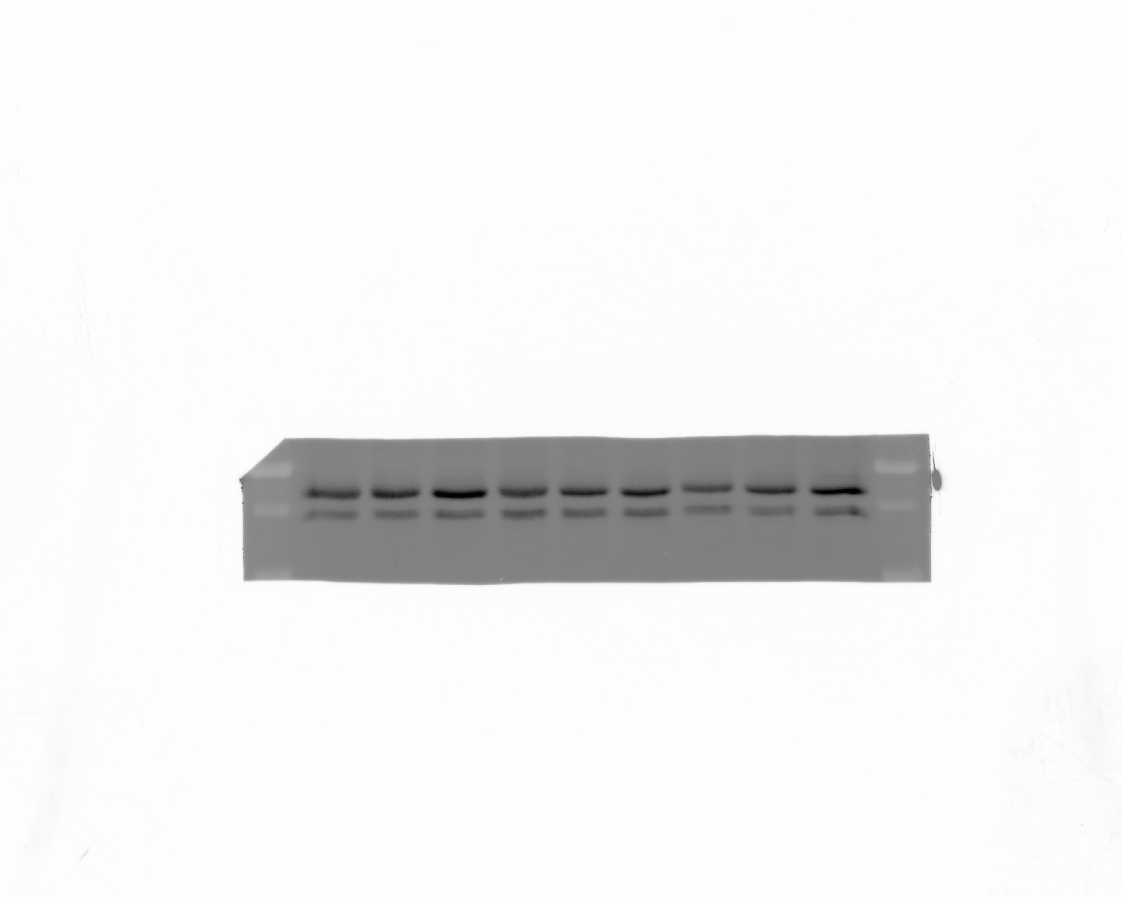

Supplement: Supplementary file 3 — Source data Fig. 1 [file 44319_2026_803_MOESM3_ESM.zip › Figure 1/1D/western -L-K-R SIRT2.tif]

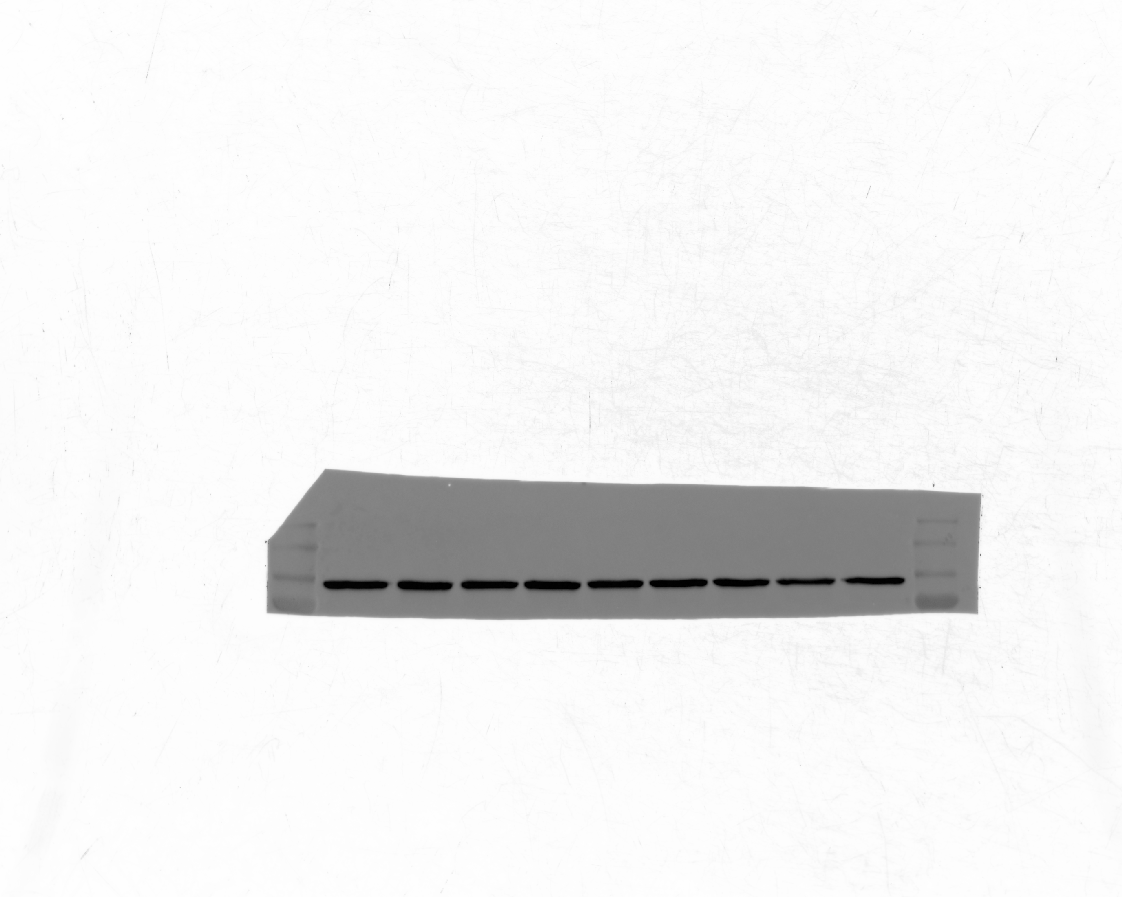

Supplement: Supplementary file 3 — Source data Fig. 1 [file 44319_2026_803_MOESM3_ESM.zip › Figure 1/1D/western -SG HSP90.tif]

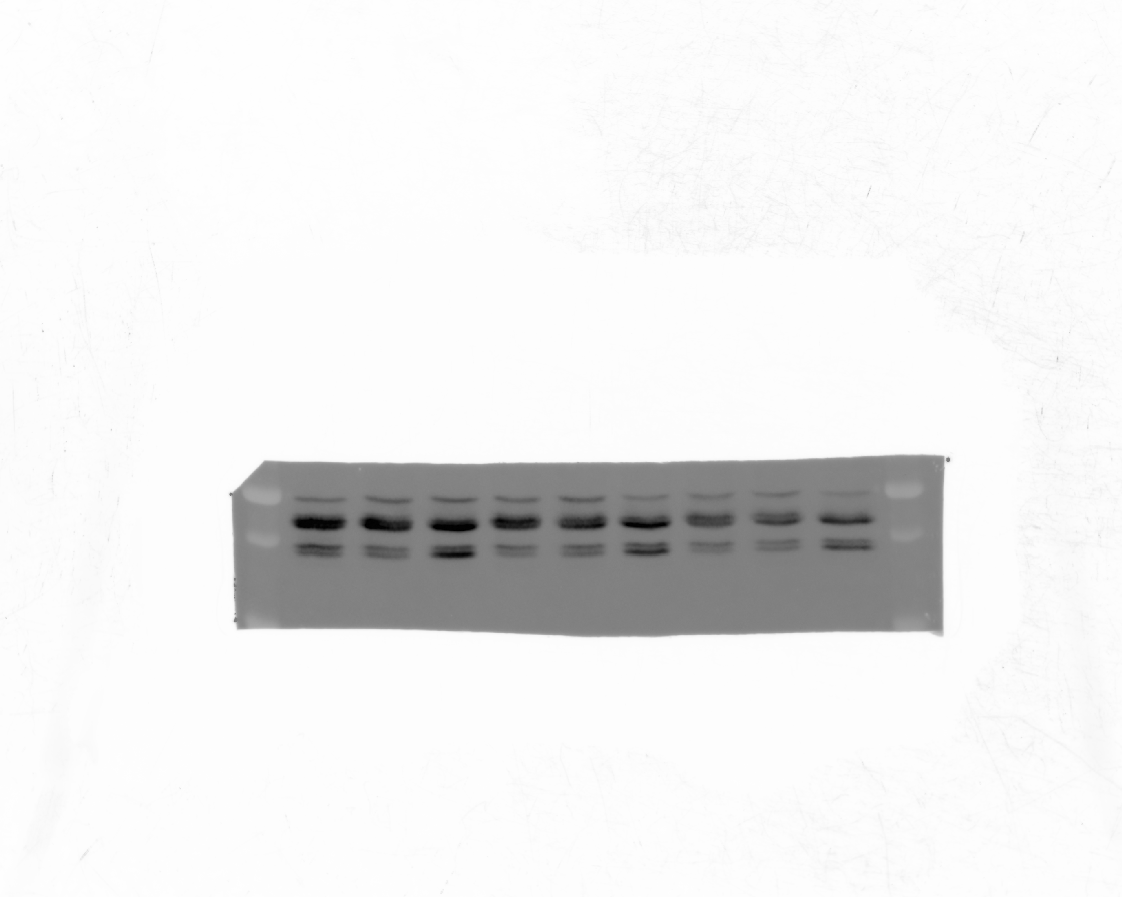

Supplement: Supplementary file 3 — Source data Fig. 1 [file 44319_2026_803_MOESM3_ESM.zip › Figure 1/1D/western -SG SIRT2.tif]

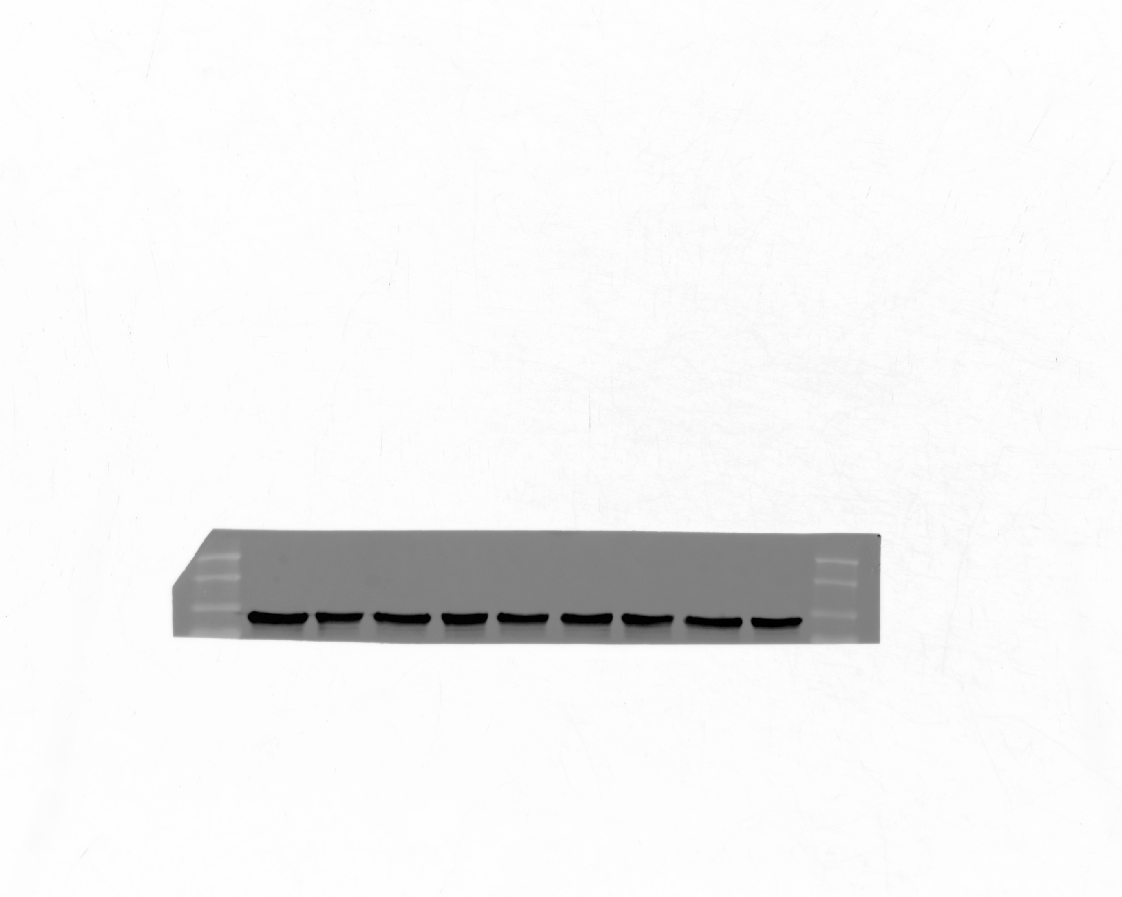

Supplement: Supplementary file 3 — Source data Fig. 1 [file 44319_2026_803_MOESM3_ESM.zip › Figure 1/1F/western HSP90.tif]

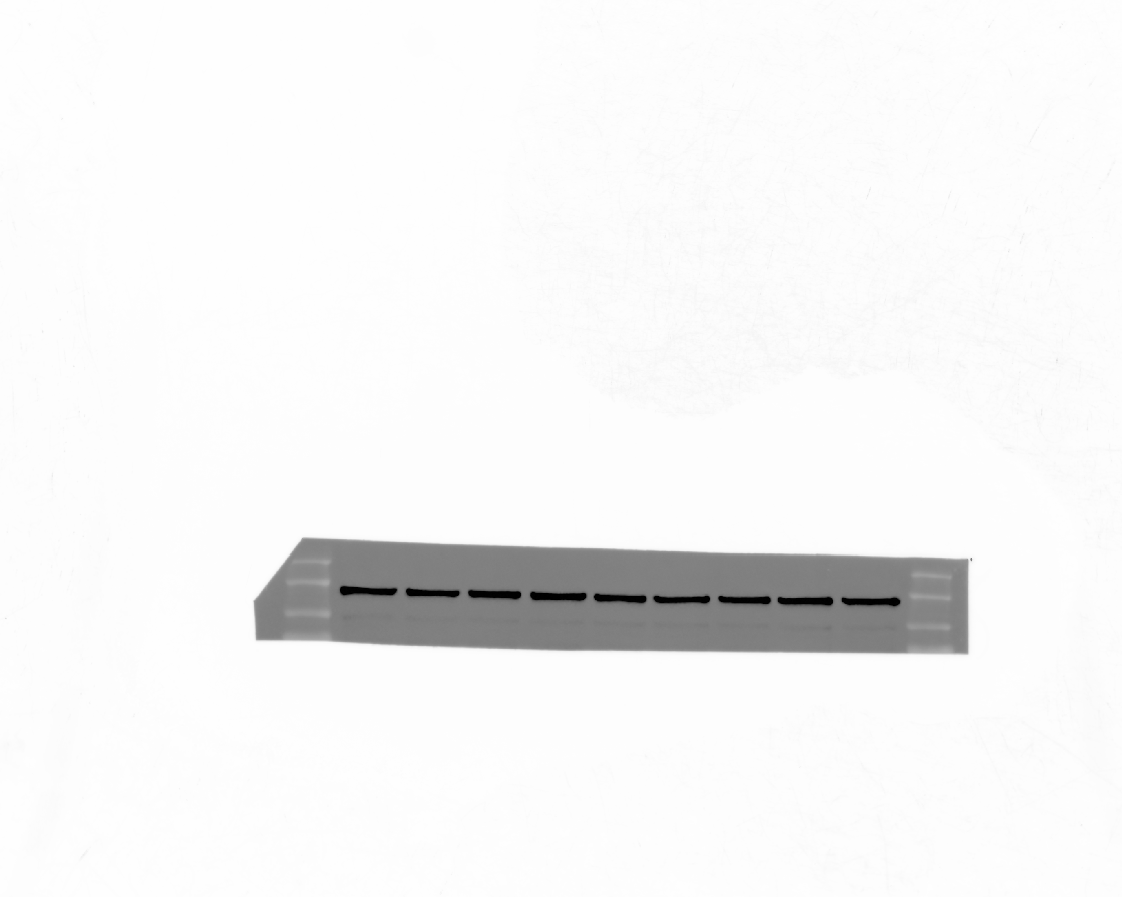

Supplement: Supplementary file 3 — Source data Fig. 1 [file 44319_2026_803_MOESM3_ESM.zip › Figure 1/1F/western SIRT1.tif]

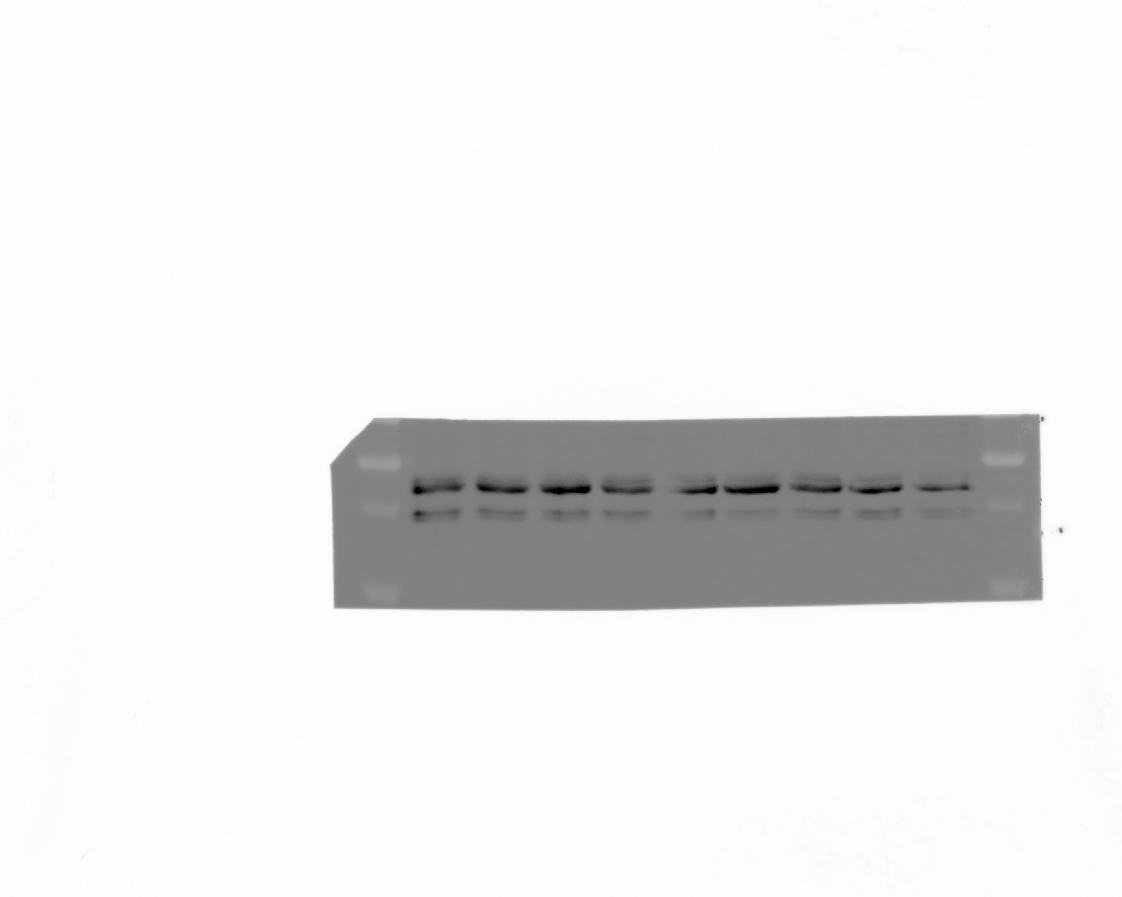

Supplement: Supplementary file 3 — Source data Fig. 1 [file 44319_2026_803_MOESM3_ESM.zip › Figure 1/1F/western SIRT2.tif]

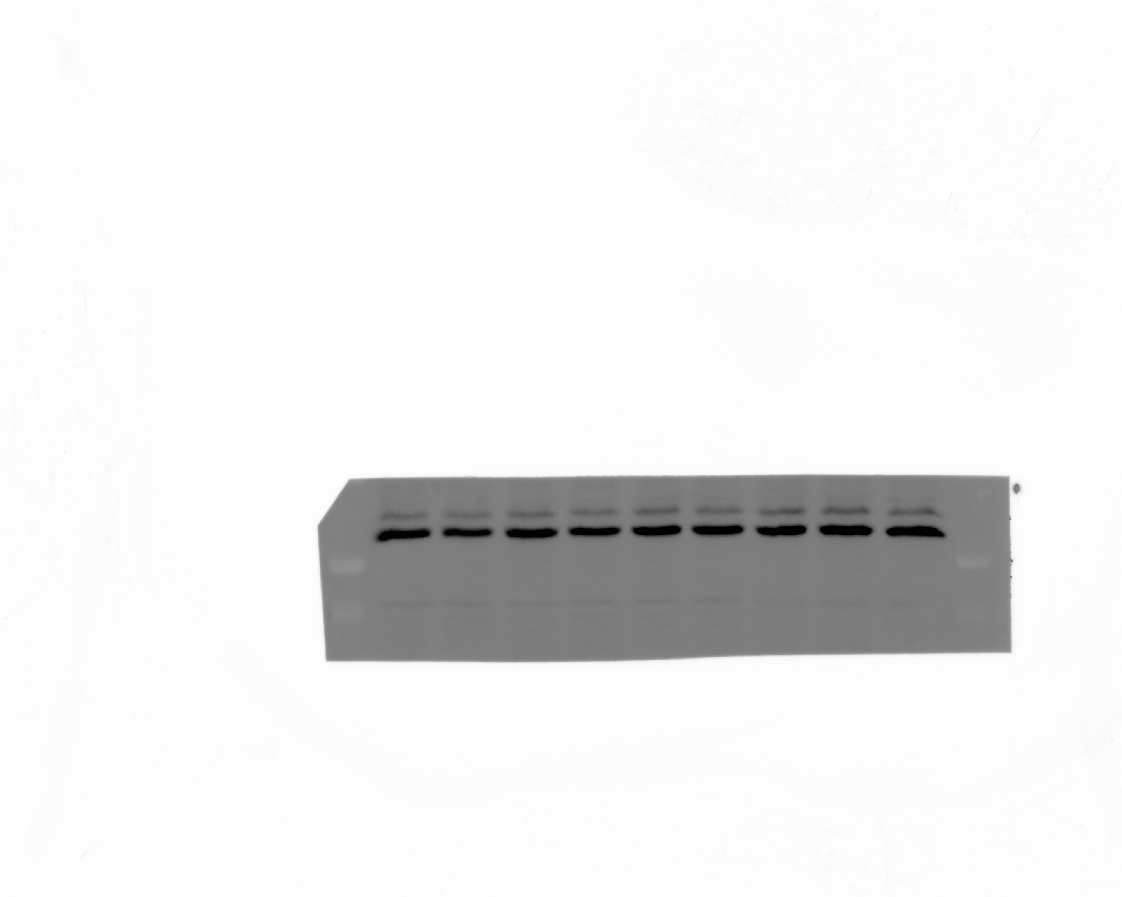

Supplement: Supplementary file 3 — Source data Fig. 1 [file 44319_2026_803_MOESM3_ESM.zip › Figure 1/1F/western SIRT3.tif]

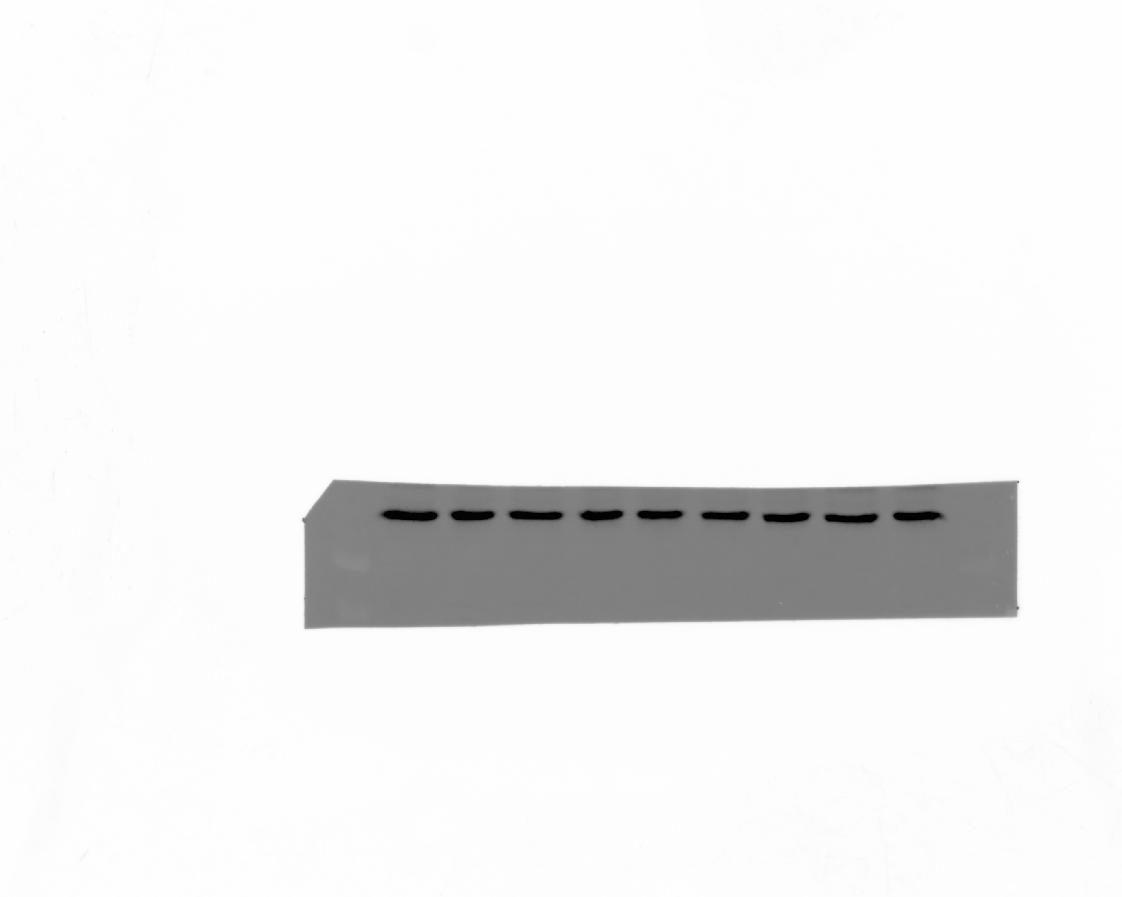

Supplement: Supplementary file 3 — Source data Fig. 1 [file 44319_2026_803_MOESM3_ESM.zip › Figure 1/1F/western SIRT5.tif]

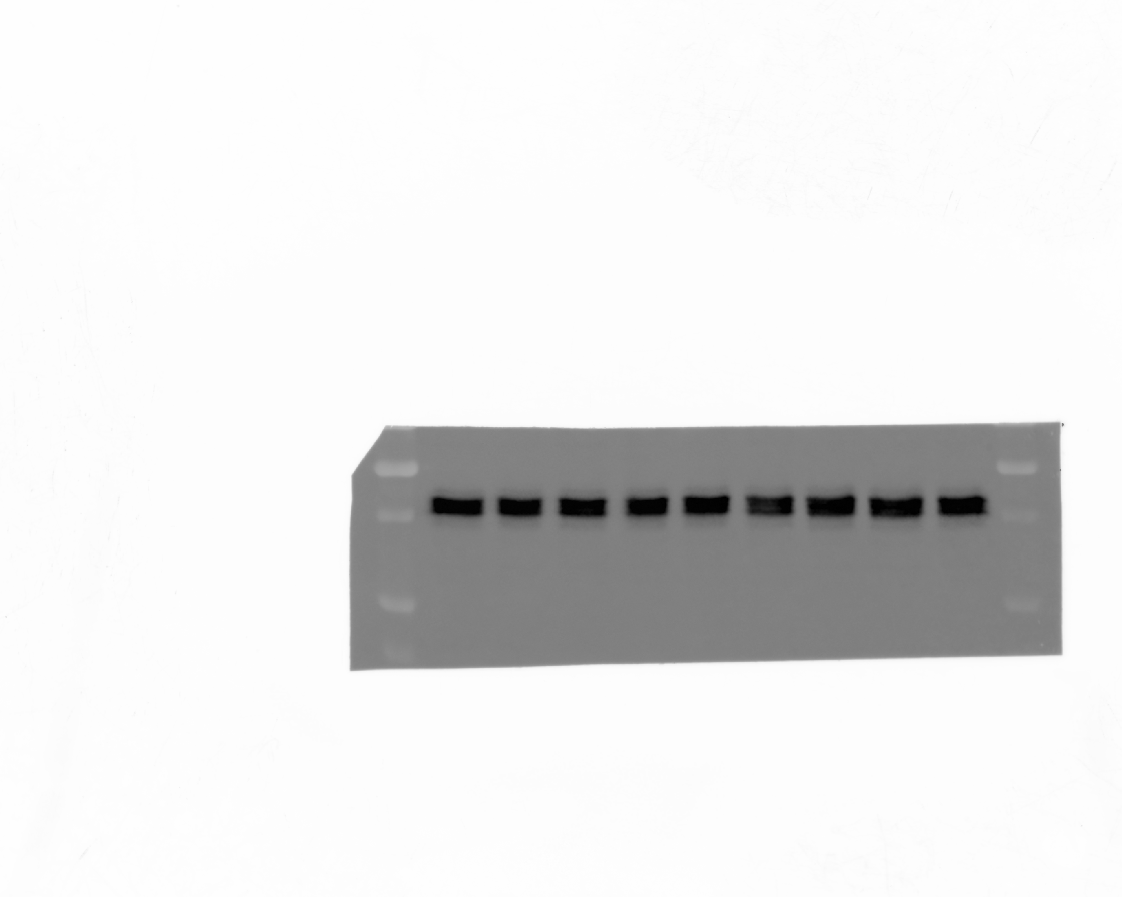

Supplement: Supplementary file 3 — Source data Fig. 1 [file 44319_2026_803_MOESM3_ESM.zip › Figure 1/1F/western SIRT6.tif]

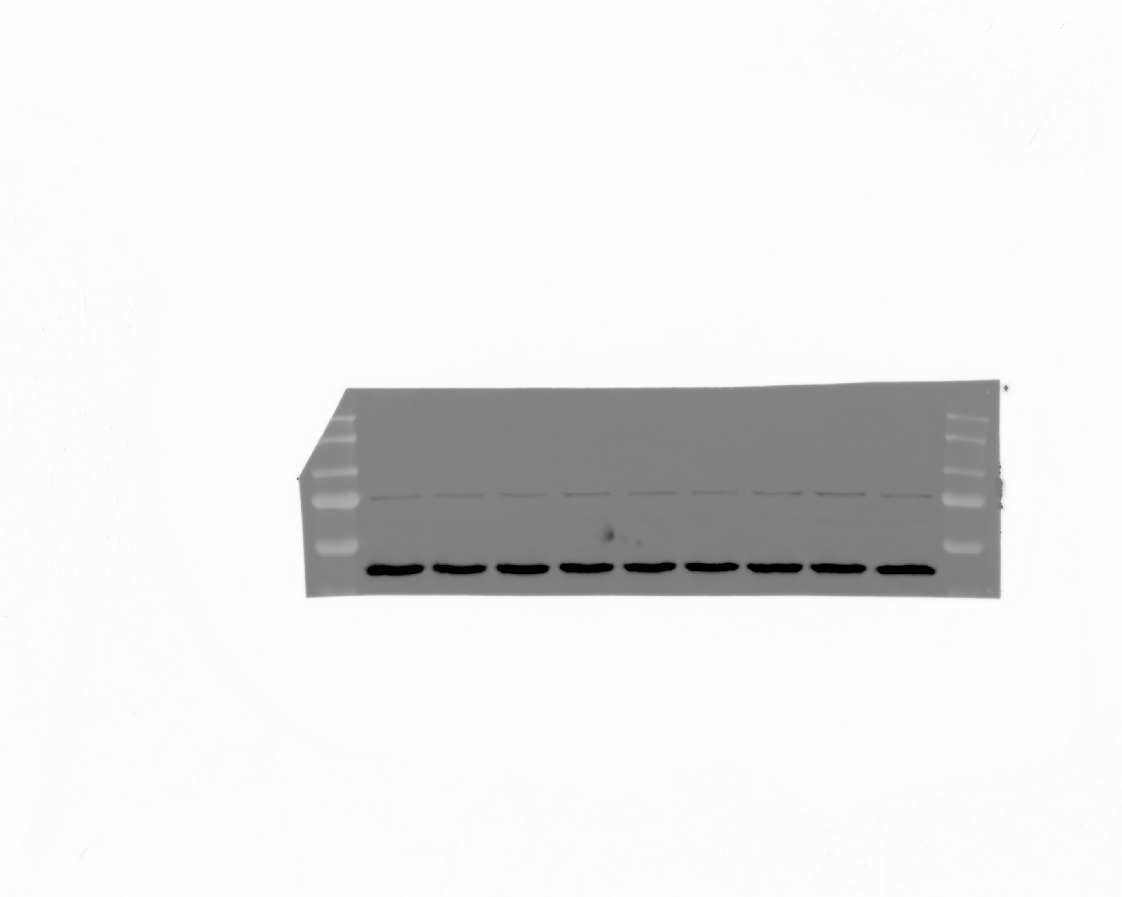

Supplement: Supplementary file 3 — Source data Fig. 1 [file 44319_2026_803_MOESM3_ESM.zip › Figure 1/1F/western SIRT7.tif]

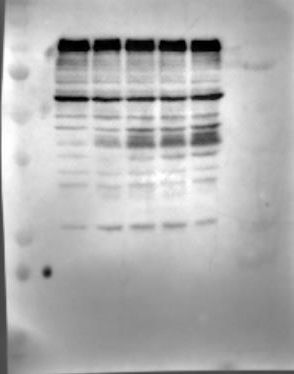

Supplement: Supplementary file 4 — Source data Fig. 2 [file 44319_2026_803_MOESM4_ESM.zip › Figure 2/2A/western ATF4.tif]

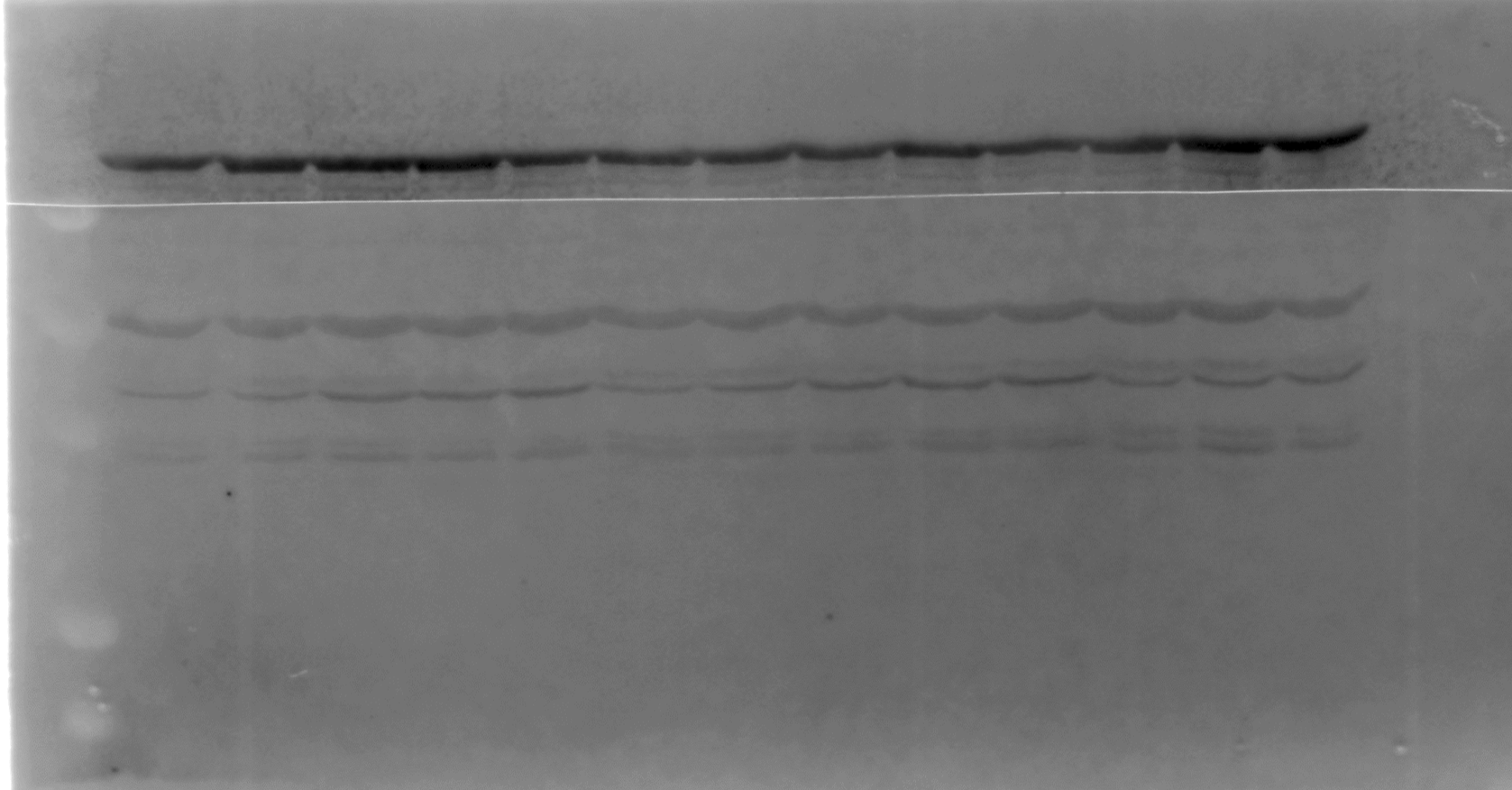

Supplement: Supplementary file 4 — Source data Fig. 2 [file 44319_2026_803_MOESM4_ESM.zip › Figure 2/2A/western HSP90.tif]

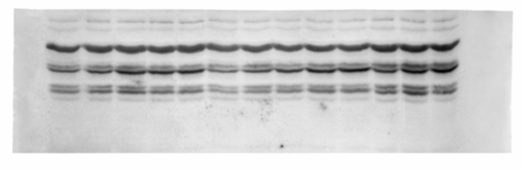

Supplement: Supplementary file 4 — Source data Fig. 2 [file 44319_2026_803_MOESM4_ESM.zip › Figure 2/2A/western SIRT2.tif]

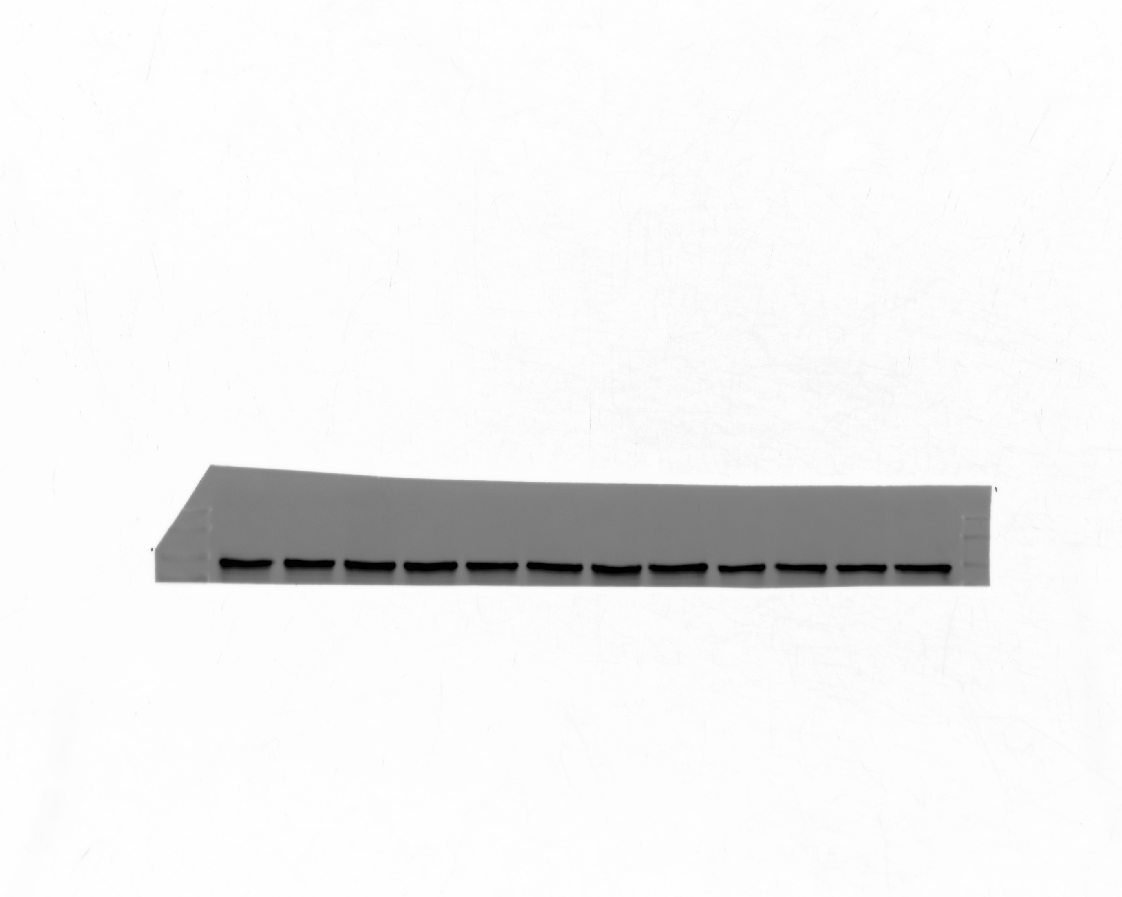

Supplement: Supplementary file 4 — Source data Fig. 2 [file 44319_2026_803_MOESM4_ESM.zip › Figure 2/2B/western HSP90.tif]

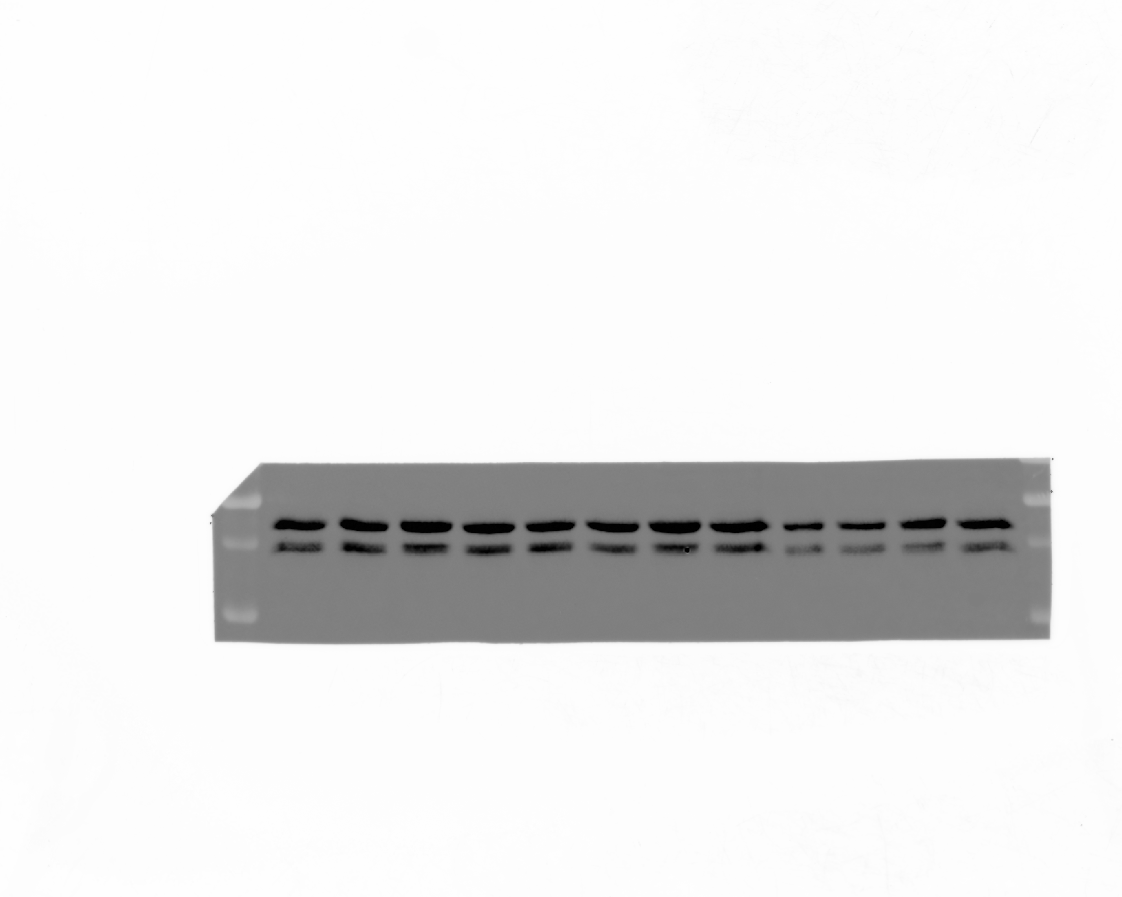

Supplement: Supplementary file 4 — Source data Fig. 2 [file 44319_2026_803_MOESM4_ESM.zip › Figure 2/2B/western SIRT2.tif]

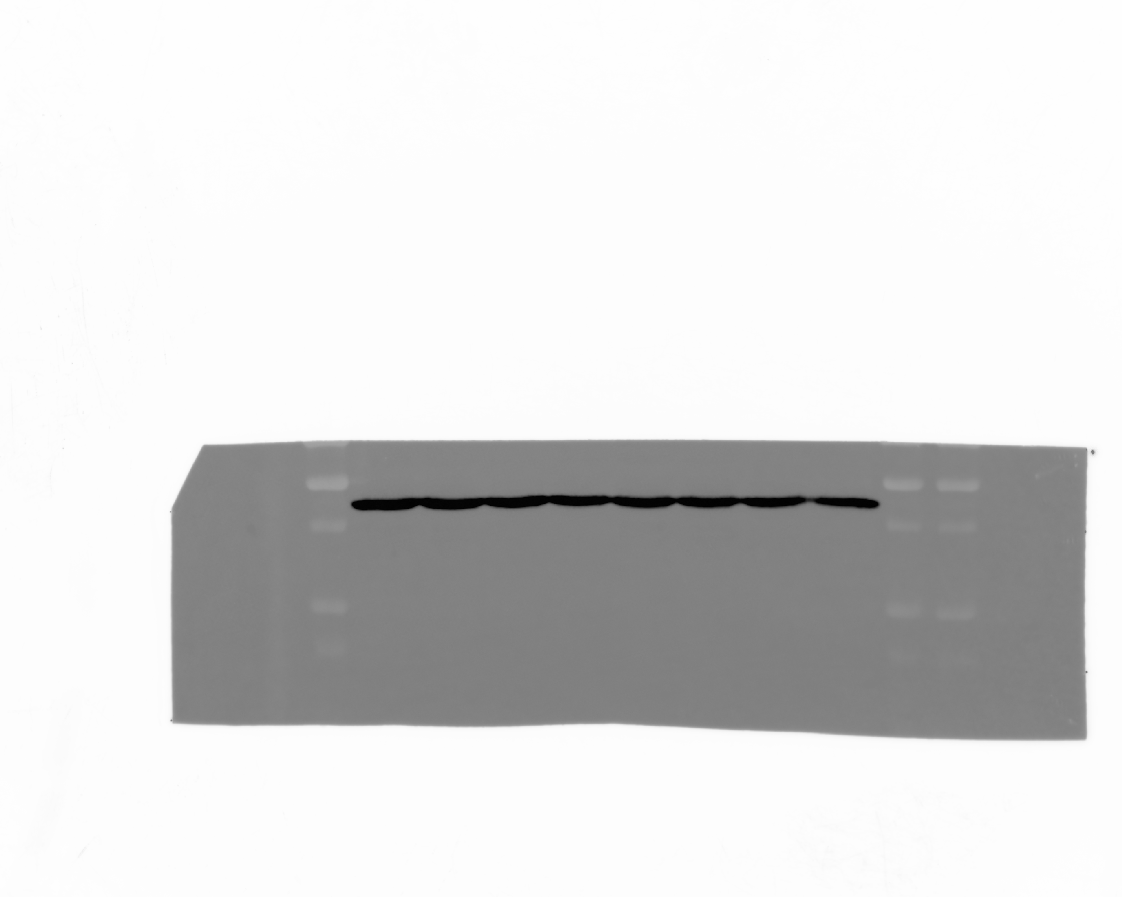

Supplement: Supplementary file 4 — Source data Fig. 2 [file 44319_2026_803_MOESM4_ESM.zip › Figure 2/2C/western beta-Actin.tif]

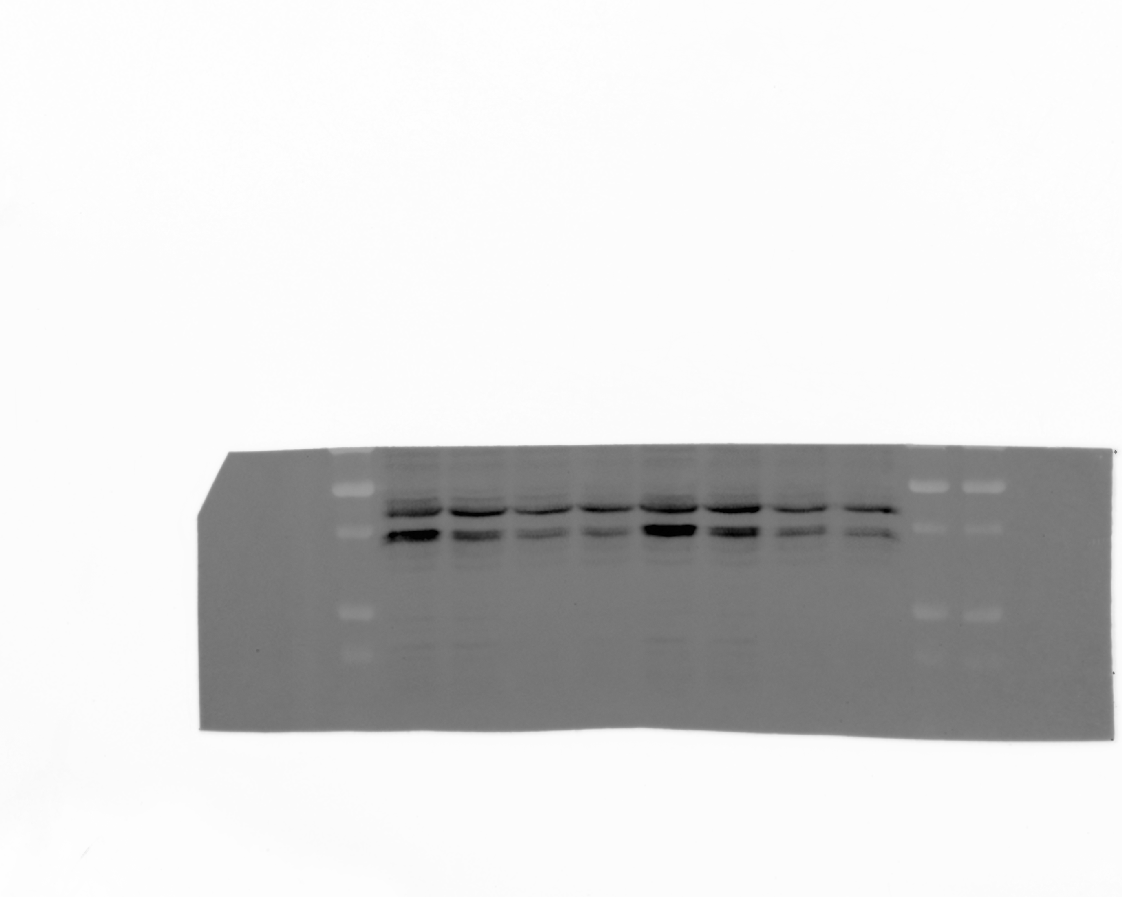

Supplement: Supplementary file 4 — Source data Fig. 2 [file 44319_2026_803_MOESM4_ESM.zip › Figure 2/2C/western SIRT2.tif]

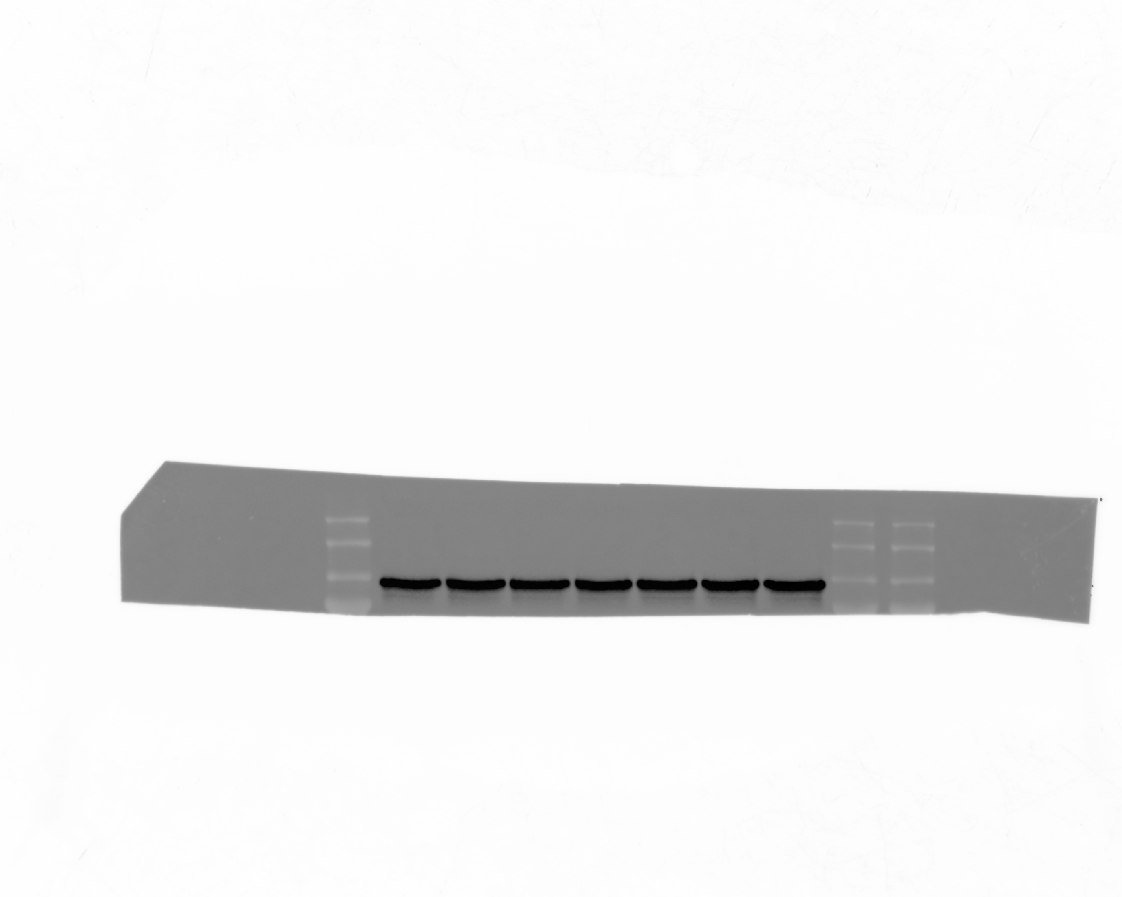

Supplement: Supplementary file 6 — Source data Fig. 4 [file 44319_2026_803_MOESM6_ESM.zip › Figure 4/4A/western HSP90.tif]

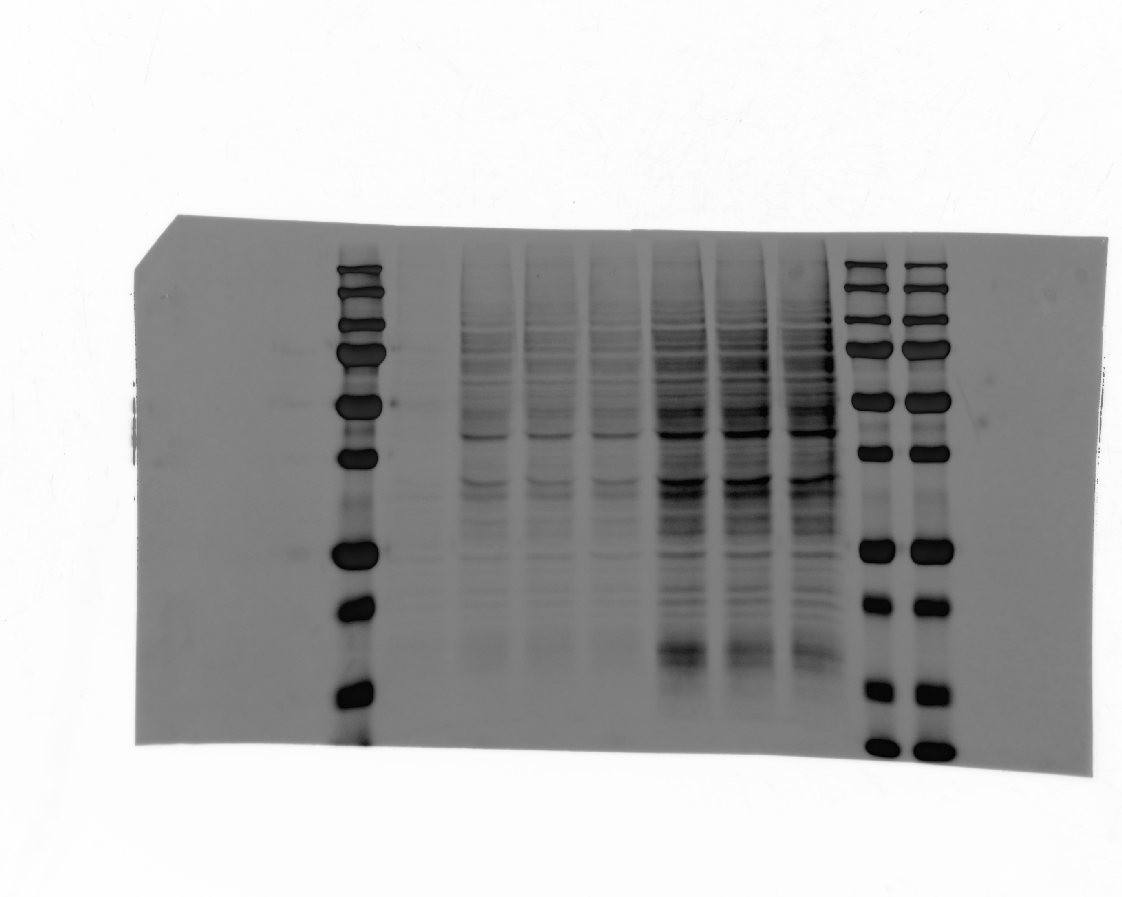

Supplement: Supplementary file 6 — Source data Fig. 4 [file 44319_2026_803_MOESM6_ESM.zip › Figure 4/4A/western puromycin.tif]

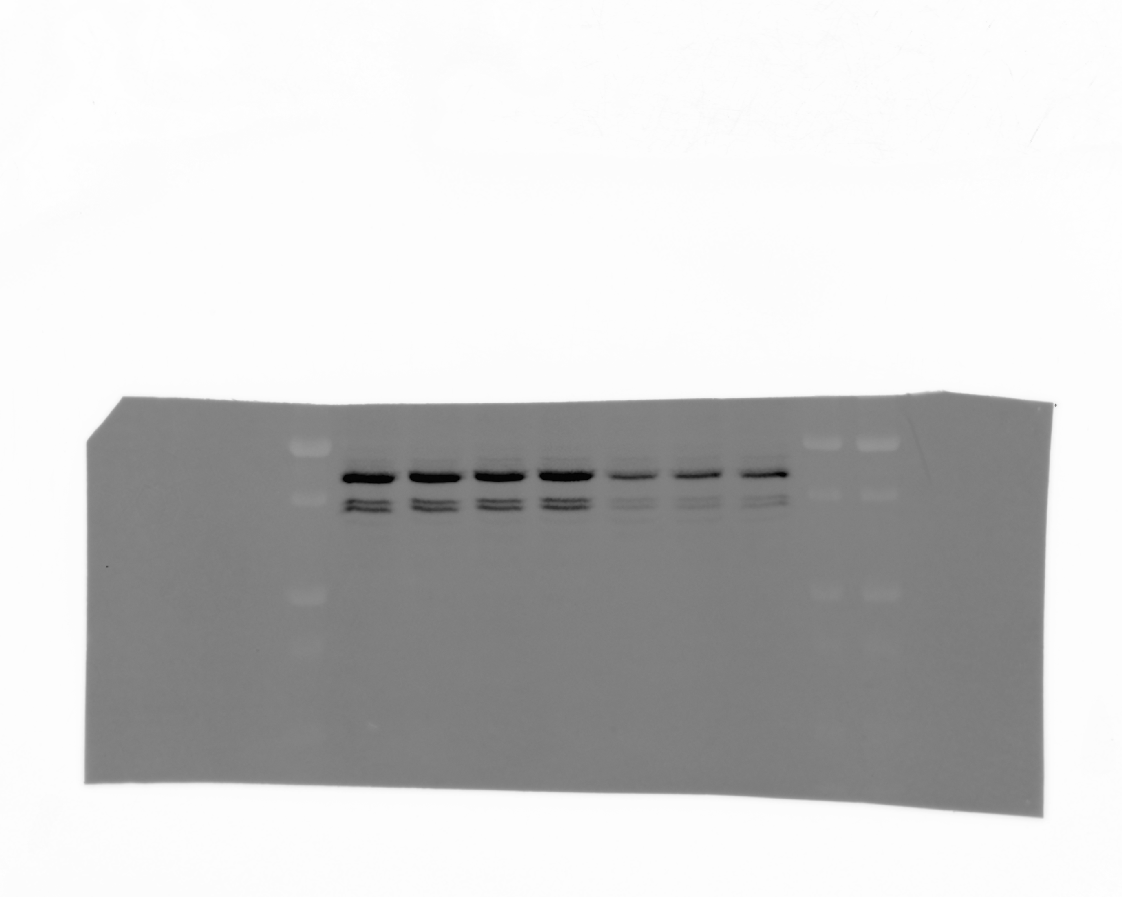

Supplement: Supplementary file 6 — Source data Fig. 4 [file 44319_2026_803_MOESM6_ESM.zip › Figure 4/4A/western SIRT2.tif]

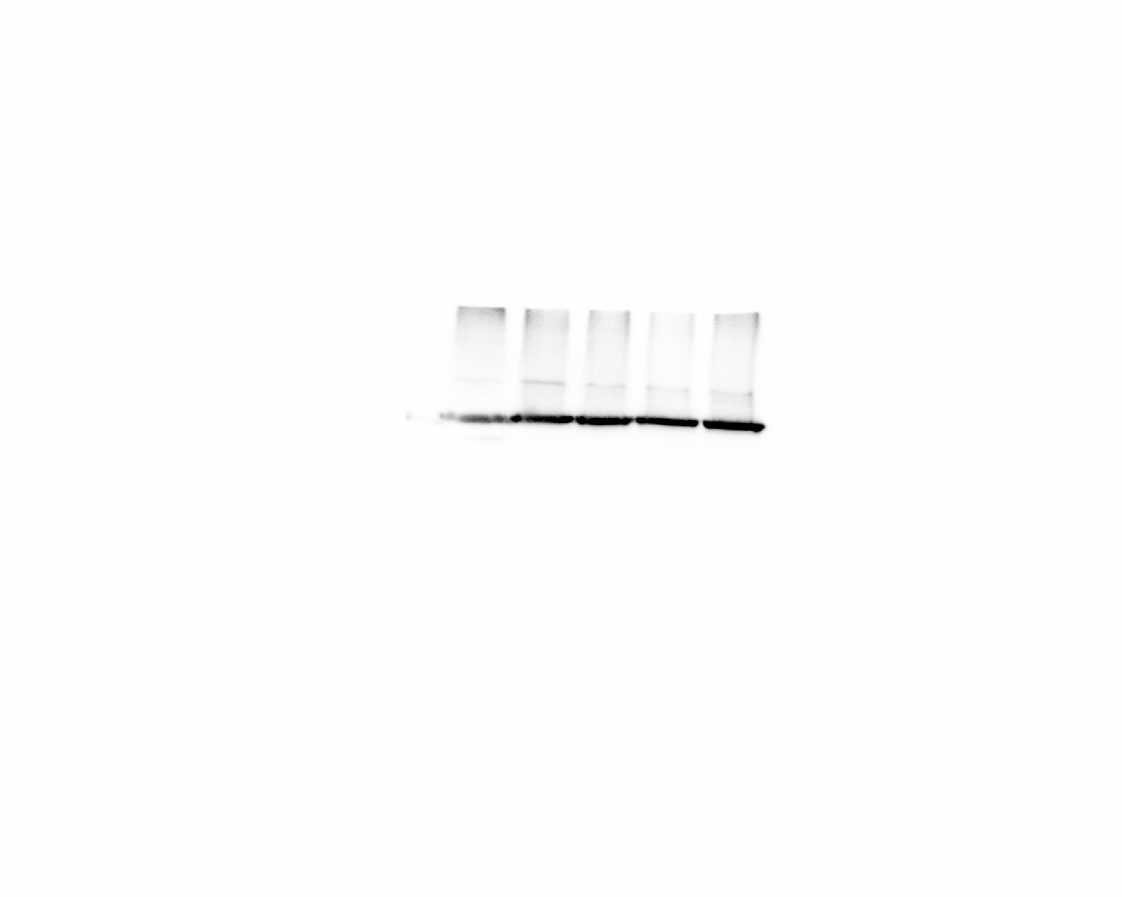

Supplement: Supplementary file 6 — Source data Fig. 4 [file 44319_2026_803_MOESM6_ESM.zip › Figure 4/4B/western HSP90.tif]

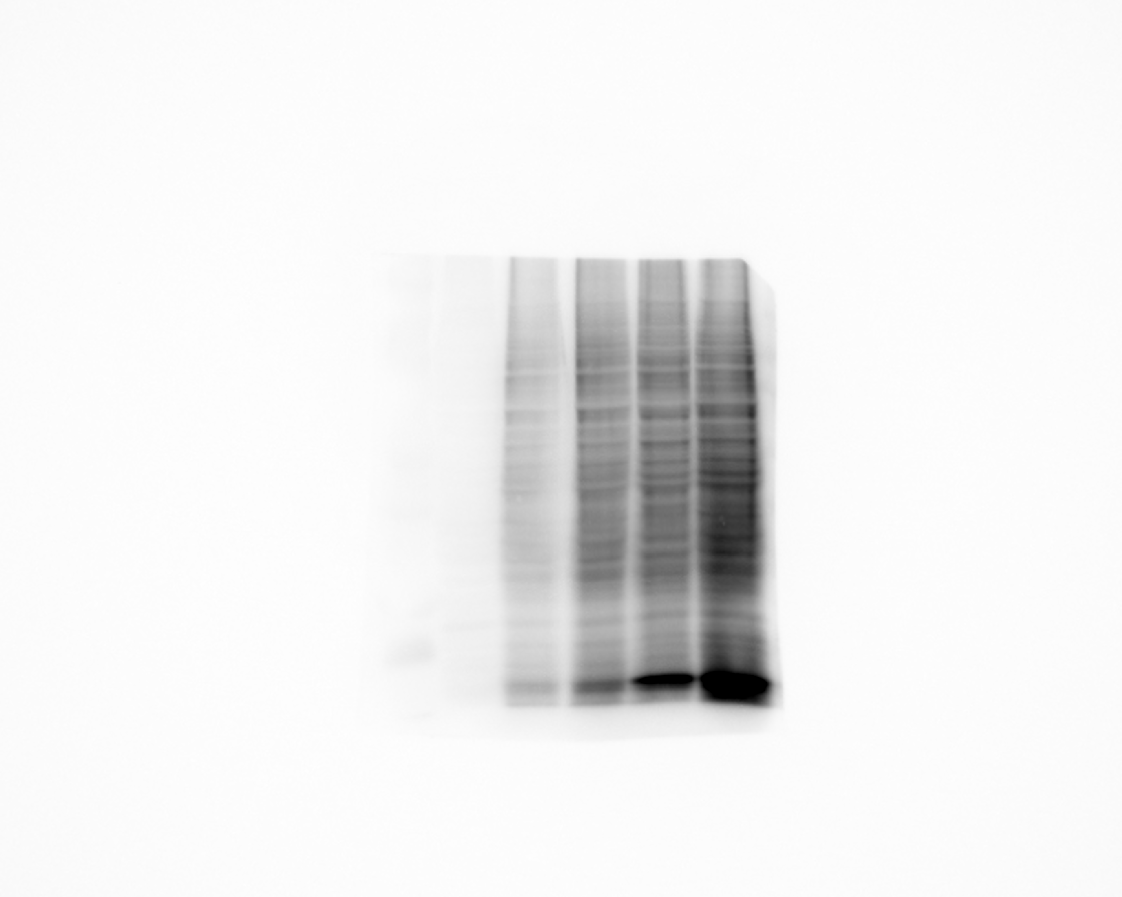

Supplement: Supplementary file 6 — Source data Fig. 4 [file 44319_2026_803_MOESM6_ESM.zip › Figure 4/4B/western puromycin.tif]

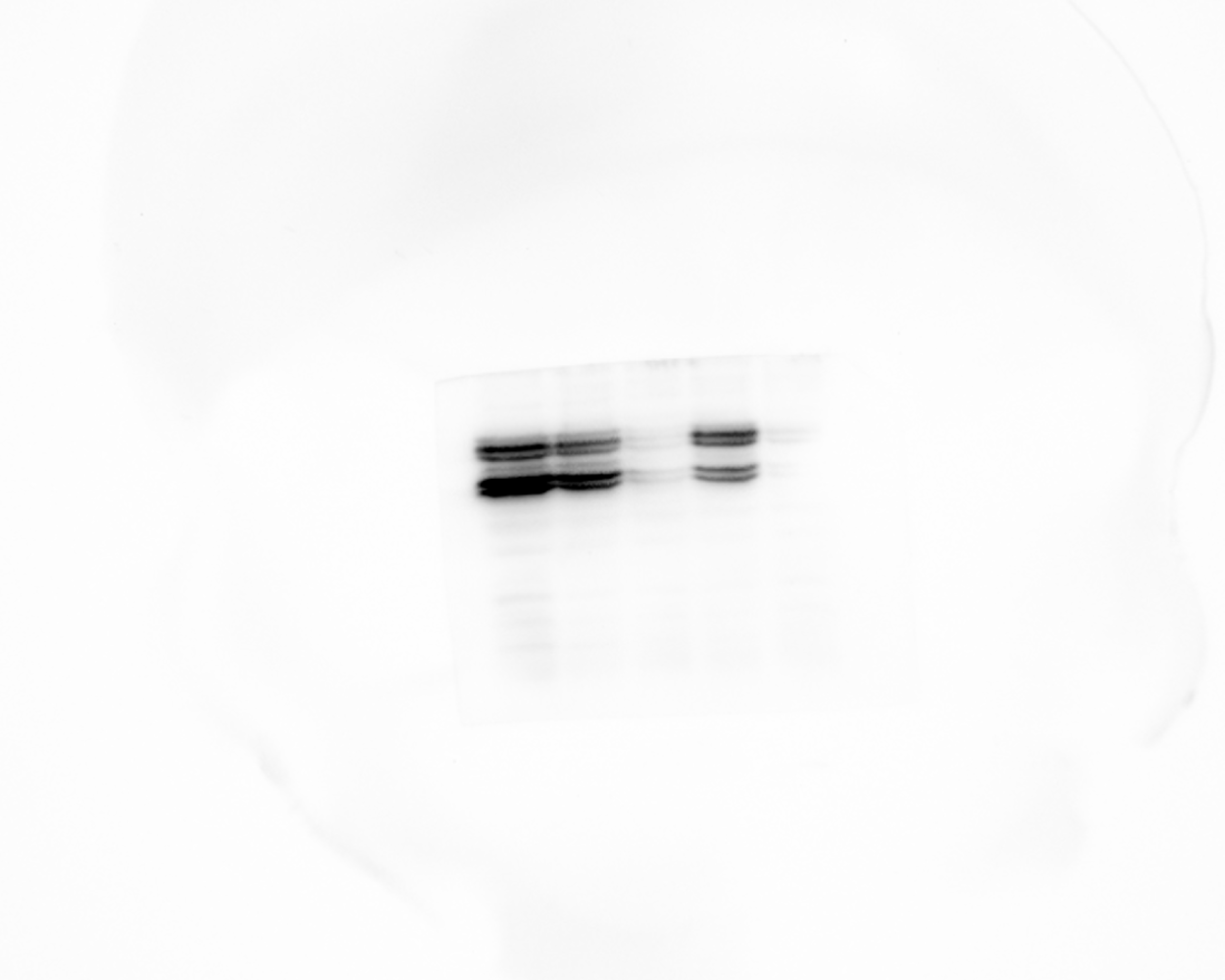

Supplement: Supplementary file 6 — Source data Fig. 4 [file 44319_2026_803_MOESM6_ESM.zip › Figure 4/4B/western SIRT2.tif]

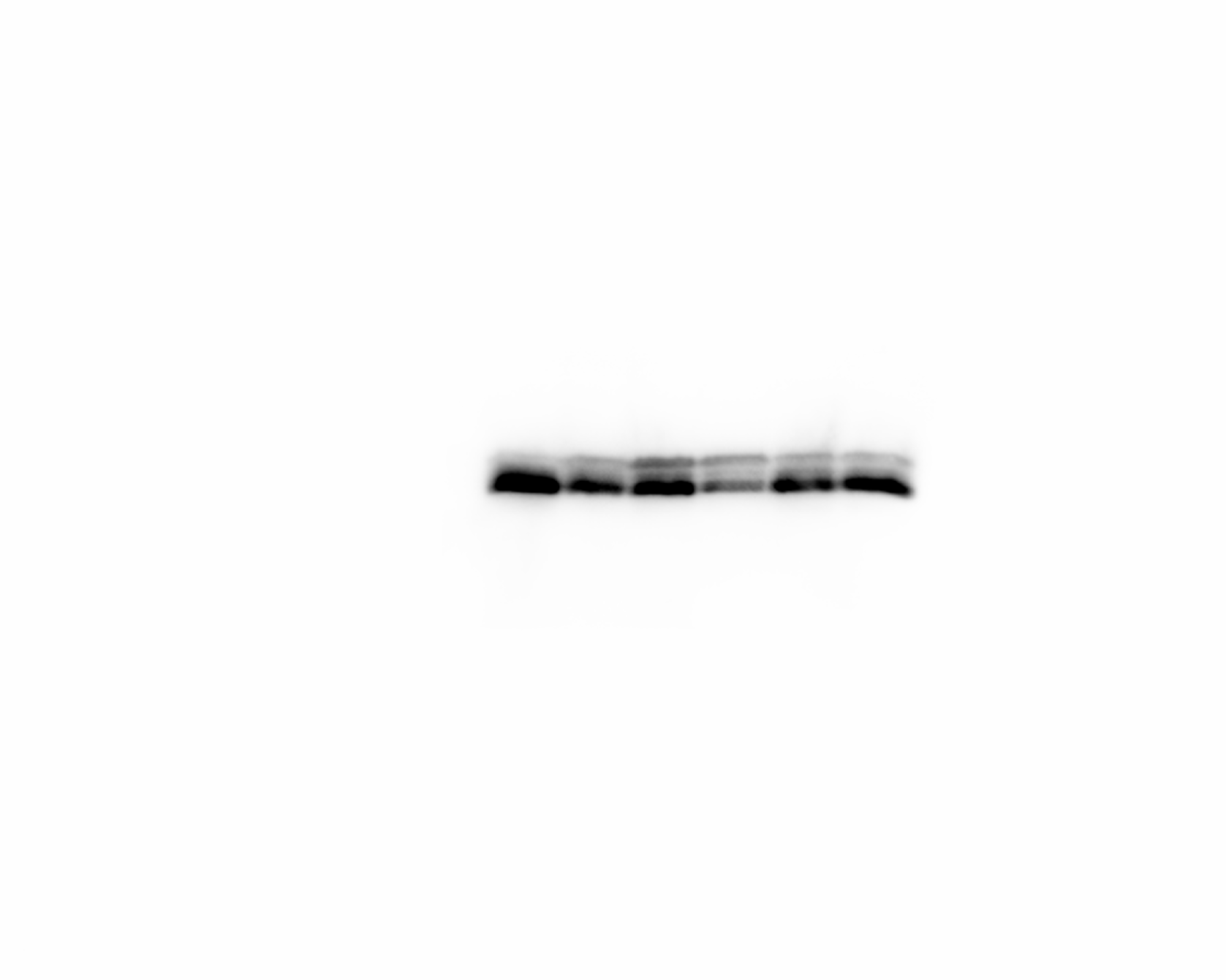

Supplement: Supplementary file 6 — Source data Fig. 4 [file 44319_2026_803_MOESM6_ESM.zip › Figure 4/4C/western 293T 4EBP1.tif]

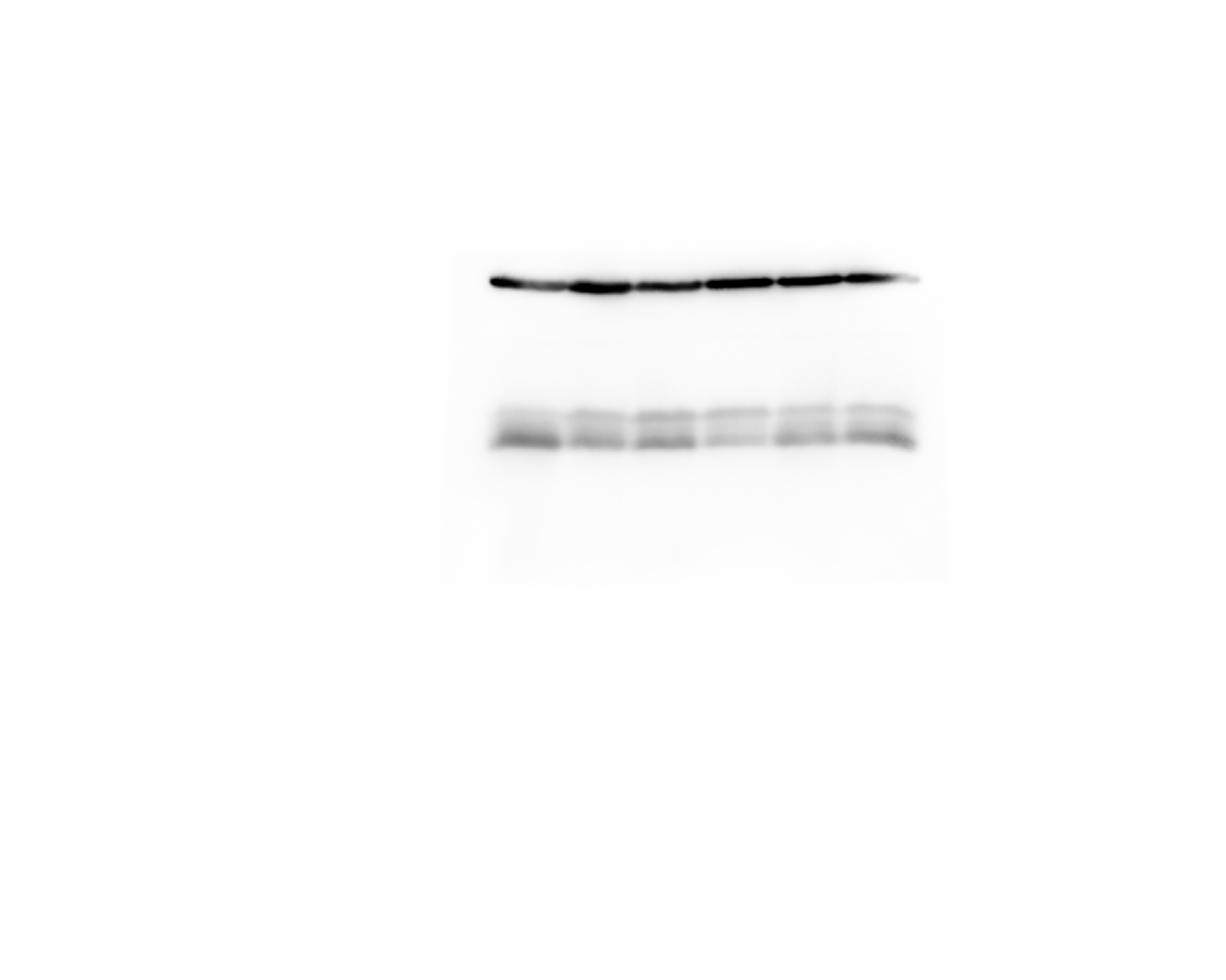

Supplement: Supplementary file 6 — Source data Fig. 4 [file 44319_2026_803_MOESM6_ESM.zip › Figure 4/4C/western 293T Actin (for 4EBP1).tif]

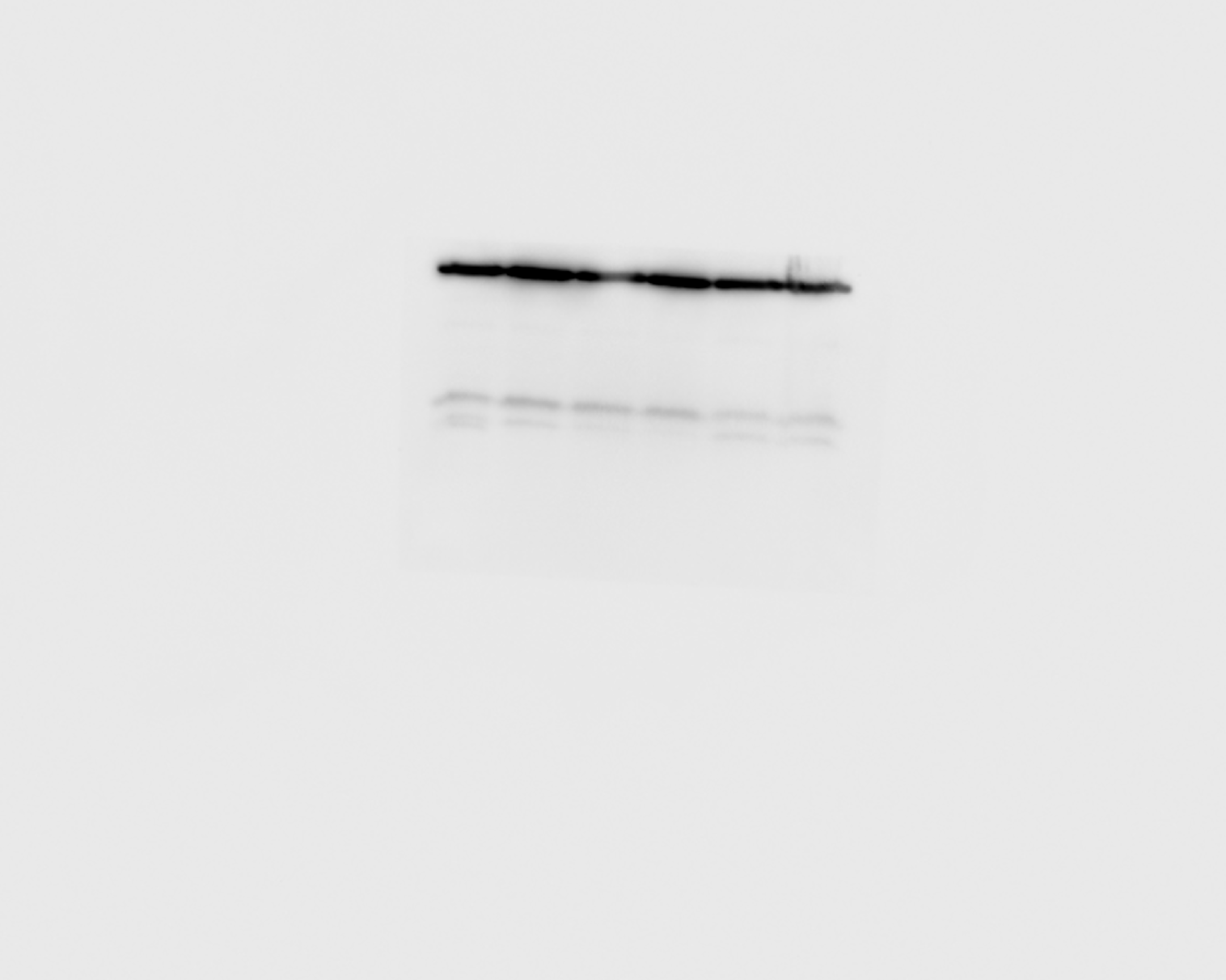

Supplement: Supplementary file 6 — Source data Fig. 4 [file 44319_2026_803_MOESM6_ESM.zip › Figure 4/4C/western 293T Actin (for p-4EBP1).tif]

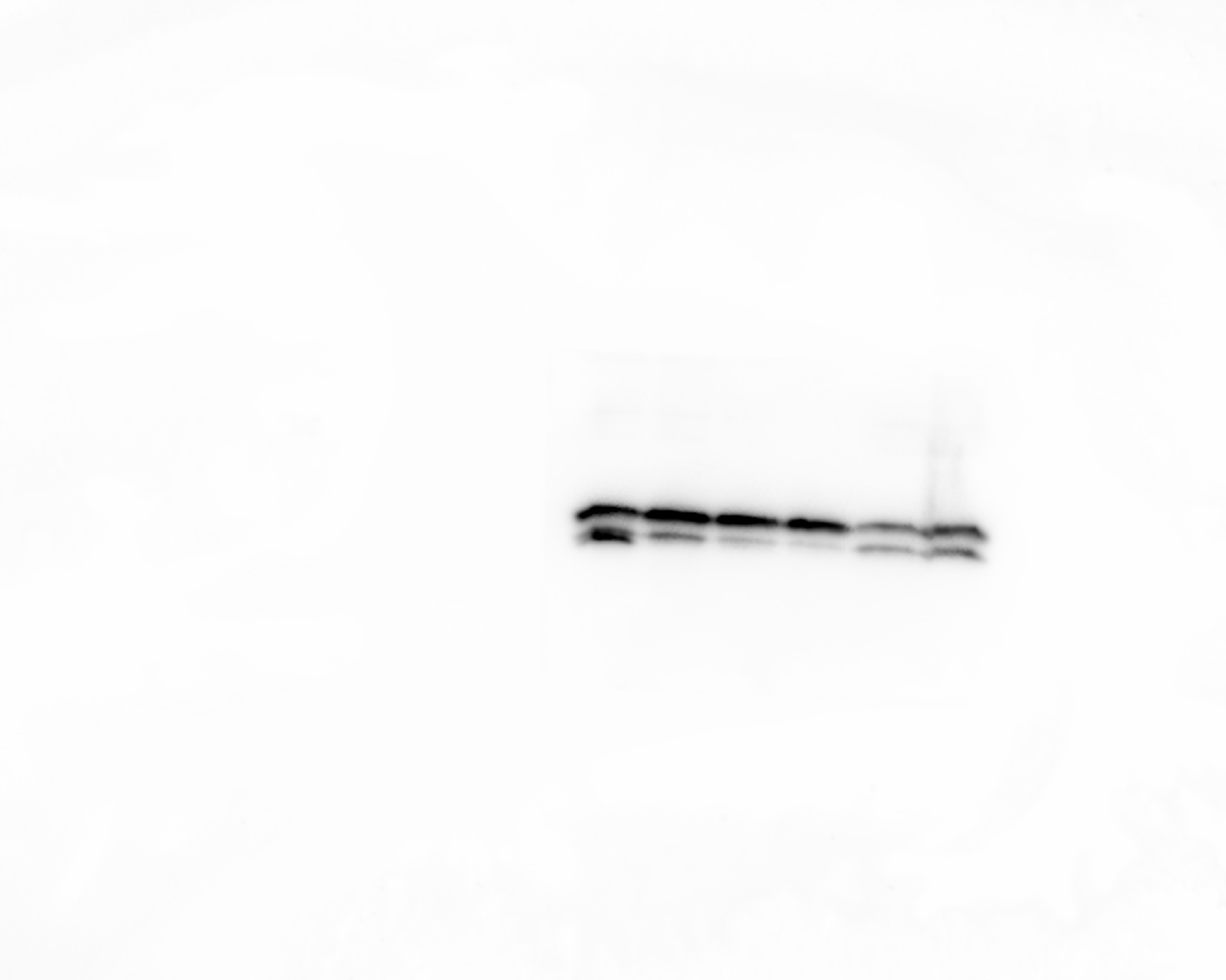

Supplement: Supplementary file 6 — Source data Fig. 4 [file 44319_2026_803_MOESM6_ESM.zip › Figure 4/4C/western 293T p-4EBP1 (Ser65).tif]

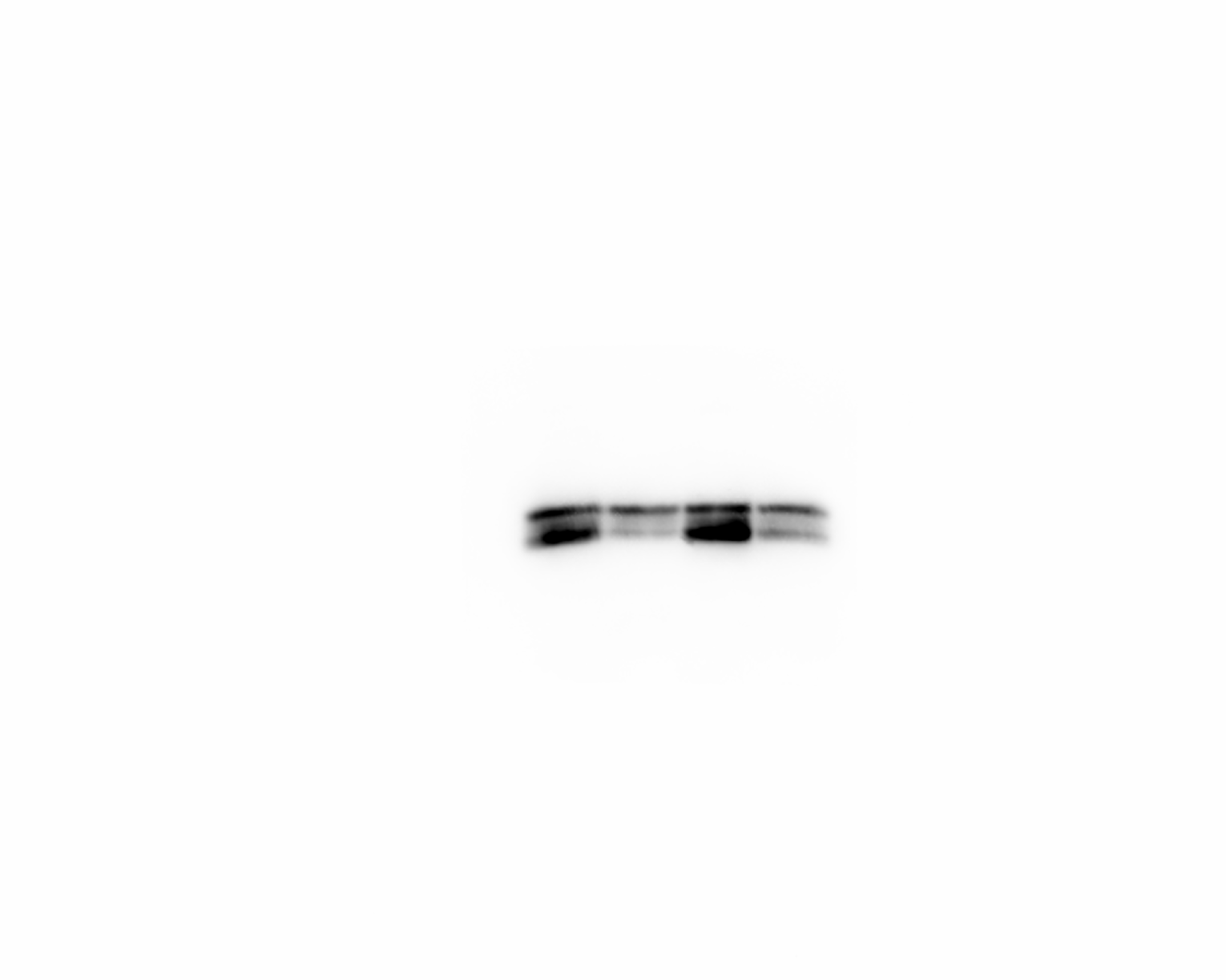

Supplement: Supplementary file 6 — Source data Fig. 4 [file 44319_2026_803_MOESM6_ESM.zip › Figure 4/4C/western A549 4EBP1.tif]

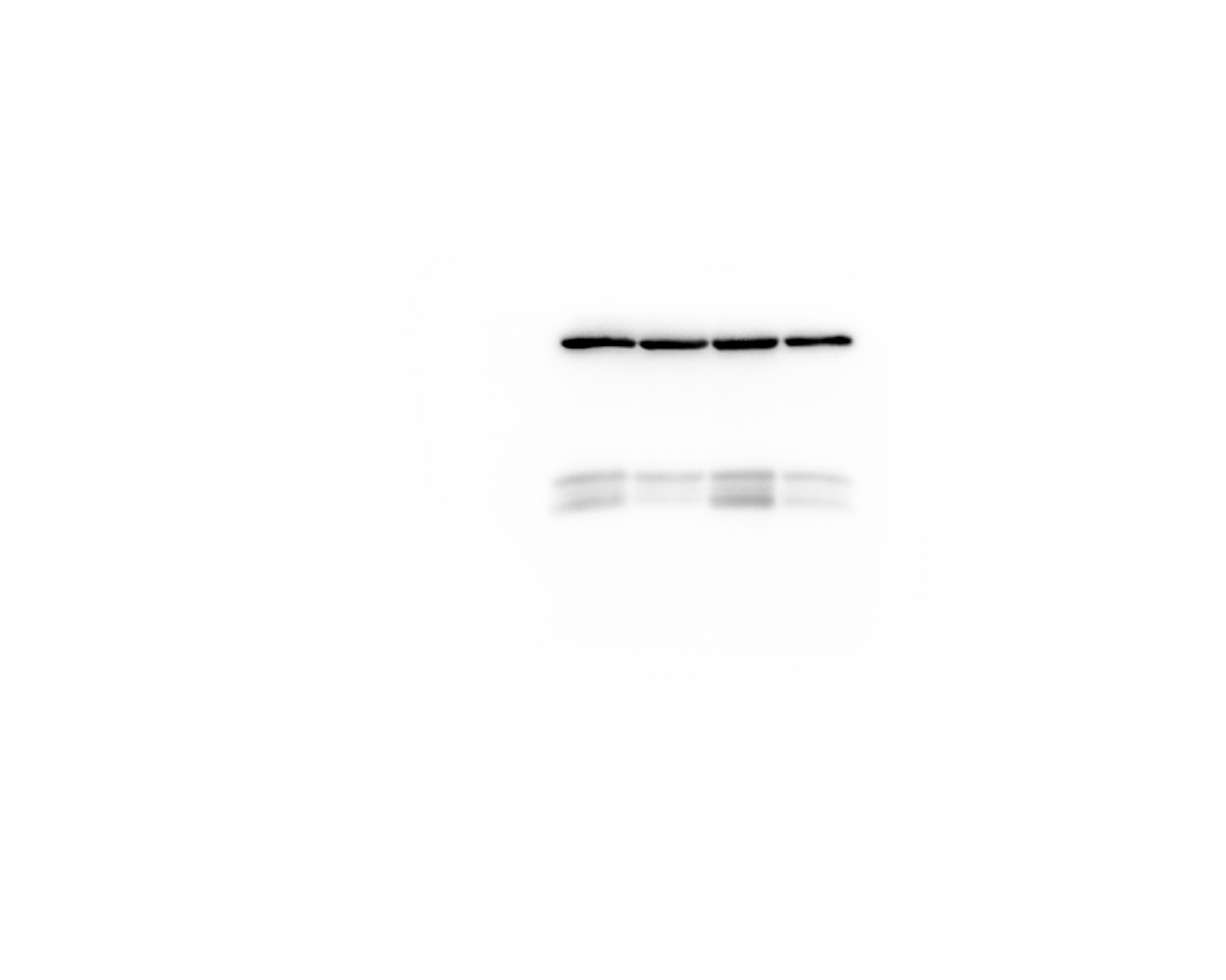

Supplement: Supplementary file 6 — Source data Fig. 4 [file 44319_2026_803_MOESM6_ESM.zip › Figure 4/4C/western A549 Actin (for 4EBP1).tif]

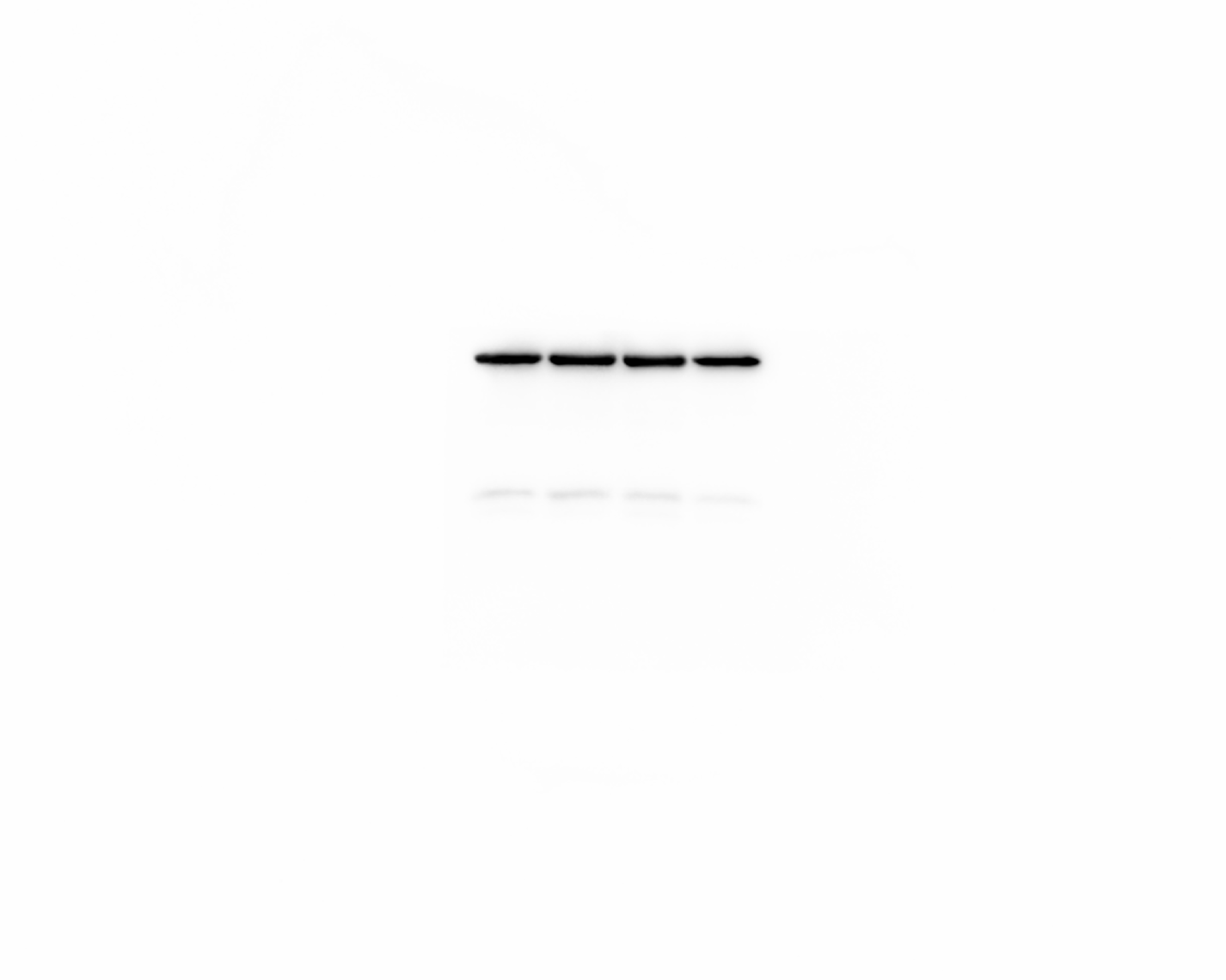

Supplement: Supplementary file 6 — Source data Fig. 4 [file 44319_2026_803_MOESM6_ESM.zip › Figure 4/4C/western A549 Actin (for p-4EBP1).tif]

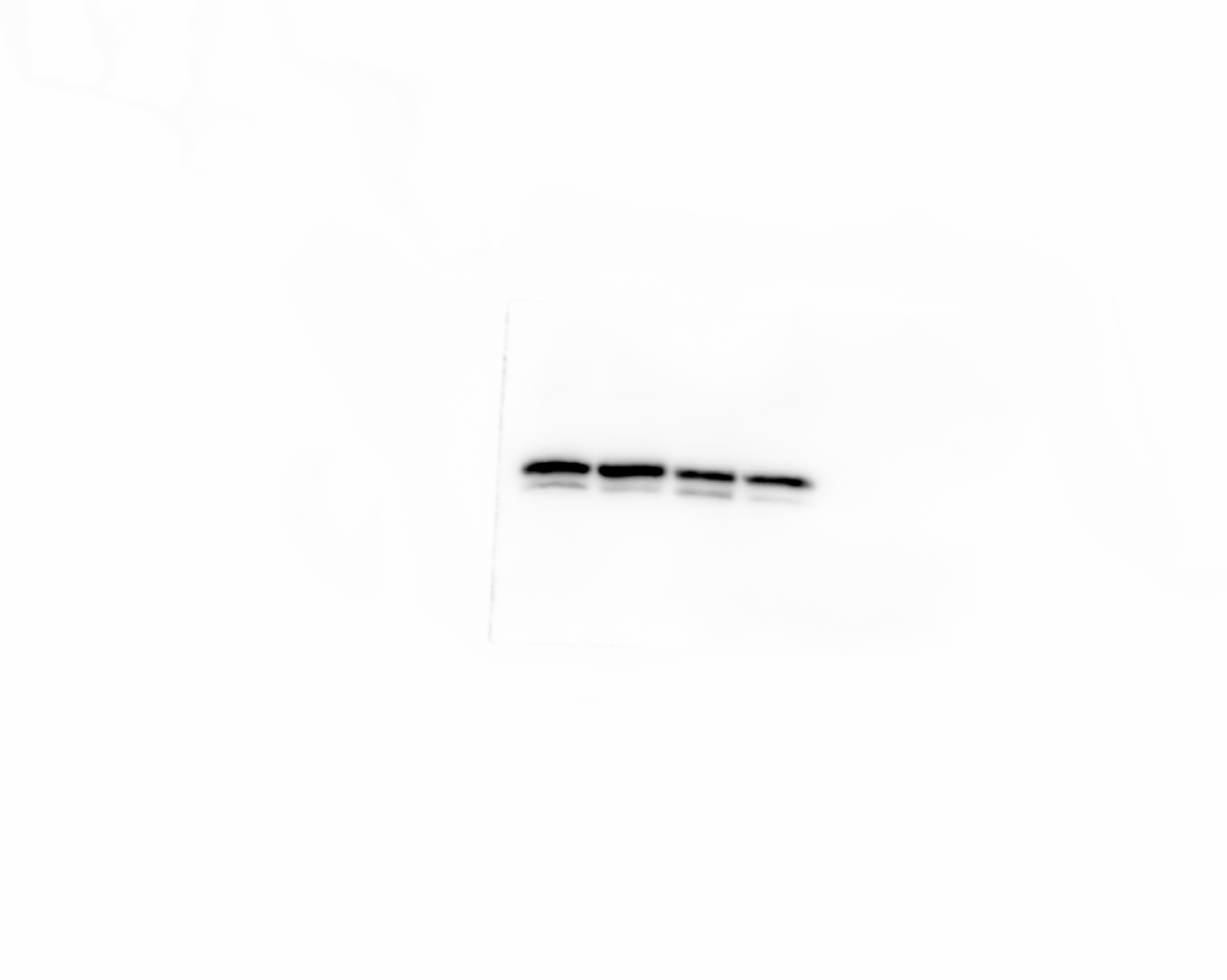

Supplement: Supplementary file 6 — Source data Fig. 4 [file 44319_2026_803_MOESM6_ESM.zip › Figure 4/4C/western A549 p-4EBP1 (Ser65).tif]

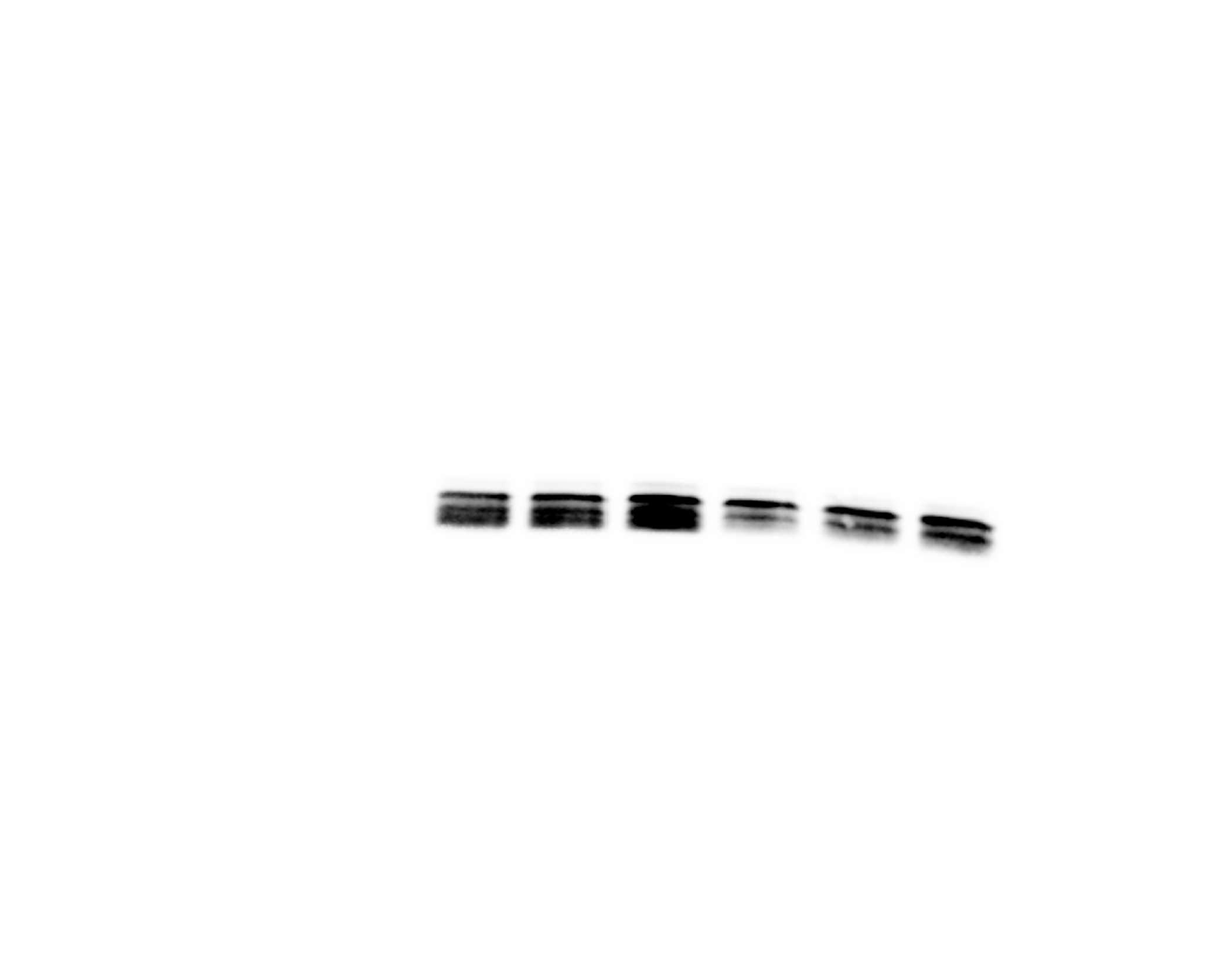

Supplement: Supplementary file 6 — Source data Fig. 4 [file 44319_2026_803_MOESM6_ESM.zip › Figure 4/4D/western 4EBP1.tif]

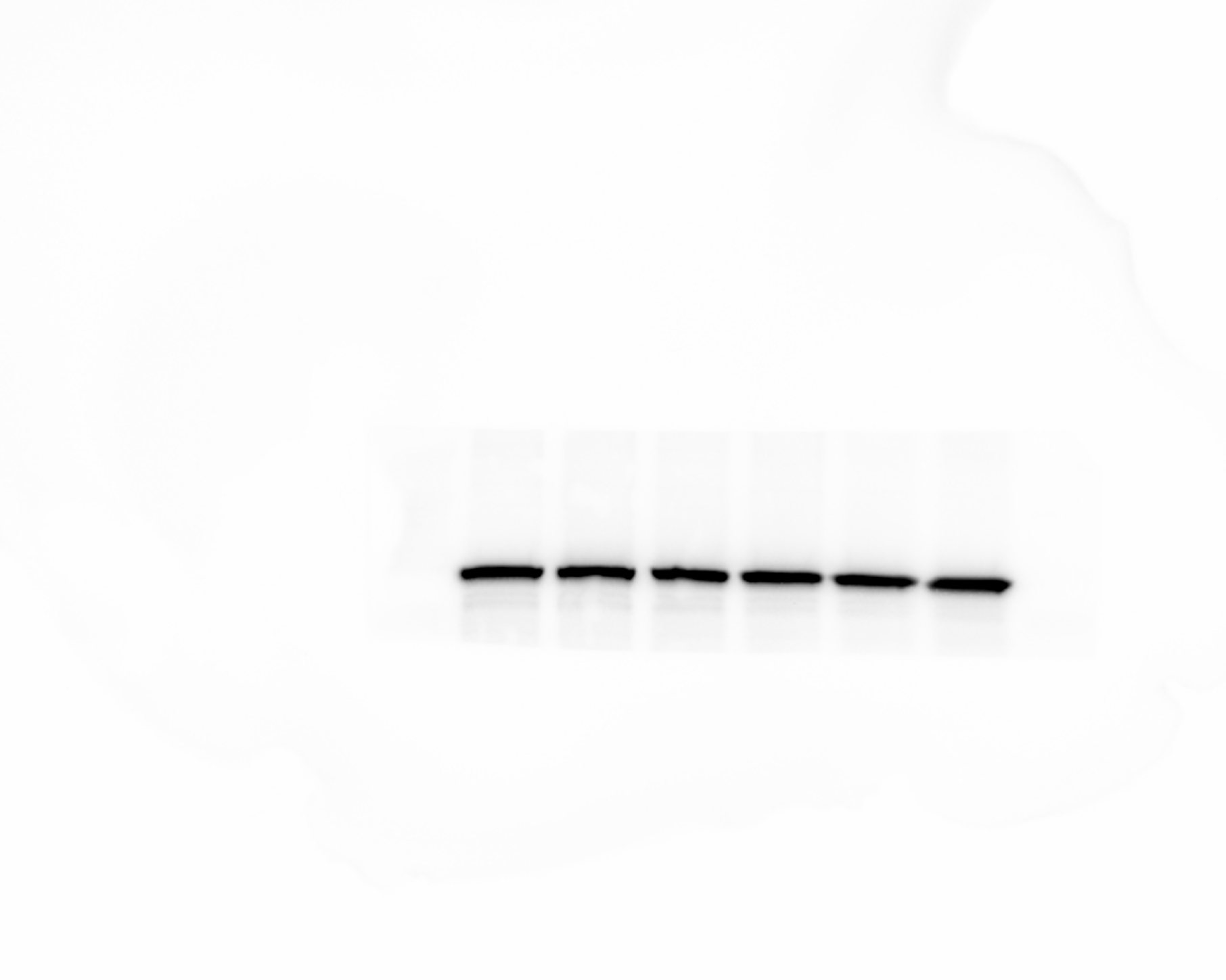

Supplement: Supplementary file 6 — Source data Fig. 4 [file 44319_2026_803_MOESM6_ESM.zip › Figure 4/4D/western HSP90.tif]

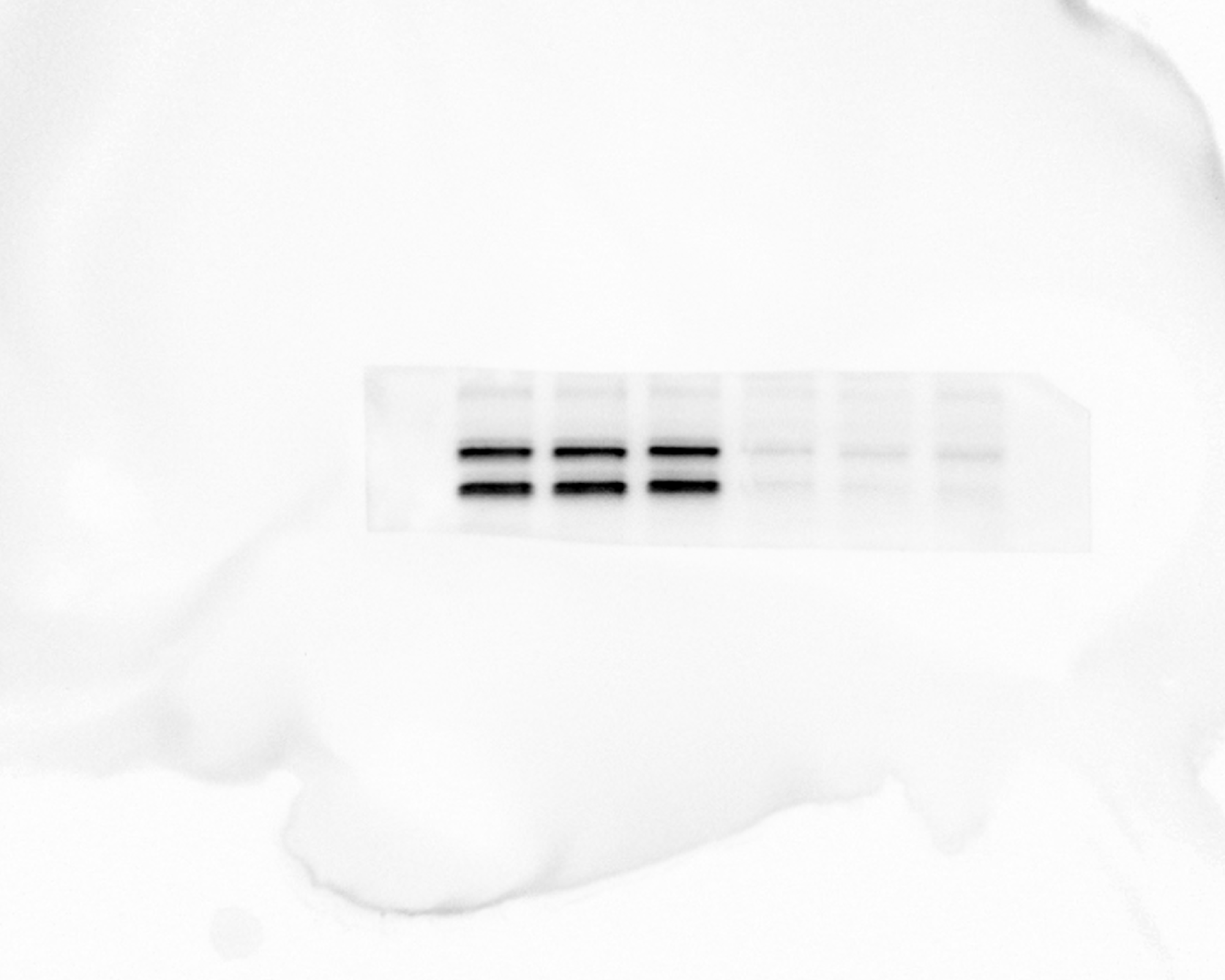

Supplement: Supplementary file 6 — Source data Fig. 4 [file 44319_2026_803_MOESM6_ESM.zip › Figure 4/4D/western SIRT2.tif]

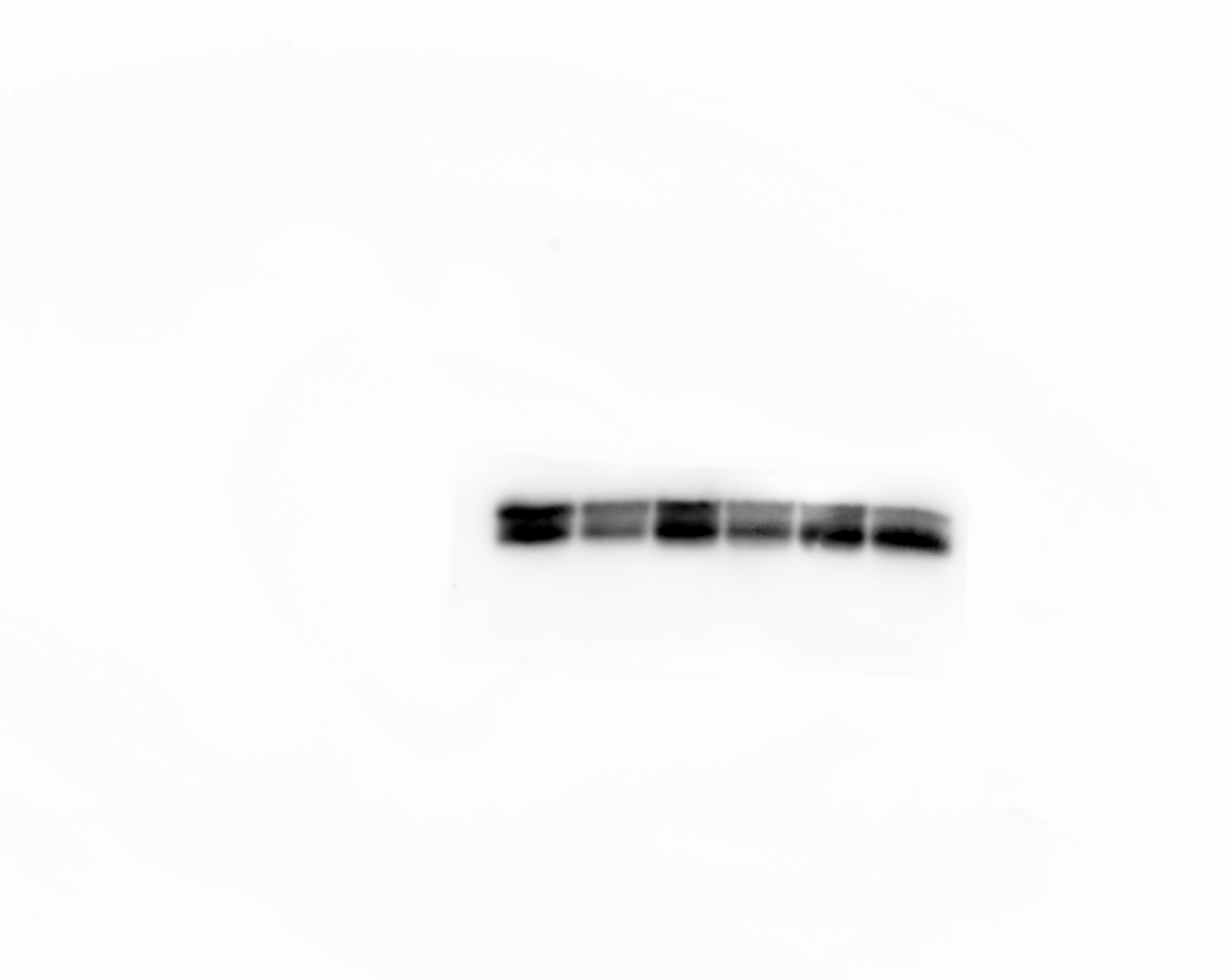

Supplement: Supplementary file 6 — Source data Fig. 4 [file 44319_2026_803_MOESM6_ESM.zip › Figure 4/4F/western 4EBP1.tif]

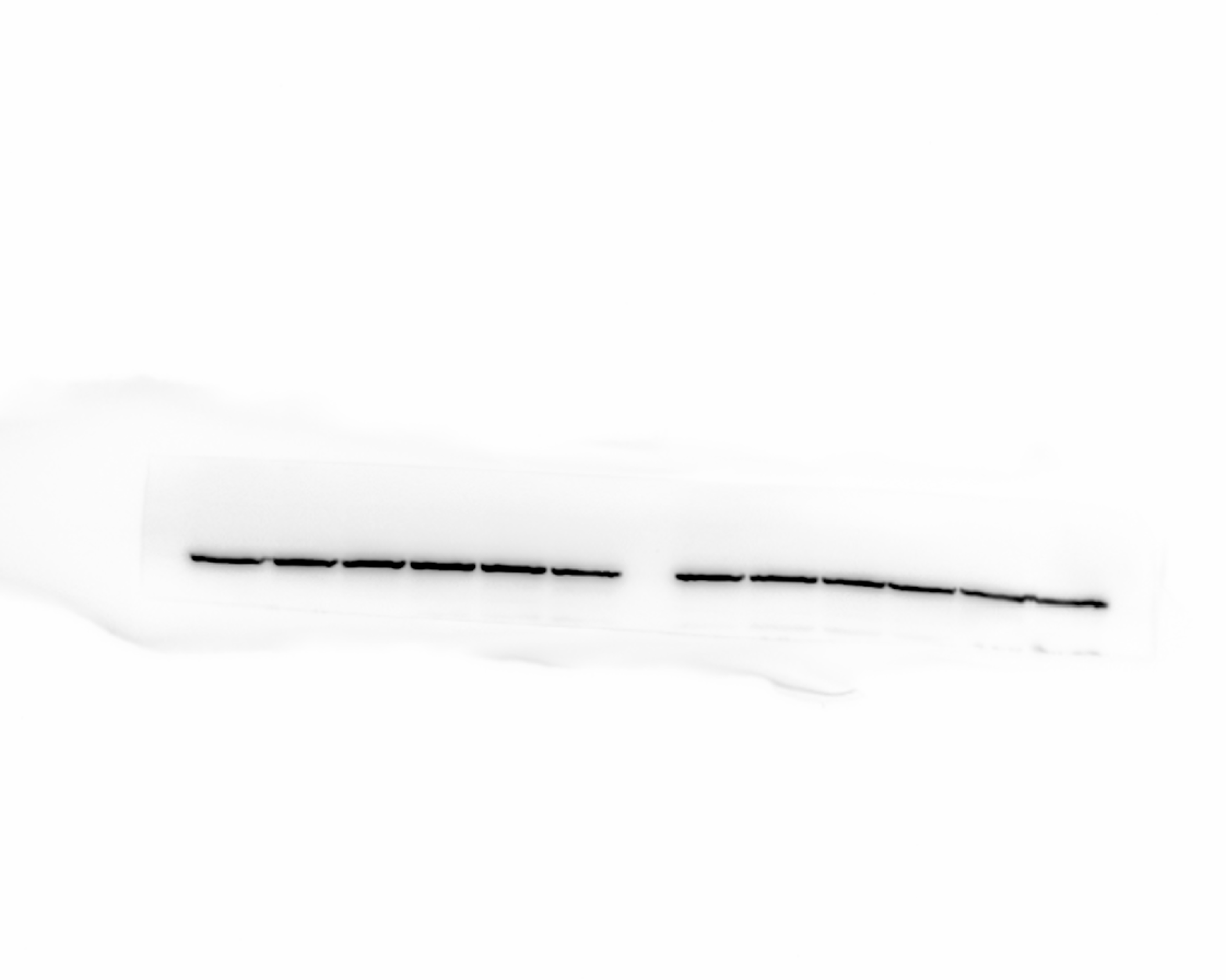

Supplement: Supplementary file 6 — Source data Fig. 4 [file 44319_2026_803_MOESM6_ESM.zip › Figure 4/4F/western HSP90.tif]

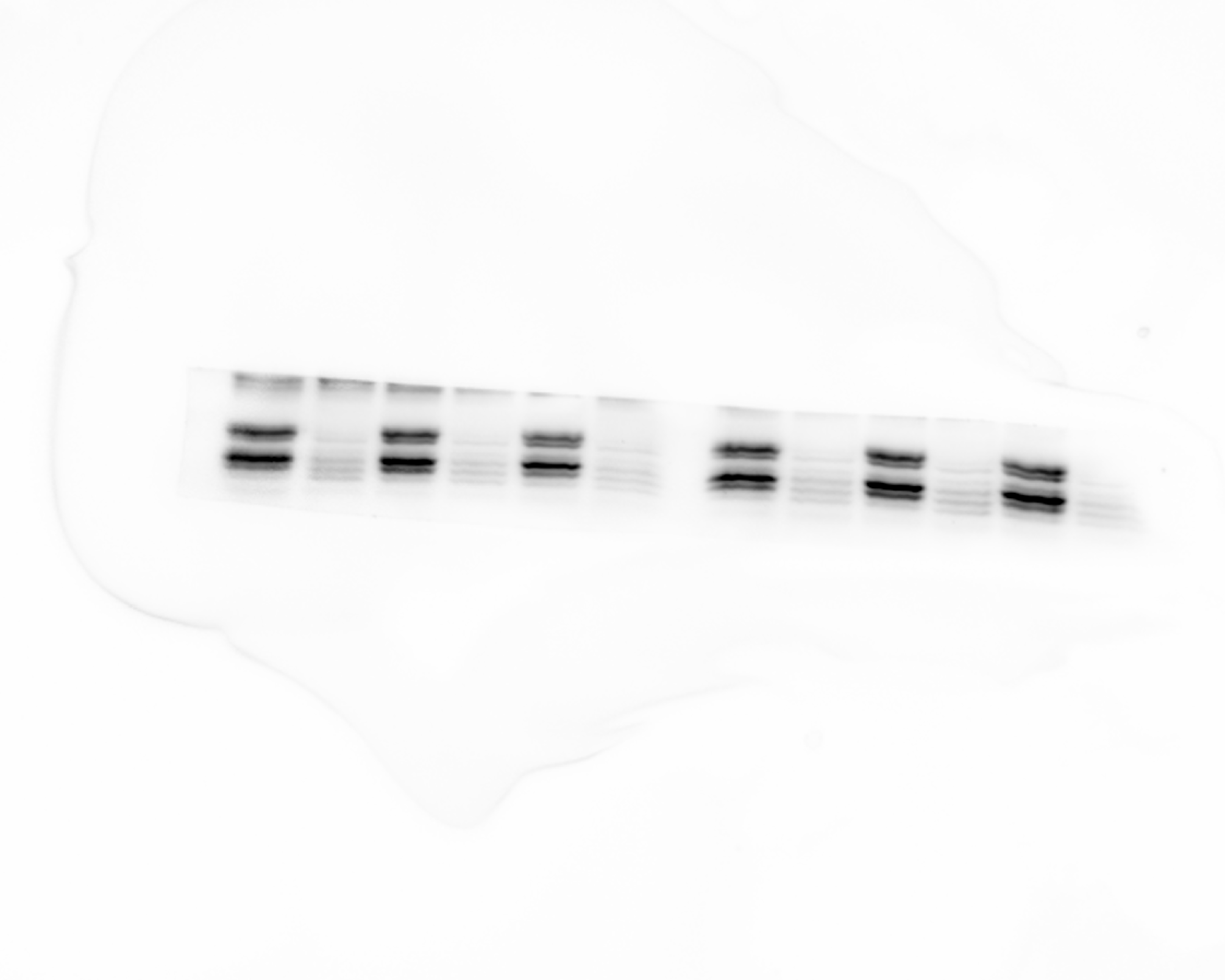

Supplement: Supplementary file 6 — Source data Fig. 4 [file 44319_2026_803_MOESM6_ESM.zip › Figure 4/4F/western SIRT2.tif]

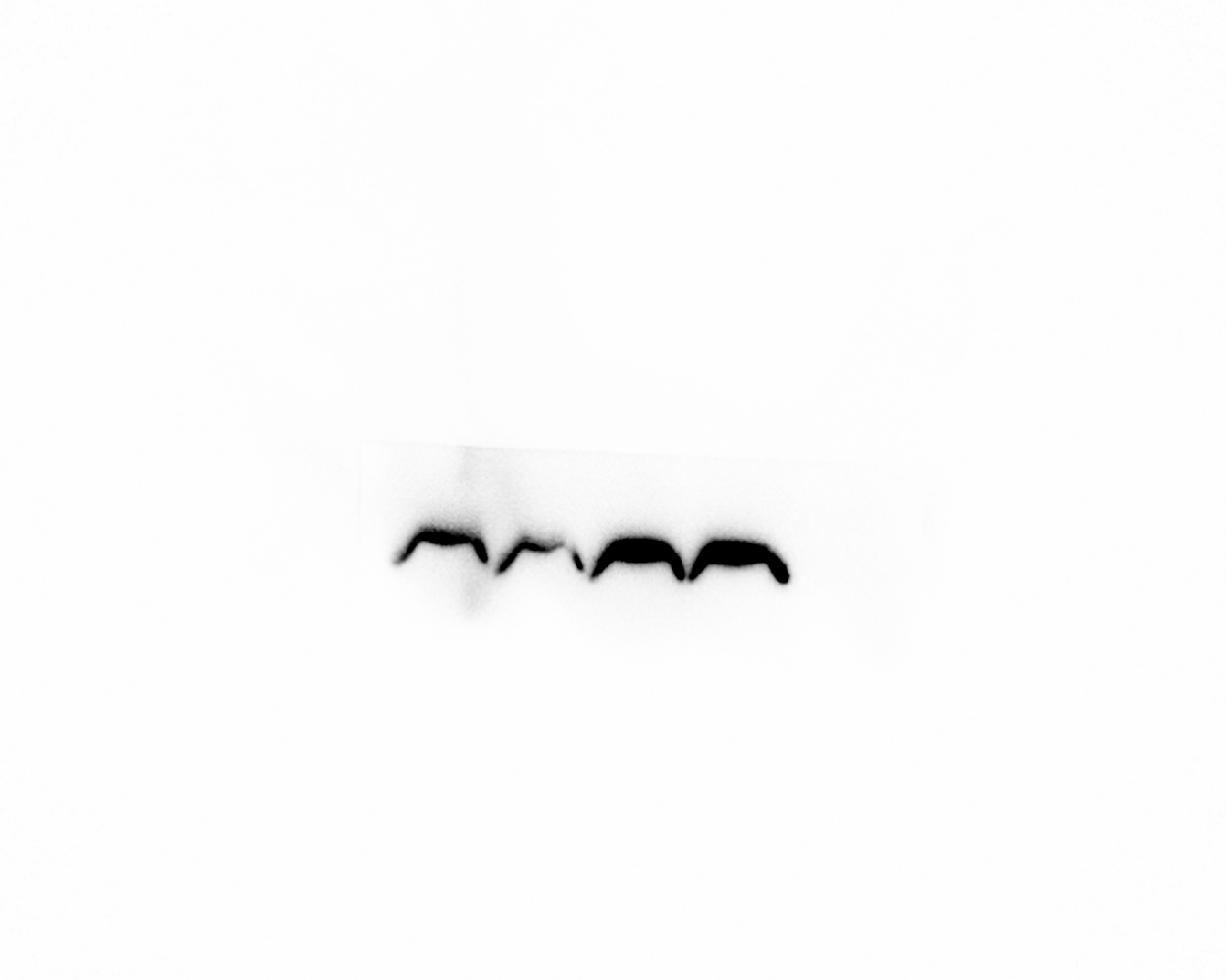

Supplement: Supplementary file 6 — Source data Fig. 4 [file 44319_2026_803_MOESM6_ESM.zip › Figure 4/4H/western Flag (input).tif]

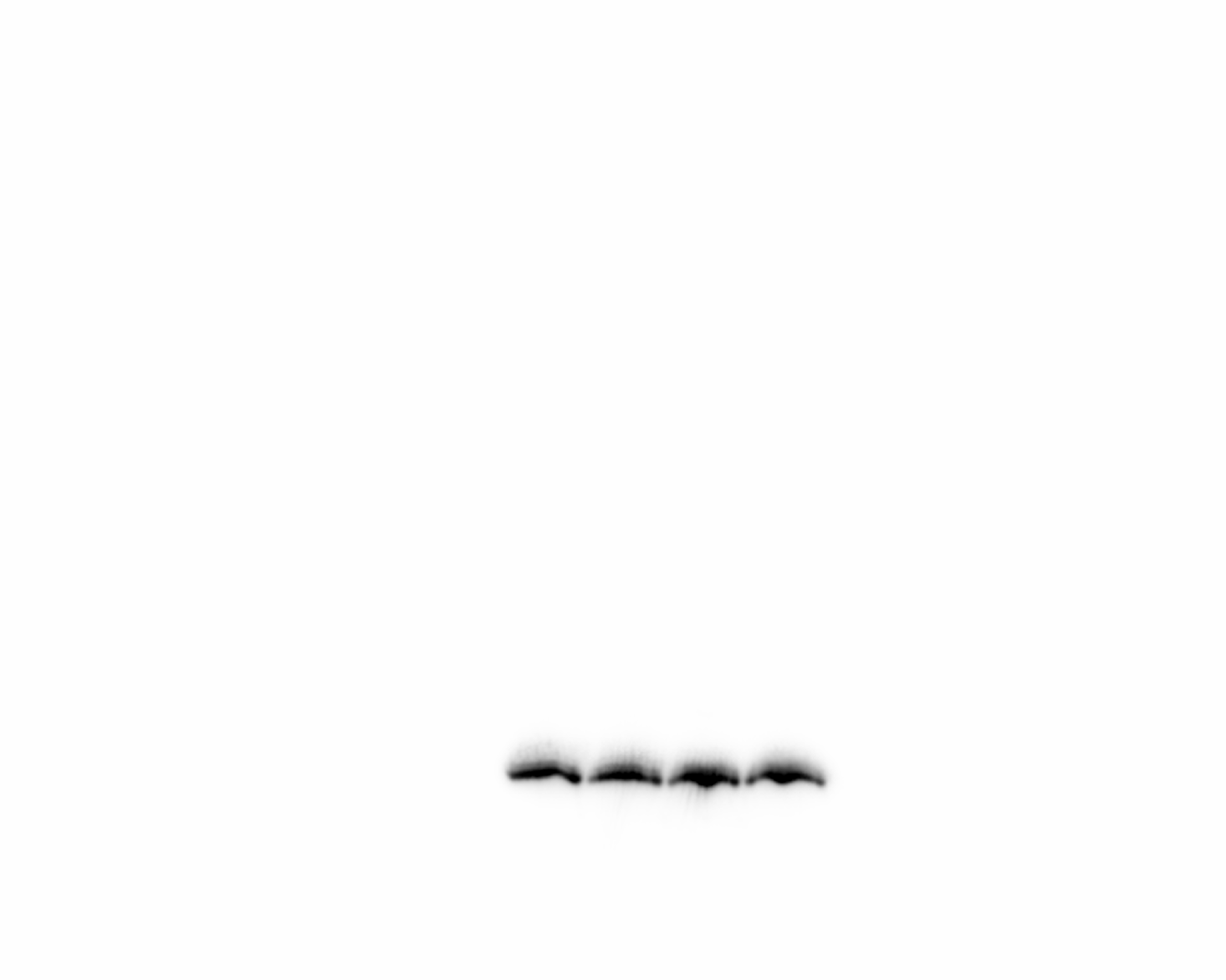

Supplement: Supplementary file 6 — Source data Fig. 4 [file 44319_2026_803_MOESM6_ESM.zip › Figure 4/4H/western Flag (IP).tif]

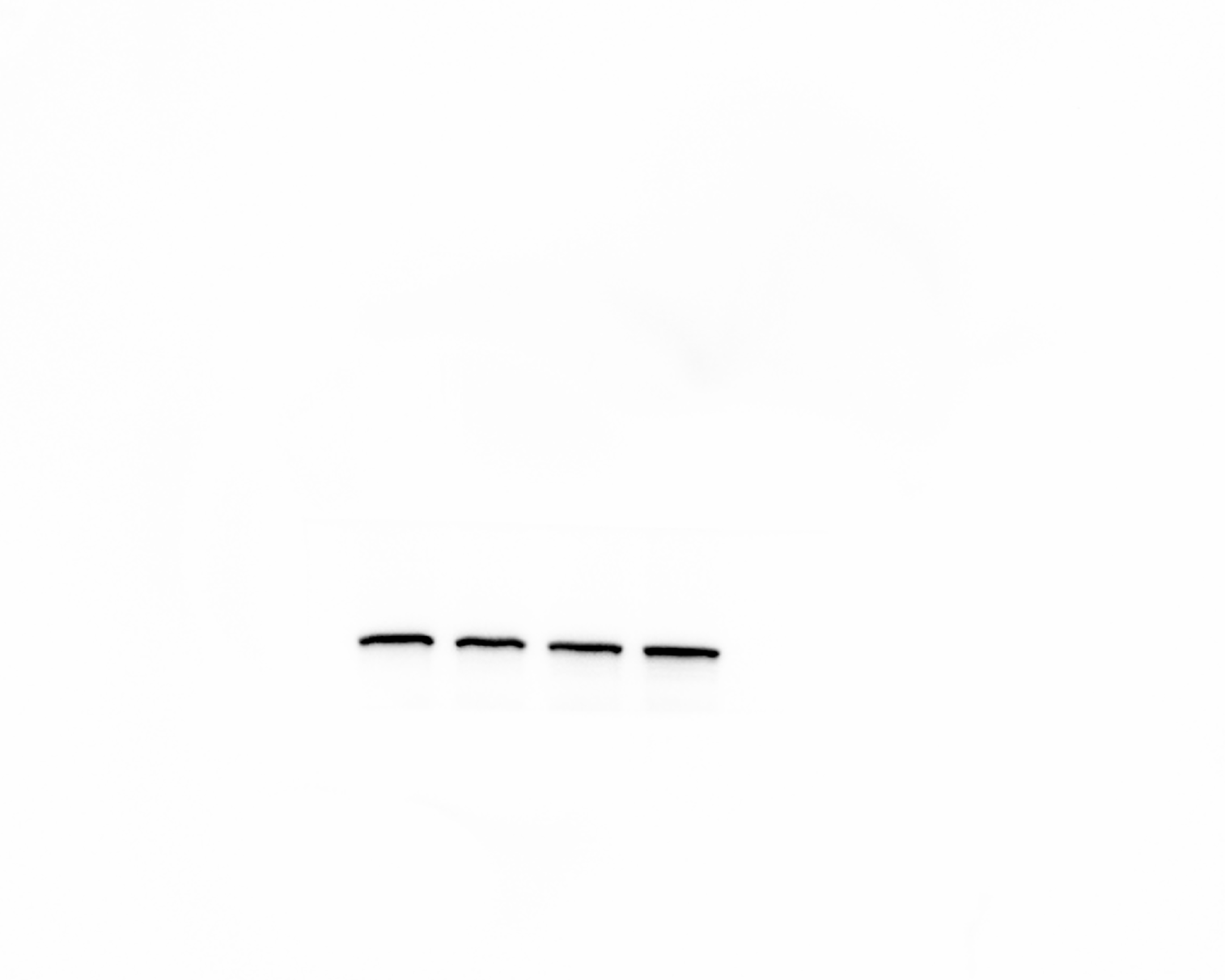

Supplement: Supplementary file 6 — Source data Fig. 4 [file 44319_2026_803_MOESM6_ESM.zip › Figure 4/4H/western HSP90 (input).tif]

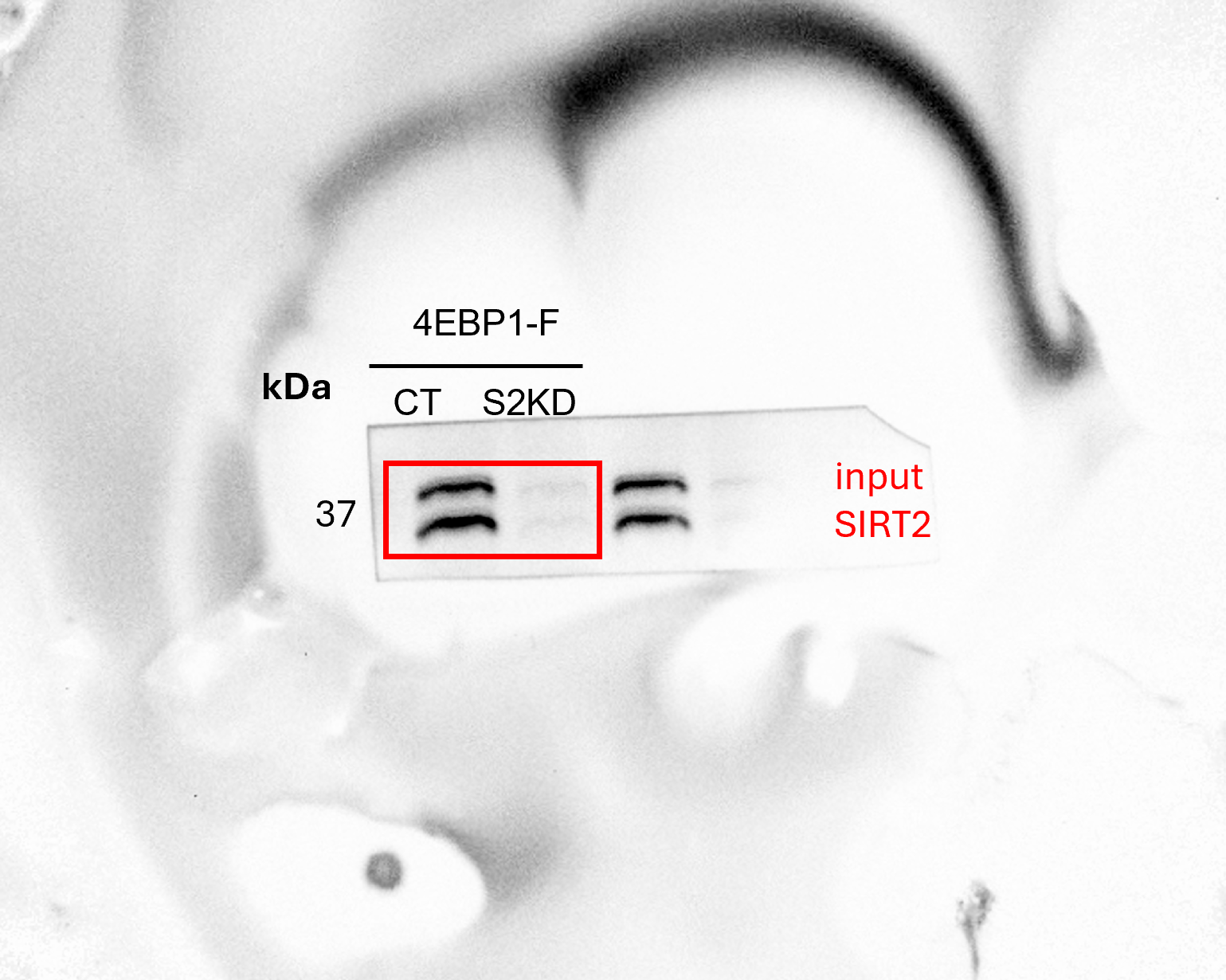

Supplement: Supplementary file 6 — Source data Fig. 4 [file 44319_2026_803_MOESM6_ESM.zip › Figure 4/4H/western SIRT2 (input).tif]

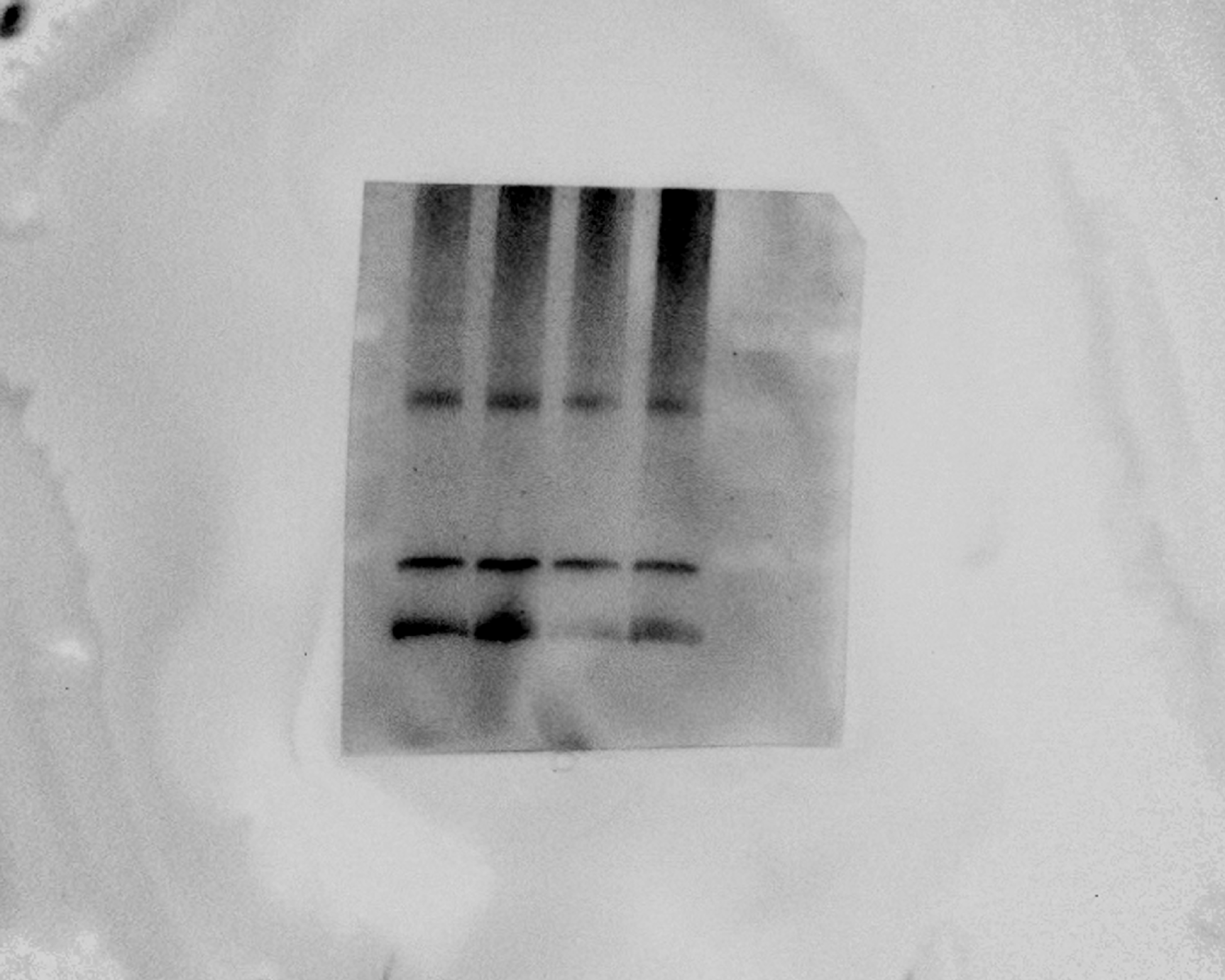

Supplement: Supplementary file 6 — Source data Fig. 4 [file 44319_2026_803_MOESM6_ESM.zip › Figure 4/4H/western Ub (IP).tif]

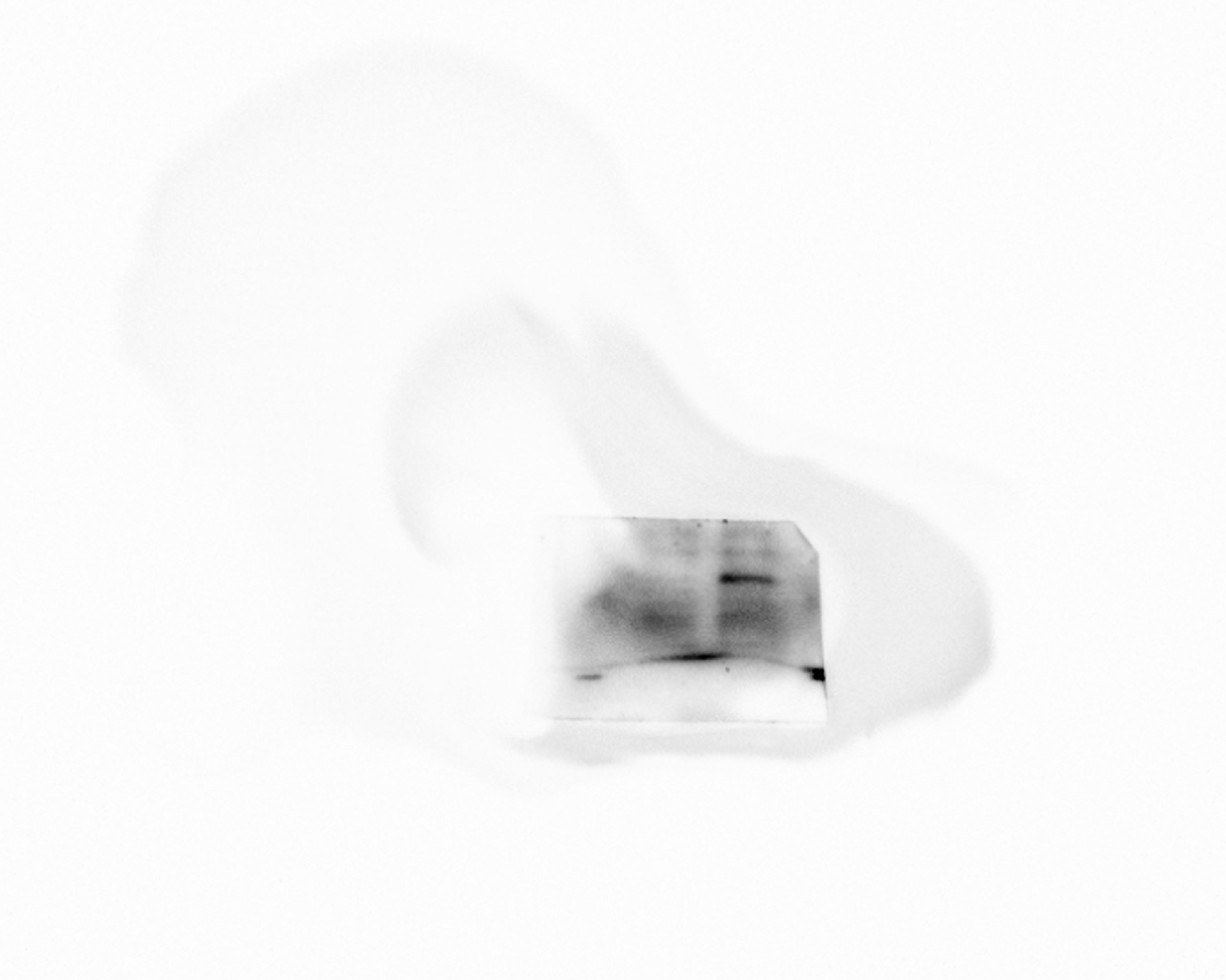

Supplement: Supplementary file 7 — Source data Fig. 5 [file 44319_2026_803_MOESM7_ESM.zip › Figure 5/5A/western Flag (input).tif]

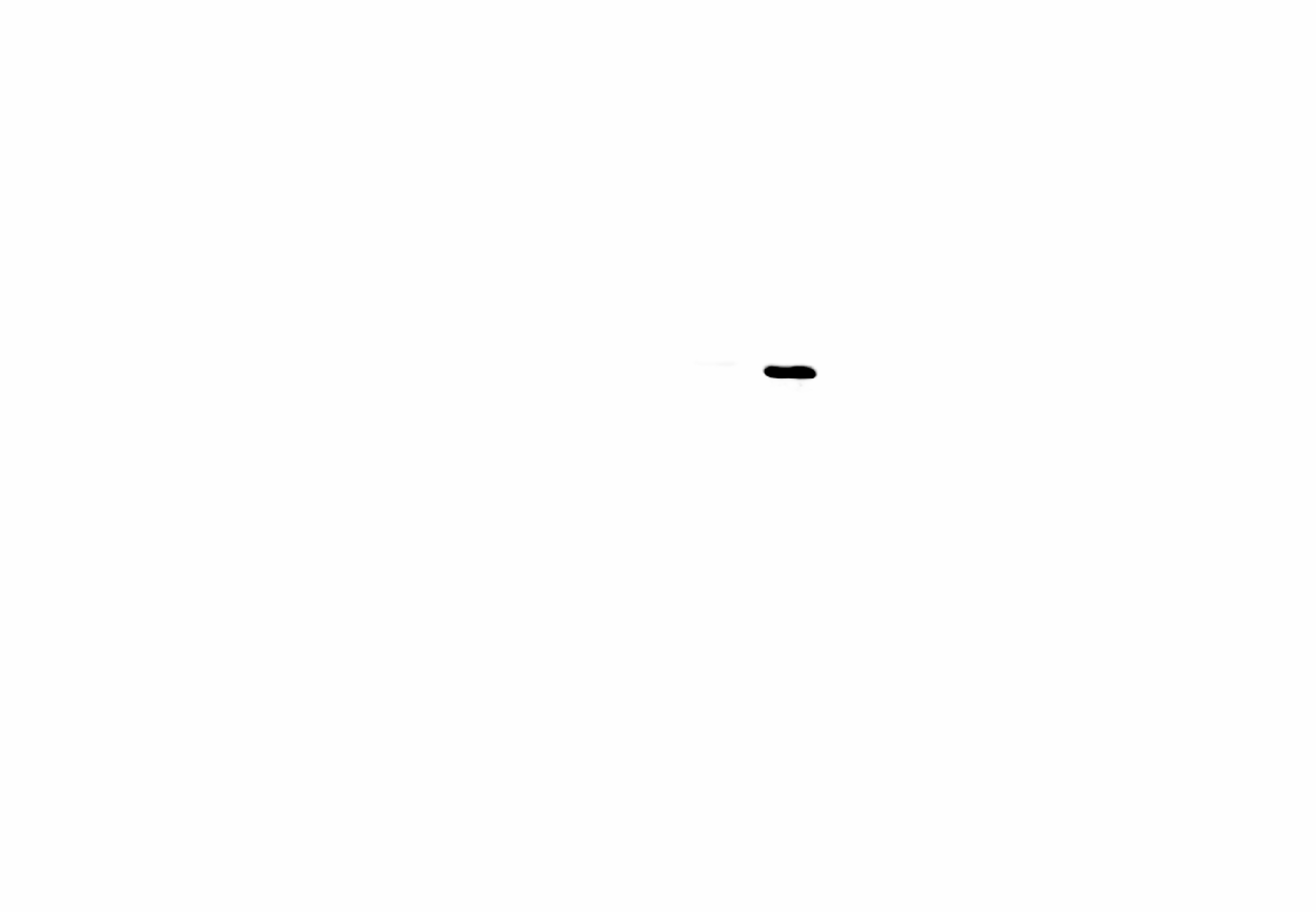

Supplement: Supplementary file 7 — Source data Fig. 5 [file 44319_2026_803_MOESM7_ESM.zip › Figure 5/5A/western Flag (IP).tif]

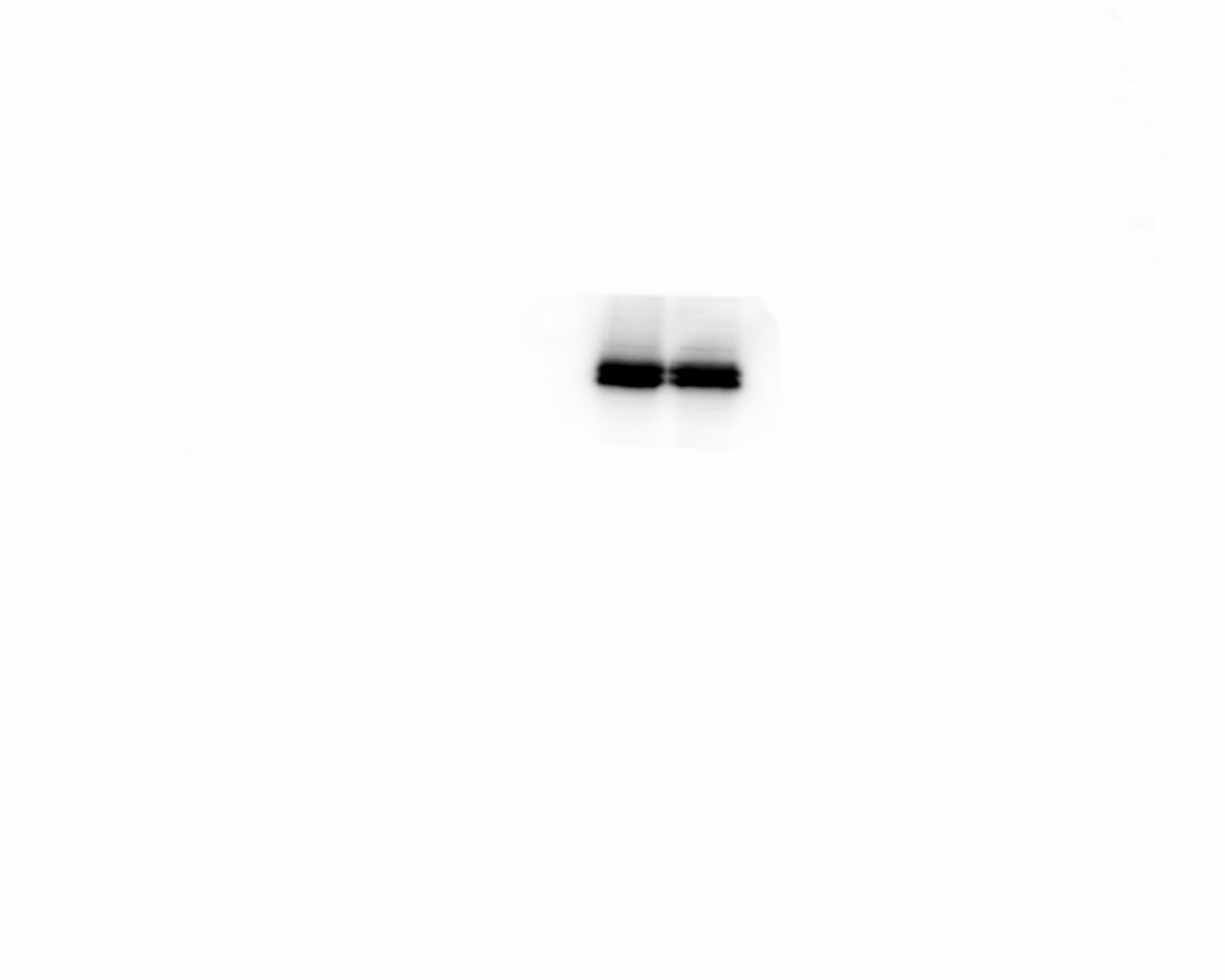

Supplement: Supplementary file 7 — Source data Fig. 5 [file 44319_2026_803_MOESM7_ESM.zip › Figure 5/5A/western HA (input).tif]

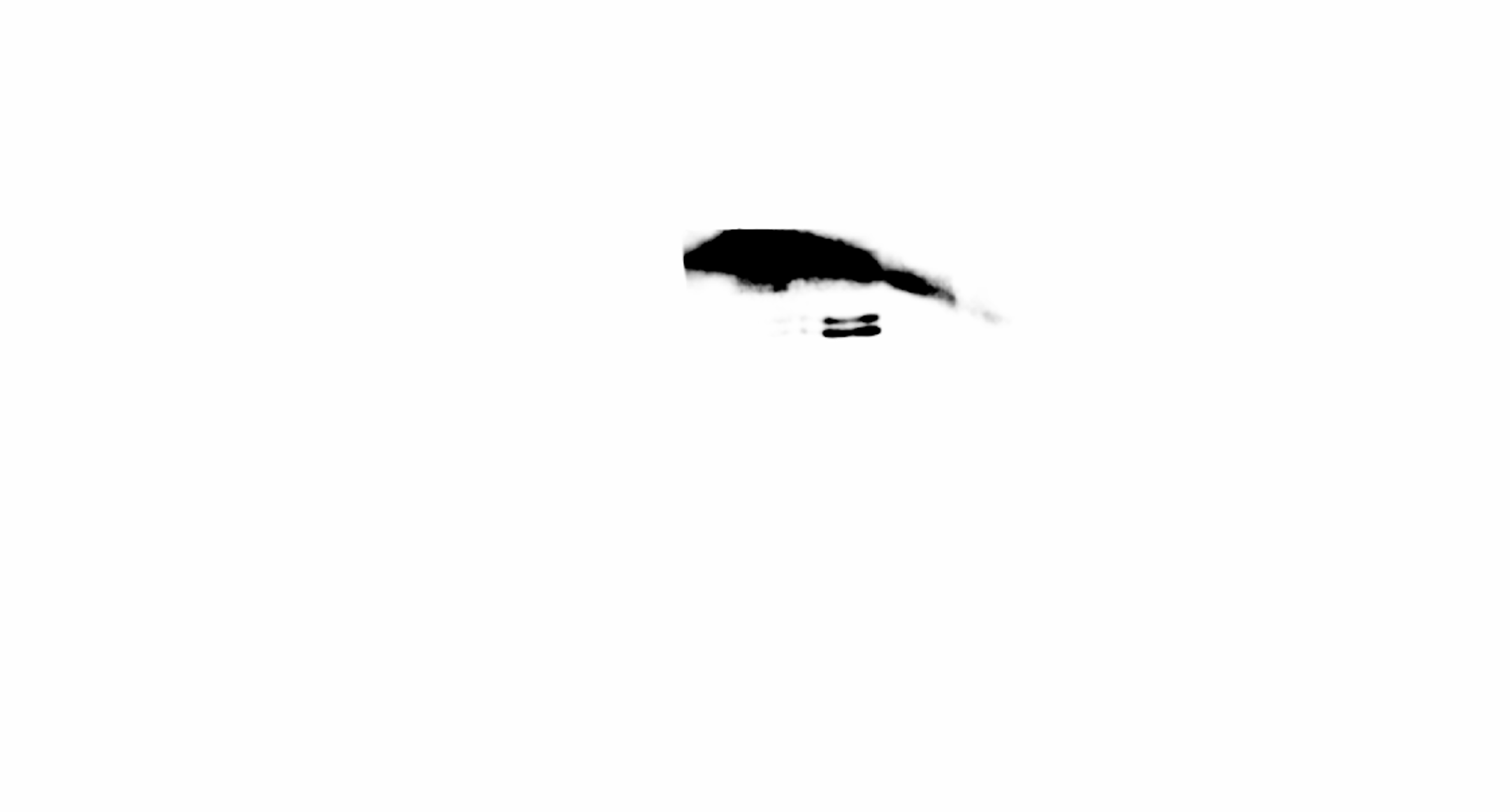

Supplement: Supplementary file 7 — Source data Fig. 5 [file 44319_2026_803_MOESM7_ESM.zip › Figure 5/5A/western HA (IP).tif]

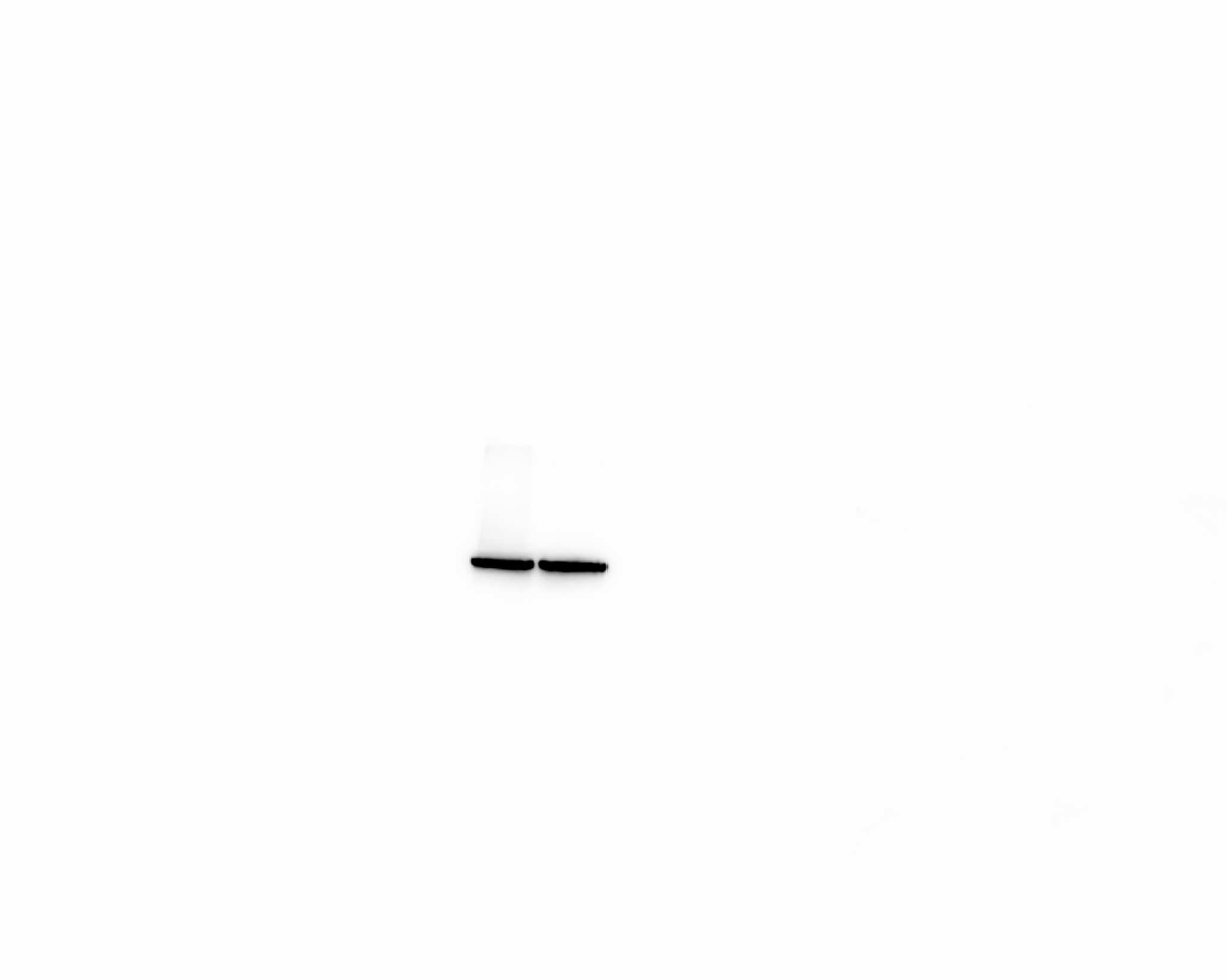

Supplement: Supplementary file 7 — Source data Fig. 5 [file 44319_2026_803_MOESM7_ESM.zip › Figure 5/5A/western HSP90 (input).tif]

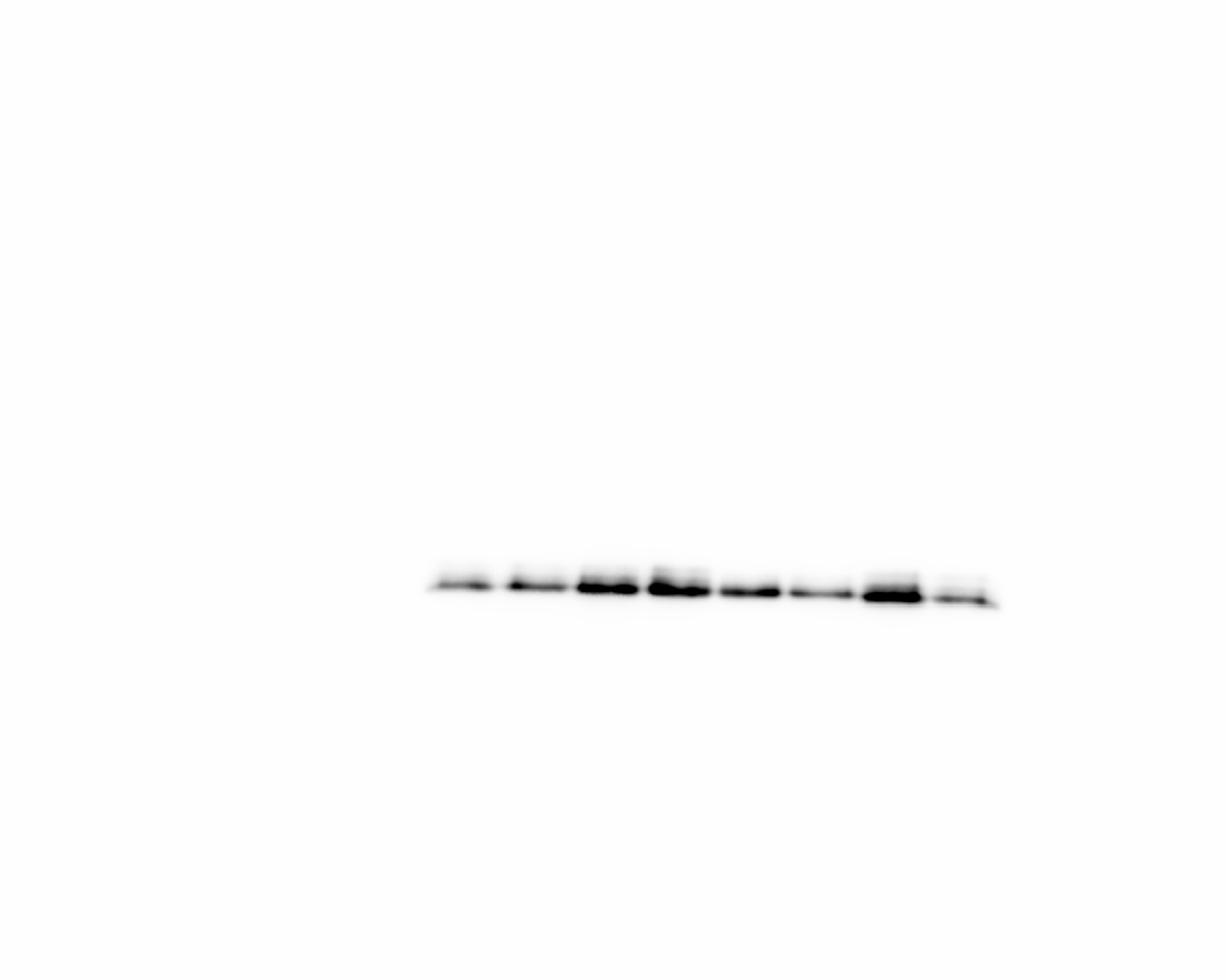

Supplement: Supplementary file 7 — Source data Fig. 5 [file 44319_2026_803_MOESM7_ESM.zip › Figure 5/5B/western Flag (input).tif]

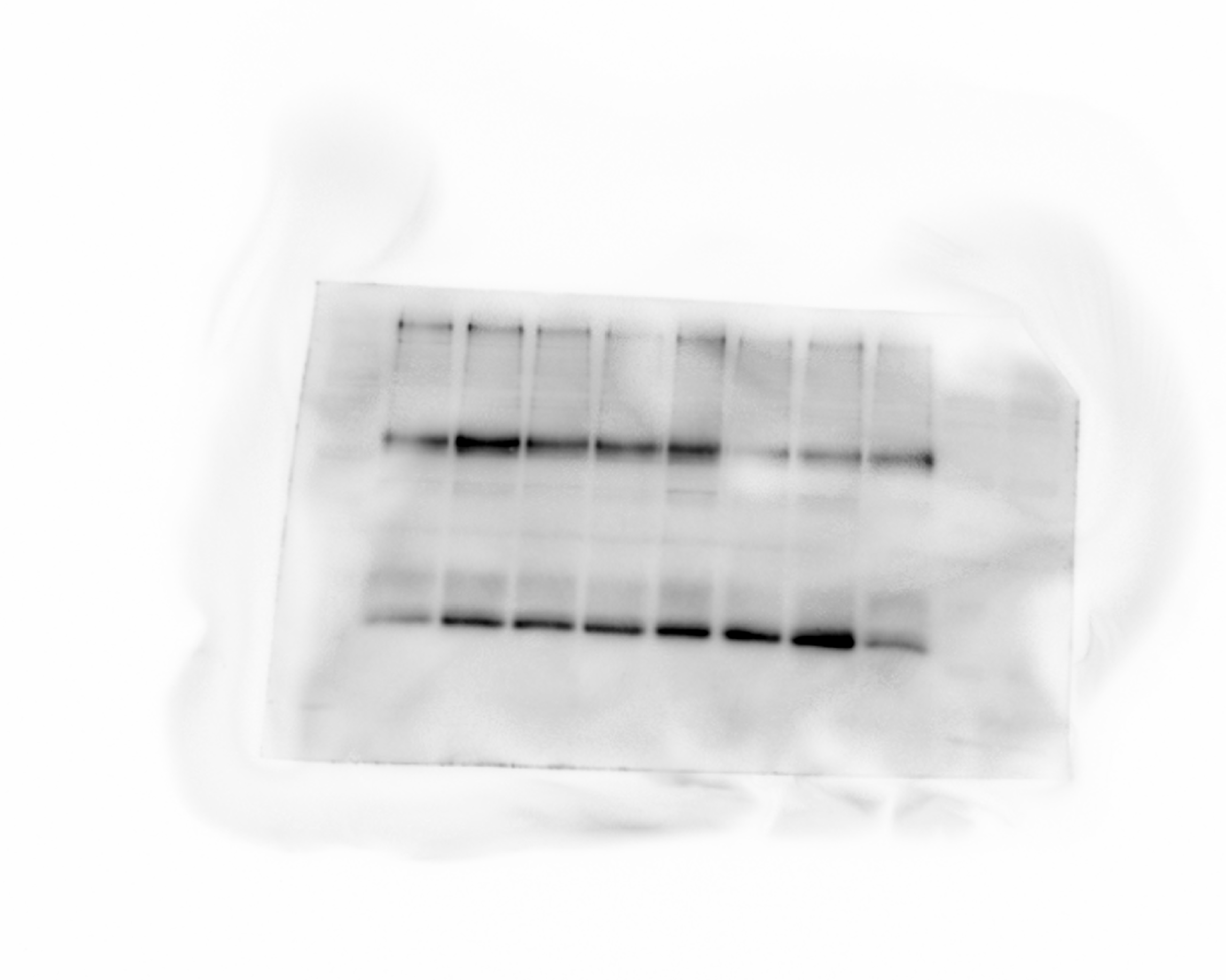

Supplement: Supplementary file 7 — Source data Fig. 5 [file 44319_2026_803_MOESM7_ESM.zip › Figure 5/5B/western Flag (IP).tif]

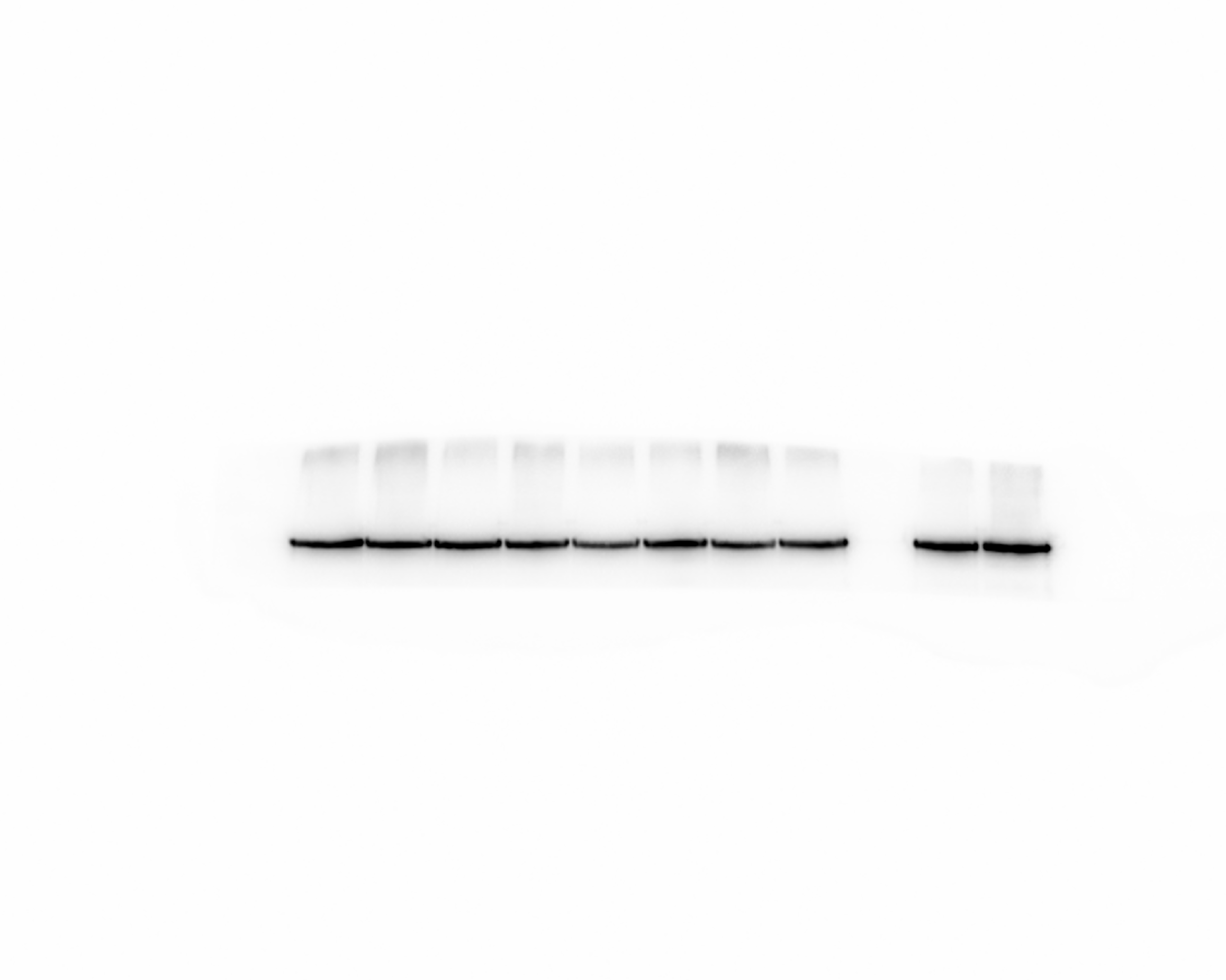

Supplement: Supplementary file 7 — Source data Fig. 5 [file 44319_2026_803_MOESM7_ESM.zip › Figure 5/5B/western HSP90 (input).tif]

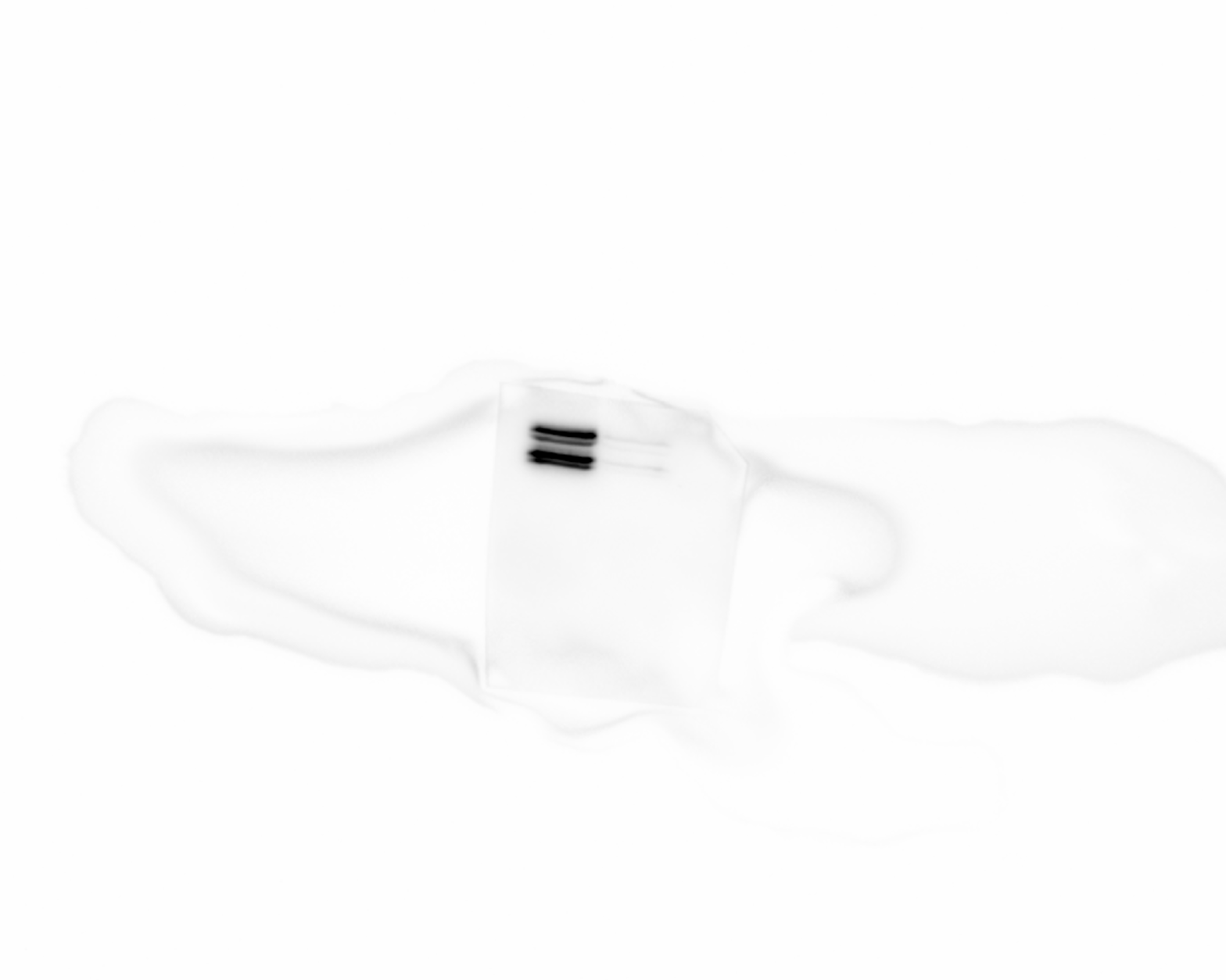

Supplement: Supplementary file 7 — Source data Fig. 5 [file 44319_2026_803_MOESM7_ESM.zip › Figure 5/5B/western SIRT2 (input).tif]

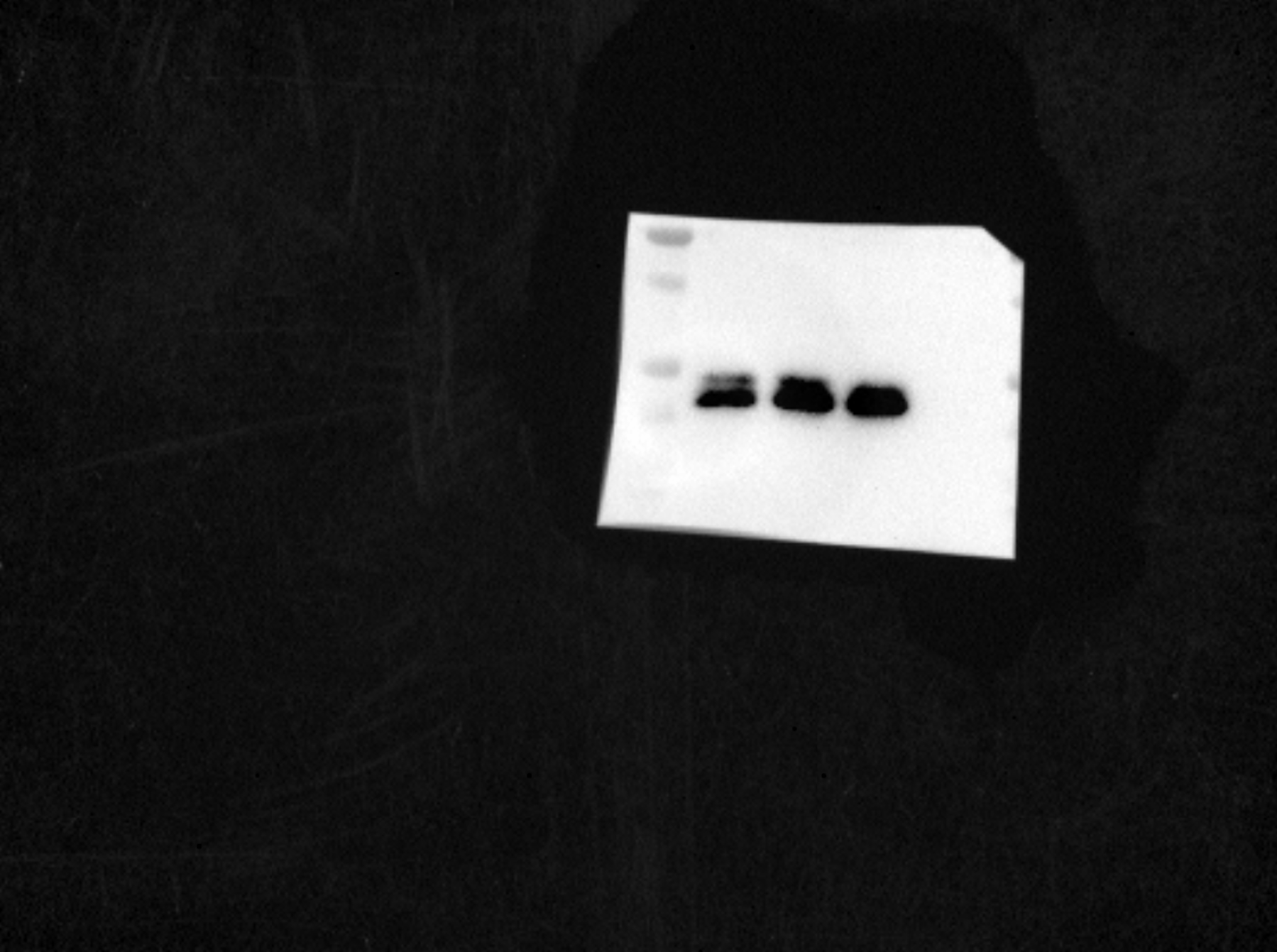

Supplement: Supplementary file 7 — Source data Fig. 5 [file 44319_2026_803_MOESM7_ESM.zip › Figure 5/5D/western Flag (input).tif]

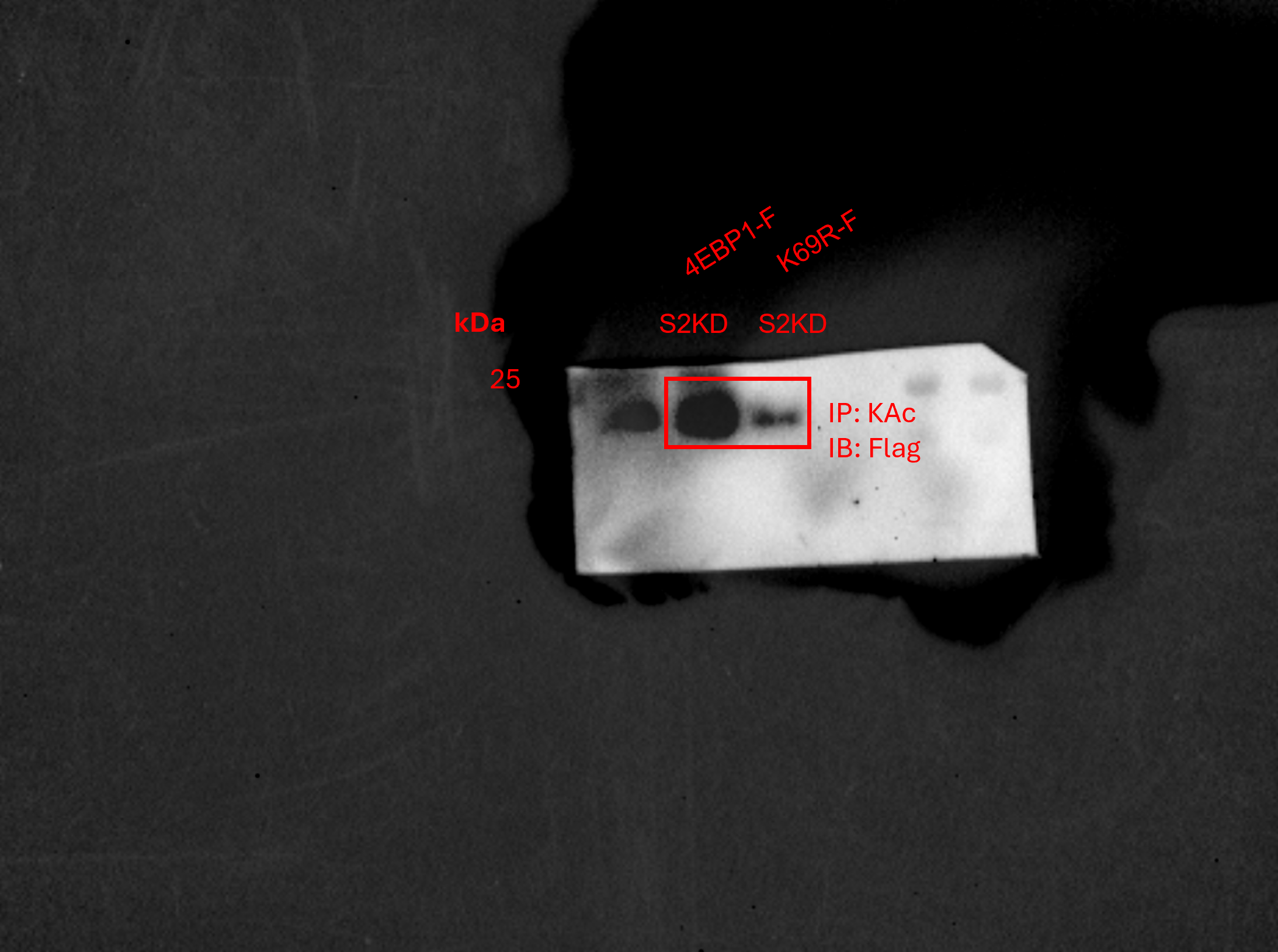

Supplement: Supplementary file 7 — Source data Fig. 5 [file 44319_2026_803_MOESM7_ESM.zip › Figure 5/5D/western Flag (IP).tif]

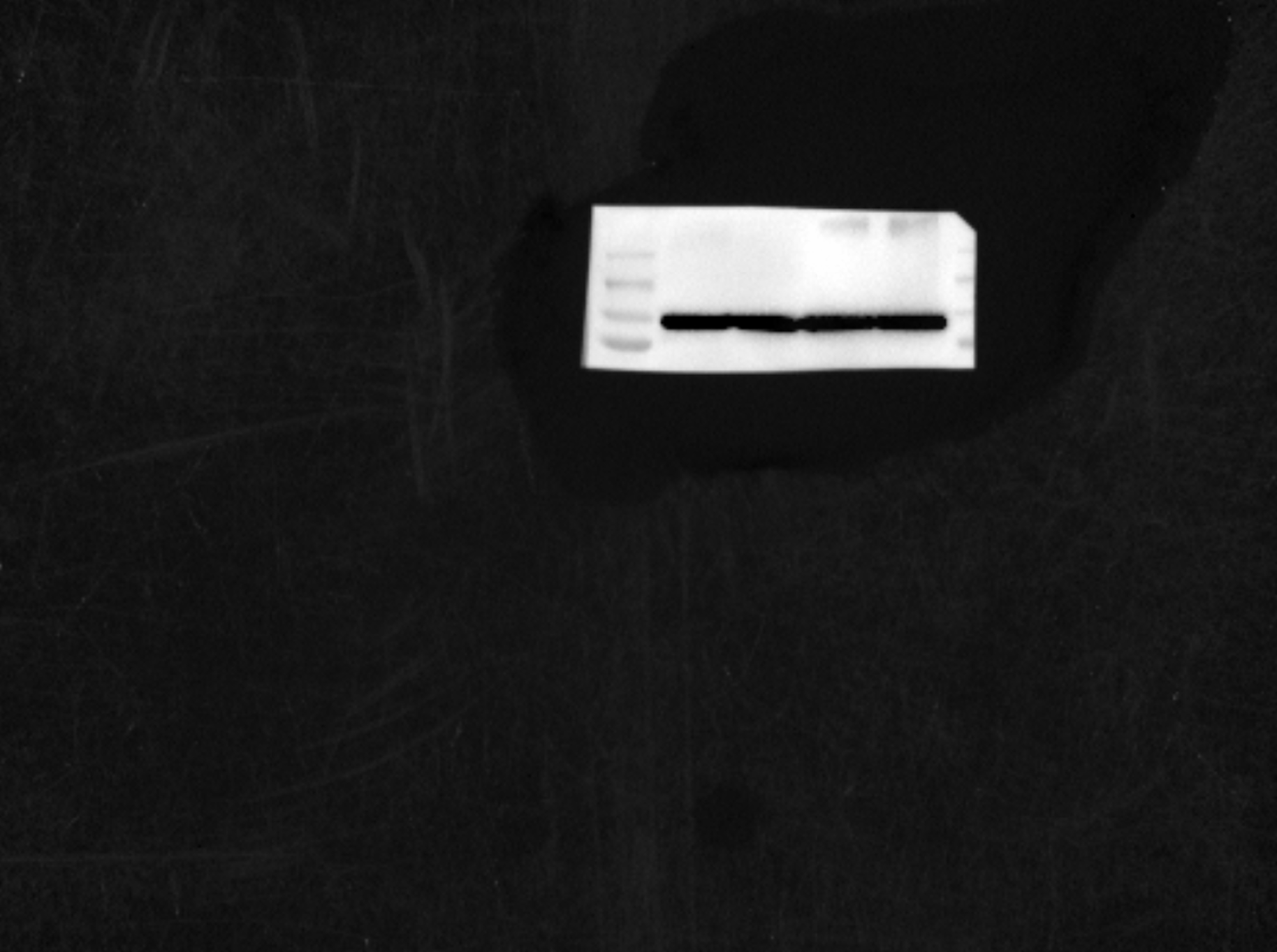

Supplement: Supplementary file 7 — Source data Fig. 5 [file 44319_2026_803_MOESM7_ESM.zip › Figure 5/5D/western HSP90 (input).tif]

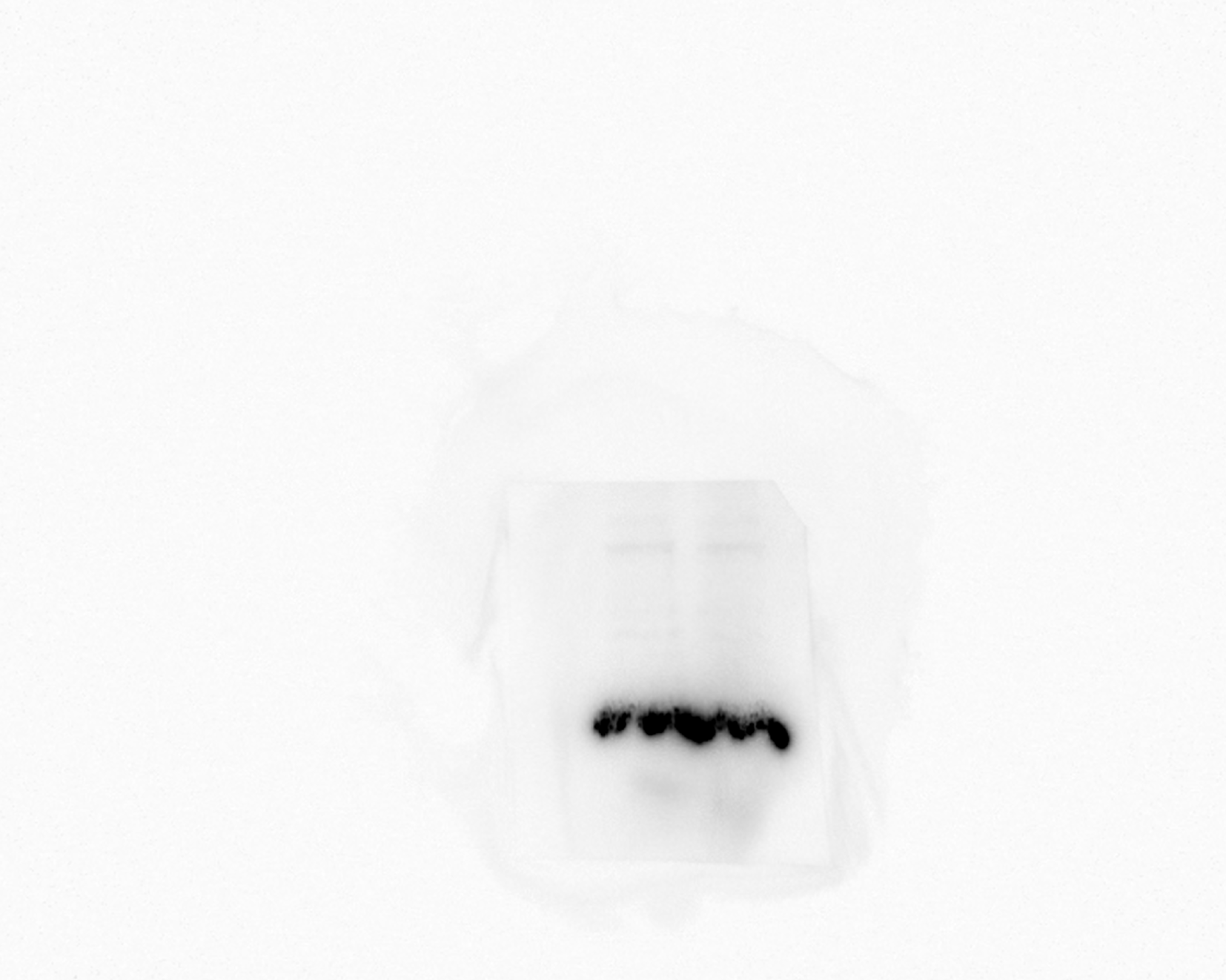

Supplement: Supplementary file 7 — Source data Fig. 5 [file 44319_2026_803_MOESM7_ESM.zip › Figure 5/5F/western Flag (input).tif]

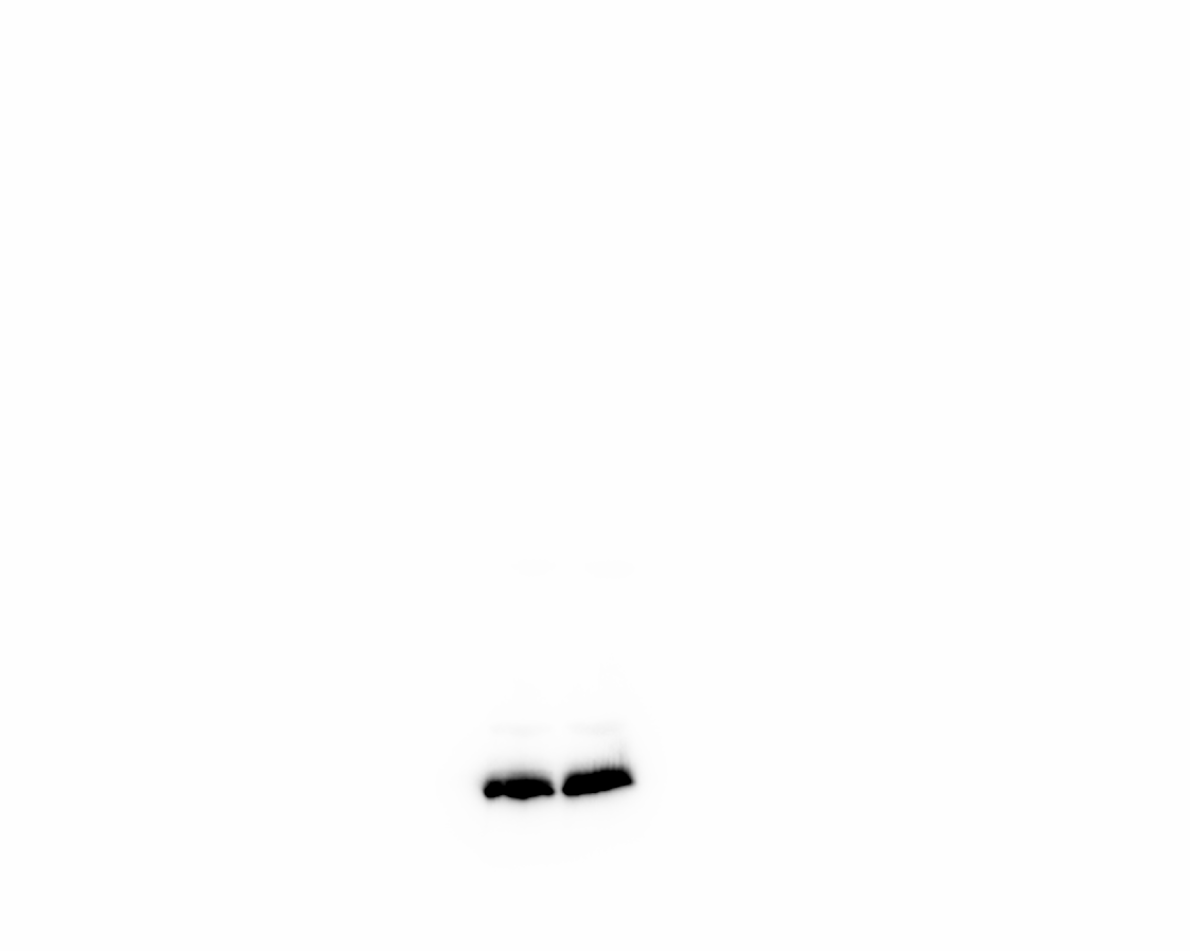

Supplement: Supplementary file 7 — Source data Fig. 5 [file 44319_2026_803_MOESM7_ESM.zip › Figure 5/5F/western Flag (IP).tif]

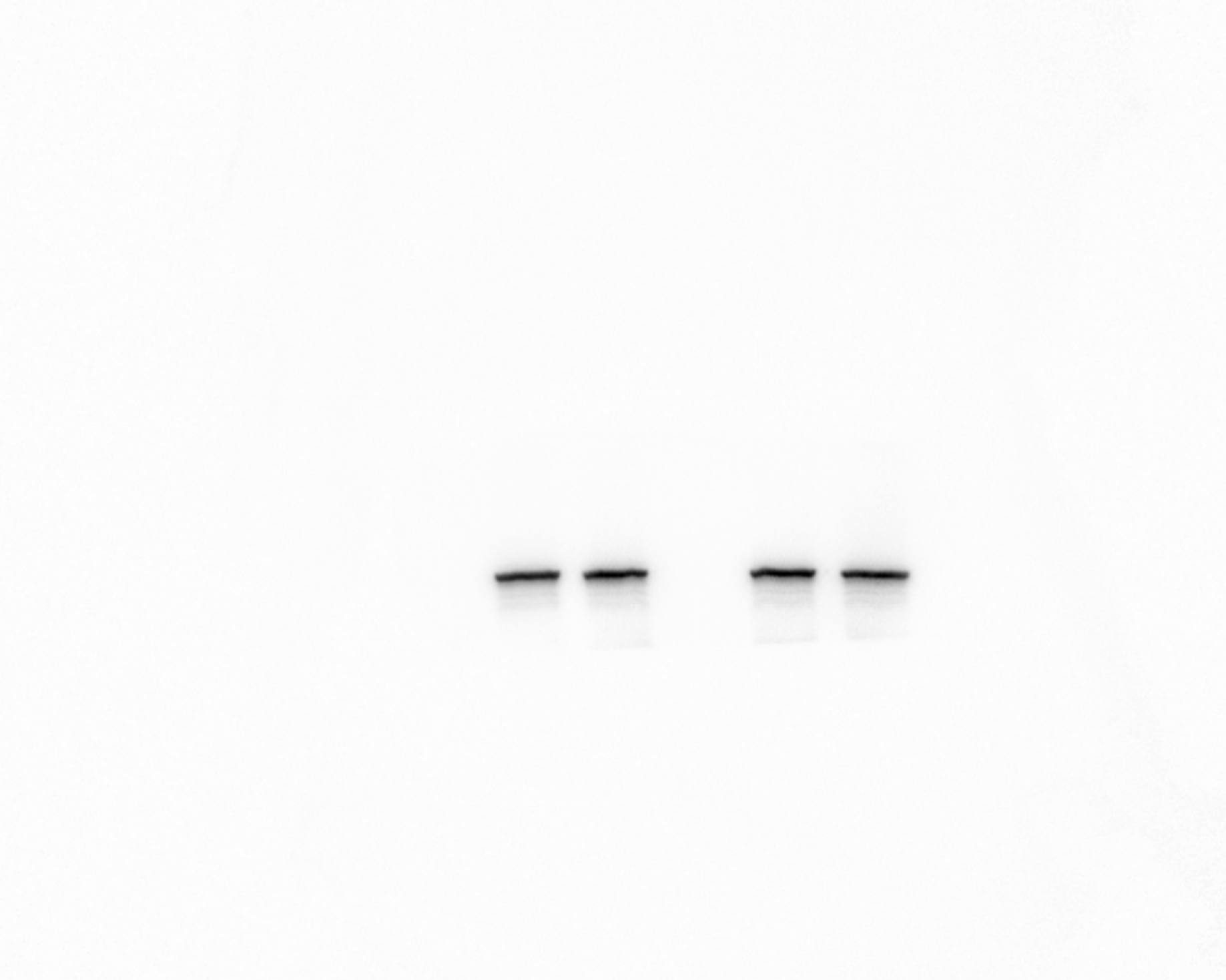

Supplement: Supplementary file 7 — Source data Fig. 5 [file 44319_2026_803_MOESM7_ESM.zip › Figure 5/5F/western HSP90 (input).tif]

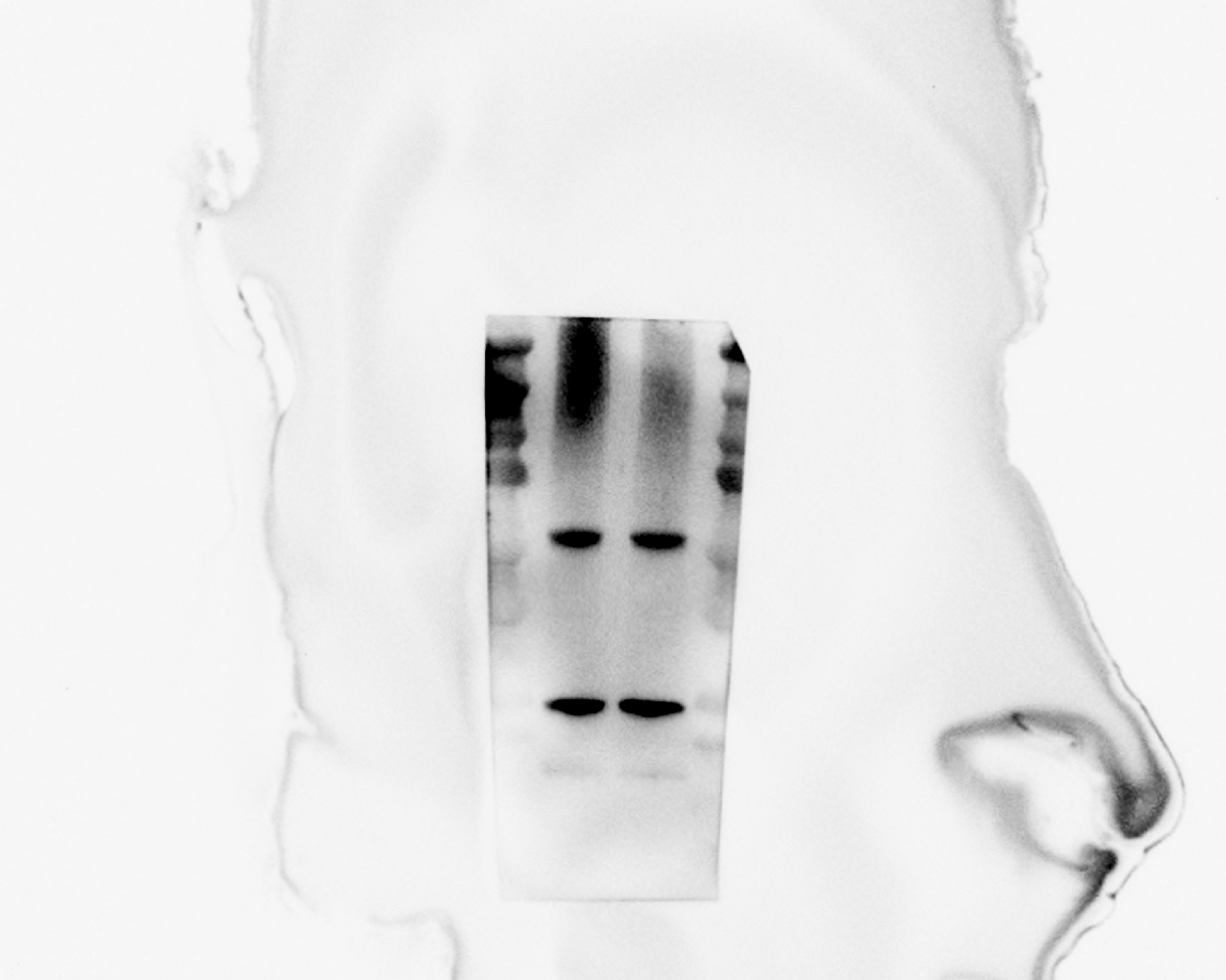

Supplement: Supplementary file 7 — Source data Fig. 5 [file 44319_2026_803_MOESM7_ESM.zip › Figure 5/5F/western Ub (IP).tif]

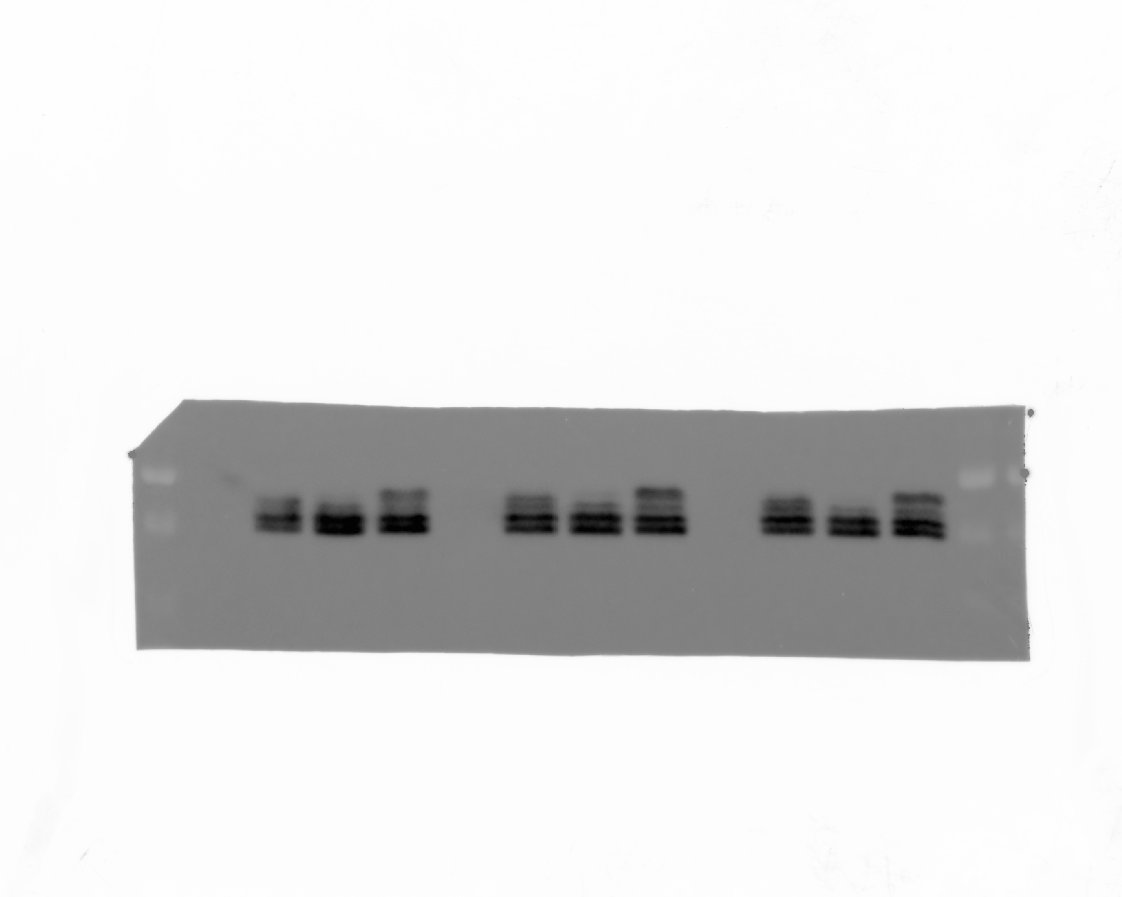

Supplement: Supplementary file 7 — Source data Fig. 5 [file 44319_2026_803_MOESM7_ESM.zip › Figure 5/5H/western Flag (input).tif]

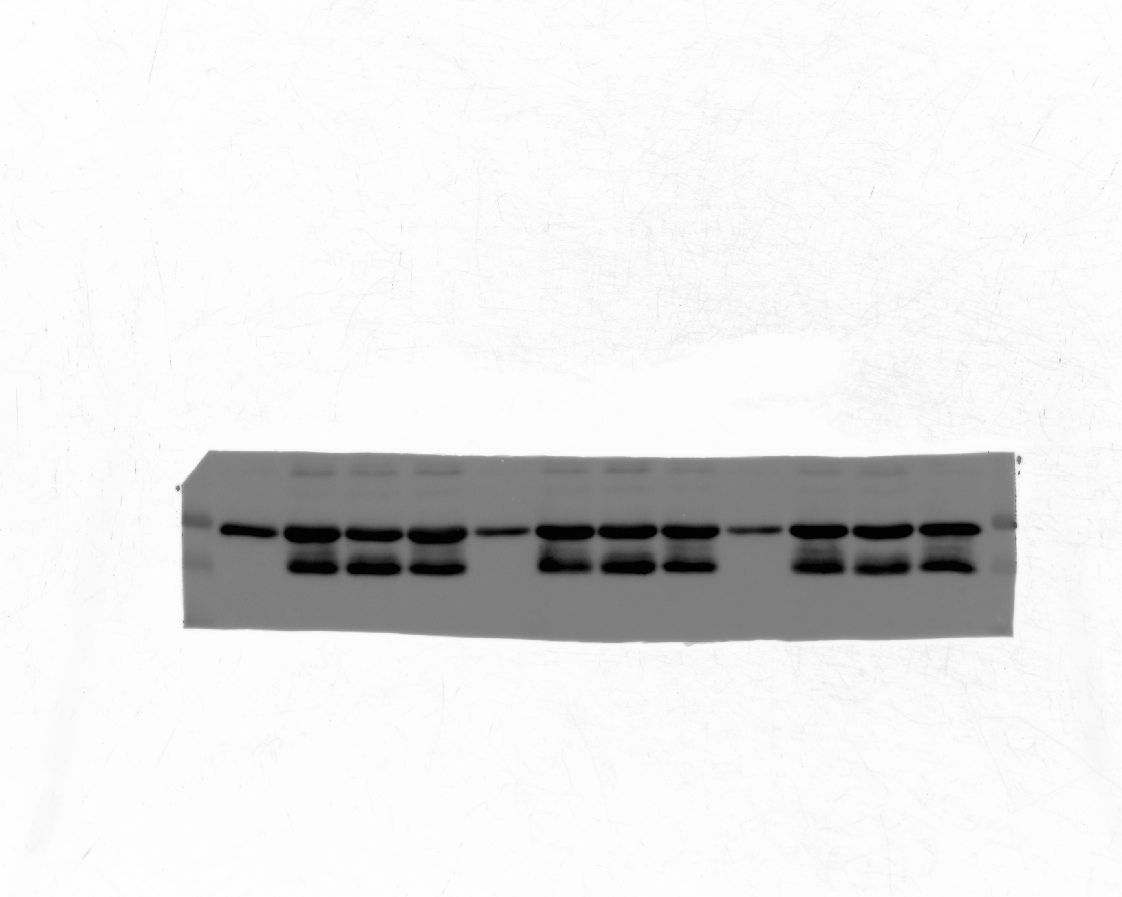

Supplement: Supplementary file 7 — Source data Fig. 5 [file 44319_2026_803_MOESM7_ESM.zip › Figure 5/5H/western Flag (IP).tif]

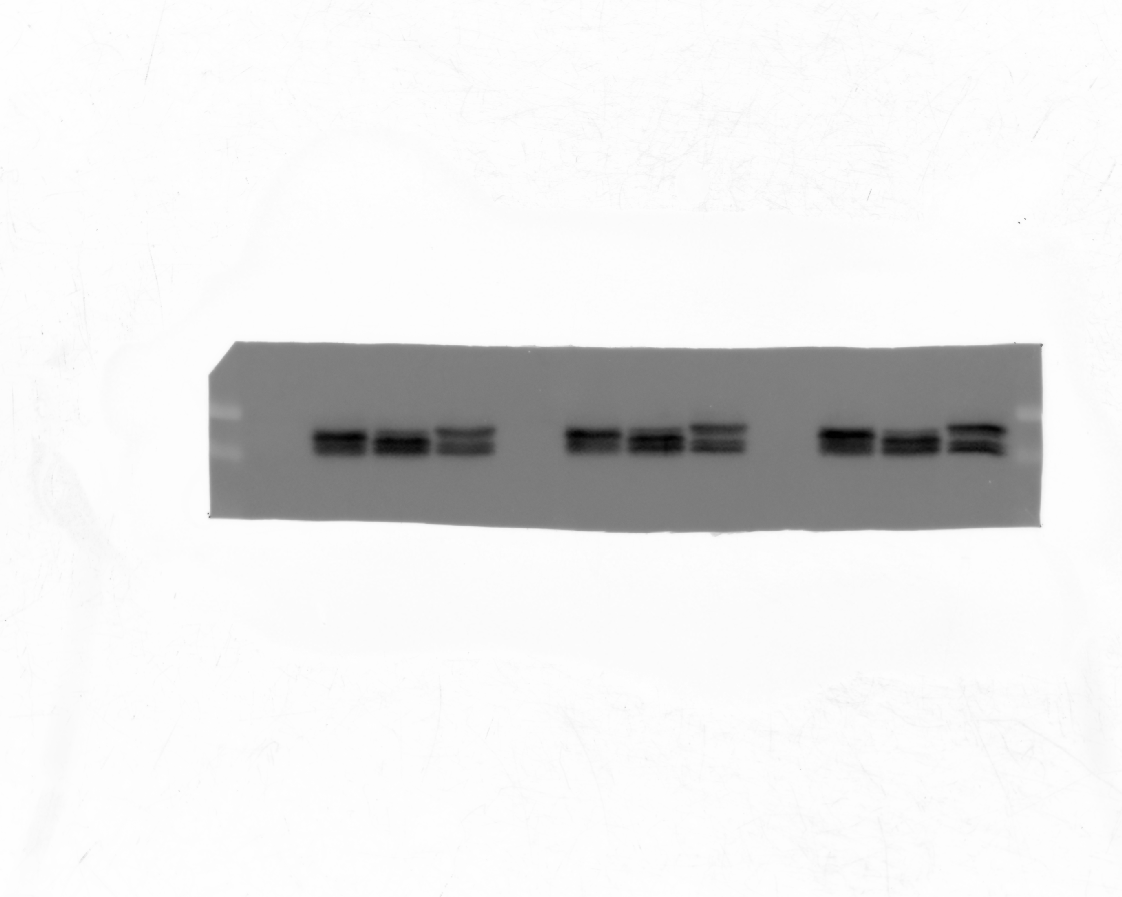

Supplement: Supplementary file 7 — Source data Fig. 5 [file 44319_2026_803_MOESM7_ESM.zip › Figure 5/5H/western p-4EBP1-S65 (IP).tif]

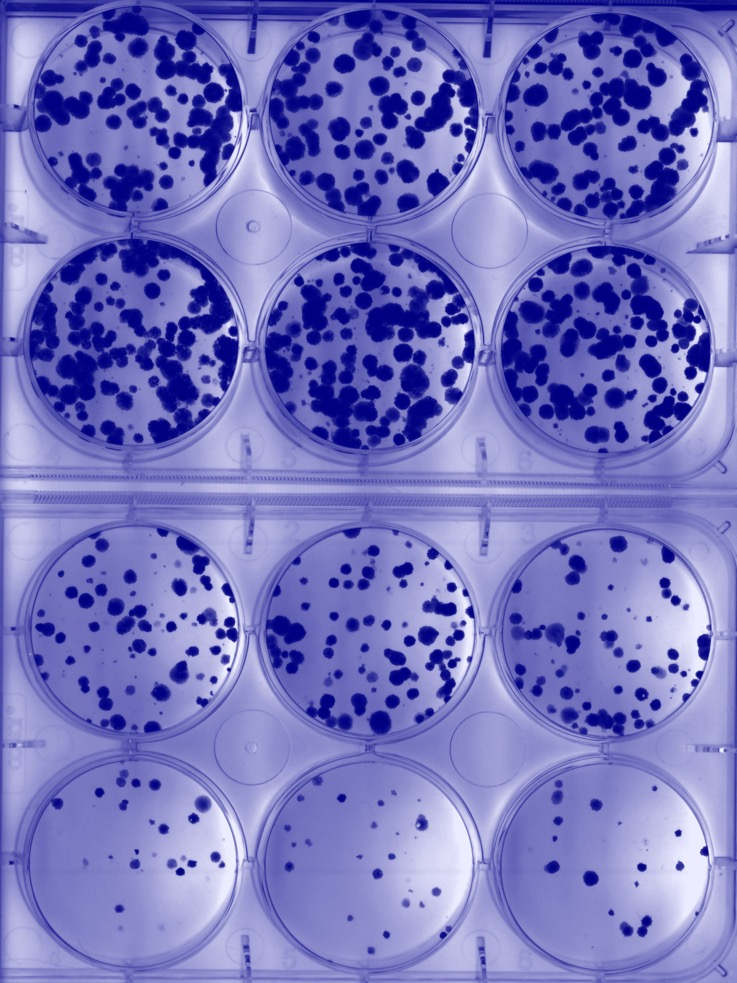

Supplement: Supplementary file 8 — Source data Fig. 6 [file 44319_2026_803_MOESM8_ESM.zip › Figure 6/6A/clonogenic survival assay.tif]

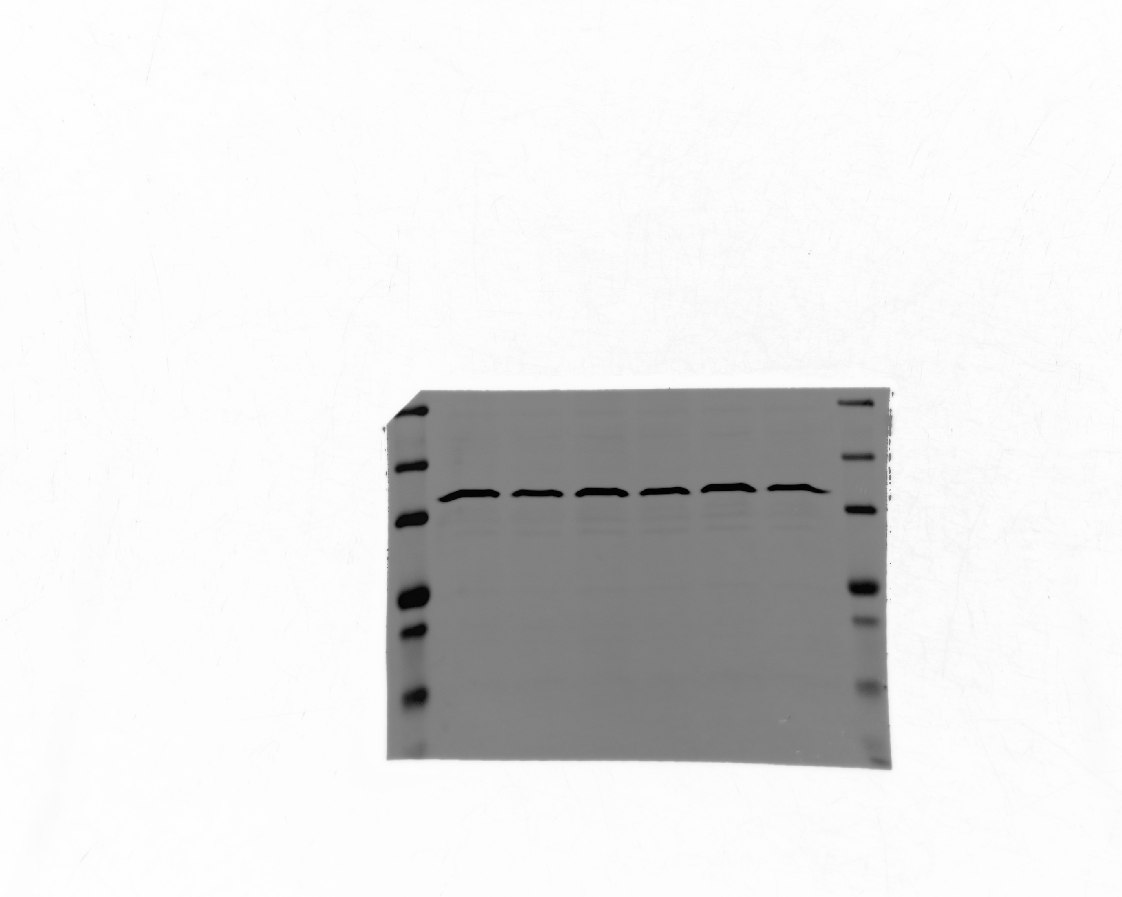

Supplement: Supplementary file 9 — Appendix Figure Source Data [file 44319_2026_803_MOESM9_ESM.zip › Appendix/Appendix S1/western A549 eIF2a.tif]

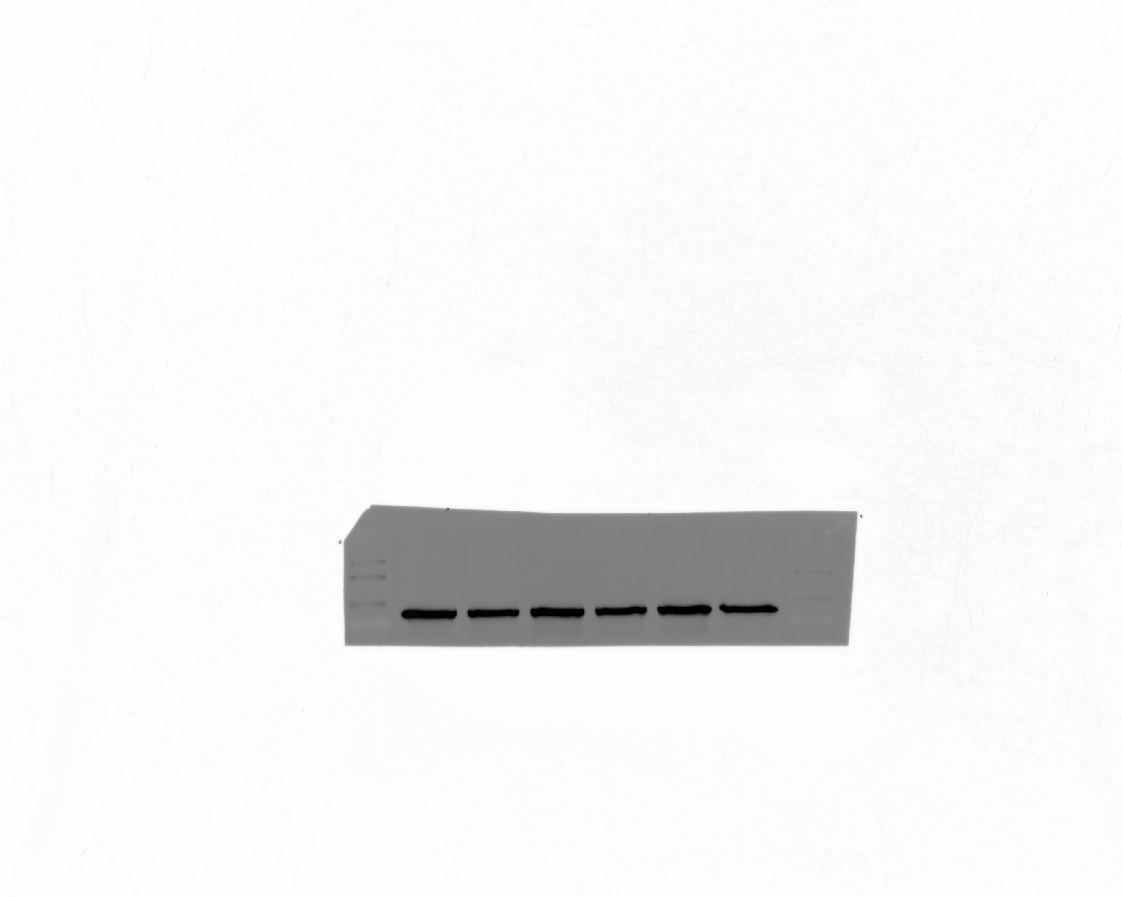

Supplement: Supplementary file 9 — Appendix Figure Source Data [file 44319_2026_803_MOESM9_ESM.zip › Appendix/Appendix S1/western A549 HSP90.tif]

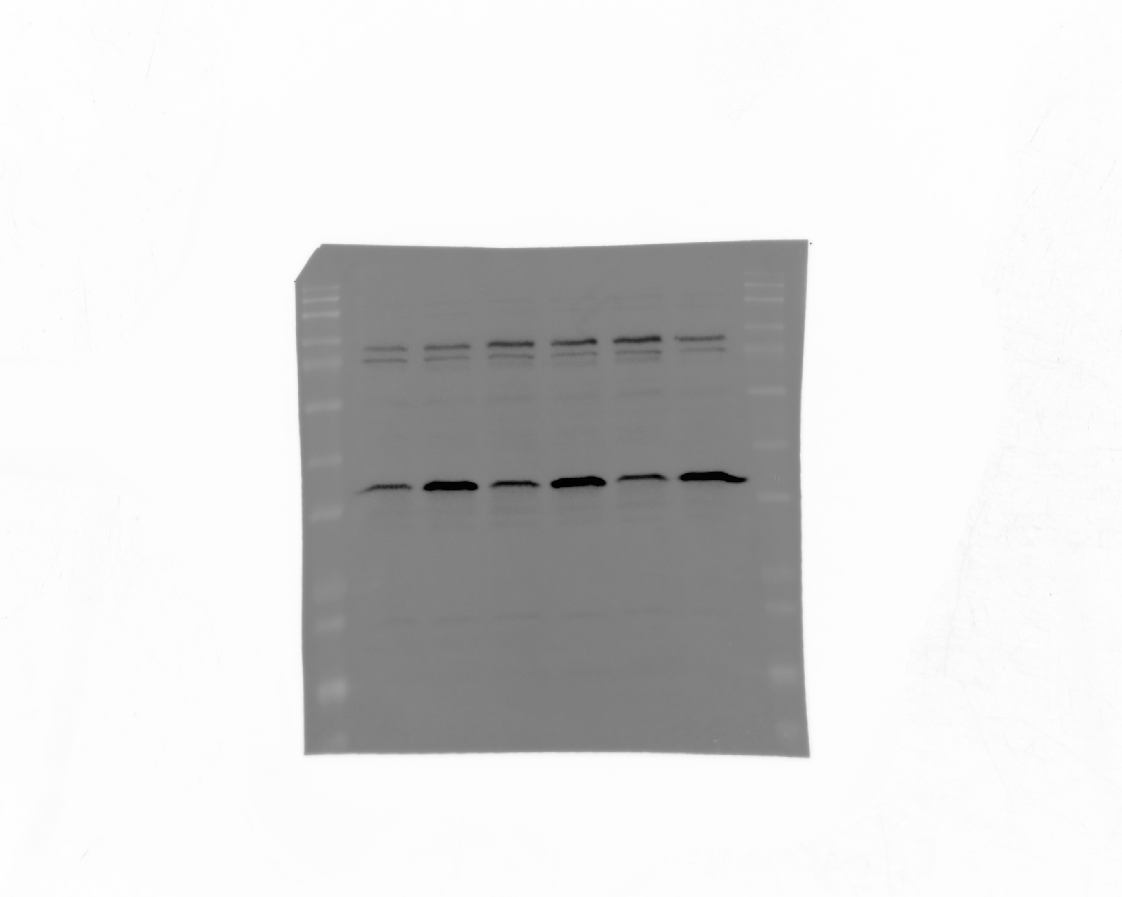

Supplement: Supplementary file 9 — Appendix Figure Source Data [file 44319_2026_803_MOESM9_ESM.zip › Appendix/Appendix S1/western A549 p-eIF2a.tif]

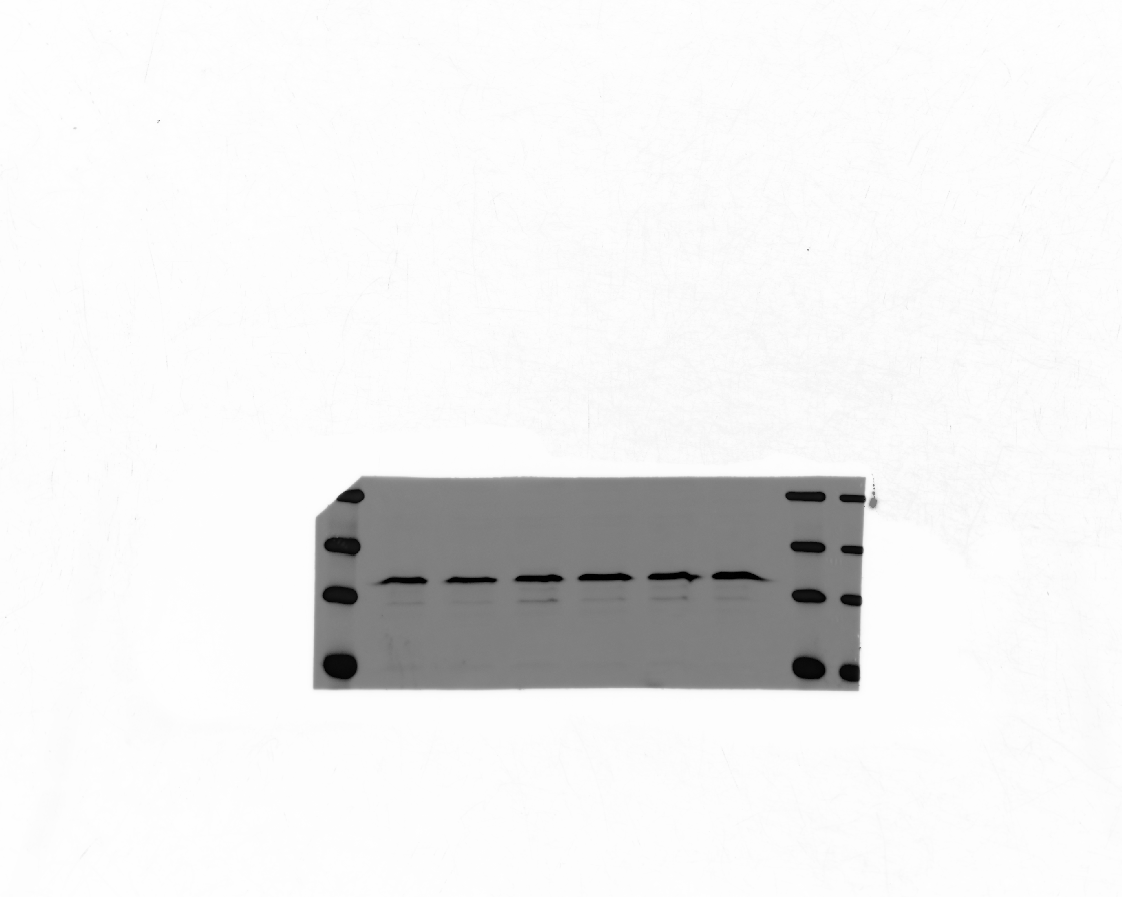

Supplement: Supplementary file 9 — Appendix Figure Source Data [file 44319_2026_803_MOESM9_ESM.zip › Appendix/Appendix S1/western HCT116 eIF2a.tif]

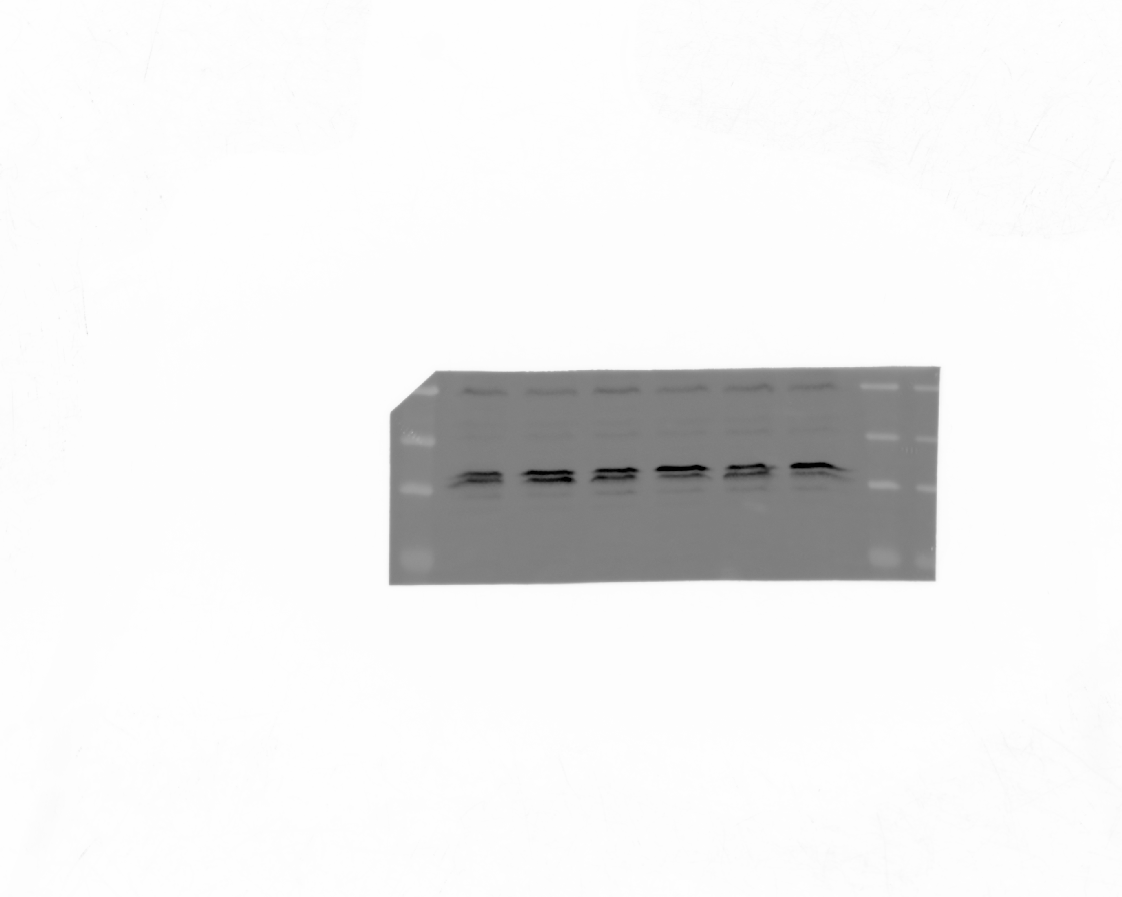

Supplement: Supplementary file 9 — Appendix Figure Source Data [file 44319_2026_803_MOESM9_ESM.zip › Appendix/Appendix S1/western HCT116 p-eIF2a.tif]

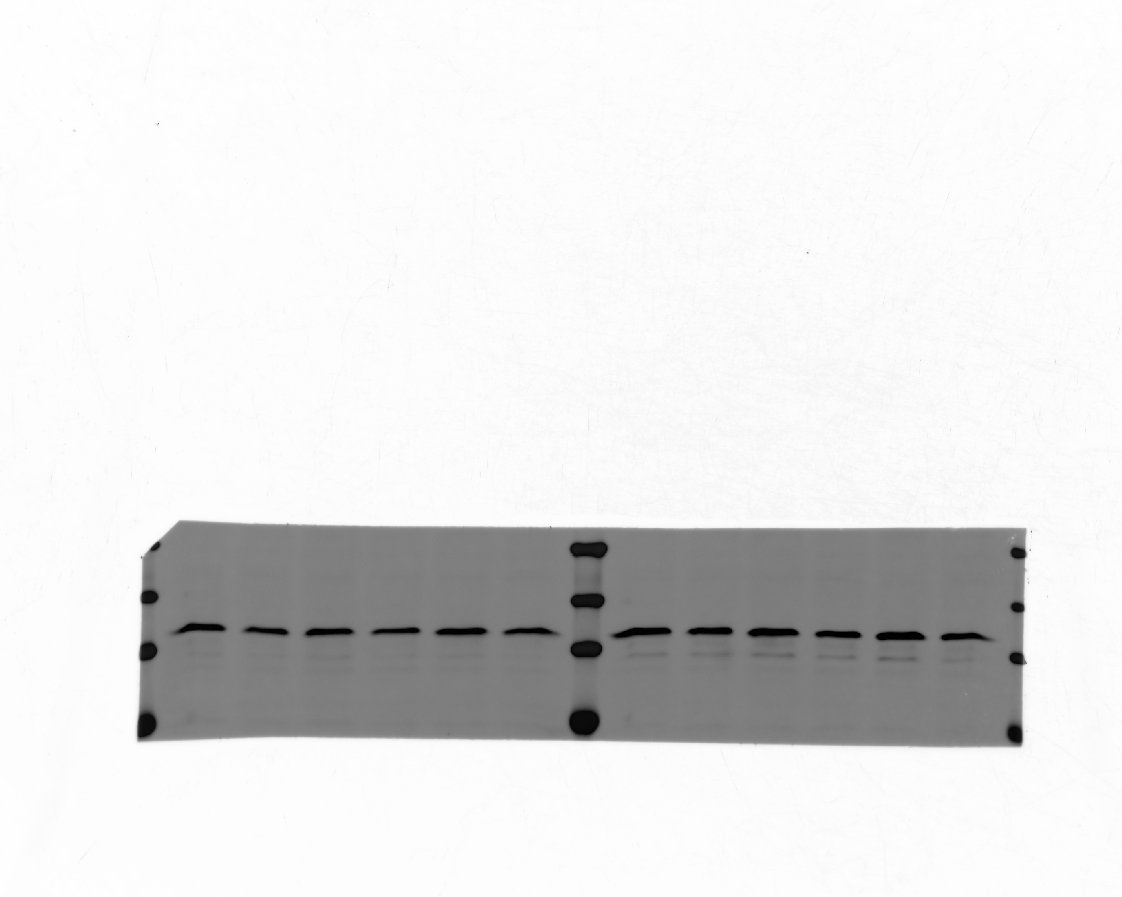

Supplement: Supplementary file 9 — Appendix Figure Source Data [file 44319_2026_803_MOESM9_ESM.zip › Appendix/Appendix S1/western HeLa-MCF7 eIF2a.tif]

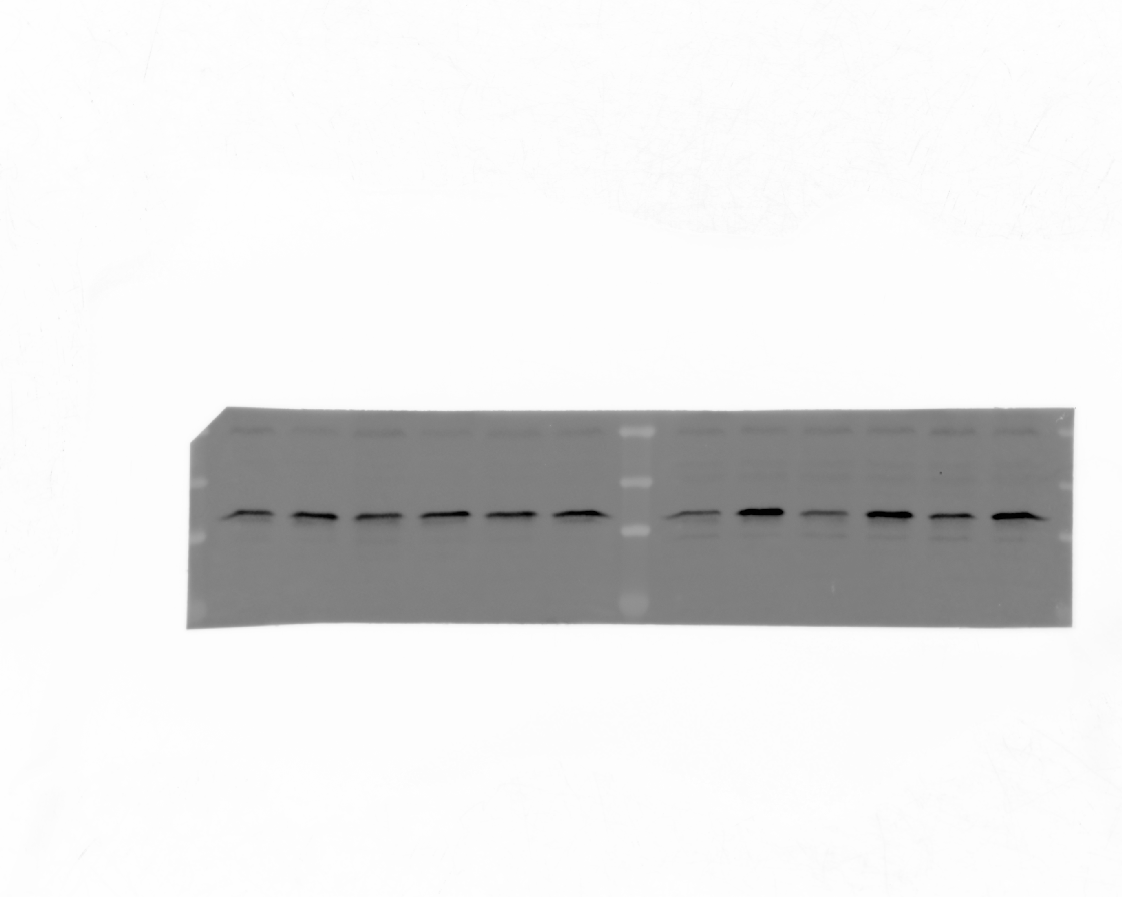

Supplement: Supplementary file 9 — Appendix Figure Source Data [file 44319_2026_803_MOESM9_ESM.zip › Appendix/Appendix S1/western HeLa-MCF7 p-eIF2a.tif]

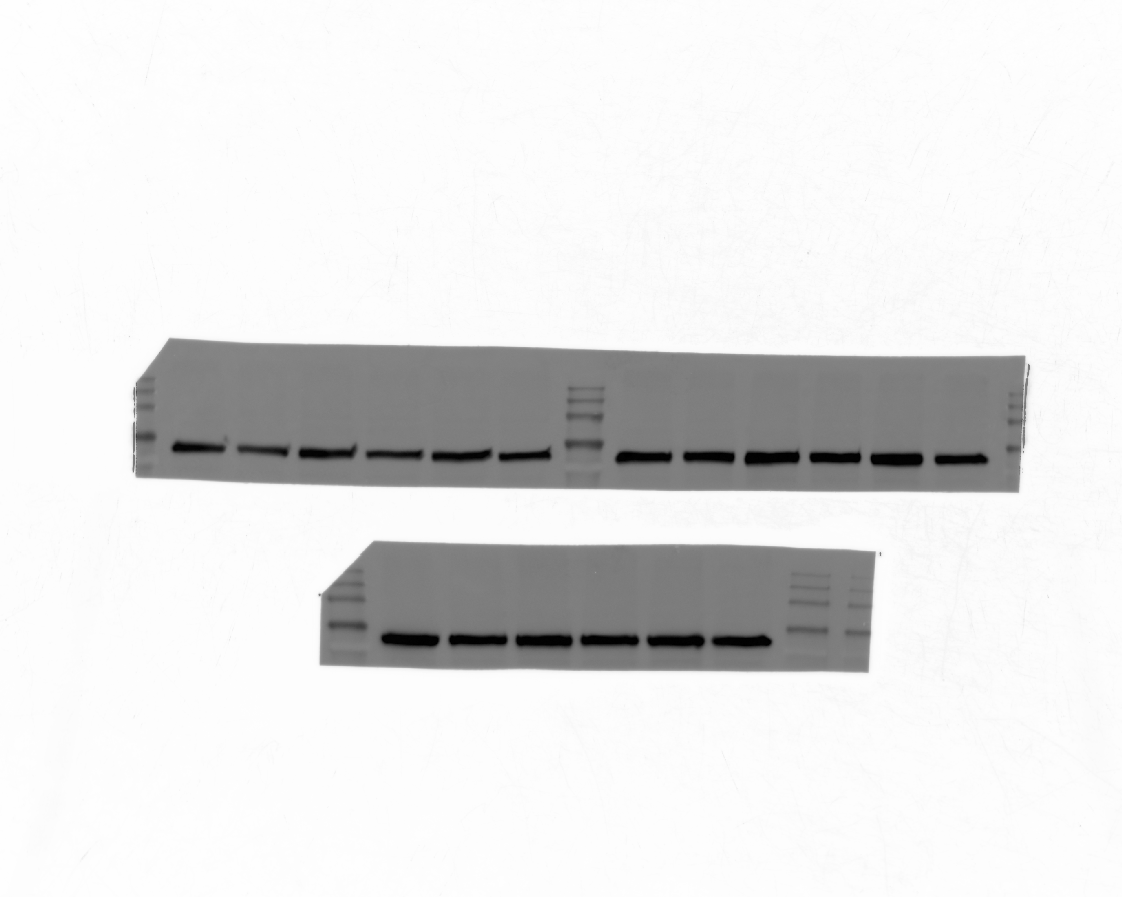

Supplement: Supplementary file 9 — Appendix Figure Source Data [file 44319_2026_803_MOESM9_ESM.zip › Appendix/Appendix S1/western HeLa-MCF7-HCT116 HSP90.tif]

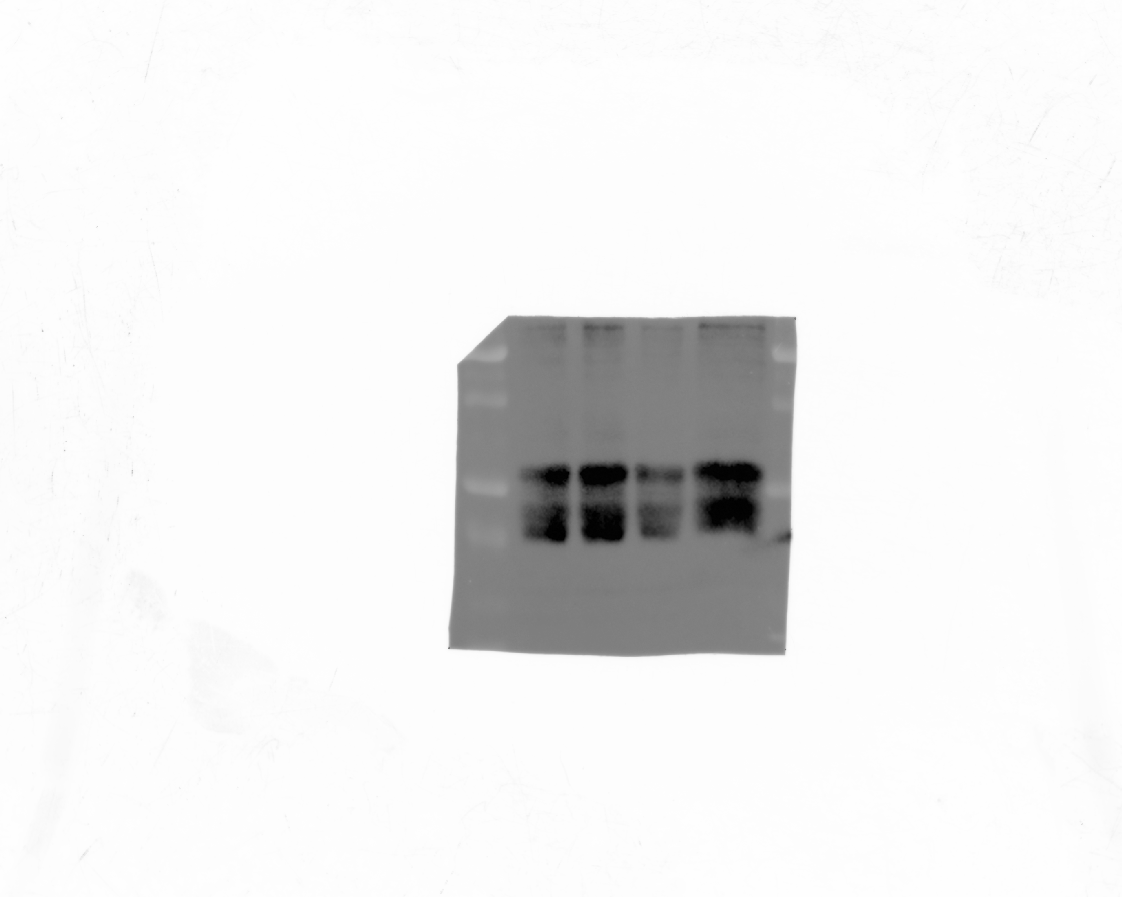

Supplement: Supplementary file 9 — Appendix Figure Source Data [file 44319_2026_803_MOESM9_ESM.zip › Appendix/Appendix S10/western AcIP Flag.tif]

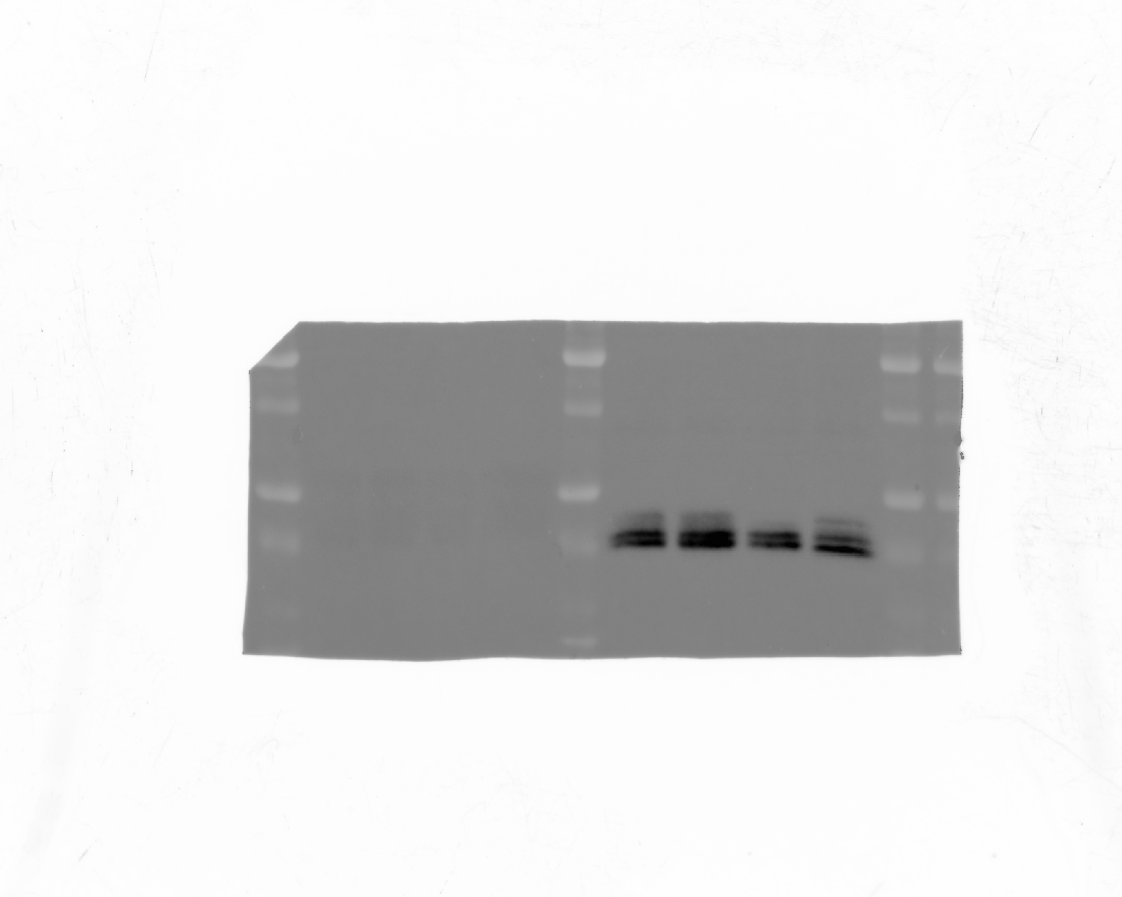

Supplement: Supplementary file 9 — Appendix Figure Source Data [file 44319_2026_803_MOESM9_ESM.zip › Appendix/Appendix S10/western input Flag.tif]

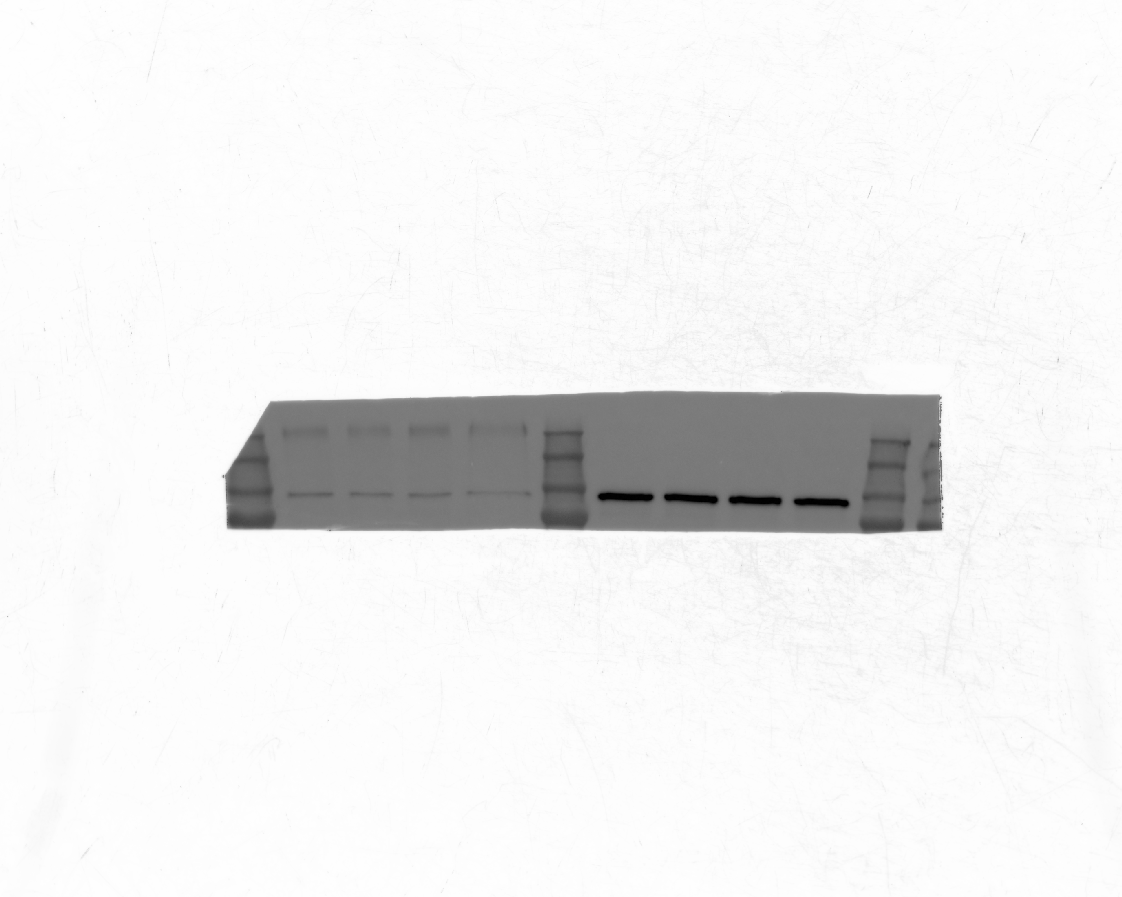

Supplement: Supplementary file 9 — Appendix Figure Source Data [file 44319_2026_803_MOESM9_ESM.zip › Appendix/Appendix S10/western input HSP90.tif]

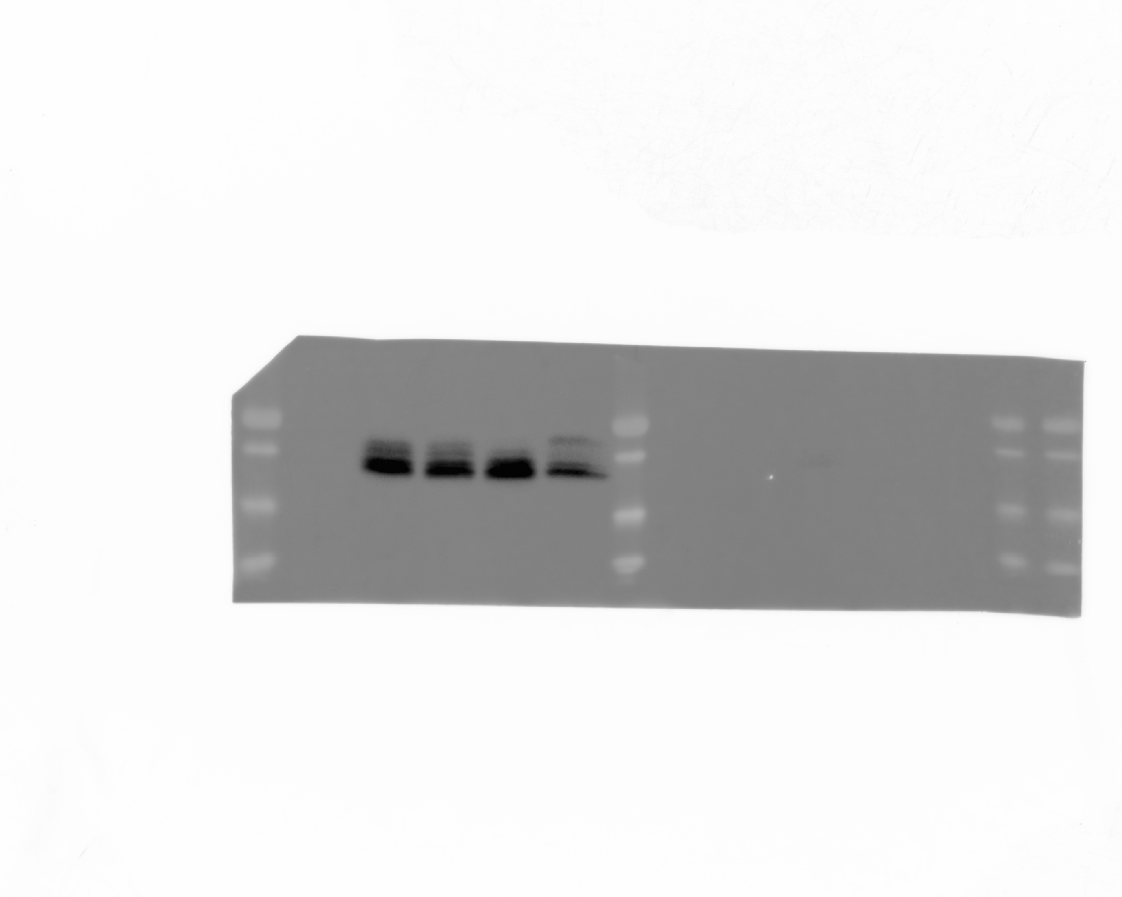

Supplement: Supplementary file 9 — Appendix Figure Source Data [file 44319_2026_803_MOESM9_ESM.zip › Appendix/Appendix S11/western input Flag.tif]

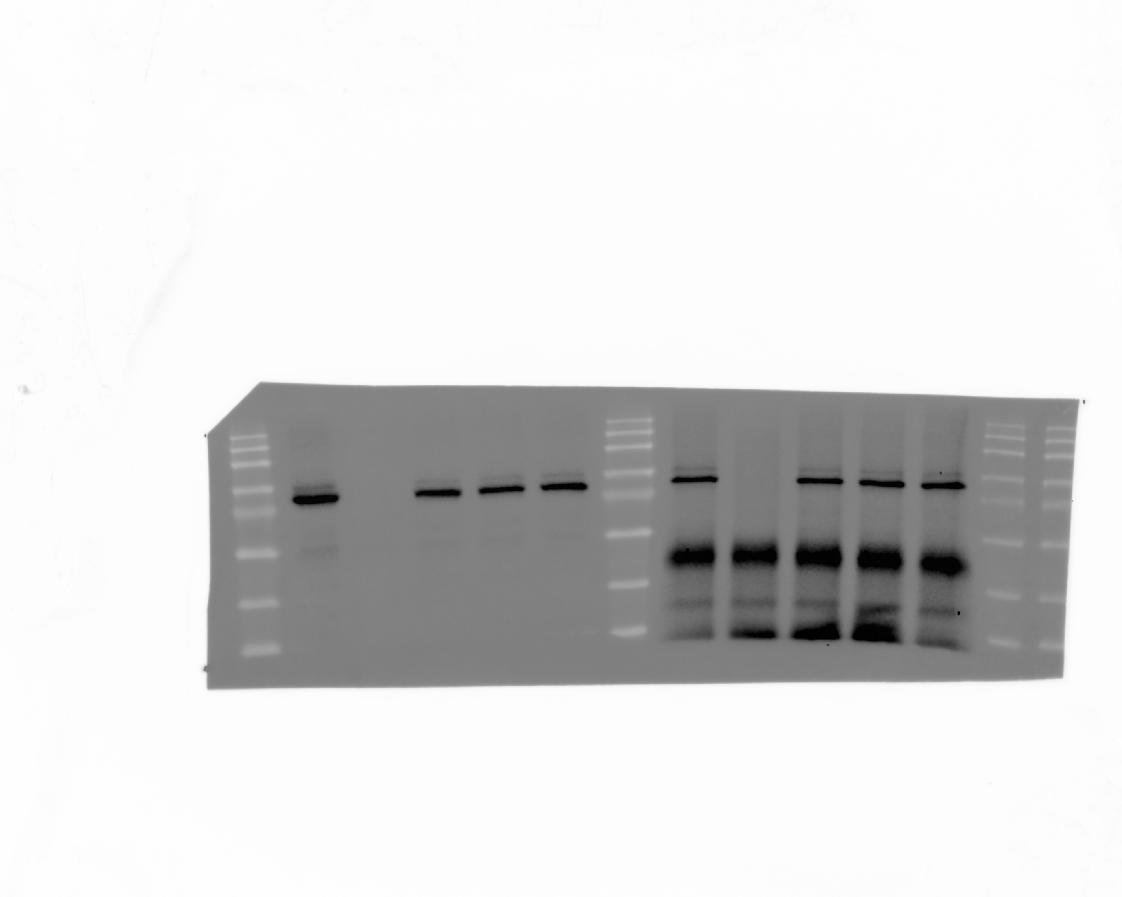

Supplement: Supplementary file 9 — Appendix Figure Source Data [file 44319_2026_803_MOESM9_ESM.zip › Appendix/Appendix S11/western input IP Myc.tif]

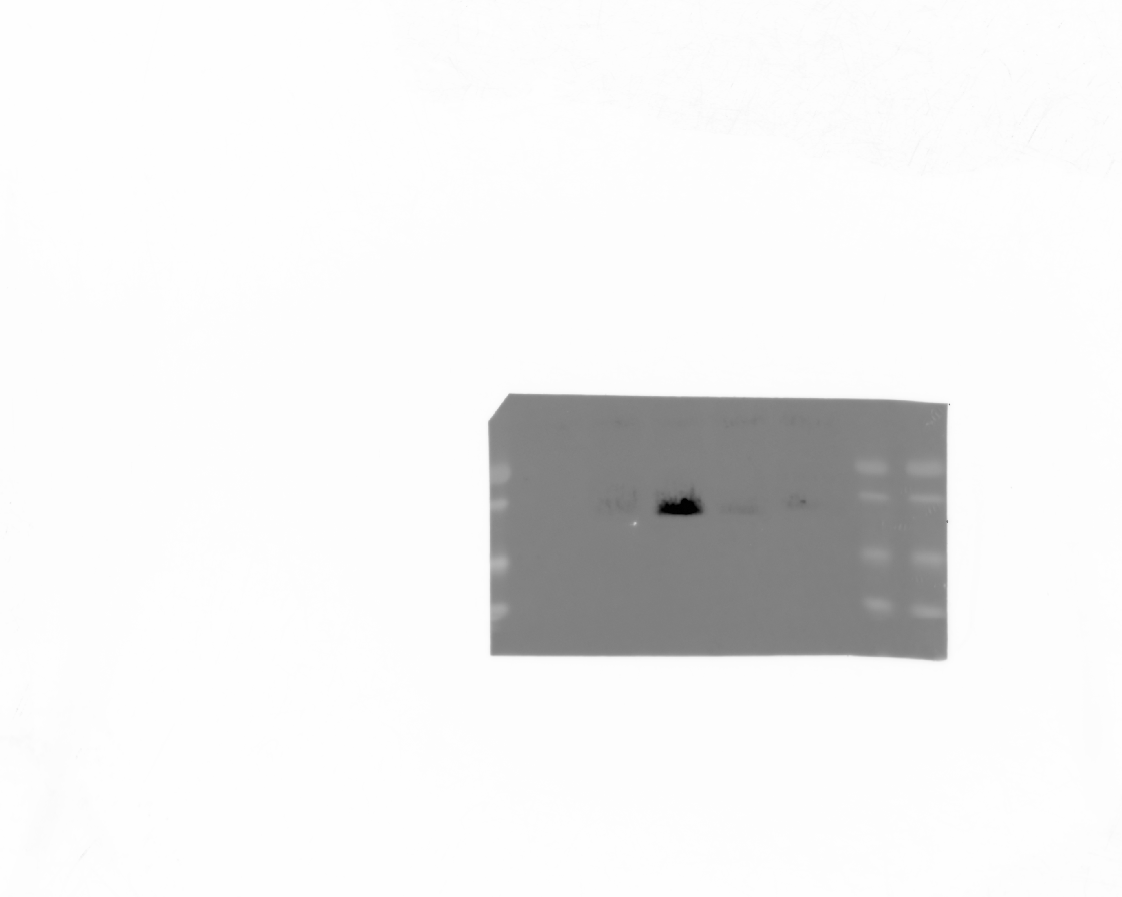

Supplement: Supplementary file 9 — Appendix Figure Source Data [file 44319_2026_803_MOESM9_ESM.zip › Appendix/Appendix S11/western IP Flag.tif]

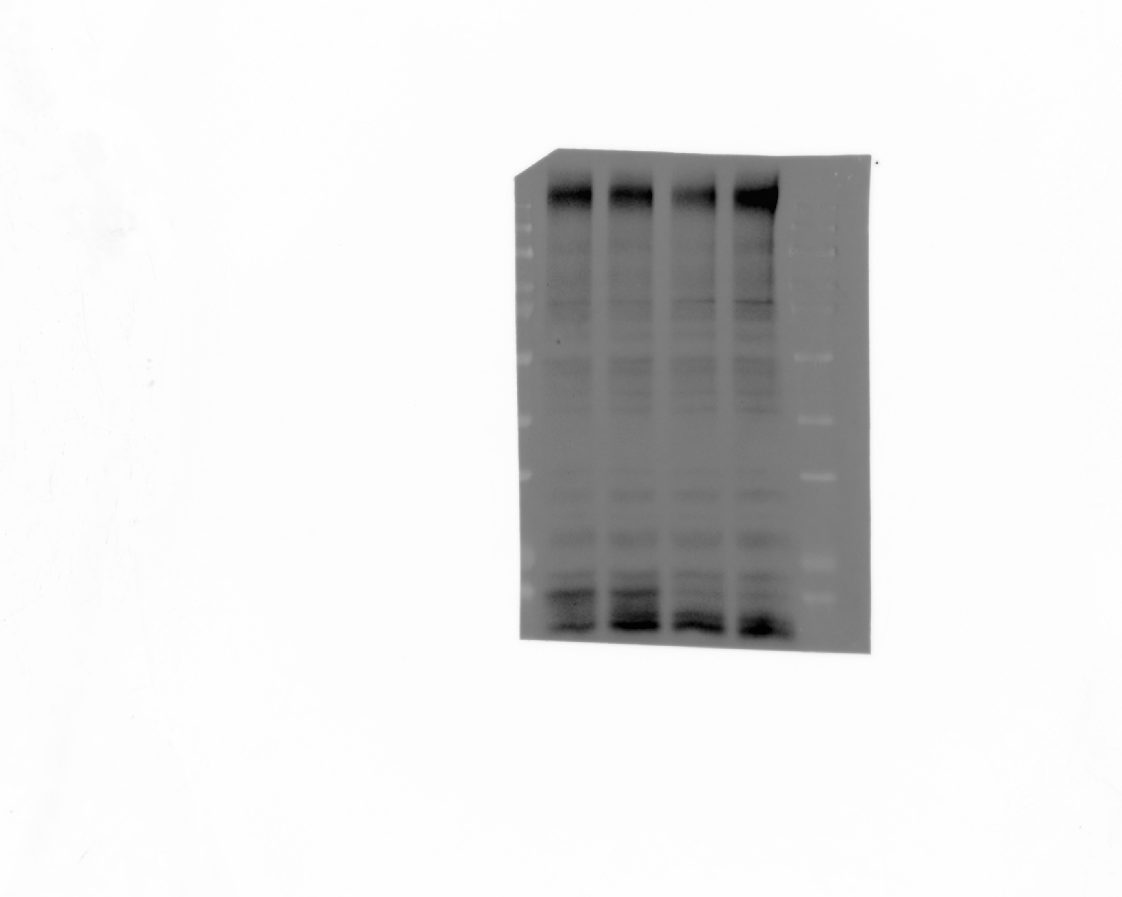

Supplement: Supplementary file 9 — Appendix Figure Source Data [file 44319_2026_803_MOESM9_ESM.zip › Appendix/Appendix S12/western AcIP 4E-BP1.tif]

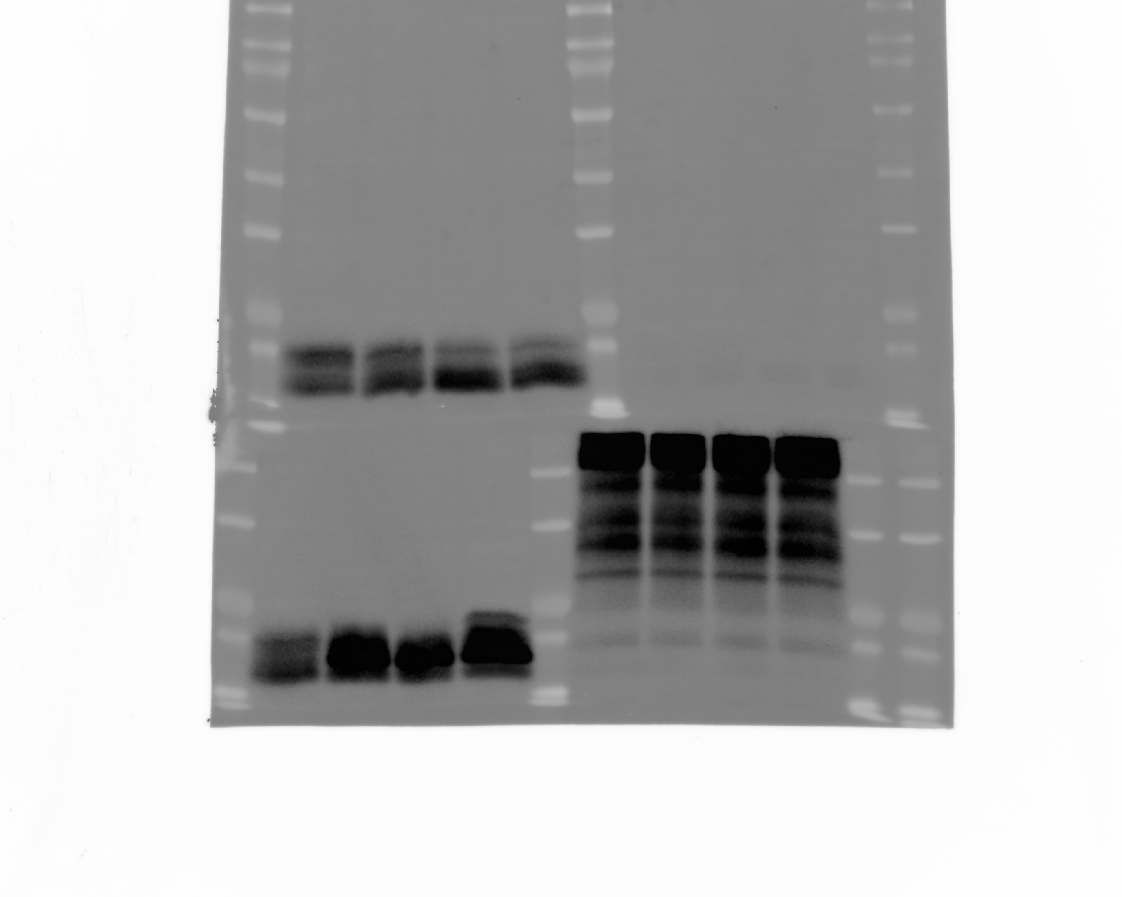

Supplement: Supplementary file 9 — Appendix Figure Source Data [file 44319_2026_803_MOESM9_ESM.zip › Appendix/Appendix S12/western input 4E-BP1.tif]

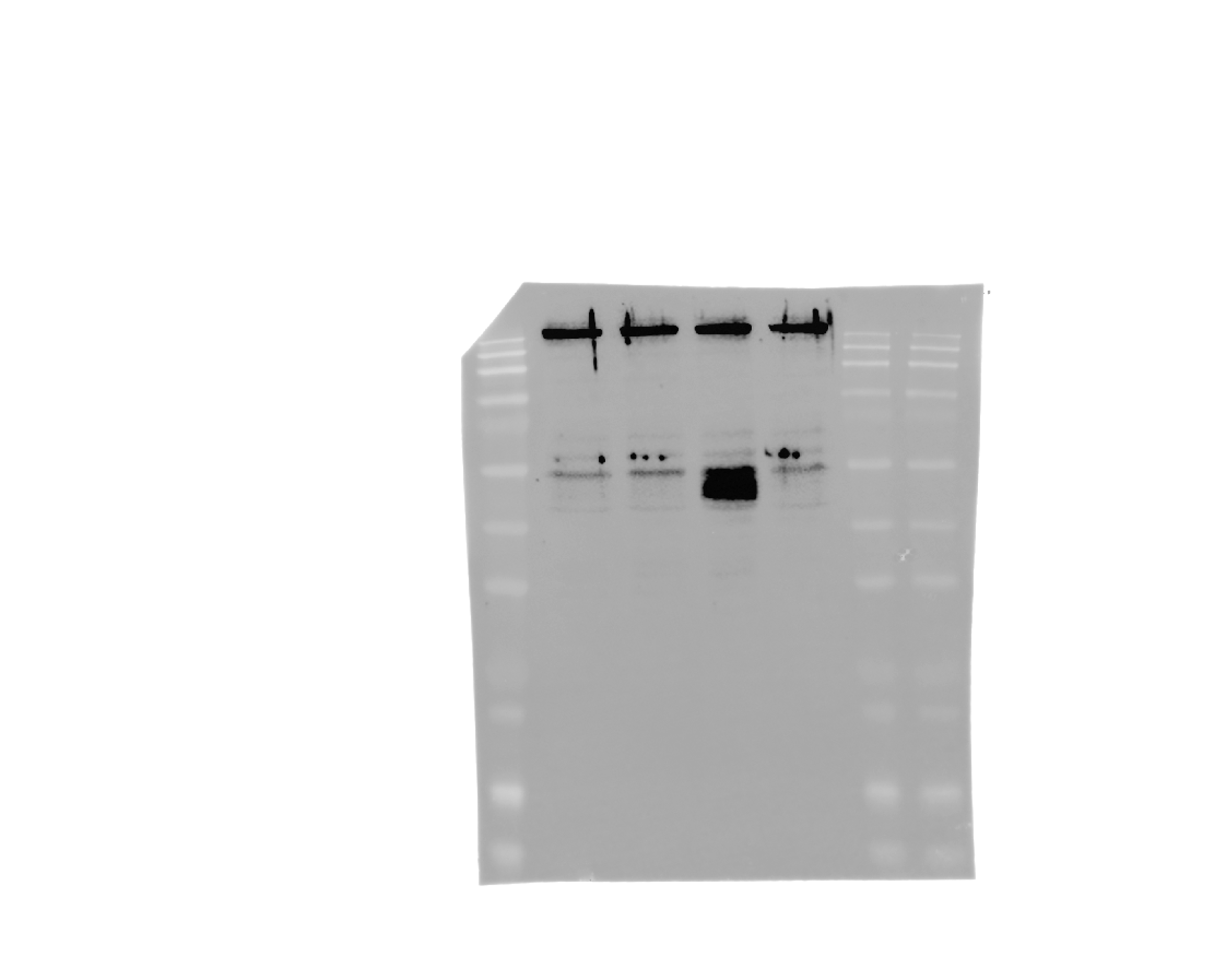

Supplement: Supplementary file 9 — Appendix Figure Source Data [file 44319_2026_803_MOESM9_ESM.zip › Appendix/Appendix S2/western ATF4.tif]

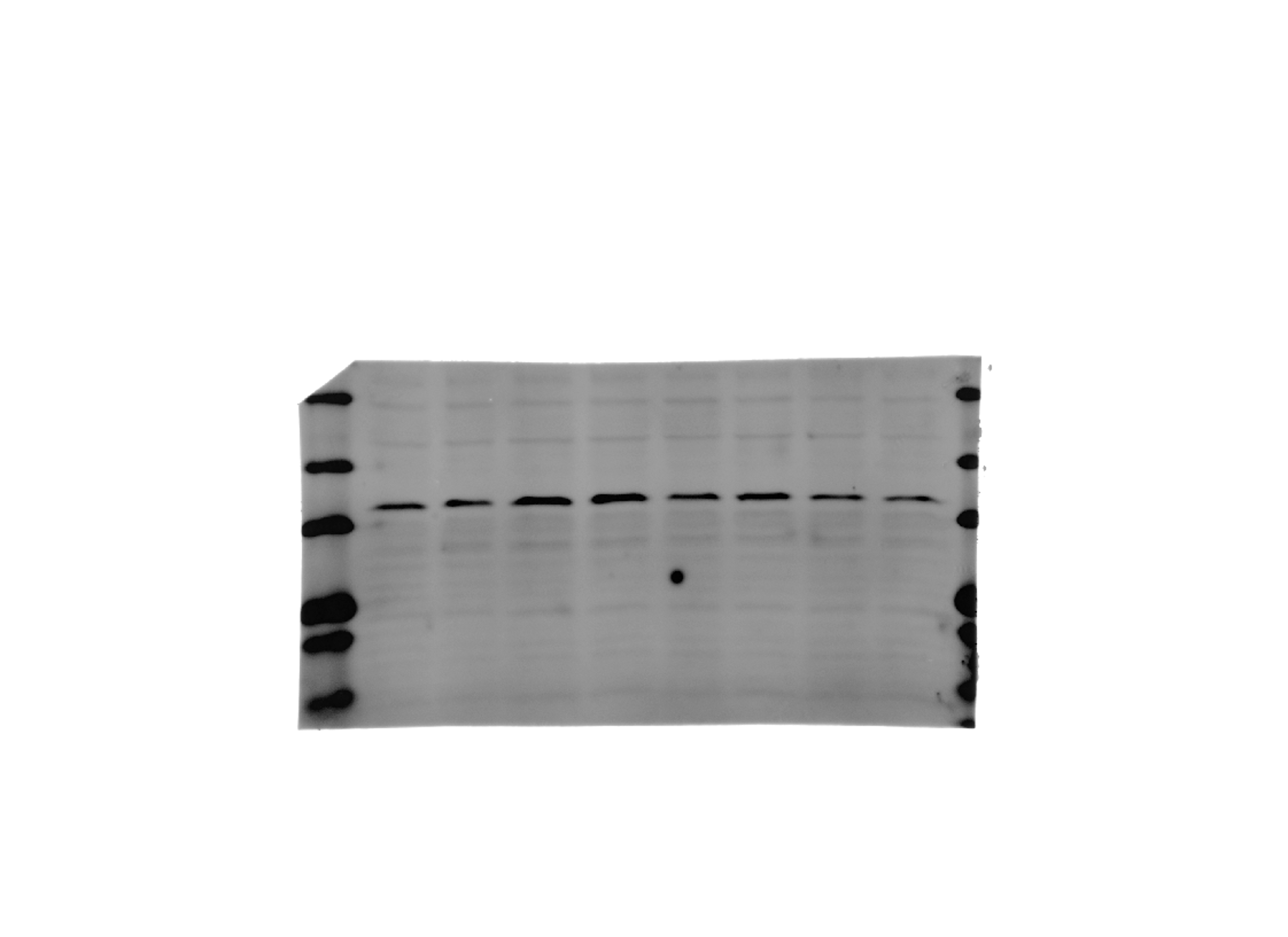

Supplement: Supplementary file 9 — Appendix Figure Source Data [file 44319_2026_803_MOESM9_ESM.zip › Appendix/Appendix S2/western eIF2a.tif]

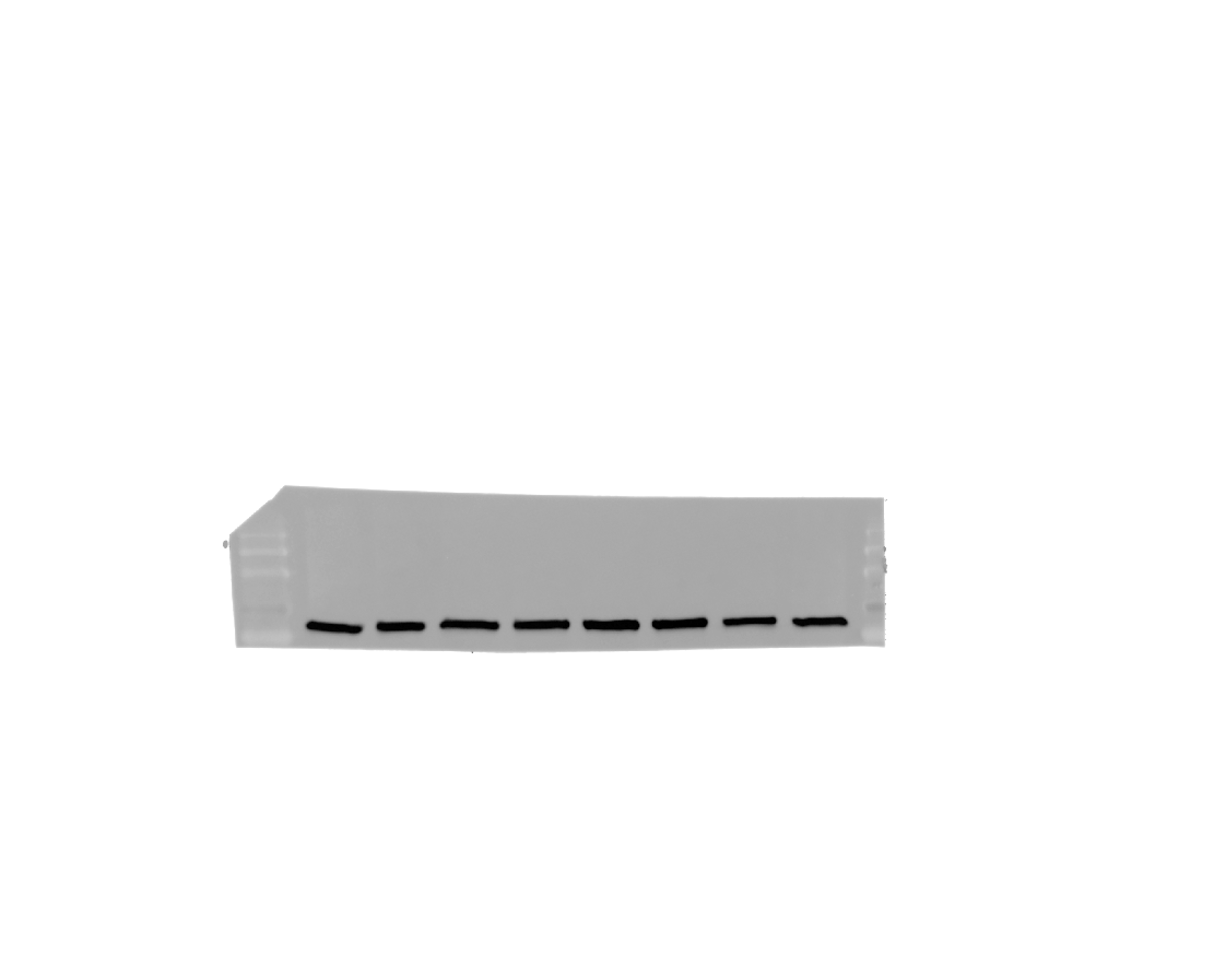

Supplement: Supplementary file 9 — Appendix Figure Source Data [file 44319_2026_803_MOESM9_ESM.zip › Appendix/Appendix S2/western HSP90.tif]

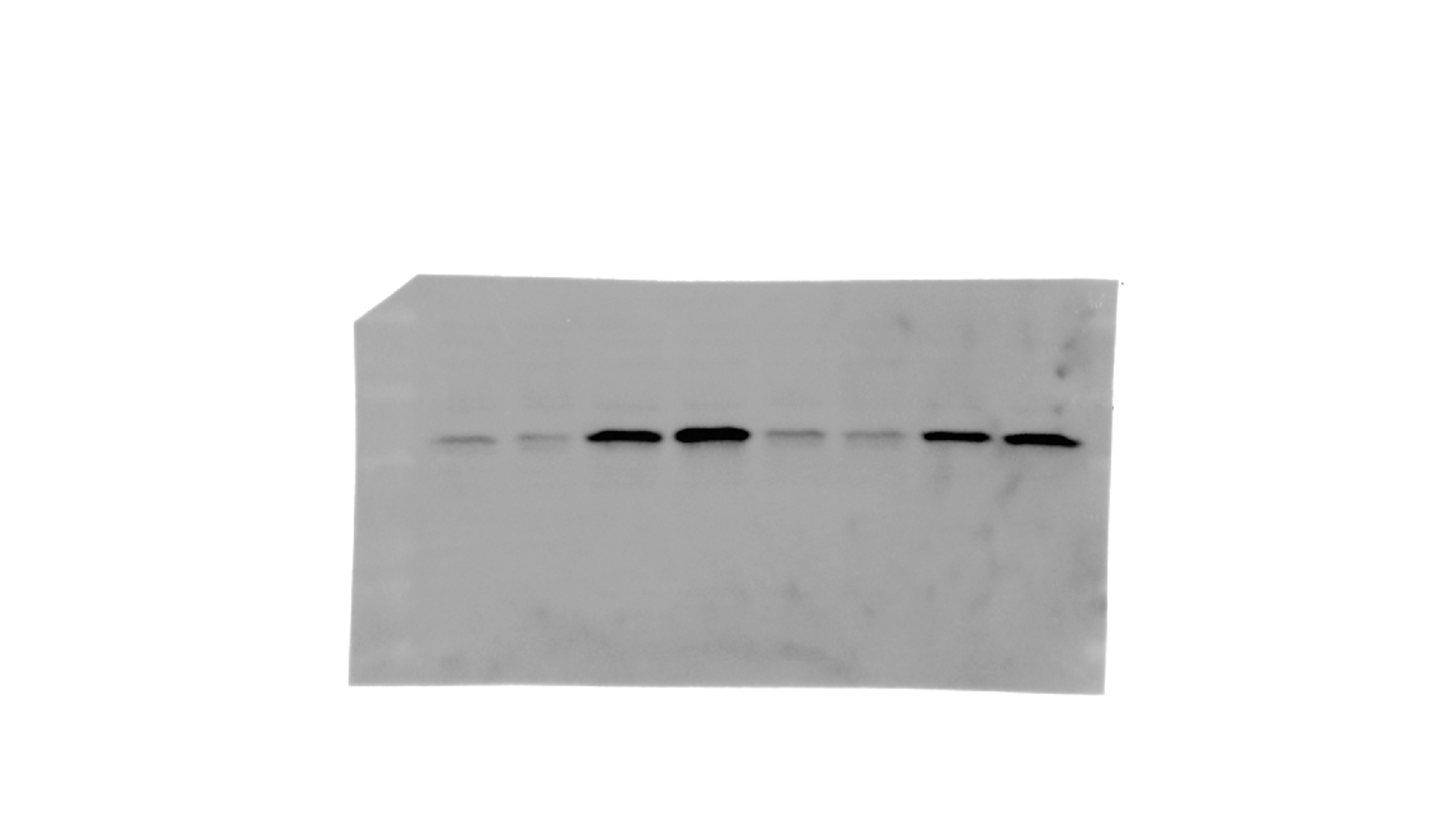

Supplement: Supplementary file 9 — Appendix Figure Source Data [file 44319_2026_803_MOESM9_ESM.zip › Appendix/Appendix S2/western p-eIF2a.tif]

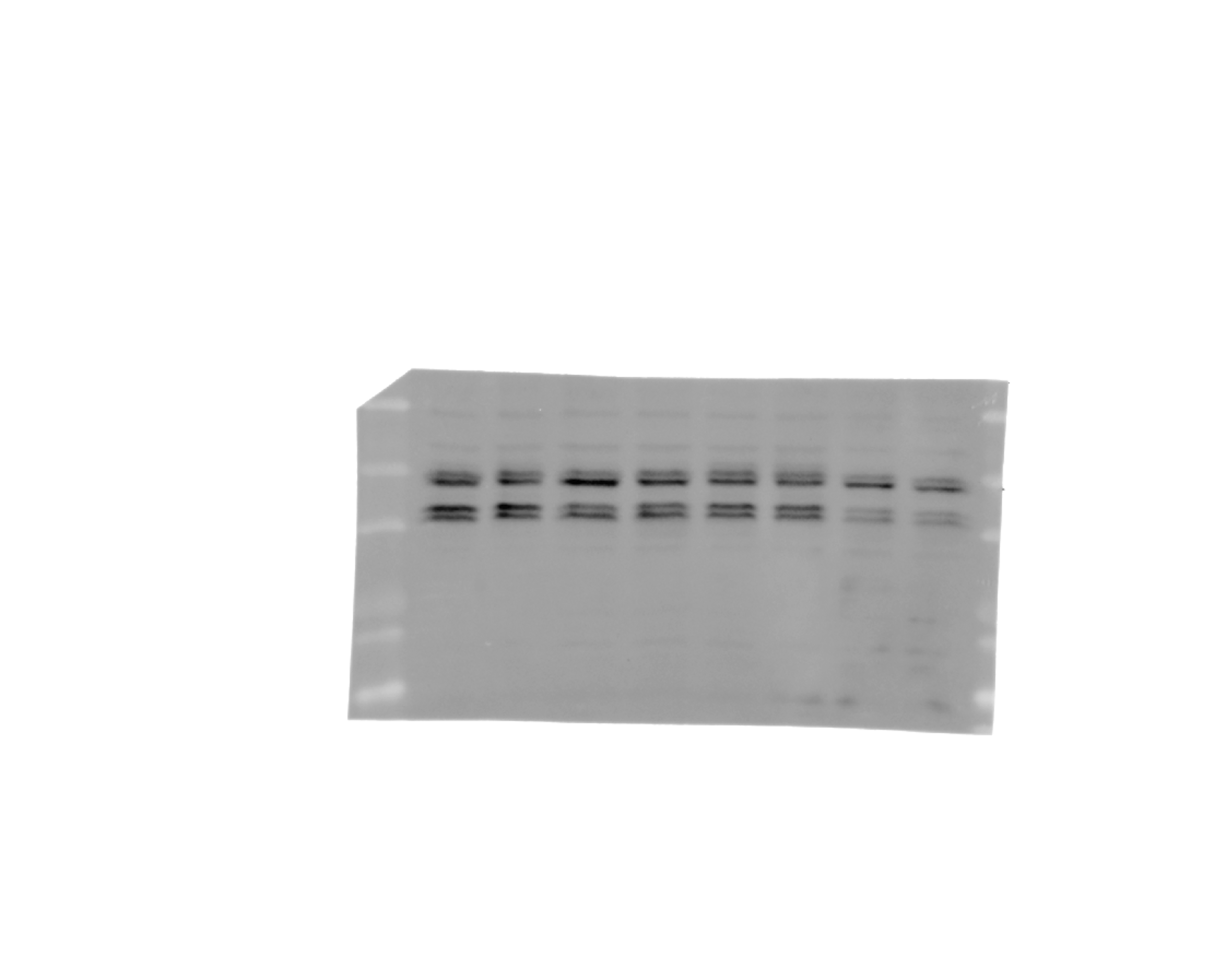

Supplement: Supplementary file 9 — Appendix Figure Source Data [file 44319_2026_803_MOESM9_ESM.zip › Appendix/Appendix S2/western SIRT2.tif]

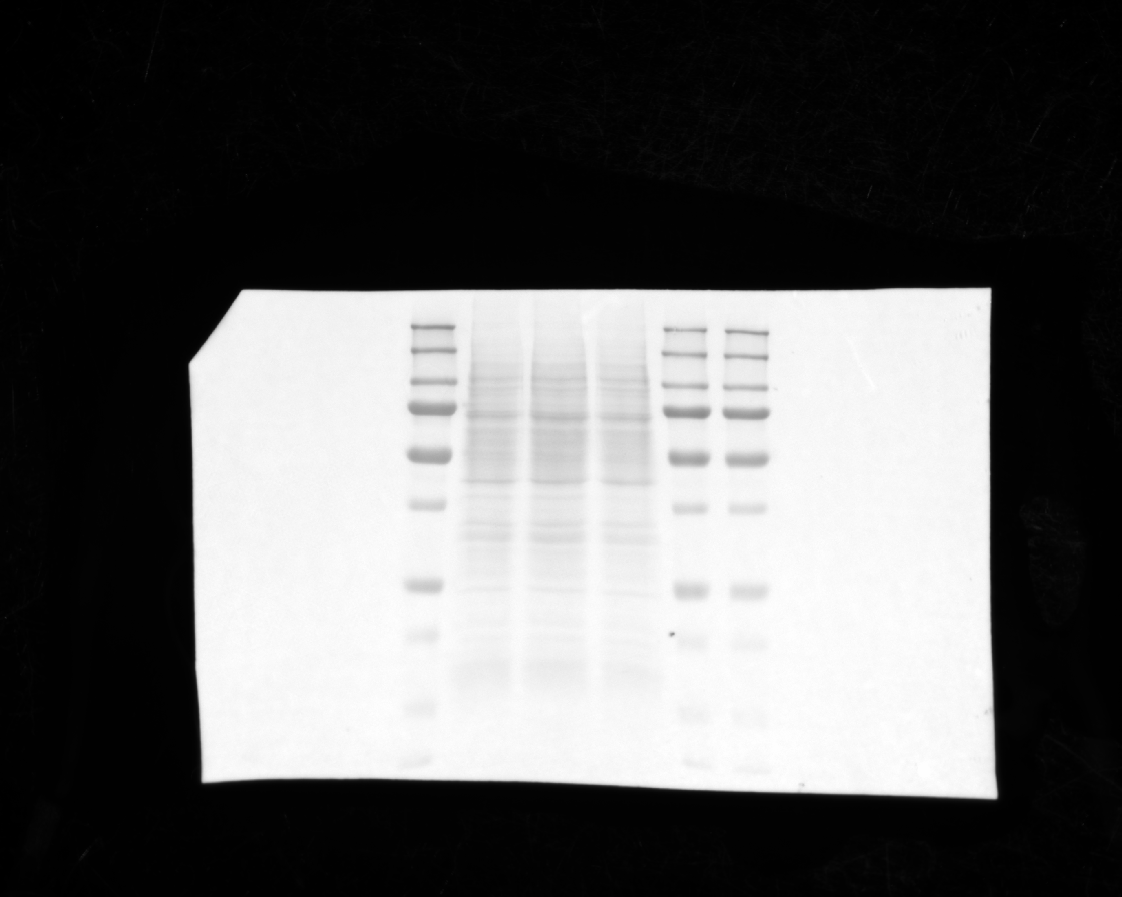

Supplement: Supplementary file 9 — Appendix Figure Source Data [file 44319_2026_803_MOESM9_ESM.zip › Appendix/Appendix S7/western Ponceau S.tif]

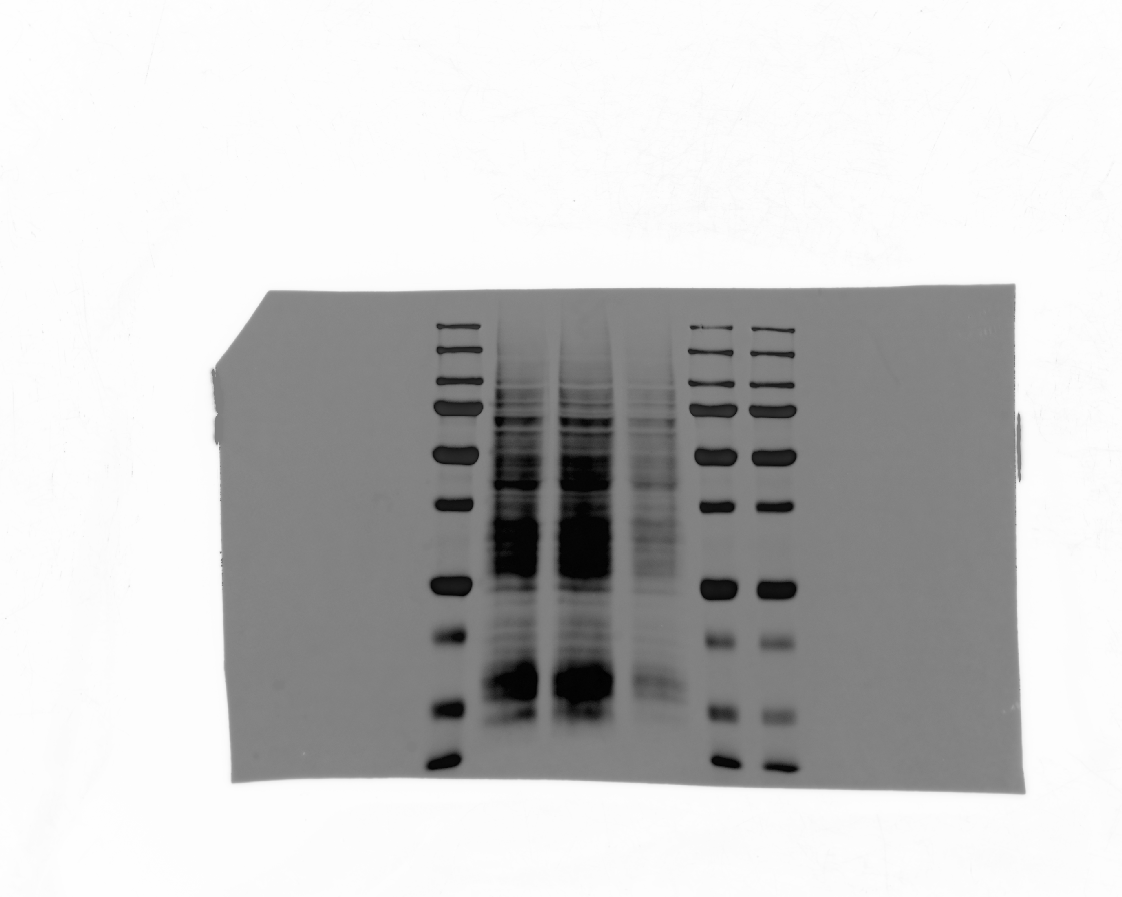

Supplement: Supplementary file 9 — Appendix Figure Source Data [file 44319_2026_803_MOESM9_ESM.zip › Appendix/Appendix S7/western Puromycin.tif]

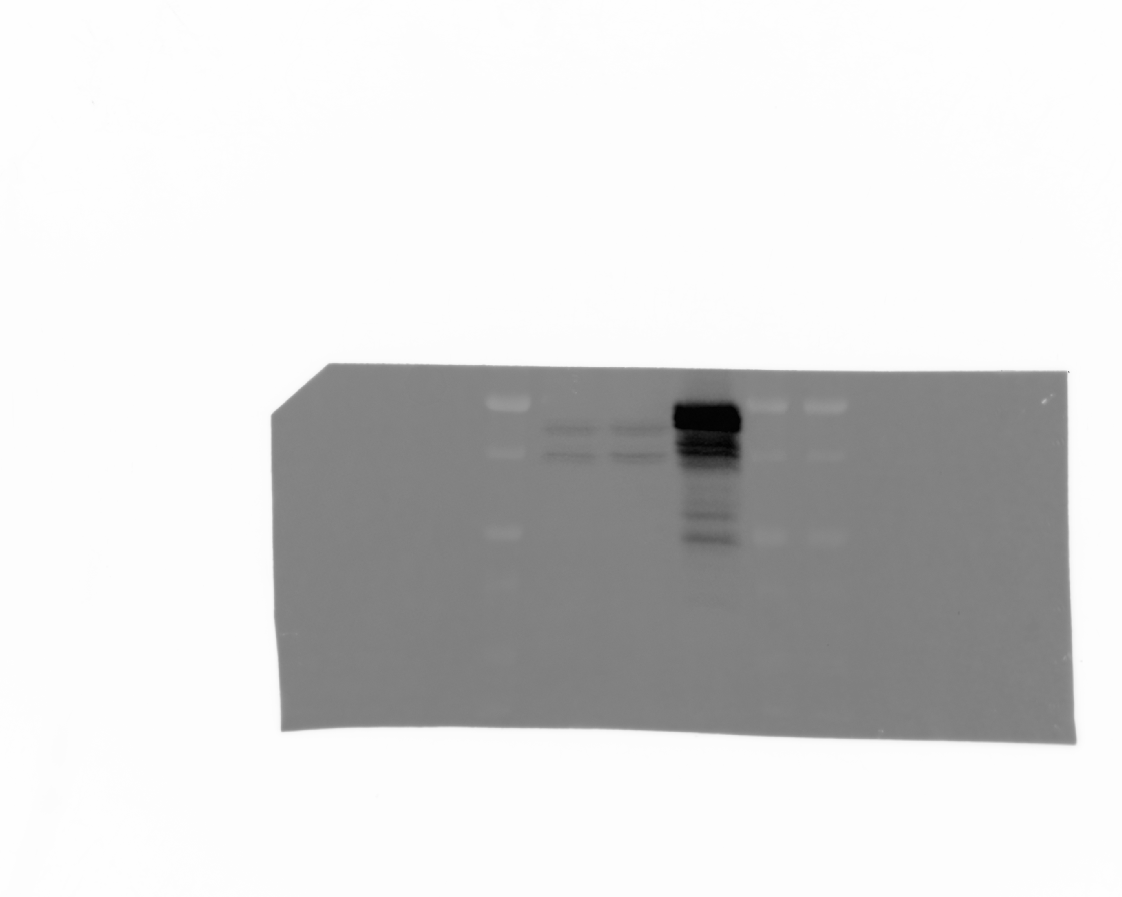

Supplement: Supplementary file 9 — Appendix Figure Source Data [file 44319_2026_803_MOESM9_ESM.zip › Appendix/Appendix S7/western SIRT2.tif]

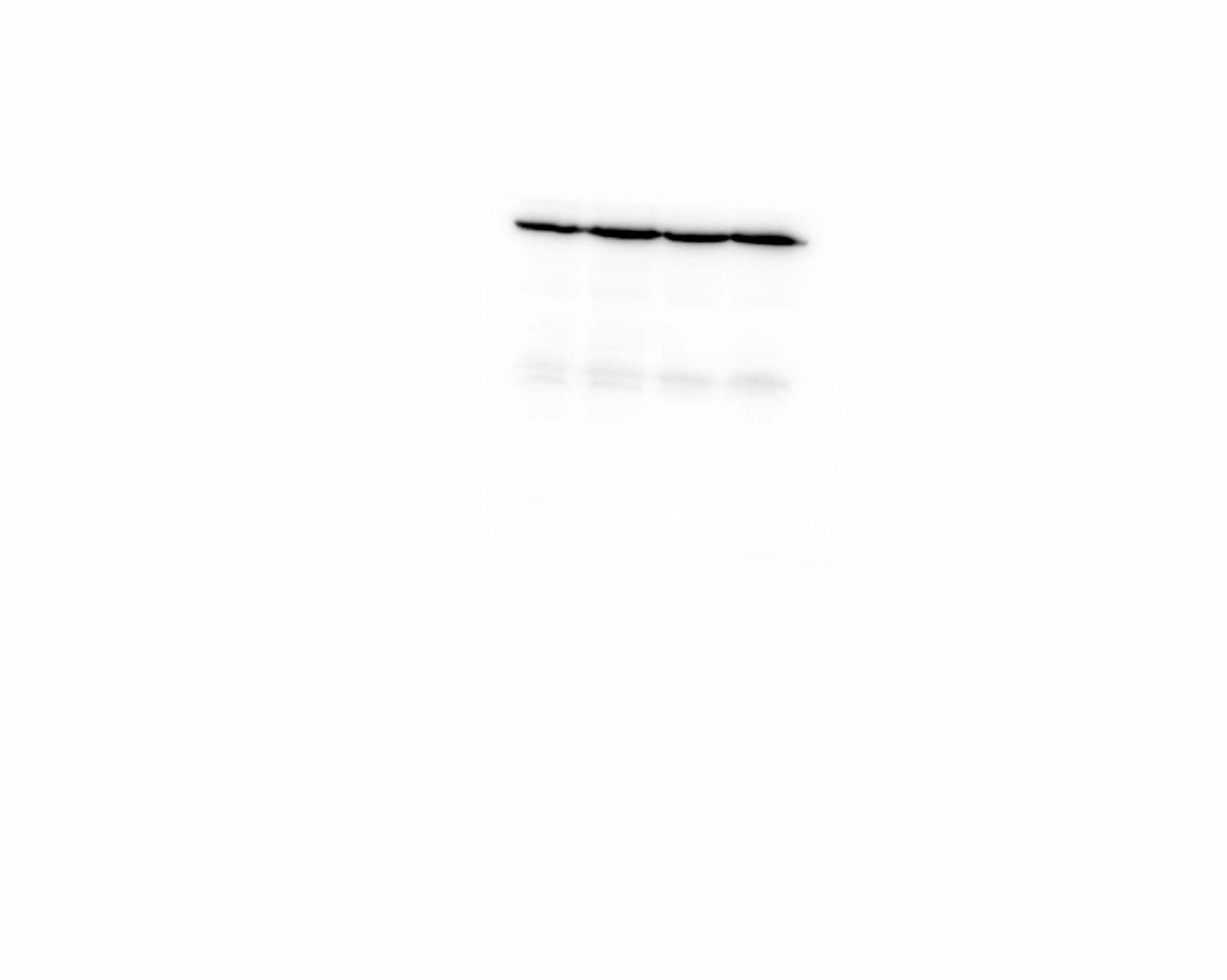

Supplement: Supplementary file 9 — Appendix Figure Source Data [file 44319_2026_803_MOESM9_ESM.zip › Appendix/Appendix S8/western Actin (for p-S6K_293T).tif]

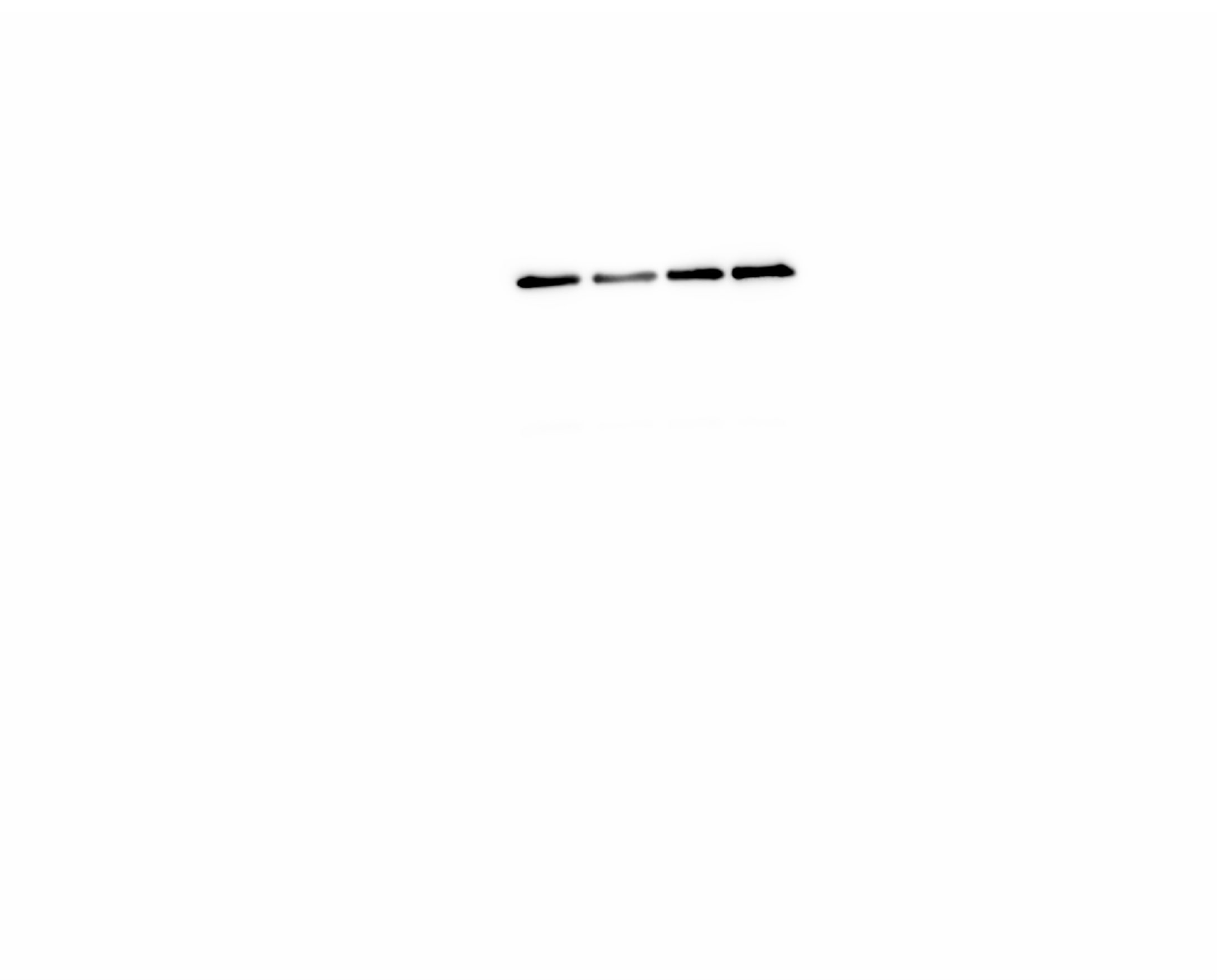

Supplement: Supplementary file 9 — Appendix Figure Source Data [file 44319_2026_803_MOESM9_ESM.zip › Appendix/Appendix S8/western Actin (for p-S6K_A549).tif]

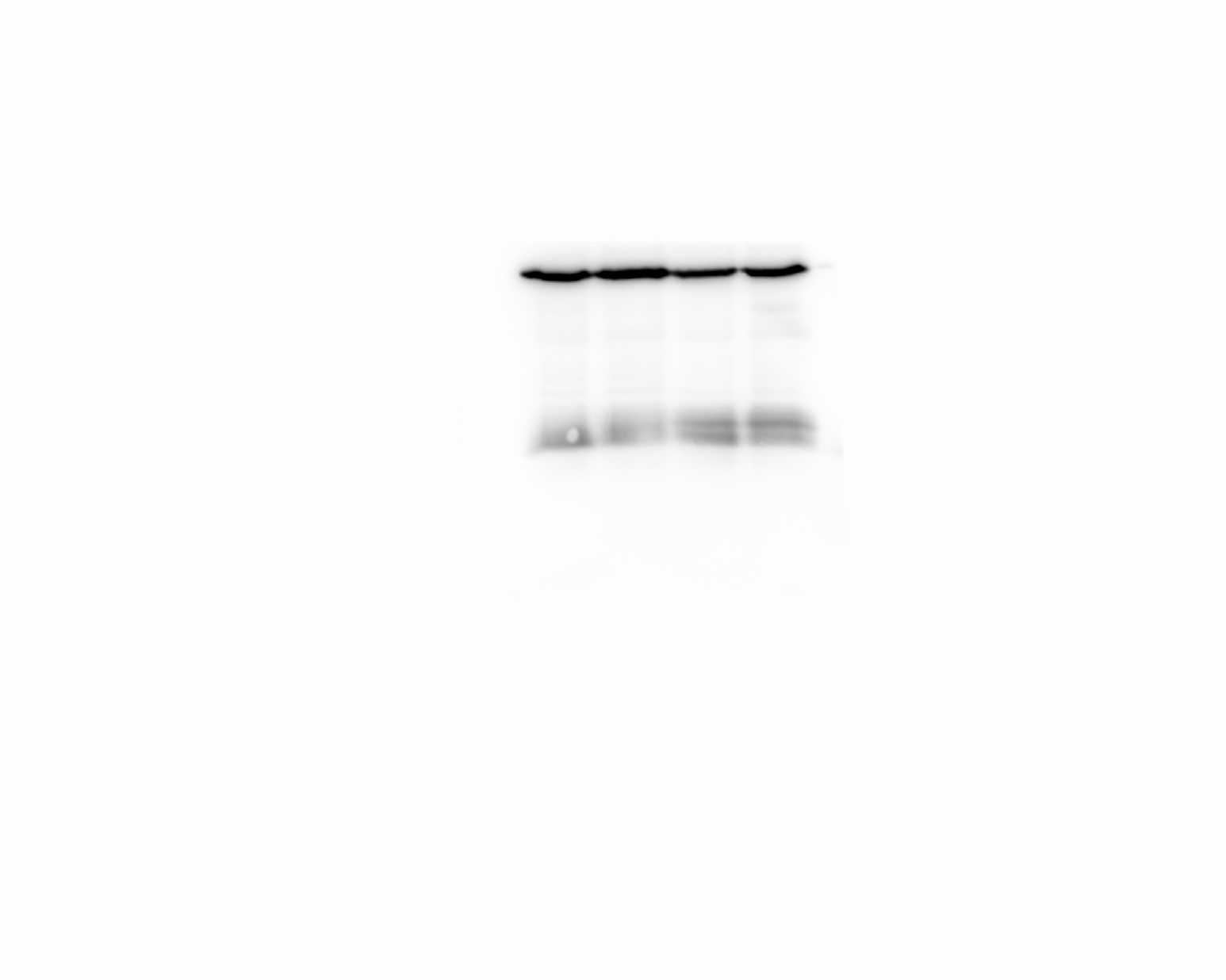

Supplement: Supplementary file 9 — Appendix Figure Source Data [file 44319_2026_803_MOESM9_ESM.zip › Appendix/Appendix S8/western Actin (for S6K_293T).tif]

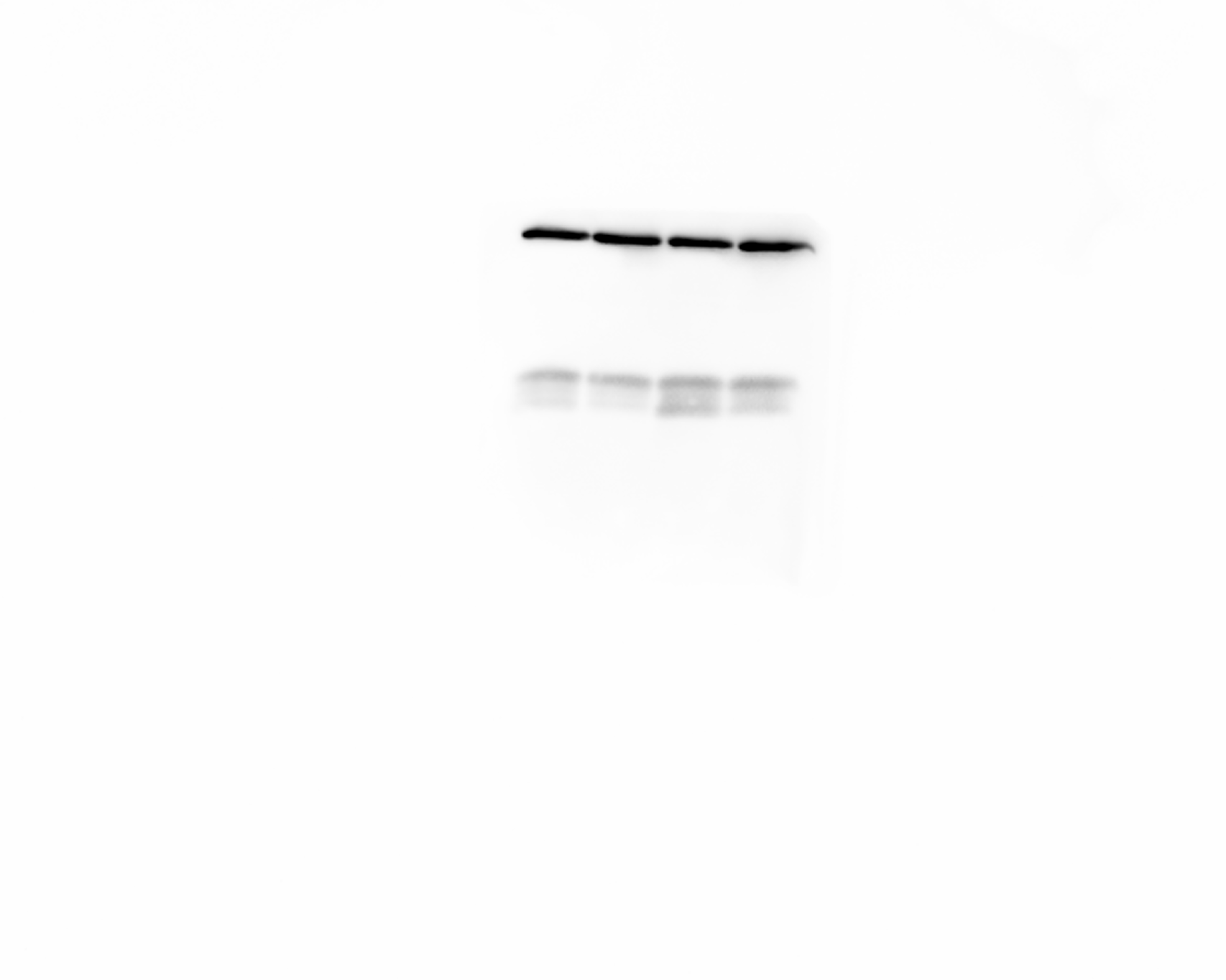

Supplement: Supplementary file 9 — Appendix Figure Source Data [file 44319_2026_803_MOESM9_ESM.zip › Appendix/Appendix S8/western Actin (for S6K_A549).tif]

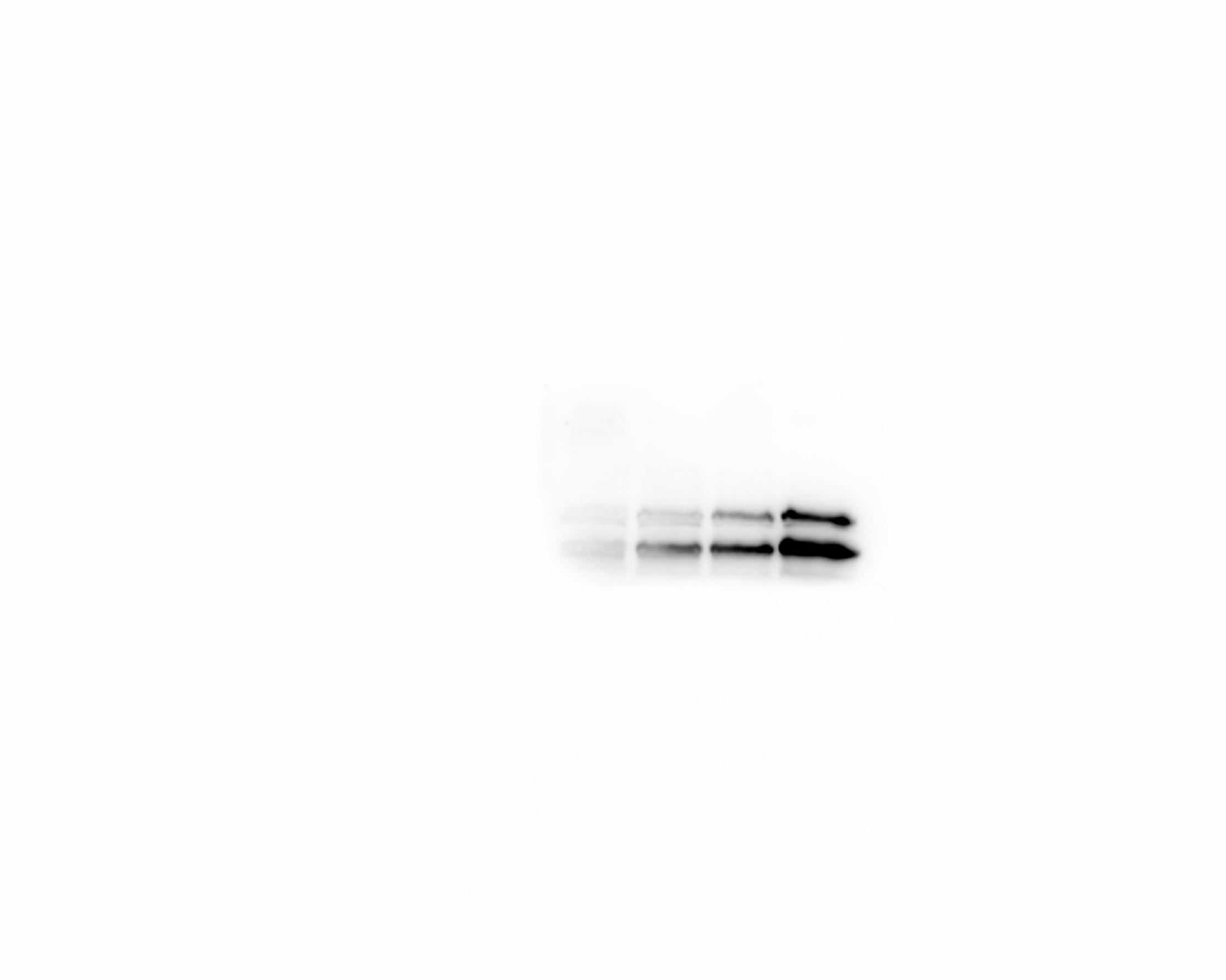

Supplement: Supplementary file 9 — Appendix Figure Source Data [file 44319_2026_803_MOESM9_ESM.zip › Appendix/Appendix S8/western p-S6K_293T.tif]
