# Supplementary figures and images for: SIRT2 mediates integrated stress response by deacetylating and stabilizing 4E-BP1 to suppress translation (part 2 of 2)
Source: EMBO Rep. 2026 May 18;27(11):3035–49. doi: 10.1038/s44319-026-00803-7 (PMC13260904; doi:10.1038/s44319-026-00803-7)

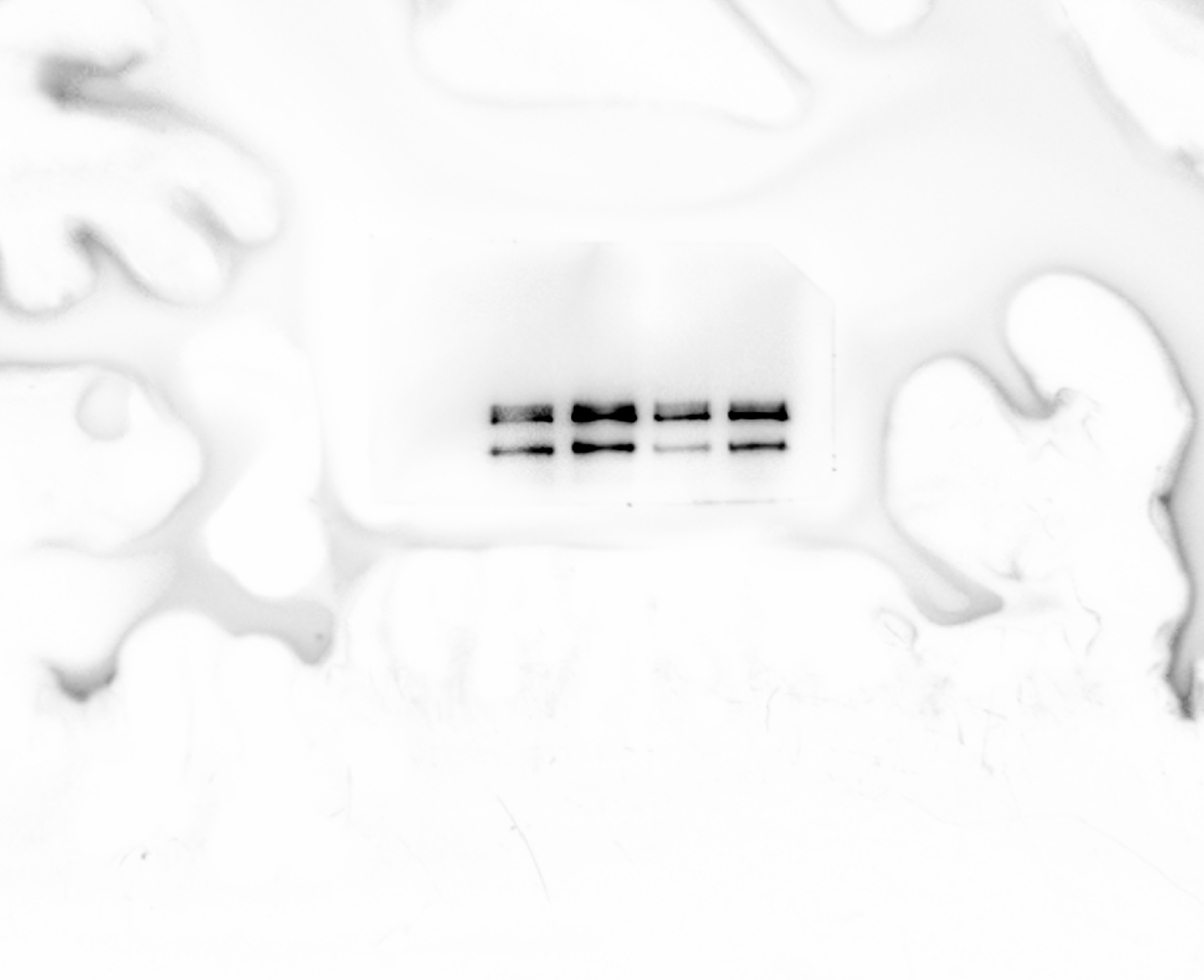

Supplement: Supplementary file 9 — Appendix Figure Source Data [file 44319_2026_803_MOESM9_ESM.zip › Appendix/Appendix S8/western p-S6K_A549.tif]

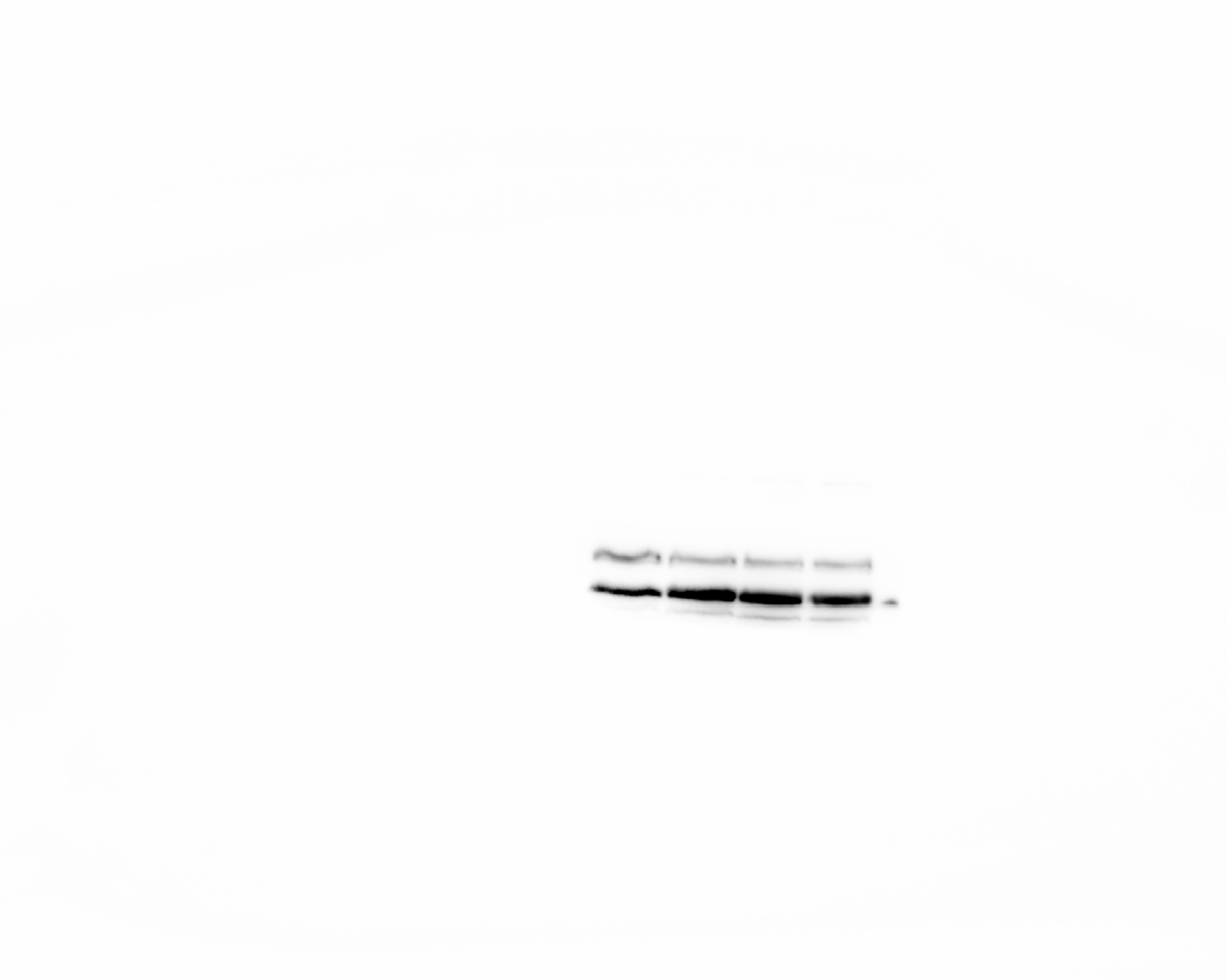

Supplement: Supplementary file 9 — Appendix Figure Source Data [file 44319_2026_803_MOESM9_ESM.zip › Appendix/Appendix S8/western S6K_293T.tif]

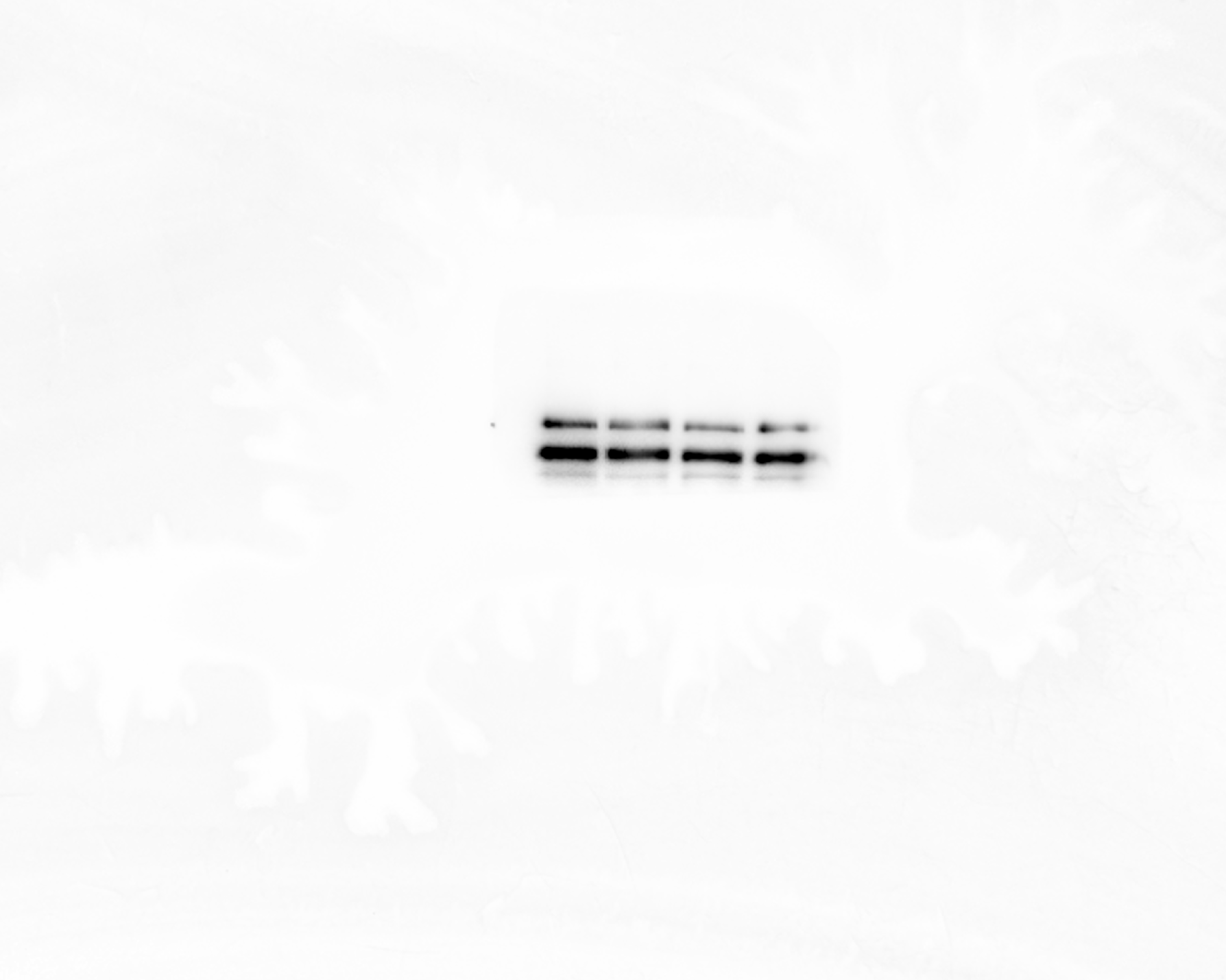

Supplement: Supplementary file 9 — Appendix Figure Source Data [file 44319_2026_803_MOESM9_ESM.zip › Appendix/Appendix S8/western S6K_A549.tif]

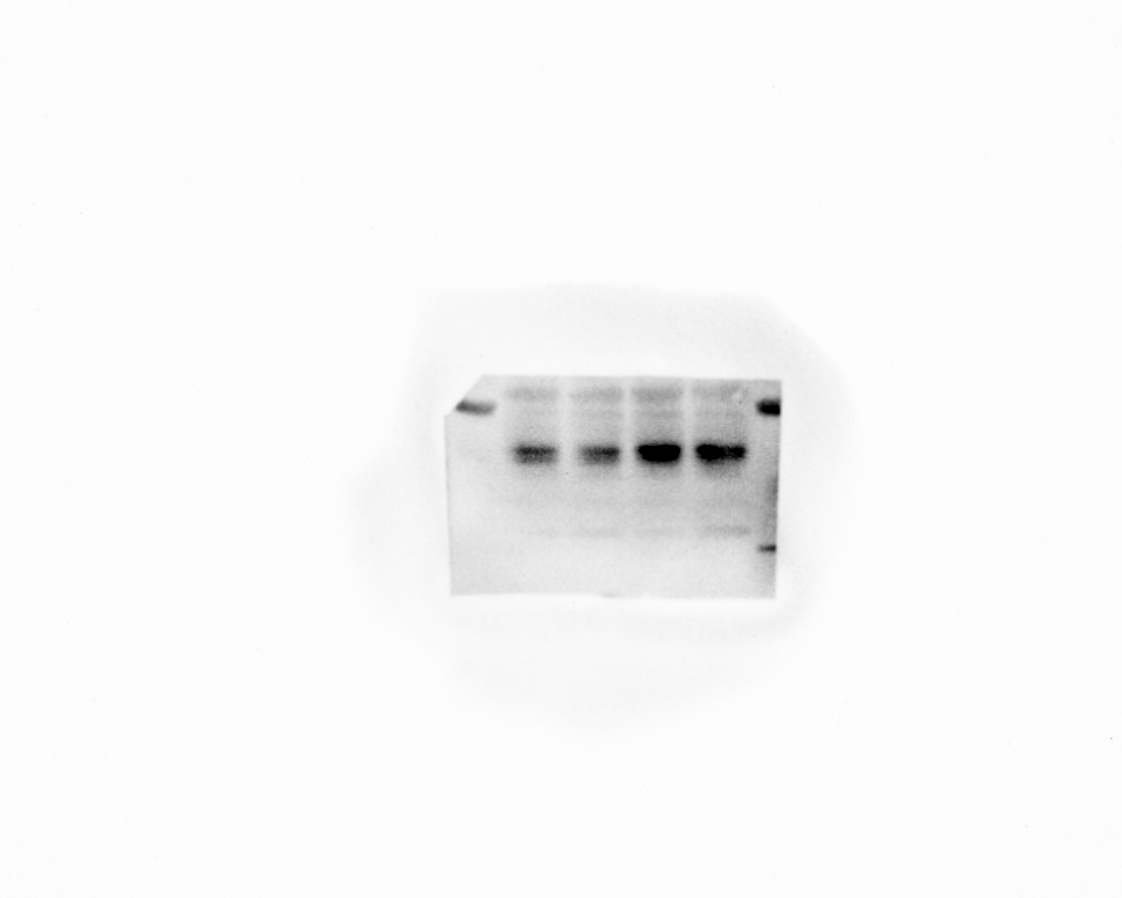

Supplement: Supplementary file 9 — Appendix Figure Source Data [file 44319_2026_803_MOESM9_ESM.zip › Appendix/Appendix S9/western AcIP 4EBP1.tif]

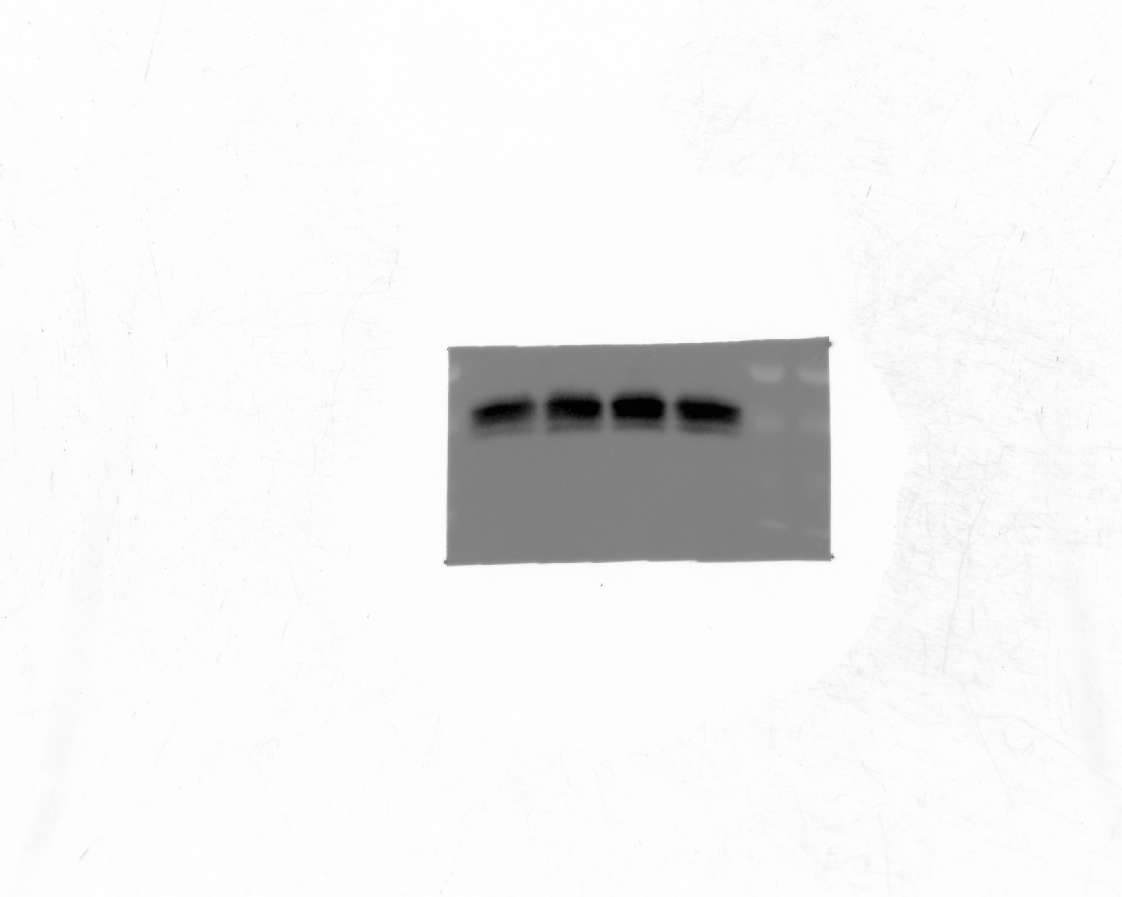

Supplement: Supplementary file 9 — Appendix Figure Source Data [file 44319_2026_803_MOESM9_ESM.zip › Appendix/Appendix S9/western input 4EBP1.tif]

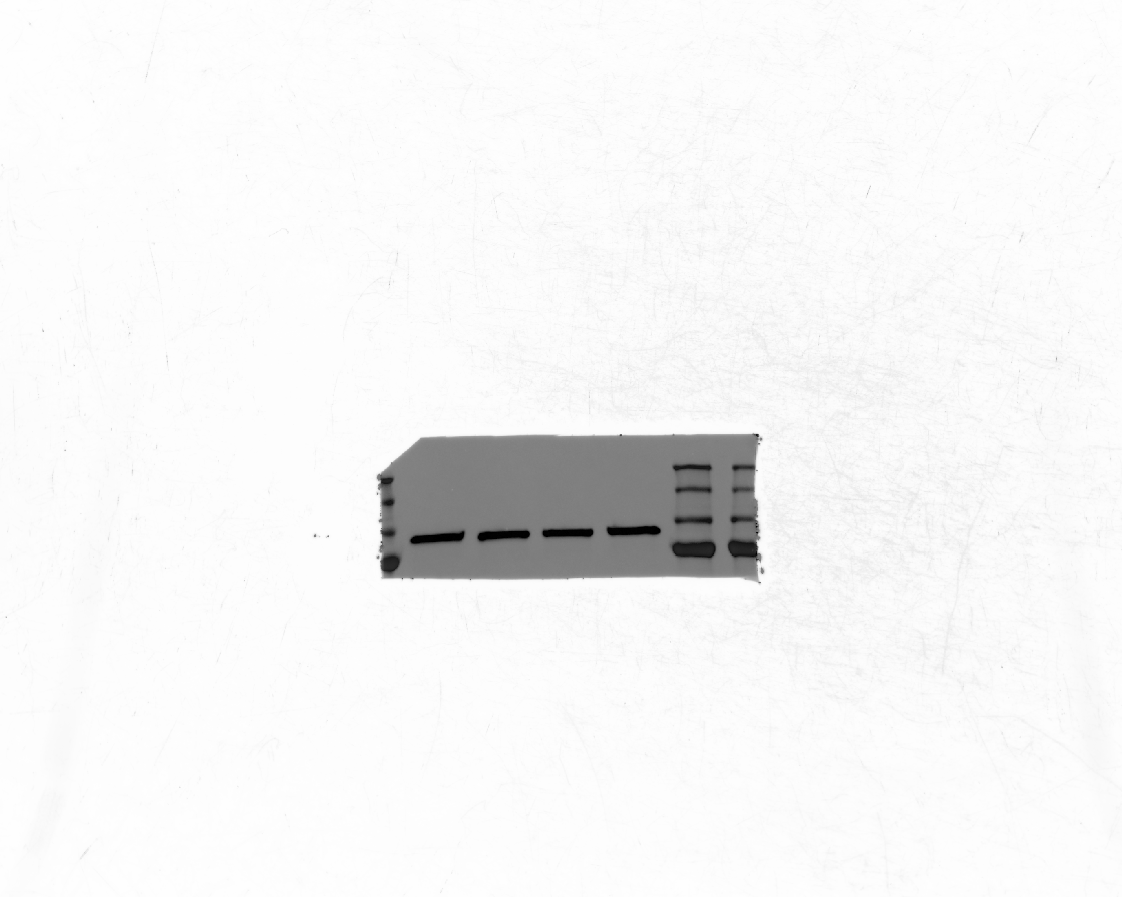

Supplement: Supplementary file 9 — Appendix Figure Source Data [file 44319_2026_803_MOESM9_ESM.zip › Appendix/Appendix S9/western input HSP90.tif]
