# Supplementary material for: SIRT2 mediates integrated stress response by deacetylating and stabilizing 4E-BP1 to suppress translation
Source: EMBO Rep. 2026 May 18;27(11):3035–49. doi: 10.1038/s44319-026-00803-7 (PMC13260904; doi:10.1038/s44319-026-00803-7)
Supplement: Supplementary file 2 — Peer Review File [file 44319_2026_803_MOESM2_ESM.pdf]

EMBO reports

SIRT2 Mediates Integrated Stress Response by Stabilizing 4EBP1 to Suppress Translation

Yanlin Zi, Jiaqi Zhao, Miao Wang, Dan Hou, Richard A. Cerione, and Hening Lin

Corresponding author(s): Hening Lin (linh1@uchicago.edu)

Review Timeline:

Submission Date:	29th Mar 25
Editorial Decision:	26th May 25
Revision Received:	20th Oct 25
Editorial Decision:	18th Dec 25
Revision Received:	3rd Apr 26
Accepted:	7th May 26

Editor: Esther Schnapp

Transaction Report:

(Note: With the exception of the correction of typographical or spelling errors that could be a source of ambiguity, letters and reports are not edited. Depending on transfer agreements, referee reports obtained elsewhere may or may not be included in this compilation. Referee reports are anonymous unless the Referee chooses to sign their reports.)

1st Editorial Decision 26th May 2025

Dear Prof. Lin,

Thank you for the submission of your manuscript to EMBO reports. We have now received the full set of referee reports that is pasted below.

As you will see, the referees acknowledge that the findings are potentially interesting. However, they also have several suggestions for how the ms should be improved and the data strengthened. I think all suggestions are good and should be addressed, except 2 requests by referee 3, which seem to be out of the scope of this study. These are "Which specific proteins target acetylated 4EBP1 for degradation?" and "How SIRT2 deletion affects stress responses in living organisms?" Naturally, if you already have data that address these points, please do add them to the ms.

Importantly, referee 3 provides additional comments on the back to back situation of both ms. It is true that despite shared authorships the two studies are not integrated and it remains unclear which mechanism of the 2 newly identified ones act when or in which context. I would like to ask you to address this issue and discuss with the authors of the related ms how both studies could be better integrated and related to each other. You can send me a proposed revision plan that we can discuss before you start revising your study. We can also talk about the revisions in a video chat, if you like.

I would thus like to invite you to revise your manuscript with the understanding that the referee concerns must be fully addressed and their suggestions taken on board. Please address all referee concerns in a complete point-by-point response. Acceptance of the manuscript will depend on a positive outcome of a second round of review. It is EMBO reports policy to allow a single round of major revision only and acceptance or rejection of the manuscript will therefore depend on the completeness of your responses included in the next, final version of the manuscript.

We realize that it is difficult to revise to a specific deadline. In the interest of protecting the conceptual advance provided by the work, we recommend a revision within 3 months (26th Aug 2025). Please discuss the revision progress ahead of this time with the editor if you require more time to complete the revisions.

IMPORTANT NOTE: we perform an initial quality control of all revised manuscripts before re-review. Your manuscript will FAIL this control and the handling will be DELAYED if the following APPLIES:
1) A data availability section providing access to data deposited in public databases is missing. If you have not deposited any data, please add a sentence to the data availability section that explains that.
2) Your manuscript contains statistics and error bars based on n=2. Please use scatter blots in these cases. No statistics should be calculated if n=2.

When submitting your revised manuscript, please carefully review the instructions that follow below. Failure to include requested items will delay the evaluation of your revision.

1) a .docx formatted version of the manuscript text (including legends for main figures, EV figures and tables). Please make sure that the changes are highlighted to be clearly visible.

2) individual production quality figure files as .eps, .tif, .jpg (one file per figure). See https://wol-prod-cdn.literatumonline.com/pb-assets/embo-site/EMBOPress_Figure_Guidelines_061115-1561436025777.pdf for more info on how to prepare your figures.

3) We replaced Supplementary Information with Expanded View (EV) Figures and Tables that are collapsible/expandable online. A maximum of 5 EV Figures can be typeset. EV Figures should be cited as 'Figure EV1, Figure EV2" etc... in the text and their respective legends should be included in the main text after the legends of regular figures.

- For the figures that you do NOT wish to display as Expanded View figures, they should be bundled together with their legends in a single PDF file called *Appendix*, which should start with a short Table of Content. Appendix figures should be referred to in the main text as: "Appendix Figure S1, Appendix Figure S2" etc. See detailed instructions regarding expanded view here: <https://www.embopress.org/page/journal/14693178/authorguide#expandedview>

- Additional Tables/Datasets should be labeled and referred to as Table EV1, Dataset EV1, etc. Legends have to be provided in a separate tab in case of .xls files. Alternatively, the legend can be supplied as a separate text file (README) and zipped together with the Table/Dataset file.

4) a .docx formatted letter INCLUDING the reviewers' reports and your detailed point-by-point responses to their comments. As part of the EMBO Press transparent editorial process, the point-by-point response is part of the Review Process File (RPF), which will be published alongside your paper.

5) a complete author checklist, which you can download from our author guidelines <https://www.embopress.org/page/journal/14693178/authorguide>. Please insert information in the checklist that is also

reflected in the manuscript. The completed author checklist will also be part of the RPF.

6) Please note that all corresponding authors are required to supply an ORCID ID for their name upon submission of a revised manuscript (<https://orcid.org/>). Please find instructions on how to link your ORCID ID to your account in our manuscript tracking system in our Author guidelines
<https://www.embopress.org/page/journal/14693178/authorguide#authorshipguidelines>

7) Before submitting your revision, primary datasets produced in this study need to be deposited in an appropriate public database (see https://www.embopress.org/page/journal/14693178/authorguide#datadeposition). Please remember to provide a reviewer password if the datasets are not yet public. The accession numbers and database should be listed in a formal "Data Availability" section placed after Materials & Method (see also
https://www.embopress.org/page/journal/14693178/authorguide#datadeposition). Please note that the Data Availability Section is restricted to new primary data that are part of this study. * Note - All links should resolve to a page where the data can be accessed. *
If your study has not produced novel datasets, please mention this fact in the Data Availability Section.

8) At EMBO Press we ask authors to provide source data for the main manuscript figures. You will receive a separate email with instructions for providing source data with your revised manuscript, including how to upload and organize the files.

9) Our journal also encourages inclusion of *data citations in the reference list* to directly cite datasets that were re-used and obtained from public databases. Data citations in the article text are distinct from normal bibliographical citations and should directly link to the database records from which the data can be accessed. In the main text, data citations are formatted as follows: "Data ref: Smith et al, 2001" or "Data ref: NCBI Sequence Read Archive PRJNA342805, 2017". In the Reference list, data citations must be labeled with "[DATASET]". A data reference must provide the database name, accession number/identifiers and a resolvable link to the landing page from which the data can be accessed at the end of the reference. Further instructions are available at https://www.embopress.org/page/journal/14693178/authorguide#referencesformat

10) Regarding data quantification (see Figure Legends:
https://www.embopress.org/page/journal/14693178/authorguide#figureformat)

The following points must be specified in each figure legend:

- the name of the statistical test used to generate error bars and P values,

- the number (n) of independent experiments (please specify technical or biological replicates) underlying each data point,

- the nature of the bars and error bars (s.d., s.e.m.),

- If the data are obtained from n Program fragment delivered error ``Can't locate object method "less" via package "than" (perhaps you forgot to load "than"?) at //ejpvfs23/sites23b/embor_www/letters/embor_decision_revise_and_review.txt line 56.' 2, use scatter blots showing the individual data points.

Discussion of statistical methodology can be reported in the materials and methods section, but figure legends should contain a basic description of n, P and the test applied.

- Please also include scale bars in all microscopy images.

11) The journal requires a statement specifying whether or not authors have competing interests (defined as all potential or actual interests that could be perceived to influence the presentation or interpretation of an article). In case of competing interests, this must be specified in your disclosure statement. Further information: https://www.embopress.org/competing-interests

12) All Materials and Methods need to be described in the main text using our 'Structured Methods' format, which is required for all research articles. According to this format, the Methods section includes a Reagents and Tools Table (listing key reagents, experimental models, software and relevant equipment and including their sources and relevant identifiers) followed by a Methods and Protocols section describing the methods using a step-by-step protocol format. The aim is to facilitate adoption of the methodologies across labs. More information on how to adhere to this format as well as a downloadable template (.docx) for the Reagents and Tools Table can be found in our author guidelines:
https://www.embopress.org/page/journal/14693178/authorguide#structuredmethods.

An example of a Method paper with Structured Methods can be found here: https://www.embopress.org/doi/full/10.1038/s44320-024-00037-6#sec-4

We would also welcome the submission of cover suggestions, or motifs to be used by our Graphics Illustrator in designing a

cover.

As part of the EMBO publication's Transparent Editorial Process, EMBO reports publishes online a Review Process File (RPF) to accompany accepted manuscripts. This File will be published in conjunction with your paper and will include the referee reports, your point-by-point response and all pertinent correspondence relating to the manuscript.

You are able to opt out of this by letting the editorial office know (emboreports@embo.org). If you do opt out, the Review Process File link will point to the following statement: "No Review Process File is available with this article, as the authors have chosen not to make the review process public in this case."

I look forward to seeing a revised form of your manuscript when it is ready.

Kind regards,
Esther

Esther Schnapp, PhD
Senior Editor
EMBO reports

Referee #1:

The manuscript by Zi et al. shows that the expression of SIRT2, a member of the Sirtuin family of NAD+-dependent deacetylases, is increased at the translational level following amino acid limitation. The translation of SIRT2 is suggested to be regulated by an upstream open reading frame (uORF) by the integrated stress response (ISR). SIRT2 is shown to regulate translation via deacetylation of 4EBP1, causing its stabilization, resulting in translational suppression. The core findings of the manuscript are of potential significance and will be of interest to the field; however, interpretation of some of the experiments, in particular the experiments describing the regulated translation of SIRT2 mRNA, is problematic. In addition, the lack of replicates and statistical rigor in some of the studies makes many experiments less convincing. Some additional major and minor comments on the manuscript are listed below.

Major Comments:

1. Starvation of MCF cells for non-essential amino acids (serine and glycine) is shown to induce SIRT2 expression. Does starvation of this cell line for serine and glycine induce the ISR? This is important to understand the proposed mechanism of translational expression of SIRT2.

2. No mention of replicates or statistical analysis is shown for the increased expression of SIRT2 in many parts of the manuscript. Biological and technical replicates must be clearly described, accompanied by rigorous statistical analysis. For example, Fig. 1A, 1C, 1D, 4E, 4F, and 5F.

3. In Fig. 2C, the experiment measuring SIRT2 protein stability shows no appreciable decrease in protein levels in either condition over the given time course. The time course needs to be altered to allow appreciable protein turnover in the control condition, to see if there are differences between control and AA limitation. In addition, starvation Leu, Lys, and Arg does not appear to induce SIRT2 expression in this experiment.

4. The authors propose that an uORF present in the 5'-UTR of SIRT2 serves to stimulate translation of SIRT2 following AA deprivation, analogous to the role of uORF1 in yeast GCN4. The uORF1 in GCN4 is a modest activator and mainly serves to allow for delayed reinitiation of translation of downstream ORFs. The interpretation that the SIRT2 uORF functions analogous to uORF1 in GCN4 is problematic. In addition, there appears to be an additional uORF at positions 26-88 in the 5'-UTR. What is the role of this uORF? Is the uORF at position 133-234 translated?

5. There is a presumption in the manuscript that increased eIF2 phosphorylation causes increased translation of SIRT2 mRNA. If eIF2 phosphorylation is blocked, does this cause a decrease in SIRT2 translation?

6. There appears to be multiple mRNA splice variants for SIRT2 mRNA and the manuscript focusses on variant 1. Do all splice variants contain the same 5'-UTR? Is variant 1 the major splice variant expressed in the cell lines discussed in the manuscript? Is the qRT-PCR analysis used to quantify SIRT2 mRNA specific for variant 1? A discussion of splice variants of SIRT2 would be helpful for the reader to fully appreciate the regulated expression of SIRT2.

7. Fig. S2. Overexpression of SIRT2 in MEF cells is suggested to reduce translation, but this is not clear from the immunoblot presented. Replicate experiments with appropriate statistical measures need to be presented to make this more convincing.

8. Does the inhibitor of SIRT2 also increase acetylation of endogenous 4EBP1?

Minor Comments:

1. It is not clear what is meant by "Amino acid deprivation is one of the four types of stresses that activate the integrated stress response (ISR)". There are multiple stressors that lead to activation of the four major eIF2 kinases to induce the ISR.

2. The manuscript switches cell lines frequently for each experiment. It would be helpful to indicate the cell line used in the individual figures, as has been done in Fig. 1b.

3. Increased expression of SIRT2 mRNA by amino acid deprivation could potentially be directly due to ATF4 or indirect by other transcription factors induced by ATF4.

4. Fig. S4, the quality of the blot does not allow the reader to interpret the results (lane 3).

Referee #2:

Review of Manuscript EMBOR-2025-61642V1 by Zi et al.
The manuscript presents novel findings linking the regulation of SIRT2 to the integrated stress response (ISR) under amino acid deprivation conditions. The authors demonstrate an inverse correlation between SIRT2 levels and global protein synthesis. Importantly, they identify for the first time that SIRT2 interacts with and deacetylates 4E-BP1, and that this correlates with changes in 4E-BP1 ubiquitination levels. The manuscript is of good quality, with mechanistic insights supporting most conclusions, and it will be of interest to EMBO Reports readers. However, the points below should be addressed to strengthen the manuscript further.
A key concern is that although the study links SIRT2 to both (i) suppression of translation and (ii) the deacetylation and stabilization of 4E-BP1, no direct experimental evidence is provided to establish a causal connection between these two processes.
Major Comments:
• In Figure 2B, the reported increase in SIRT2 protein levels under amino acid deprivation is not very pronounced. Quantification of Western blot bands would help to support this observation more robustly.
• The same applies to Figure S2; quantification would clarify the comparison between lanes 1 and 3.
• In the Figure 4C legend and panel, please indicate which phosphorylation site(s) on 4E-BP1 are being shown. The same clarification is needed for phospho-S6K in Figure S3.
• In Figure 4E, SIRT2 knockdown (S2KD) appears to reduce both hypo-phosphorylated (alpha) and hyper-/intermediate-phosphorylated (beta/gamma) forms of 4E-BP1 under both control and BafA conditions. This reduction in beta/gamma forms is not observed in previous panels. Please clarify the apparent discrepancy between Figures 4C/D and 4E regarding changes in 4E-BP1 phosphorylation states.
• The statement "Taken together, our data suggest that SIRT2 deacetylates 4E-BP1 at K69, and this deacetylation inhibits 4E-BP1 ubiquitination, preventing it from being degraded through the proteasome, which in turn decreases protein translation" (Figure 6) overstates the findings. While S2KD increases total protein synthesis (Figures 4A-B), no causal link is shown between this increase and the levels or acetylation state of 4E-BP1. Additional experiments are required to support this claim. If not provided, the manuscript should be revised to reflect the current evidence.
Minor Comments:
• Throughout the text-including the abstract, introduction, and figure legends-replace "protein translation" with either "mRNA translation" or "protein synthesis," as appropriate (noted 15 times).
• In the introduction, "defatty-acylase" should be replaced with "fatty-deacylase."
• Also in the introduction: the sentence "When 4E-BP1 undergoes SIRT2 catalyzes deacetylation at lysine 69 (K69)" should be corrected to "SIRT2 catalyzes deacetylation of 4E-BP1 at lysine 69 (K69)."
• In the legend of Figure 1, correct "MCF" to "MCF7."
• Indicating the cell lines directly in figure panels (e.g., Figures 1C and 1D) would improve clarity.
• In the sentence "We observed that when SIRT2 was knocked down in A549 and HEK293T cells, while phosphorylated 4E-BP1 levels remained unchanged, endogenous 4E-BP1 protein level was significantly decreased..." it would be clearer to replace "endogenous" with "total," since all forms of 4E-BP1 shown are endogenous. The same applies to "total" S6K in Figure S3.

Referee #3:

This study reveals an important new mechanism in cellular stress response. Zi et al. demonstrate how cells regulate protein production during amino acid shortages through a previously unknown pathway. Their work shows that the enzyme SIRT2

increases during amino acid stress through a specialized regulatory mechanism. SIRT2 then modifies a key protein called 4EBP1 by removing specific chemical tags (deacetylation at K69). This modification prevents 4EBP1's destruction and allows it to suppress protein production, helping cells adapt to stress conditions.
The identification of 4EBP1 as a SIRT2 target represents a significant advance in our understanding of translational control during stress. However, three important conceptual gaps require attention before this manuscript is suitable for publication in EMBO Reports.
Major Concerns
1. Causal mechanism between acetylation and ubiquitination
The authors show that SIRT2 removes acetyl groups from 4EBP1 at position K69 and that blocking acetylation at this site (using a K69R mutation) reduces protein degradation. However, the mechanistic connection between these events remains unclear. Key questions need addressing: - Which specific proteins target acetylated 4EBP1 for degradation? - How does acetylation create recognition sites for the degradation machinery? - What is the structural relationship between K69 acetylation and K57 ubiquitination?
2. Limited exploration of physiological relevance
The biological importance of this pathway requires stronger experimental support. While the authors demonstrate SIRT2 upregulation and 4EBP1 stabilization during stress, they haven't shown: - Whether this pathway is essential for cell survival during amino acid limitation - How SIRT2 deletion affects stress responses in living organisms - Whether 4EBP1 overexpression can rescue SIRT2-deficient cells
3. Incomplete characterization of SIRT2-4EBP1 interaction with mTORC1 signaling
The study needs to address how this newly discovered mechanism integrates with known regulatory pathways, particularly mTORC1 signaling. Critical questions include: - How does acetylation status affect mTORC1-mediated phosphorylation? - Do these modifications influence each other? - How do cells coordinate these different regulatory mechanisms during stress?
Minor Concerns
The specificity of SIRT2's response to amino acid stress requires additional validation through testing other nutrient stress conditions. Additionally, the authors should address how this mechanism relates to SIRT2's known role in translational control through RheB deacetylation, including their relative importance during stress responses.

Additional comments from referee 3:

I'm including some comments directly to you, considering them both together. For my nomenclature: Paper 1 = Zi et al. and Paper 2 = Sharma et al.

Overall, these companion papers present disconnected mechanisms for SIRT2's regulation of protein translation without adequate integration. For example, Paper 1 demonstrates SIRT2 deacetylates 4EBP1 downstream of mTORC1, while Paper 2 shows SIRT2 deacetylates Rheb upstream of mTORC1-with both ultimately suppressing translation; despite shared authorship, the manuscripts fail to reconcile these pathways, explain their relative importance in different contexts, or investigate potential competition between substrates; neither paper presents a unified model showing how these mechanisms interact during stress responses or in cardiac hypertrophy, leaving readers without a comprehensive understanding of SIRT2's role in translation regulation. So while both papers offer valuable insights into SIRT2's role in regulating protein translation, they present largely disconnected mechanisms without adequately addressing how these pathways interact. This represents a significant missed opportunity to develop an integrated framework that would better explain SIRT2's biological functions and potential therapeutic applications. For back-to-back publications, there's a surprising lack of integration that would provide a more comprehensive understanding of SIRT2's role in translation regulation. Here are some specific points:

1. Different Mechanisms for the Same Outcome
Both papers describe SIRT2's role in suppressing protein translation, but through entirely different mechanisms:
- Paper 1 claims SIRT2 deacetylates 4EBP1, stabilizing it and preventing eIF4E from initiating translation
- Paper 2 claims SIRT2 deacetylates Rheb, promoting its degradation and reducing mTORC1 activity

While complementary mechanisms are possible, neither paper acknowledges the alternative pathway described by the other, which is peculiar for back-to-back publications.

2. Insufficient Integration of Findings
Paper 2 briefly mentions Paper 1 in a single sentence: "Interestingly enough, in another manuscript under submission by Prof. Hening Lin's lab, SIRT2 is shown to deacetylate 4EBP1 leading to its stabilization, thereby suppressing translation by inactivating mTORC1."

However, this integration is minimal and doesn't address:
- The relative importance of each mechanism
- Whether these mechanisms operate simultaneously or in different contexts
- How these mechanisms interact in the regulatory network

3. Conflicting Statements About mTORC1 Inactivation

The papers present different accounts of how SIRT2 affects mTORC1:
- Paper 1 suggests SIRT2 acts downstream of mTORC1 by stabilizing 4EBP1 (which is a target of mTORC1)
- Paper 2 claims SIRT2 acts upstream of mTORC1 by destabilizing Rheb (which activates mTORC1)

Paper 2's brief mention that SIRT2's 4EBP1 deacetylation "suppress[es] translation by inactivating mTORC1" contradicts Paper 1, which doesn't claim 4EBP1 inactivates mTORC1 but rather acts downstream of it.

4. Incomplete Physiological Context
Both papers examine SIRT2's role in cardiac hypertrophy:
- Paper 1 only mentions it briefly, and states SIRT2 inhibitors have anticancer activity
- Paper 2 provides more detail about SIRT2-KO mice developing cardiac hypertrophy

However, neither paper fully integrates both mechanisms to explain cardiac hypertrophy development. How do the 4EBP1 and Rheb pathways together contribute to hypertrophy? Which pathway predominates in this condition?

5. Inadequate Comparative Analysis Under Stress Conditions
Both papers examine SIRT2 under stress conditions:
- Paper 1 focuses on amino acid limitation
- Paper 2 examines complete starvation (EBSS)

A systematic comparison of how these different stress conditions affect the two regulatory mechanisms (4EBP1 vs. Rheb) is missing. Do different types of nutrient stress preferentially activate one pathway over the other?

6. Experimental Design Differences
The papers use different experimental approaches:
- Paper 1 primarily uses amino acid deprivation experiments
- Paper 2 mainly uses SIRT2 inhibition, knockdown, and overexpression

While complementarity could be a strength, these differing approaches make direct comparison of results impossible, and neither paper attempts to reconcile methodological differences.

7. Shared Authorship Without Integrated Discussion
Despite having some shared authors (including Yanlin Zi and Hening Lin), the papers don't present a unified conceptual framework. This raises questions about whether these are truly complementary studies or competing models that haven't been fully reconciled.

We would like to thank the editor and reviewer for the helpful comments. We have addressed all the comments as detailed below. Editor and Reviewer's comments are shown in black fonts, while our responses are shown in blue fonts.

Referee #1:

The manuscript by Zi et al. shows that the expression of SIRT2, a member of the Sirtuin family of NAD+-dependent deacetylases, is increased at the translational level following amino acid limitation. The translation of SIRT2 is suggested to be regulated by an upstream open reading frame (uORF) by the integrated stress response (ISR). SIRT2 is shown to regulate translation via deacetylation of 4EBP1, causing its stabilization, resulting in translational suppression. The core findings of the manuscript are of potential significance and will be of interest to the field; however, interpretation of some of the experiments, in particular the experiments describing the regulated translation of SIRT2 mRNA, is problematic. In addition, the lack of replicates and statistical rigor in some of the studies makes many experiments less convincing. Some additional major and minor comments on the manuscript are listed below.

We thank the reviewers for the positive comments. We have addressed the concerns by adding more experimental data and enhancing the statistical rigor as detailed below.

Major Comments:

1. Starvation of MCF cells for non-essential amino acids (serine and glycine) is shown to induce SIRT2 expression. Does starvation of this cell line for serine and glycine induce the ISR? This is important to understand the proposed mechanism of translational expression of SIRT2.

To directly address the reviewer's question, we examined whether serine and glycine starvation induces the ISR in MCF7 cells. We cultured MCF7 cells in serine and glycine depleted medium for 6 or 12 hours and measured ATF4 expression by RT-qPCR as a marker of ISR activation. We observed an increase in ATF4 transcript levels under these conditions (**Figure R1**), indicating that the ISR was induced in MCF7 cells. Although the induction fold was modest, this magnitude is consistent with previous reports in other cell lines, including H1229 (Jin et al., *Cell Death Dis* **12**, 1127, 2021) and HT-29, HCT116, and DLD-1 (Tajan et al., *Nat Commun* 12, 366, 2021). These results support that serine and glycine deprivation is sufficient to activate the ISR in MCF7 cells and suggest that the translational upregulation of SIRT2 observed under these conditions occurs in the context of ISR activation.

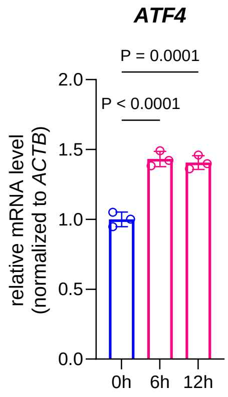

Figure R1. ATF4 expression is induced upon serine and glycine depletion in MCF7 cells. MCF7 cells were cultured with or without serine and glycine depletion for 6 or 12 hours. The amino acid limitation-induced ATF4 expression was assessed via RT-qPCR. Representative data from two biological replicates.

2. No mention of replicates or statistical analysis is shown for the increased expression of SIRT2 in many parts of the manuscript. Biological and technical replicates must be clearly described, accompanied by rigorous statistical analysis. For example, Fig. 1A, 1C, 1D, 4E, 4F, and 5F.

We have performed multiple biological replicates and have included the statistical analysis for Figure 1A, 1C, 1D, 4E, 4F, and 5F. Please find the statistical analysis and the corresponding methods in the revised figures and figure legends.

3. In Fig. 2C, the experiment measuring SIRT2 protein stability shows no appreciable decrease in protein levels in either condition over the given time course. The time course needs to be altered to allow appreciable protein turnover in the control condition, to see if there are differences between control and AA limitation. In addition, starvation Leu, Lys, and Arg does not appear to induce SIRT2 expression in this experiment.

To address the reviewer's concern, we optimized the CHX-chase experiment by increasing the CHX concentration to 100 µg/mL and extending the chase to 36 h. Under these conditions, we observed ~50% reduction of SIRT2 protein after 24 h of CHX treatment, with no significant difference between the +LKR and –LKR groups. Because our primary assay assessing SIRT2 expression differences is performed after 24 h of LKR depletion, this degree of turnover within the same time frame indicates that protein degradation does not account for the observed induction.

We would like to emphasize that in this specific experiment, induction of SIRT2 under amino acid limitation is not expected, as CHX blocks protein synthesis. Consistent with this, in the absence of CHX we observe significant upregulation of SIRT2 protein after

24 h of LKR depletion (see Fig. 1A, A549). Together, these results support a model in which SIRT2 is regulated predominantly at the level of translation rather than protein degradation.

4. The authors propose that an uORF present in the 5'-UTR of SIRT2 serves to stimulate translation of SIRT2 following AA deprivation, analogous to the role of uORF1 in yeast GCN4. The uORF1 in GCN4 is a modest activator and mainly serves to allow for delayed reinitiation of translation of downstream ORFs. The interpretation that the SIRT2 uORF functions analogous to uORF1 in GCN4 is problematic. In addition, there appears to be an additional uORF at positions 26-88 in the 5'-UTR. What is the role of this uORF? Is the uORF at position 133-234 translated?

We agree with the reviewer that uORF1 in GCN4 mainly functions to allow delayed reinitiation of downstream ORFs. However, prior work has also shown that removal of uORF1 decreases basal GCN4 expression even under normal growth conditions, which parallels our observation that mutation of the SIRT2 uORF (133–234) reduces basal reporter activity. For example, Abastado et al. (*Mol. Cell. Biol.*, 11: 486-496, 1991) reported that loss of uORF1 led to decreased basal GCN4 expression (see Figure 6, comparing p413 WT and p237 uORF1 mutant under regular conditions).

Regarding additional uORFs in the SIRT2 5′UTR, we tested the 26-88 uORF by mutating its AUG codon to AUA in our 5′UTR-luciferase construct and found no significant change in Firefly/Renilla luciferase activity (**Figure R2**), suggesting that this upstream element is not efficiently translated or does not play a regulatory role under our experimental conditions.

In contrast, mutation of the AUG codon of the 133-234 uORF caused a marked reduction in reporter activity (**Figure R2** and **3B**). While we did not directly demonstrate translation of this uORF in the endogenous SIRT2 transcript, these results strongly support that the 133-234 uORF is functionally translated in the context of the 5′UTR and acts to promote downstream SIRT2 expression. We have revised the main text of the manuscript to clarify this point.

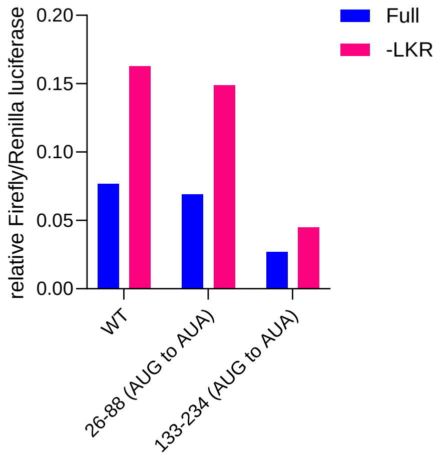

Figure R2. SIRT2 5'UTR luciferase reporter assay with uORF mutations. Firefly luciferase constructs containing either the WT SIRT2 5'UTR, a uORF 26-88 AUG mutant, or a uORF 133-234 AUG mutant were transfected into HeLa cells together with a *Renilla* luciferase control plasmid. The ratio of Firefly luciferase activity over *Renilla* luciferase activity was assessed with or without LKR (leucine, lysine, and arginine) starvation. Mutation of the 133-234 uORF, but not the 26-88 uORF, markedly reduced reporter activity. This figure has been added to the revised manuscript as **Figure S3**.

5. There is a presumption in the manuscript that increased eIF2 phosphorylation causes increased translation of SIRT2 mRNA. If eIF2 phosphorylation is blocked, does this cause a decrease in SIRT2 translation?

To address the reviewer's concern, we treated cells with ISRIB, an ISR inhibitor, and observed that amino acid limitation-induced SIRT2 protein level increases were diminished (**Figure R3**). Even though ISRIB does not directly block eIF2a phosphorylation, it serves as an alternative to counter the effects of eIF2a phosphorylation. Our results supported that ISR and eIF2 phosphorylation caused the increased translation of SIRT2 mRNA.

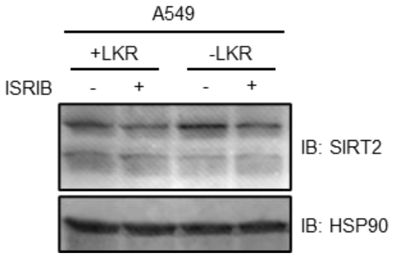

Figure R3. SIRT2 levels change upon ISRIB treatment. A549 cells were cultured with or without LKR depletion under 2 mM ISRIB for 24 hours. The amino acid limitation-induced SIRT2 protein level increases was diminished when treated with the ISR

inhibitor ISRIB. Representative data from two biological replicates. This figure has been added to the revised manuscript as **Figure S1**.

6. There appears to be multiple mRNA splice variants for SIRT2 mRNA and the manuscript focusses on variant 1. Do all splice variants contain the same 5'-UTR? Is variant 1 the major splice variant expressed in the cell lines discussed in the manuscript? Is the qRT-PCR analysis used to quantify SIRT2 mRNA specific for variant 1? A discussion of splice variants of SIRT2 would be helpful for the reader to fully appreciate the regulated expression of SIRT2.

We thank the reviewer for raising this point. Human SIRT2 has three RefSeq coding transcripts: variant 1 (NM_012237.3), variant 2 (NM_030593.3), and variant 3 (NM_001193286.2). According to RefSeq annotations, these transcripts share the same 5′ UTR sequence, and the uORFs we analyzed lie within this shared 5′ UTR. Thus, the 5′ UTR reporter derived from variant 1 is representative for the uORFs present in all three variants.

Regarding SIRT2 isoform expression, immunoblotting under our conditions detects protein species consistent with variants 1 and 2, with variant 1 predominant across the cell lines examined in this study. For transcript quantification, the RT-qPCR primers used in this study target exons common to all three variants; therefore, our measurements reflect total SIRT2 mRNA rather than variant-specific abundance. We have clarified this in the Methods section.

7. Fig. S2. Overexpression of SIRT2 in MEF cells is suggested to reduce translation, but this is not clear from the immunoblot presented. Replicate experiments with appropriate statistical measures need to be presented to make this more convincing.

Because we focused on human cell lines in most other experiments, we replaced this figure with data produced in HEK239T cells, where overexpression of SIRT2 was much more efficient (**Figure R4**, SIRT2 blot). With the better overexpression of SIRT2, we observed a much stronger decrease in puromycin labeling. We have also provided a statistical analysis in the revised manuscript.

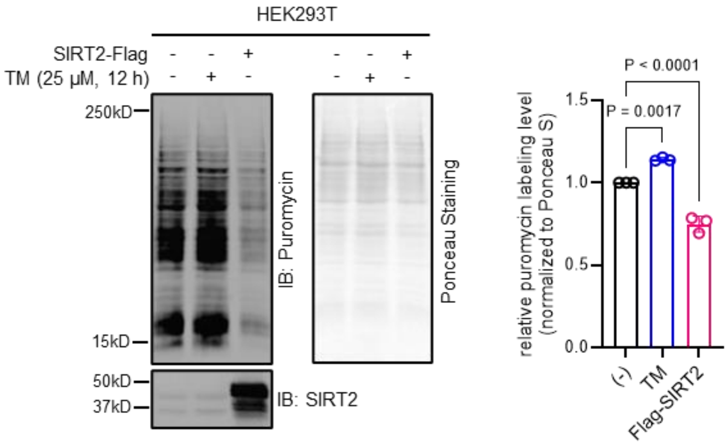

Figure R4. Puromycin labeling in HEK293T cells with SIRT2 overexpression or SIRT2 inhibitor TM treatment. Efficient SIRT2 overexpression was achieved in HEK293T cells. Representative data from three biologically independent replicates. Data with error bars are mean ± s.d. *P* values are determined using one-way ANOVA. Please refer to **Figure S4** in the revised manuscript.

8. Does the inhibitor of SIRT2 also increase acetylation of endogenous 4EBP1?

We examined the acetylation level of endogenous 4EBP1 with and without treatment of a SIRT2 inhibitor, TM. Our result suggested that inhibitor of SIR2 increased acetylation of endogenous 4EBP1 in a dose-dependent manner (**Figure R5**).

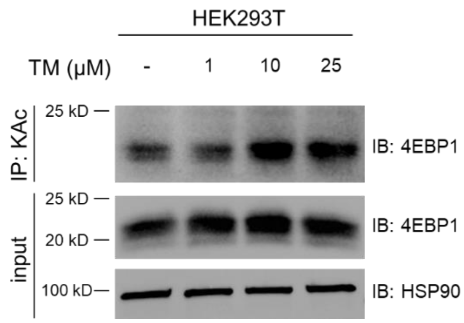

Figure R5. Acetylation IP of the endogenous 4EBP1 with SIRT2 inhibitor TM treatment. The acetylation levels of 4EBP1 increased upon TM treatment in a dose-dependent manner. Representative results from two biological replicates. This figure has been added to the revised manuscript as **Figure S6**.

Minor Comments:

1. It is not clear what is meant by "Amino acid deprivation is one of the four types of stresses that activate the integrated stress response (ISR)". There are multiple stressors that lead to activation of the four major eIF2 kinases to induce the ISR.

We rephrased this sentence to be “Amino acid deprivation is one of the stresses that activates the integrated stress response (ISR)”.

2. The manuscript switches cell lines frequently for each experiment. It would be helpful to indicate the cell line used in the individual figures, as has been done in Fig. 1b.

We thank the reviewer for this suggestion. We have indicated all cell lines in each individual figure for clarity.

3. Increased expression of SIRT2 mRNA by amino acid deprivation could potentially be directly due to ATF4 or indirect by other transcription factors induced by ATF4.

We thank the reviewer for this insightful question. While this is possible, we have shown that SIRT2 mRNA does not change upon amino acid deprivation (**Figure 2B**). If it is through ATF4, then we would expect SIRT2 mRNA to increase upon amino acid deprivation.

4. Fig. S4, the quality of the blot does not allow the reader to interpret the results (lane 3).

We have replaced this figure with a better-quality replicate as shown below (**Figure R6**).

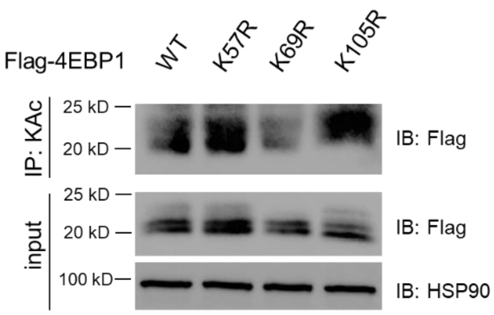

Figure R6. Acetylation level of 4EBP1 mutants. We have replaced the original **Figure S4** with this replicate. Please refer to **Figure S7** in the revised manuscript.

Referee #2:

The manuscript presents novel findings linking the regulation of SIRT2 to the integrated stress response (ISR) under amino acid deprivation conditions. The authors demonstrate an inverse correlation between SIRT2 levels and global protein synthesis. Importantly, they identify for the first time that SIRT2 interacts with and deacetylates 4E-BP1, and that this correlates with changes in 4E-BP1 ubiquitination levels. The

manuscript is of good quality, with mechanistic insights supporting most conclusions, and it will be of interest to EMBO Reports readers. However, the points below should be addressed to strengthen the manuscript further.
A key concern is that although the study links SIRT2 to both (i) suppression of translation and (ii) the deacetylation and stabilization of 4E-BP1, no direct experimental evidence is provided to establish a causal connection between these two processes.

Major Comments:

• In Figure 2B, the reported increase in SIRT2 protein levels under amino acid deprivation is not very pronounced. Quantification of Western blot bands would help to support this observation more robustly.

We have provided quantification and statistical analysis in the revised manuscript for Figure 2B.

• The same applies to Figure S2; quantification would clarify the comparison between lanes 1 and 3.

Because we focused on human cell lines in most other experiments, we replaced this figure with data produced in HEK239T cells, where overexpression of SIRT2 was much more efficient (**Figure R4**, SIRT2 blot). With the better overexpression of SIRT2, we observed a much stronger decrease in puromycin labeling. We have also provided a statistical analysis in the revised manuscript.

• In the Figure 4C legend and panel, please indicate which phosphorylation site(s) on 4E-BP1 are being shown. The same clarification is needed for phospho-S6K in Figure S3.

We have revised the figure legends and panel accordingly.

• In Figure 4E, SIRT2 knockdown (S2KD) appears to reduce both hypo-phosphorylated (alpha) and hyper-/intermediate-phosphorylated (beta/gamma) forms of 4E-BP1 under both control and BafA conditions. This reduction in beta/gamma forms is not observed in previous panels. Please clarify the apparent discrepancy between Figures 4C/D and 4E regarding changes in 4E-BP1 phosphorylation states.

We appreciate the reviewer’s careful observation. The panels in Figure 4C and 4E were run on hand-cast gels, whereas Figure 4D used a commercial gradient gel, which provides better resolution of the closely migrating 4E-BP1 phospho-isoforms (α vs β/γ). On hand-cast gels, the β and γ species partially co-migrate, making the β/γ region appear as a single, broader band; this can obscure changes in the γ fraction and give the impression of a uniform decrease across species. By contrast, the gradient gel in Figure 4D separates these isoforms more cleanly, allowing visualization of phospho-

state distribution without conflating bands. We have included this explanation in the manuscript.

• The statement "Taken together, our data suggest that SIRT2 deacetylates 4E-BP1 at K69, and this deacetylation inhibits 4E-BP1 ubiquitination, preventing it from being degraded through the proteasome, which in turn decreases protein translation" (Figure 6) overstates the findings. While S2KD increases total protein synthesis (Figures 4A-B), no causal link is shown between this increase and the levels or acetylation state of 4E-BP1. Additional experiments are required to support this claim. If not provided, the manuscript should be revised to reflect the current evidence.

We agree with the reviewer that we did not establish the causal relationship. We can only say that SIRT2 deacetylation of 4E-BP1 could be one mechanism contributing to SIRT2's effect on translation. In fact, our associated manuscript shows that SIRT2 also deacetylates and inhibits RheB. It is likely that deacetylation of both 4EBP1 and RheB contributes to the regulation of translation. So we have revised the statement to more accurately state our finding: "Taken together, our data suggest that SIRT2 deacetylates 4E-BP1 at K69, and this deacetylation inhibits 4E-BP1 ubiquitination, preventing it from being degraded through the proteasome. This could be one mechanism contributing to SIRT2's ability to suppress protein synthesis during amino acid limitation".

Minor Comments:

• Throughout the text-including the abstract, introduction, and figure legends-replace "protein translation" with either "mRNA translation" or "protein synthesis," as appropriate (noted 15 times).

We thank the reviewer for this suggestion and have revised all noted "protein translation" to "protein synthesis".

• In the introduction, "defatty-acylase" should be replaced with "fatty-deacylase."

We actually think "defatty-acylase" is the proper term to describe SIRT2's alternative function, but to avoid confusion, we rephrased the sentence to avoid using the term.

• Also in the introduction: the sentence "When 4E-BP1 undergoes SIRT2 catalyzes deacetylation at lysine 69 (K69)" should be corrected to "SIRT2 catalyzes deacetylation of 4E-BP1 at lysine 69 (K69)."

We have revised the manuscript accordingly.

• In the legend of Figure 1, correct "MCF" to "MCF7."

We have revised the manuscript accordingly.

• Indicating the cell lines directly in figure panels (e.g., Figures 1C and 1D) would improve clarity.

We thank the reviewer for this suggestion. We have indicated all cell lines in each individual figure for clarity.

• In the sentence "We observed that when SIRT2 was knocked down in A549 and HEK293T cells, while phosphorylated 4E-BP1 levels remained unchanged, endogenous 4E-BP1 protein level was significantly decreased..." it would be clearer to replace "endogenous" with "total," since all forms of 4E-BP1 shown are endogenous. The same applies to "total" S6K in Figure S3.
We thank the reviewer for this suggestion. We have replaced “endogenous” with “total”.

Referee #3:

This study reveals an important new mechanism in cellular stress response. Zi et al. demonstrate how cells regulate protein production during amino acid shortages through a previously unknown pathway. Their work shows that the enzyme SIRT2 increases during amino acid stress through a specialized regulatory mechanism. SIRT2 then modifies a key protein called 4EBP1 by removing specific chemical tags (deacetylation at K69). This modification prevents 4EBP1's destruction and allows it to suppress protein production, helping cells adapt to stress conditions.
The identification of 4EBP1 as a SIRT2 target represents a significant advance in our understanding of translational control during stress. However, three important conceptual gaps require attention before this manuscript is suitable for publication in EMBO Reports.

We thank the reviewer for the positive comments, and we addressed the concerns of the reviewer as detailed below.

Major Concerns

1. Causal mechanism between acetylation and ubiquitination

The authors show that SIRT2 removes acetyl groups from 4EBP1 at position K69 and that blocking acetylation at this site (using a K69R mutation) reduces protein degradation. However, the mechanistic connection between these events remains unclear. Key questions need addressing: - Which specific proteins target acetylated 4EBP1 for degradation? - How does acetylation create recognition sites for the degradation machinery? - What is the structural relationship between K69 acetylation and K57 ubiquitination?

We thank the reviewer for these insightful and potentially important questions. While we agree that these are valuable and need to be explored in future research, after discussion with the editors, we believe they fall outside the scope of the current study. We have added a sentence in the discussion to mention this.

2. Limited exploration of physiological relevance

The biological importance of this pathway requires stronger experimental support. While the authors demonstrate SIRT2 upregulation and 4EBP1 stabilization during stress, they haven't shown: - Whether this pathway is essential for cell survival during amino acid limitation - How SIRT2 deletion affects stress responses in living organisms - Whether 4EBP1 overexpression can rescue SIRT2-deficient cells

To address the reviewer's question, we performed the following experiment: we seeded an equal number of SIRT2 KD and control A549 cells and exposed them to lysine, arginine, and glutamine (KRQ) depletion. After 7 days, the cells were reseeded at a low confluency in complete medium and allowed to proliferate and form foci. The number and size of the foci formed were indications of cell survival under amino acid depletion. As shown in **Figure R7**, the numbers and sizes of foci formed were similar for control and SIRT2 KD cells under normal conditions. In contrast, control cells formed larger and more foci than SIRT2 KD cells after amino acid depletion. This result highlights that SIRT2 is essential for cell survival during amino acid limitation.

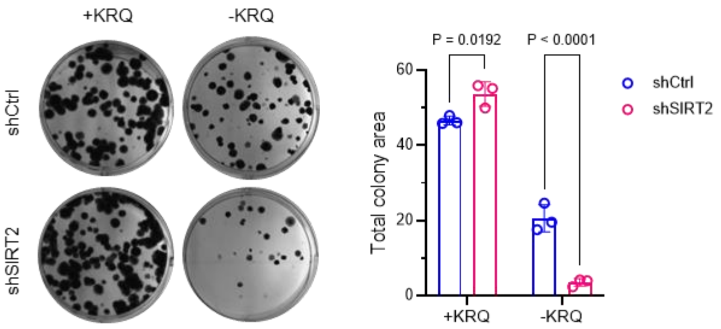

Figure R7. Clonogenic survival assays for SIRT2 KD and control A549 cells after KRQ depletion. Representative results from three biologically independent replicates. Data with error bars are mean ± s.d. *P* values are determined using 2way ANOVA. This figure has been added to the revised manuscript as **Figure S8**.

3. Incomplete characterization of SIRT2-4EBP1 interaction with mTORC1 signaling

The study needs to address how this newly discovered mechanism integrates with known regulatory pathways, particularly mTORC1 signaling. Critical questions include: -

How does acetylation status affect mTORC1-mediated phosphorylation? - Do these modifications influence each other? - How do cells coordinate these different regulatory mechanisms during stress?

To answer the reviewer's question, we performed the following experiment: we overexpressed 4E-BP1 WT, the acetylation-deficient mutant K69R, and the acetylation-mimic mutant K69Q, and examined 4E-BP1 phosphorylation level at Ser 65, a canonical mTORC1 target stie. We observed that the acetylation-deficient mutant (K69R) showed a significantly lower level of hyperphosphorylated 4E-BP1, whereas the acetylation-mimic mutant (K69Q) showed a significantly higher level of hyperphosphorylated form (**Figure R8**). This result indicates that the acetylation status of 4E-BP1 modulates its phosphorylation by mTORC1.

The above experiment was conducted in wildtype HEK293T cells, under the assumption that bulk mTORC1 activity remains similar across conditions. We note, however, that the regulatory landscape is more complex. Our accompanying manuscript demonstrates that SIRT2 also targets RheB, a direct activator of mTORC1. Specifically, SIRT2 deacetylates RheB at K151, which diminishes mTORC1 signaling. Thus, during nutrient stress, elevated SIRT2 levels may coordinate multiple nodes: (i) by deacetylating RheB, SIRT2 lowers mTORC1 activity, and (ii) by deacetylating 4E-BP1, SIRT2 reduces its phosphorylation competence. Together, these effects increase the proportion of hypophosphorylated 4E-BP1 bound to eIF4E, thereby restraining cap-dependent translation.

We therefore propose that during nutrient stress, cells upregulate SIRT2, which suppress protein synthesis via several different mechanisms. SIRT2 inhibition mTORC1 at the upstream and also regulate 4E-BP1, which is a mTORC1 substrate and more downstream, ensuring robust translational repression.

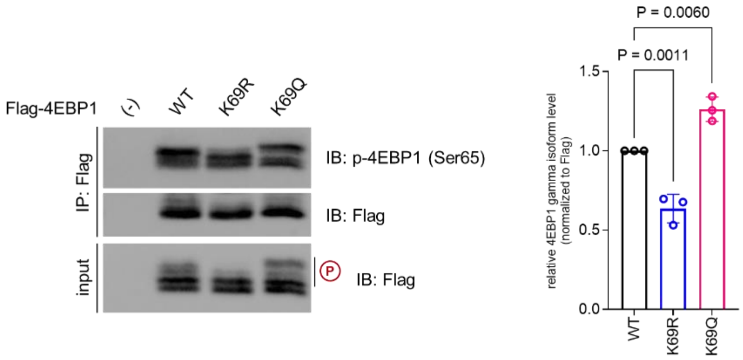

Figure R8. Phosphorylation of 4E-BP1 acetylation-deficient or acetylation-mimic mutants. HEK293T cells were transfected with either empty vector, 4E-BP1 wildtype (WT), acetylation-deficient mutant (K69R), or acetylation-mimic mutant (K69Q). After enrichment, phosphorylation of 4E-BP1 at Ser65 was examined via immunoblotting.

Representative results from three biologically independent replicates. P values were calculated with one-way ANOVA. This figure has been added to the revised manuscript as **Figure 5H** and **5I**.

Minor Concerns

The specificity of SIRT2's response to amino acid stress requires additional validation through testing other nutrient stress conditions. Additionally, the authors should address how this mechanism relates to SIRT2's known role in translational control through RheB deacetylation, including their relative importance during stress responses.

We appreciate the reviewer's point. Regarding specificity, we previously tested glucose limitation and did not observe an increase in SIRT2 levels under our experimental conditions. In contrast, amino acid depletion – tested using several different amino acid combinations - consistently induced SIRT2 upregulation across cell lines. We therefore believe that amino acid depletion is a robust and specific stress to induce SIRT2 levels.

As for the relationship to SIRT2's role in Rheb regulation, our accompanying manuscript demonstrates that SIRT2 deacetylates Rheb at K151 to suppress mTORC1 signaling. We therefore propose a dual mechanism: SIRT2 acts **upstream**, via Rheb deacetylation to attenuate mTORC1 activity, and **downstream**, via 4E-BP1 deacetylation to reduce its phosphorylation competence. Together, these two arms reinforce translational repression under stress. It is quite general that PTM enzymes regulate a pathway via multiple substrates. For example, mTORC1 promotes translation by phosphorylating both 4E-BP1 and S6K, as well as by. It is possible that in the future, new substrate proteins of SIRT2 are discovered that also contribute to the regulation of translation.

Additional comments from Referee 3:

I'm including some comments directly to you, considering them both together. For my nomenclature: Paper 1 = Zi et al. and Paper 2 = Sharma et al.

Overall, these companion papers present disconnected mechanisms for SIRT2's regulation of protein translation without adequate integration. For example, Paper 1 demonstrates SIRT2 deacetylates 4EBP1 downstream of mTORC1, while Paper 2 shows SIRT2 deacetylates Rheb upstream of mTORC1-with both ultimately suppressing translation; despite shared authorship, the manuscripts fail to reconcile these pathways, explain their relative importance in different contexts, or investigate potential competition between substrates; neither paper presents a unified model showing how these mechanisms interact during stress responses or in cardiac hypertrophy, leaving readers without a comprehensive understanding of SIRT2's role in

translation regulation. So while both papers offer valuable insights into SIRT2's role in regulating protein translation, they present largely disconnected mechanisms without adequately addressing how these pathways interact. This represents a significant missed opportunity to develop an integrated framework that would better explain SIRT2's biological functions and potential therapeutic applications. For back-to-back publications, there's a surprising lack of integration that would provide a more comprehensive understanding of SIRT2's role in translation regulation. Here are some specific points:

1. Different Mechanisms for the Same Outcome

Both papers describe SIRT2's role in suppressing protein translation, but through entirely different mechanisms:
- Paper 1 claims SIRT2 deacetylates 4EBP1, stabilizing it and preventing eIF4E from initiating translation
- Paper 2 claims SIRT2 deacetylates Rheb, promoting its degradation and reducing mTORC1 activity

While complementary mechanisms are possible, neither paper acknowledges the alternative pathway described by the other, which is peculiar for back-to-back publications.

We thank the reviewer for highlighting this important point. We agree that, taken in isolation, the two manuscripts describe distinct mechanisms by which SIRT2 suppresses protein synthesis. However, rather than being contradictory, these findings are complementary.

The accompanying manuscript establishes that SIRT2 deacetylates Rheb at K151, leading to its destabilization and reduced mTORC1 activity. In the current study, we show that SIRT2 also deacetylates 4E-BP1, decreasing its phosphorylation competence and thereby stabilizing its inhibitory interaction with eIF4E. Together, these findings reveal that SIRT2 suppresses translation through multiple mechanisms. It is quite general that PTM enzymes regulate a pathway via multiple substrates. For example, mTORC1 promotes translation by phosphorylating both 4E-BP1 and S6K. It is possible that in the future, new substrate proteins of SIRT2 are discovered that also contribute to the regulation of translation.

We acknowledge that the original versions of each manuscript did not explicitly reference the other pathway. We have now revised the *Discussion* section to highlight

this integration and to propose that SIRT2 functions as a multi-node regulator of translational control.

2. Insufficient Integration of Findings

Paper 2 briefly mentions Paper 1 in a single sentence: "Interestingly enough, in another manuscript under submission by Prof. Hening Lin's lab, SIRT2 is shown to deacetylate 4EBP1 leading to its stabilization, thereby suppressing translation by inactivating mTORC1."
However, this integration is minimal and doesn't address:

- The relative importance of each mechanism

- Whether these mechanisms operate simultaneously or in different contexts

- How these mechanisms interact in the regulatory network

As discussed above, we believe SIRT2 suppresses translation via multiple mechanisms to ensure tight regulation, just like mTORC1 regulates translation via multiple substrates. We believe these mechanisms operate simultaneously but definitive proof, as well as how these mechanisms interact, deserves its own separate study.

3. Conflicting Statements About mTORC1 Inactivation

The papers present different accounts of how SIRT2 affects mTORC1:

- Paper 1 suggests SIRT2 acts downstream of mTORC1 by stabilizing 4EBP1 (which is a target of mTORC1)

- Paper 2 claims SIRT2 acts upstream of mTORC1 by destabilizing Rheb (which activates mTORC1)
Paper 2's brief mention that SIRT2's 4EBP1 deacetylation "suppress[es] translation by inactivating mTORC1" contradicts Paper 1, which doesn't claim 4EBP1 inactivates mTORC1 but rather acts downstream of it.

We hope our replies above have addressed this concern. We do not think findings in the two papers contradict each other. Instead, we believe that SIRT2 controls translation through multiple mechanisms. These different mechanisms are to ensure efficient regulation.

4. Incomplete Physiological Context

Both papers examine SIRT2's role in cardiac hypertrophy:

- Paper 1 only mentions it briefly, and states SIRT2 inhibitors have anticancer activity

- Paper 2 provides more detail about SIRT2-KO mice developing cardiac hypertrophy

However, neither paper fully integrates both mechanisms to explain cardiac hypertrophy development. How do the 4EBP1 and Rheb pathways together contribute to hypertrophy? Which pathway predominates in this condition?

We think both mechanisms are likely to contribute to physiology, which is a typical mode of action for PTM enzymes. For example, mTOR phosphorylates many different substrate proteins to promote growth under nutrient-rich conditions. In our study, we focused on the mechanism through which SIRT2 is regulated and how it controls translation through 4E-BP1. The accompanying paper focused on physiological outcomes via Rheb in vivo. Together, these complementary papers highlight different but synergistic layers of SIRT2 function in regulating translation, both at the upstream through RheB and downstream through 4E-BP1.

5. Inadequate Comparative Analysis Under Stress Conditions

Both papers examine SIRT2 under stress conditions:

- Paper 1 focuses on amino acid limitation

- Paper 2 examines complete starvation (EBSS)

A systematic comparison of how these different stress conditions affect the two regulatory mechanisms (4EBP1 vs. Rheb) is missing. Do different types of nutrient stress preferentially activate one pathway over the other?

Our data indicates that both amino acid limitation and EBSS ultimately converge on the same outcome: upregulation of SIRT2 protein level. Once SIRT2 is elevated, its enzymatic activity is expected to act on multiple substrates, consistent with the typical mode of action of PTM enzymes. Therefore, we believe both RheB and 4E-BP1 pathways are engaged under these conditions.

While our study focused on amino acid limitation and the accompanying manuscript focused on EBSS, together our results suggest that SIRT2 functions as a stress-responsive regulator that can integrate nutrient stresses through multiple downstream effectors.

6. Experimental Design Differences

The papers use different experimental approaches:

- Paper 1 primarily uses amino acid deprivation experiments

- Paper 2 mainly uses SIRT2 inhibition, knockdown, and overexpression

While complementarity could be a strength, these differing approaches make direct comparison of results impossible, and neither paper attempts to reconcile methodological differences.

Both papers use SIRT2 inhibition, knockdown, and overexpression to examine the effect on translation and substrate acetylation/degradation. Again, we emphasize that the two papers are complementary instead of conflicting each other.

7. Shared Authorship Without Integrated Discussion

Despite having some shared authors (including Yanlin Zi and Hening Lin), the papers don't present a unified conceptual framework. This raises questions about whether these are truly complementary studies or competing models that haven't been fully reconciled.

We hope the expanded discussion in the revised manuscript have addressed this concern.

Dear Prof. Lin,

Thank you for the submission of your revised manuscript. We have now received the enclosed reports from the referees. Both referees 1 and 3 still have some concerns and suggestions that I would like you to address and incorporate before we can make a final decision on your ms.

Referee 1's comments must all be addressed. Referee 3 points 1 and 3 can be carefully discussed in the ms text. I am not sure whether you can address referee 3 points 2,4,5 ? Please let me know what you think.

Regarding the back-to-back publication, I would like to clarify that you will not need to experimentally integrate both papers, however, the discussion needs to be improved in this regard. At EMBO reports related papers can just be linked, which is different from a "true back-to-back publication" as mentioned by referee 4. In case there are obvious and straight forward experiments that can be done to increase our understanding of how both papers/mechanisms could be linked, these should be performed of course.

If we can agree on a set of revisions, there are also some editorial requests that will need to be addressed:

- Please reduce the number of keywords to 5.

- The resolution of the figure files is too low, please upload all figures as production-quality figure files.

- Please add a "Disclosure and Competing Interest Statement"

- Please remove the author credits from the ms file. All credits need to be entered during online ms submission.

- The REFERENCE format is not correct, it needs to be alphabetical, not numerical; et al needs to be used after 10 author names; DOIs should only be used for preprints and datasets that have not been published yet. Please correct to the EMBO reports reference style.

- All questions regarding statistics need to be answered in the author checklist. Please send us a new, completed checklist.

- The supplementary figures can either all be EV figures (you can have up to 8 EV figures), which need to be uploaded as individual files, or they can be bundled in an Appendix file, which needs a title page with the table of content and page numbers. In either case, the nomenclature will need to be corrected to either Figure EV1-EV8 or Appendix Figure S1, etc. in all places (throughout the files and in the ms callouts). You can find more information in our guide to authors online.

- Please upload the source data as one folder per main figure and one folder for all Appendix or EV figures.

- Please remove the lead contact section.

* Figure Legends - Comments *

- Please note that the exact p values are not provided in the legends of figures 1B, E, G; 2A, B; 3A, B, please provide exact values as reasonable.
- Please note that information related to n is missing in the legends of figures 3A, B, please add.

I would like to suggest some minor changes to the abstract that needs to be written in present tense. Please let me know if you agree with the following:

The ability to adapt to nutrient stress, such as amino acid limitation, is crucial for cell survival. The mTORC1 complex and integrated stress response (ISR) are two well-known mechanisms that sense the availability of amino acids and regulate protein synthesis. Here, we report a new SIRT2-mediated pathway, downstream of the ISR, that senses the limitation of amino acids to regulate translation. Under amino acid deprivation, SIRT2 protein levels are upregulated translationally by its upstream open reading frame (uORF). SIRT2 in turn suppresses global protein synthesis, which helps cells to survive amino acid limitation. We identify eukaryotic translation initiation factor 4E (eIF4E) binding protein 1 (4E-BP1) as a novel substrate of SIRT2, which inhibits protein synthesis by inhibiting eIF4E. SIRT2 deacetylates 4E-BP1 at lysine 69 and protects 4E-BP1 from proteasomal degradation. Our study reveals a novel role for SIRT2 in regulating protein synthesis and a new regulatory mechanism for 4E-BP1.

EMBO press papers are accompanied online by A) a short (1-2 sentences) summary of the findings and their significance, B) 2-3 bullet points highlighting key results and C) a synopsis image that is exactly 550 pixels wide and 200-600 pixels high (the height is variable). The synopsis image should provide a sketch of the major findings, like a graphical abstract. Please note that text needs to be readable at the final size. Please send us this information along with the final manuscript.

I look forward to seeing a newly revised manuscript as soon as possible.

Best regards,
Esther

Esther Schnapp, PhD
Senior Editor
EMBO reports

Referee #1:

This revised manuscript by Zi et al. investigates the regulation of the NAD^{+}-dependent deacetylase SIRT2 and proposes that its expression is modulated at the translational level via uORFs within the 5'-UTR of its mRNA during amino acid starvation. The authors further report that SIRT2-mediated deacetylation of 4EBP1 enhances its stability and thereby suppresses global protein synthesis. In the initial submission, several concerns were raised regarding the interpretation of key experiments underpinning these conclusions and the statistical rigor of the analyses. While some of these issues have been satisfactorily addressed, significant concerns remain-particularly those related to evidence supporting ISR-dependent control of SIRT2 expression and to the approaches used for data normalization. These outstanding issues are detailed below.
Concerns:

1. The ISR is suggested to be causative for SIRT2 expression; however, there are no measures of AA-depleted conditions inducing the ISR, and the modest increase in ATF4 mRNA is not sufficient. It would substantially bolster the manuscript to show that the conditions that increase SIRT2 expression in Fig. 1 in MCF7, A549, HCT116, and Hela cells also induce eIF2 phosphorylation, given that eIF2 phosphorylation is central to the proposed mechanism leading to increased SIRT2 translation. The assumption is that this increase is due to GCN2 activation; however, this is not currently addressed (or even mentioned) anywhere in the manuscript.

2. There appears to be no variance in the control samples in Figure 1B, 1E, 1G, 2B, 3A, 3B, 4G, 4I, 5C, 5E, 5G, and 5I. It is best practice to avoid pairwise normalization of samples for statistical analysis.

3. To examine the role of eIF2 phosphorylation in the translational control of SIRT2 mRNA, the authors treat amino acid-starved A549 cells with ISRIB. The experimental data presented in the immunoblot in Figure R3 are challenging to interpret. First, it does not appear that amino acid starvation induces SIRT2 expression in this experiment, as compared to, for example, the experiment shown in Fig. 1. Secondly, ISRIB seems to have only a very modest effect on SIRT2 expression, and it's not clear if this is significant. A control to verify that ISRIB reduces the ISR is not included in the experiment.

4. All other major and minor concerns have been addressed.

Referee #2:

The revised manuscript addressed the suggested modifications in a satisfactory manner.

Referee #3:

Reviewer Report: Zi et al. - SIRT2-mediated deacetylation of 4EBP1

Manuscript Summary
This manuscript demonstrates that SIRT2 is translationally upregulated during amino acid starvation through an ISR-linked mechanism involving a regulatory uORF. The authors show that elevated SIRT2 deacetylates 4EBP1 at K69, preventing ubiquitination and degradation, thereby stabilizing 4EBP1 and suppressing protein translation.

Major Strengths
Novel mechanistic insight: K69 acetylation site identification is well-validated
Comprehensive uORF characterization: 133-234 uORF functionally validated

Improved experimental rigor: Added replicates and statistical analyses
Physiological relevance: Clonogenic survival assays demonstrate SIRT2 importance
mTORC1 integration: K69 acetylation affects phosphorylation (Figure R8)

Remaining Major Concerns
1. Incomplete Acetylation-Ubiquitination Mechanism
E3 ligase not identified
Spatial/structural relationship between K69 acetylation and K57 ubiquitination is unclear
The authors claim "outside scope," but this is a critical mechanistic gap

Recommendation: Test candidate E3 ligases, perform structural modeling, or explicitly acknowledge this limitation

2. Under-Explored Phosphorylation-Acetylation Crosstalk
Figure R8 shows K69 acetylation modulates phosphorylation-a major finding buried in responses:
K69R (deacetylation-mimic) → reduced hyperphosphorylation
K69Q (acetylation-mimic) → increased hyperphosphorylation
Critical questions unaddressed:

Does acetylation affect mTORC1 binding?
Temporal dynamics of acetylation vs. phosphorylation?
Relative contribution when both mTORC1 activity and acetylation change?

Recommendation: Elevate this to a central finding with time-course, co-IP with mTORC1, and rapamycin effects on acetylation

3. Minimal Integration with Companion Manuscript
Only one sentence acknowledging the Rheb pathway
No Rheb measurements under amino acid starvation
No pathway hierarchy discussion
No substrate competition experiments
Recommendation: Add Rheb measurements in the same conditions OR substantially expand the Discussion explaining: Why 4EBP1 regulation matters if SIRT2 already inhibits mTORC1 via Rheb, Temporal dynamics and relative contributions, and whether effects are additive/synergistic/redundant

4. uORF Mechanism Incomplete
Translation of uORF inferred, not demonstrated
GCN4 analogy imperfect (single vs. multiple uORFs)
Mechanistic model unclear (slow scanning? reinitiation?)
Recommendation: An approach like ribosome profiling to demonstrate uORF translation; clarify the mechanistic model

5. Cell Survival Data Needs Expansion
Good addition, but lacks rescue experiments
Mechanism of reduced survival is unclear
Specificity to 4EBP1 not tested
Recommendation: SIRT2 re-expression rescue (WT but not catalytic mutant); ideally test if 4EBP1 overexpression rescues

Minor Concerns
ISRIB experiment (Fig R3): Only n=2, acts downstream of eIF2α-P. Need eIF2α S51A mutant for definitive test
Temporal dynamics missing: No time-course showing kinetics of all events
Cell line variability: Could include a summary table???
Acetyltransferase unknown: Would complete the regulatory cycle
Antibody validation: K69 acetyl-specific antibody not validated; may need mass spec confirmation
Discussion Needs: Comprehensive model figure showing full pathway including Rheb, Limitations section: E3 ligase, acetyltransferase, relative contribution vs. Rheb, Biological significance: Disease relevance, why PTM control needed beyond phosphorylation

More comments from referee 3:

These manuscripts should be published separately, not as integrated companion papers. While both describe SIRT2 regulating translation, they actually represent distinct stress-response pathways rather than a unified mechanism. Zi et al. show amino acid starvation increases SIRT2 protein levels (via ISR-mediated translation), which then stabilizes 4EBP1. Sharma et al. show glucose starvation increases SIRT2 activity (not protein), which degrades Rheb and inhibits mTORC1. These are fundamentally different regulatory contexts engaging SIRT2 through different mechanisms.

More importantly, there's no experimental evidence that the pathways work together. Despite reviewer requests, neither manuscript measures both substrates under the same conditions, tests substrate competition, or performs epistasis experiments. The "integration" exists only in the Discussion paragraphs, using nearly identical language about PTM enzymes having multiple substrates. The in vivo data from Sharma et al. actually suggest Rheb regulation dominates-SIRT2-KO hearts have high Rheb, low 4EBP1, yet still show increased protein synthesis and hypertrophy. If 4EBP1 stabilization were equally important, low 4EBP1 levels should provide some protection, but they don't.

The one finding that could meaningfully link them-Zi et al. Figure R8 showing K69 acetylation increases 4EBP1's phosphorylation competence-is buried in revision responses rather than integrated into either narrative. This could explain why both upstream (Rheb) and downstream (4EBP1) regulation matter, but neither manuscript develops this insight.

I would recommend publishing these as independent studies, each acknowledging the other exists but not claiming functional integration that isn't experimentally demonstrated. For true back-to-back publication, you would need at minimum: one experiment testing both substrates under the same stress conditions, an epistasis test showing whether 4EBP1 overexpression rescues SIRT2-KO phenotypes, and a unified model explaining when and where each mechanism predominates. Without this, they're two separate papers about SIRT2, not a coordinated study of multi-substrate regulation.

We would like to thank the editor and reviewers for the helpful comments. We have revised the manuscript accordingly. Review comments are shown in black fonts and our responses are shown in blue fonts.

Comments from Editor

Referee 1's comments must all be addressed. Referee 3 points 1 and 3 can be carefully discussed in the ms text. I am not sure whether you can address referee 3 points 2,4,5 ? Please let me know what you think.

We have tried to address all the comments from Reviewer 1 and 3.

Regarding the back-to-back publication, I would like to clarify that you will not need to experimentally integrate both papers, however, the discussion needs to be improved in this regard. At EMBO reports related papers can just be linked, which is different from a "true back-to-back publication" as mentioned by referee 4. In case there are obvious and straight forward experiments that can be done to increase our understanding of how both papers/mechanisms could be linked, these should be performed of course. Thanks for the clarification. We would like to publish the two papers as linked related papers. We have revised the manuscript's discussion to integrate both papers.

- Please reduce the number of keywords to 5.

We have reduced the number of keywords to 5.

- The resolution of the figure files is too low, please upload all figures as production-quality figure files.

We have uploaded high resolution figures.

- Please add a "Disclosure and Competing Interest Statement"

We have added this section.

- Please remove the author credits from the ms file. All credits need to be entered during online ms submission.

We have removed all information related to author credits.

- The REFERENCE format is not correct, it needs to be alphabetical, not numerical; et

al needs to be used after 10 author names; DOIs should only be used for preprints and datasets that have not been published yet. Please correct to the EMBO reports reference style.

We have corrected the reference format.

- All questions regarding statistics need to be answered in the author checklist. Please send us a new, completed checklist.

We have updated the author checklist.

- The supplementary figures can either all be EV figures (you can have up to 8 EV figures), which need to be uploaded as individual files, or they can be bundled in an Appendix file, which needs a title page with the table of content and page numbers. In either case, the nomenclature will need to be corrected to either Figure EV1-EV8 or Appendix Figure S1, etc. in all places (throughout the files and in the ms callouts). You can find more information in our guide to authors online.

We have updated the supplementary figures as the Appendix file.

- Please upload the source data as one folder per main figure and one folder for all Appendix or EV figures.

We have formatted the source data as required.

- Please remove the lead contact section.

We have removed the lead contact section.

* Figure Legends - Comments *

- Please note that the exact p values are not provided in the legends of figures 1B, E, G; 2A, B; 3A, B, please provide exact values as reasonable.

- Please note that information related to n is missing in the legends of figures 3A, B, please add.

We have updated all figure legends with the exact p values. We have provided information related to n in the legends of Figure 3A and 3B (n=3).

I would like to suggest some minor changes to the abstract that needs to be written in present tense. Please let me know if you agree with the following:

The ability to adapt to nutrient stress, such as amino acid limitation, is crucial for cell survival. The mTORC1 complex and integrated stress response (ISR) are two well-known mechanisms that sense the availability of amino acids and regulate protein synthesis. Here, we report a new SIRT2-mediated pathway, downstream of the ISR, that senses the limitation of amino acids to regulate translation. Under amino acid deprivation, SIRT2 protein levels are upregulated translationally by its upstream open reading frame (uORF). SIRT2 in turn suppresses global protein synthesis, which helps cells to survive amino acid limitation. We identify eukaryotic translation initiation factor 4E (eIF4E) binding protein 1 (4E-BP1) as a novel substrate of SIRT2, which inhibits protein synthesis by inhibiting eIF4E. SIRT2 deacetylates 4E-BP1 at lysine 69 and protects 4E-BP1 from proteasomal degradation. Our study reveals a novel role for SIRT2 in regulating protein synthesis and a new regulatory mechanism for 4E-BP1.

We thank the editor's suggestions and agree with this version of the abstract.

EMBO press papers are accompanied online by A) a short (1-2 sentences) summary of the findings and their significance, B) 2-3 bullet points highlighting key results and C) a synopsis image that is exactly 550 pixels wide and 200-600 pixels high (the height is variable). The synopsis image should provide a sketch of the major findings, like a graphical abstract. Please note that text needs to be readable at the final size. Please send us this information along with the final manuscript.

We have included the information requested.

Referee #1:

This revised manuscript by Zi et al. investigates the regulation of the NAD^+-dependent deacetylase SIRT2 and proposes that its expression is modulated at the translational level via uORFs within the 5'-UTR of its mRNA during amino acid starvation. The authors further report that SIRT2-mediated deacetylation of 4EBP1 enhances its stability and thereby suppresses global protein synthesis. In the initial submission, several concerns were raised regarding the interpretation of key experiments underpinning these conclusions and the statistical rigor of the analyses. While some of these issues have been satisfactorily addressed, significant concerns remain-particularly those related to evidence supporting ISR-dependent control of SIRT2

expression and to the approaches used for data normalization. These outstanding issues are detailed below.

Concerns:

1. The ISR is suggested to be causative for SIRT2 expression; however, there are no measures of AA-depleted conditions inducing the ISR, and the modest increase in ATF4 mRNA is not sufficient. It would substantially bolster the manuscript to show that the conditions that increase SIRT2 expression in Fig. 1 in MCF7, A549, HCT116, and Hela cells also induce eIF2 phosphorylation, given that eIF2 phosphorylation is central to the proposed mechanism leading to increased SIRT2 translation. The assumption is that this increase is due to GCN2 activation; however, this is not currently addressed (or even mentioned) anywhere in the manuscript.

We thank the reviewer for this important suggestion. To directly demonstrate that the same amino acid depletion conditions used in **Figure 1** to induce SIRT2 also activate the ISR, we measured eIF2α phosphorylation (Ser51) in all four cell lines (MCF7, A549, HCT116, and HeLa) under lysine/arginine starvation (KR, 24 h). As shown in **Figure R9**, amino acid starvation increased p-eIF2α (Ser51) in each cell line under the same conditions where SIRT2 protein levels increase, supporting ISR induction and strengthening the proposed mechanism in which eIF2α phosphorylation promotes increased SIRT2 translation.

In addition, we have expanded the discussion to address the upstream kinase responsible for eIF2α phosphorylation under amino acid limitation. Because GCN2 is the canonical ISR kinase activated by amino acid deprivation, we now discuss GCN2 as the most likely mediator in our KR starvation setting and clarify this point in the revised text.

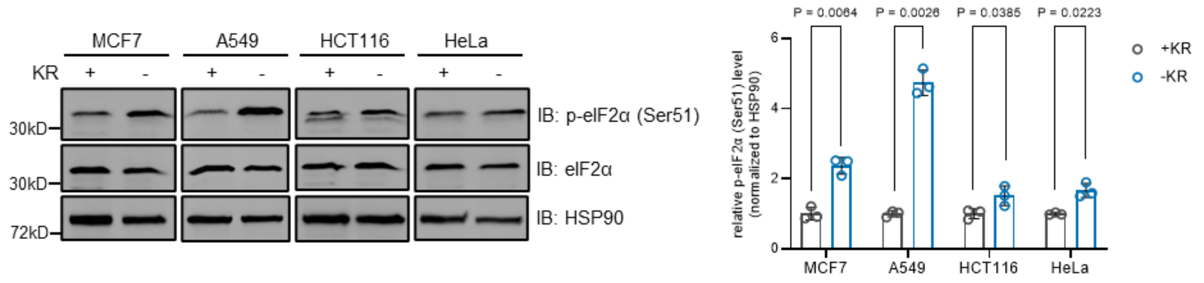

Figure R9. Amino acid starvation conditions that increase SIRT2 also induce eIF2α phosphorylation. p-eIF2α (Ser51) level is upregulated in multiple cell lines when starved with lysine and arginine (KR) for 24 hrs. p-eIF2α (Ser51) and total eIF2α levels in MCF7, A549, HCT116, and HeLa cells with or without lysine and arginine starvation were measured by western blot. Representative data from three biologically

independent experiments. This figure has been added to the revised manuscript as **Appendix Figure S1**.

2. There appears to be no variance in the control samples in Figure 1B, 1E, 1G, 2B, 3A, 3B, 4G, 4I, 5C, 5E, 5G, and 5I. It is best practice to avoid pairwise normalization of samples for statistical analysis.

We thank the reviewer for this comment. We have updated most of the figures to include variance in the control samples. However, for some experiments, such as the ubiquitination levels in **Figure 4I** and **5G**, the immunoblot signals can vary substantially across independent runs (antibody incubation, exposure, imaging, etc.), we therefore report the data as within-replicate normalized values (scaled to the matched control), which necessarily sets the control to 1 by definition and thus does not display between-experiment variance in the control group. Specifically, for each replicate, all conditions were run on the same gel/membrane, and band intensities were quantified and normalized to the loading control (e.g. Flag IP signal) from the same membrane and corresponding lanes. In these cases, we utilized the one-sample t-test (compared to a hypothetical mean set to 1) to reflect statistical significance, and we have specified in the figure legends. We also acknowledge that the relatively small number of biological replicates (n = 3) may limit statistical power. Nonetheless, the direction and magnitude of the effects are reproducible across these independent biological replicates, supporting the conclusions. We hope this explanation addresses the reviewer’s concerns.

3. To examine the role of eIF2 phosphorylation in the translational control of SIRT2 mRNA, the authors treat amino acid-starved A549 cells with ISRIB. The experimental data presented in the immunoblot in Figure R3 are challenging to interpret. First, it does not appear that amino acid starvation induces SIRT2 expression in this experiment, as compared to, for example, the experiment shown in Fig. 1. Secondly, ISRIB seems to have only a very modest effect on SIRT2 expression, and it's not clear if this is significant. A control to verify that ISRIB reduces the ISR is not included in the experiment.

We thank the reviewer for raising these concerns regarding the ISRIB experiment. To address this, we repeated the experiment in A549 cells and included additional ISR controls (**Figure R10**). Under LKR amino acid depletion for 24 h, SIRT2 protein levels increased, consistent with **Figure 1**. Importantly, ISRIB treatment attenuated the starvation-induced increase in SIRT2. In the same samples, p-eIF2α (Ser51) and ATF4 were strongly induced by amino acid starvation, confirming robust ISR activation. As a functional control for ISRIB activity, ISRIB reduced ATF4 protein abundance while leaving p-eIF2α (Ser51) unchanged, consistent with ISRIB acting downstream of eIF2α

phosphorylation to restore translation. Together, these data support that eIF2α phosphorylation-dependent ISR signaling contributes to the translational upregulation of SIRT2 under amino acid limitation.

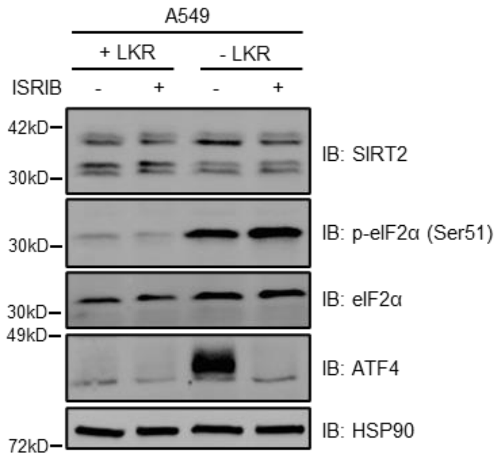

Figure R10. ISRIB attenuates amino acid starvation–induced SIRT2 upregulation and suppresses ISR output downstream of eIF2α phosphorylation. A549 cells were cultured with or without LKR depletion under 2 μM ISRIB or DMSO for 24 hours. p-eIF2α (Ser51) and ATF4 were included as ISR markers. Amino acid starvation induced p-eIF2α (Ser51) and ATF4 and increased SIRT2 protein levels; ISRIB reduced ATF4 and attenuated the starvation-induced increase in SIRT2 without reducing p-eIF2α (Ser51), consistent with ISRIB acting downstream of eIF2α phosphorylation. Representative data from three biological replicates. This figure has been added to the revised manuscript as **Appendix Figure S2**.

4. All other major and minor concerns have been addressed.

We thank the reviewer for this positive feedback.

Referee #2:

The revised manuscript addressed the suggested modifications in a satisfactory manner.

We thank the reviewer for this positive feedback.

Referee #3:

Manuscript Summary

This manuscript demonstrates that SIRT2 is translationally upregulated during amino acid starvation through an ISR-linked mechanism involving a regulatory uORF. The authors show that elevated SIRT2 deacetylates 4EBP1 at K69, preventing ubiquitination and degradation, thereby stabilizing 4EBP1 and suppressing protein translation.

Major Strengths

Novel mechanistic insight: K69 acetylation site identification is well-validated Comprehensive uORF characterization: 133-234 uORF functionally validated Improved experimental rigor: Added replicates and statistical analyses Physiological relevance: Clonogenic survival assays demonstrate SIRT2 importance mTORC1 integration: K69 acetylation affects phosphorylation (Figure R8)

Remaining Major Concerns

1. Incomplete Acetylation-Ubiquitination Mechanism

E3 ligase not identified
Spatial/structural relationship between K69 acetylation and K57 ubiquitination is unclear
The authors claim "outside scope," but this is a critical mechanistic gap

Recommendation: Test candidate E3 ligases, perform structural modeling, or explicitly acknowledge this limitation

We appreciate the reviewer's concern regarding the identity of the E3 ligase responsible for 4E-BP1 ubiquitination. A prior Molecular Cell study (doi: 10.1016/j.molcel.2012.04.004) identified KLHL25–CUL3 as an E3 ligase complex that ubiquitinates and promotes degradation of hypophosphorylated 4E-BP1. Our data show that K69 acetylation predominantly affects the hypophosphorylated α form of 4E-BP1, aligning with a phospho-state-selective degradation mechanism. Consistent with this, we found that under SIRT2 knockdown, CUL3 has a stronger interaction with 4E-BP1 WT compared to the K69R mutant, as evidenced by the higher level of co-immunoprecipitated 4E-BP1 WT with CUL3 pull-down (**Figure R11**), suggesting that the KLHL25–CUL3 complex may participate in this regulation.

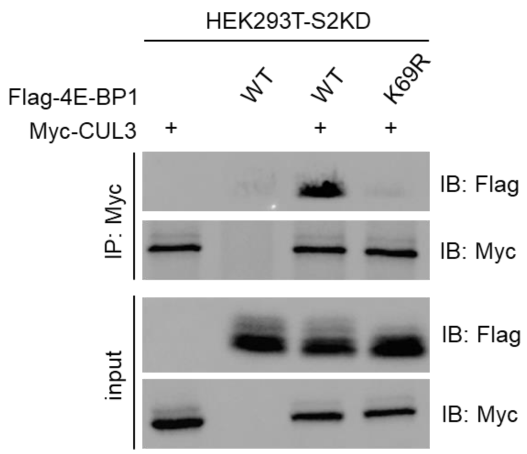

Figure R11. 4E-BP1 binds more strongly to CUL3 than K69R. HEK293T-SIRT2 KD cells were transfected with Flag-4E-BP1, Myc-CUL3, or both. Cells were collected and lysed via freeze-thaw cycles. Myc-CUL3 were then immunoprecipitated, and the interactions between 4E-BP1 and CUL3 were examined via western blotting. Representative data from three biological replicates. This figure has been added to the revised manuscript as **Appendix Figure S11**.

2. Under-Explored Phosphorylation-Acetylation Crosstalk

Figure R8 shows K69 acetylation modulates phosphorylation-a major finding buried in responses:
K69R (deacetylation-mimic) → reduced hyperphosphorylation

K69Q (acetylation-mimic) → increased hyperphosphorylation

Critical questions unaddressed:

Does acetylation affect mTORC1 binding?

Temporal dynamics of acetylation vs. phosphorylation?

Relative contribution when both mTORC1 activity and acetylation change?

Recommendation: Elevate this to a central finding with time-course, co-IP with mTORC1, and rapamycin effects on acetylation

We thank the reviewer for this insightful suggestion regarding the potential crosstalk between 4E-BP1 acetylation and phosphorylation and its possible impact on mTORC1 binding.

To address whether acetylation affects the interaction between 4E-BP1 and the mTOR complex, we attempted multiple co-immunoprecipitation strategies. These included immunoprecipitation of overexpressed Flag-4E-BP1 followed by detection of co-precipitated endogenous mTOR or Raptor, as well as immunoprecipitation of endogenous mTOR to examine co-precipitated Flag-4E-BP1. However, these approaches consistently resulted in high background signals and did not allow reliable comparison among the 4E-BP1 variants (data not shown).

We also immunoprecipitated endogenous Raptor and probe for associated Flag-4E-BP1. With this approach, we were able to weakly pull down Flag-4E-BP1, but the signal intensity was unstable and we are unable to reliably differentiate WT 4E-BP1, the acetylation-deficient mutant (K69R), or the acetylation-mimic mutant (K69Q) (**Figure R12**). P phosphorylation of 4E-BP1 by mTORC1 is known to occur in a highly dynamic manner (Mol Cell, 2021, 10.1016/j.molcel.2021.03.031), and therefore transient interaction differences may be difficult to capture by conventional co-IP approaches.

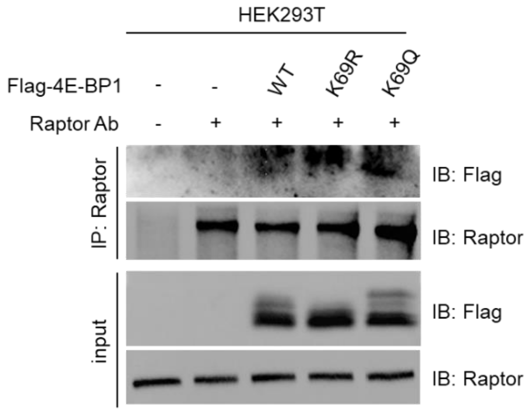

Figure R12. Attempts to examine the effects of 4E-BP1 acetylation on mTORC1 binding. Flag-4E-BP1 WT, K69R, and K69Q were overexpressed in HEK293T cells, cells were lysed in the CHAPS lysis buffer and endogenous Raptor was immunoprecipitated. The co-IPed Flag-4E-BP1 WT and mutants were examined via immunoblotting. Representative results from five biological replicates.

To further explore potential structural implications of K69 acetylation, we analyzed the structural model of the 4E-BP1–mTORC1 complex reported by Böhm et al. (Mol Cell, 2021, 10.1016/j.molcel.2021.03.031). We reached out to Prof. Sebastian Hiller, whose laboratory generated this model, and he kindly shared the structural coordinates with us. This model represents a late phosphorylation state in which Thr37 and Thr46 are phosphorylated while Ser65 and Thr70 remain available for phosphorylation. Since our study focuses on changes in Ser65 phosphorylation, this model provides a relevant structural context. As shown in **Figure R13**, residue K69 of 4E-BP1 is positioned at the

interface between 4E-BP1 and mTOR. In this model, K69 is located near mTOR residues K2166 and R2168. Because these residues carry positive charges, electrostatic repulsion could influence the local interaction environment. Acetylation of K69 neutralizes its positive charge and may therefore modulate this interface and affect subsequent phosphorylation events without necessarily altering stable complex formation. Together, these results suggest that K69 acetylation does not markedly alter stable association between 4E-BP1 and mTORC1 but may influence phosphorylation through electrostatic effects at the mTOR–4E-BP1 interface.

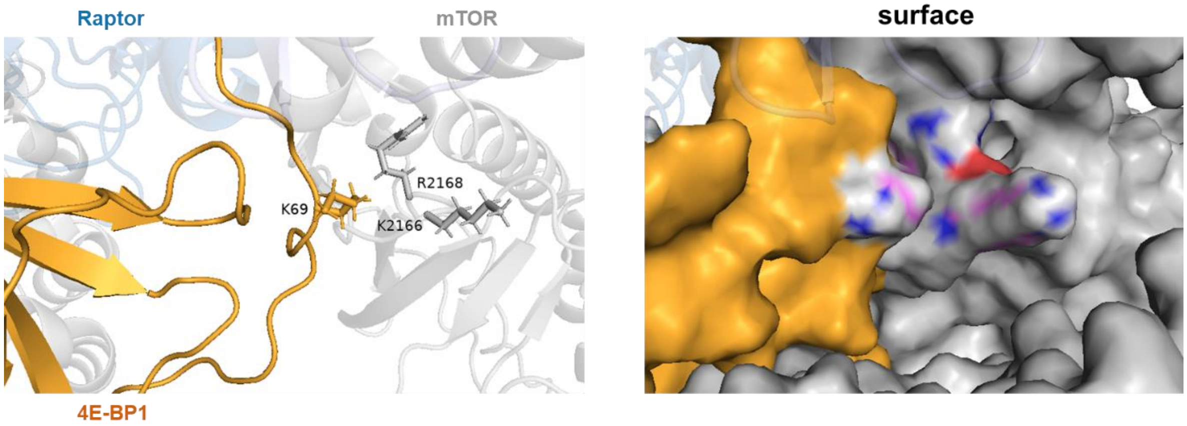

Figure R13. Modeled structure of p-4E-BP1 (T37/T46) in complex with mTOR and Raptor. Left: The positions of 4E-BP1 K69 (pink) and mTOR K2166/R2168 (magenta). 4E-BP1 K69 is within the electrostatic interaction range between K2166 and R2168. Right: protein surface of mTOR and 4E-BP1 are shown. 4E-BP1 K69 sits right at the interaction surface between the two proteins. 4E-BP1 (orange), mTOR (cyan), and Raptor (green). This figure has been added to the revised manuscript as **Appendix Figure S13**.

Finally, to determine whether mTORC1 activity influences 4E-BP1 acetylation, we treated cells with increasing concentrations of rapamycin and examined 4E-BP1 acetylation using acetyl-lysine immunoprecipitation. Rapamycin treatment did not produce noticeable changes in the acetylation level of 4E-BP1 (**Figure R14**), suggesting that mTORC1 inhibition does not significantly alter this acetylation event. Work from Shrama et al. suggested that RheB acetylation could promote mTORC1 activation. Our observation that inhibition of mTORC1 does not significantly affect 4E-BP1 acetylation suggests that the K69 acetylation event is unlikely to be directly regulated by mTORC1 activity. Instead, these results support a model in which 4E-BP1 acetylation regulates the level and the phosphorylation state of 4E-BP1 rather than being a downstream consequence of mTORC1 signaling.

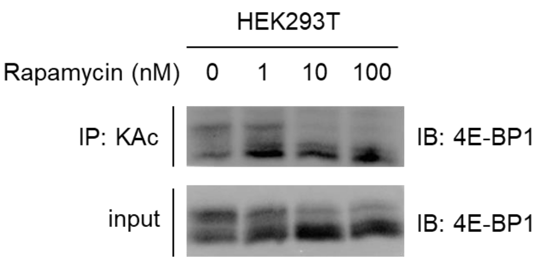

Figure R14. The effect of rapamycin on 4E-BP1 acetylation. Rapamycin treatment did not significantly affect the acetylation of 4E-BP1. Representative results from two independent biological replicates. This figure has been added to the revised manuscript as **Appendix Figure S12**.

3. Minimal Integration with Companion Manuscript
Only one sentence acknowledging the Rheb pathway
No Rheb measurements under amino acid starvation
No pathway hierarchy discussion
No substrate competition experiments
Recommendation: Add Rheb measurements in the same conditions OR substantially expand the Discussion explaining: Why 4EBP1 regulation matters if SIRT2 already inhibits mTORC1 via Rheb, Temporal dynamics and relative contributions, and whether effects are additive/synergistic/redundant
We expanded the discussion explaining Why 4EBP1 regulation matters if SIRT2 already inhibits mTORC1 via Rheb, Temporal dynamics and relative contributions, and whether effects are additive/synergistic/redundant. The model we propose is that by affecting RheB and 4E-BP1, SIRT2 can inhibit translation more effectively, allow cells to adjust more efficiently. When amino acids become deficient, if only RheB is inhibited, the existing phosphorylated 4E-BP1 and S6K will still permit some translation. In contrast, if unphosphorylated 4E-BP1 accumulates, then it will help to shut down the remaining translation. Similarly, if only RheB was affected, when amino acids become available, RheB acetylation will immediately allow translation to resume. However, with the additional 4E-BP1 regulation, the cells would have accumulated more 4E-BP1 during the absence of amino acids. The accumulated 4E-BP1 will slow down the resumption of translation to give cell more time to recover.

4. uORF Mechanism IncompleteTranslation of uORF inferred, not demonstrated

GCN4 analogy imperfect (single vs. multiple uORFs)

Mechanistic model unclear (slow scanning? reinitiation?)

Recommendation: An approach like ribosome profiling to demonstrate uORF translation; clarify the mechanistic model

To address the reviewer's concern, we analyzed publicly available ribosome profiling datasets using the GWIPS-viz genome browser and examined ribosome footprint coverage over the annotated SIRT2 uORF (133–234). Across multiple independent studies and cell types, ribosome footprint signal is consistently detected over this uORF region (**Figure R15**), indicating that this uORF is ribosome-engaged.

As a representative example, we show the Zhang et al. dataset in **Figure R16**, where the initiation-focused track shows a localized peak at/near the uORF AUG, and the elongation track shows signal extending downstream within the uORF. This data supports the presence of translation initiation events at the uORF (133-234) AUG region.

Our study show that amino acid limitation induces ISR (**Appendix Figure S1**), ISRIB attenuates SIRT2 induction (**Appendix Figure S2**), the SIRT2 5′UTR is sufficient to confer stress-responsive translational upregulation (**Figure 3A**), and disruption of the uORF AUG reduces translation output (**Figure 3B**) without altering reporter mRNA levels (**Figure 3C**). Together, these data support our conclusion that amino acid limitation activates the ISR to increase translation output from the SIRT2 main ORF through a 5′UTR/uORF-dependent mechanism. We have revised the manuscript text to clarify this mechanistic framework.

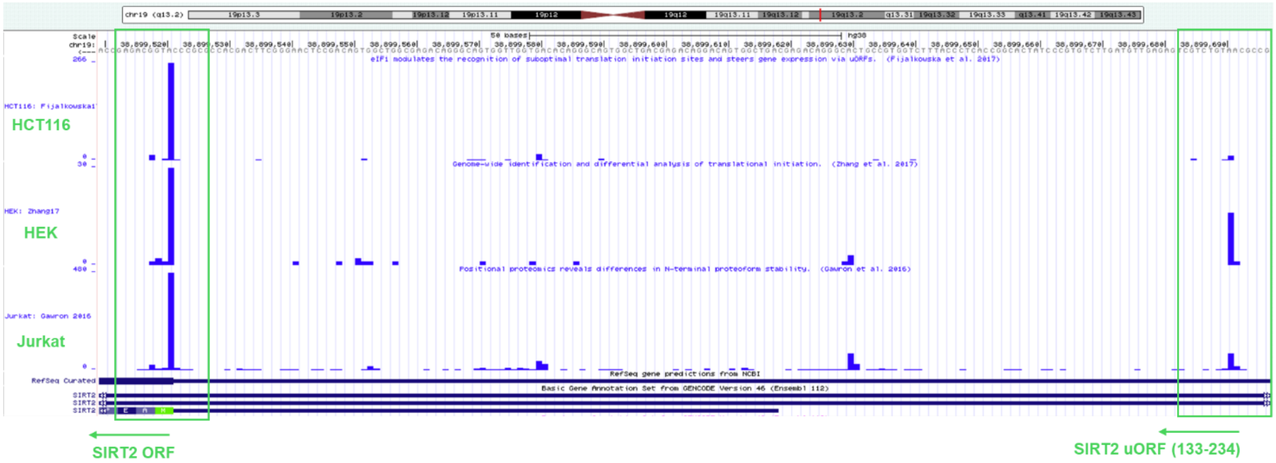

Figure R15. Ribosome footprint signal over the SIRT2 uORF region in multiple public ribosome profiling datasets. Ribosome profiling tracks from independent studies were visualized in the GWIPS-viz genome browser and aligned to the human SIRT2 transcript. Ribosome footprint coverage is detected over the annotated SIRT2 uORF (133–234), consistent with ribosome occupancy on this uORF. Datasets shown include HCT116 study (doi:10.1093/nar/gkx469), HEK study (doi:10.1038/s41467-017-

01981-8), and Jurkat study (doi:10.15252/msb.20156662). This figure has been added to the revised manuscript as **Appendix Figure S5**.

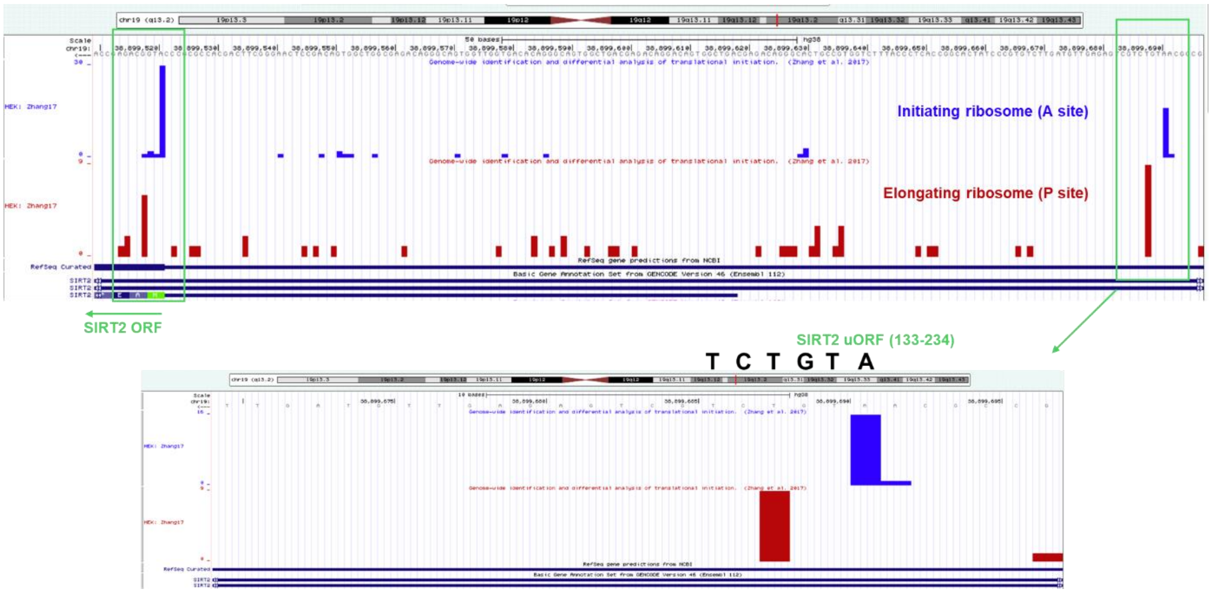

Figure R16. Representative GWIPS-viz initiation- and elongation-view tracks over the SIRT2 uORF. Shown is an example dataset ("HEK study" from **Figure R11**) visualized in the GWIPS-viz genome browser (Zhang *et al.*, doi:10.1038/s41467-017-01981-8). The A-site (initiation-focused) and P-site (elongation) tracks display signal at/near the uORF (133–234) AUG and downstream within the uORF, respectively. This figure has been added to the revised manuscript as **Appendix Figure S6**.

5. Cell Survival Data Needs Expansion
Good addition, but lacks rescue experiments
Mechanism of reduced survival is unclear
Specificity to 4EBP1 not tested
Recommendation: SIRT2 re-expression rescue (WT but not catalytic mutant); ideally test if 4EBP1 overexpression rescues

We thank the reviewer for this suggestion. To address this question, we re-expressed either wild-type SIRT2 or the catalytic-dead H187Y mutant in A549-shSIRT2 cells and assessed survival after amino acid-limiting conditions (-QKR) using both CellTiter-Glo and clonogenic assays. In the CellTiter-Glo assay (**Figure R17**), re-expression of WT SIRT2 significantly restored survival after the -QKR stress compared with shSIRT2

cells, whereas the H187Y did not restore as WT SIRT2. This data supports the conclusion that the reduced survival caused by SIRT2 depletion can be functionally rescued by SIRT2 re-expression.

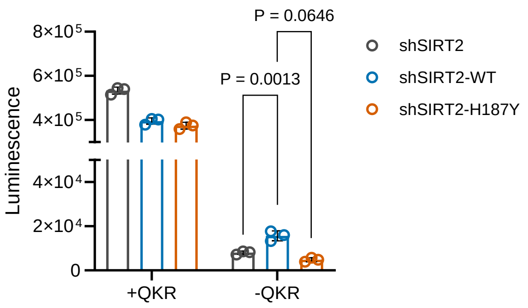

Figure R17. Survival of A549-shSIRT2 cells rescued with SIRT2-WT or H187Y mutant after amino acid limitation. A549-shSIRT2, shSIRT2-WT, shSIRT2-H187Y cells were cultured with or without amino acid limitation for seven days (+QKR or -QKR). The cells were then reseeded in 96-well plates with complete medium at the same split ratio and cell growth was assessed after three days via CellTiter-Glo 2.0. This figure has been added to the revised manuscript as **Appendix Figure 6B**.

Minor Concerns
ISRIB experiment (Fig R3): Only n=2, acts downstream of eIF2α-P. Need eIF2α S51A mutant for definitive test

We thank the reviewer for this suggestion. We have increased **Figure R3** to n=3 independent biological replicates and the result is reproducible. We agree that ISRIB acts downstream of eIF2α phosphorylation, and we therefore use this experiment to support involvement of the ISR translational control node rather than to claim a definitive requirement for eIF2α Ser51 phosphorylation. Generating and validating an eIF2α S51A mutant line would be beyond the scope of this revision and would substantially extend the timeline. We have clarified the interpretation in the text.

Temporal dynamics missing: No time-course showing kinetics of all events

We agree that temporal information is important, and we note that time-course experiments are already included in the manuscript. Specifically, the kinetics of the relevant events are presented in **Figure 1D–1G**, **Figure 2A**, **Figure 2C,** and **Figure 3A**.

Cell line variability: Could include a sumsmary table???

The cell line used for each experiment is indicated in the corresponding figure/legend. In addition, we have now added a summary table (**Appendix Table S1**) of all cell lines used in the Supplementary Materials.

Acetyltransferase unknown: Would complete the regulatory cycle

We thank the reviewer for this comment. While identification of the acetyltransferase would further complete the regulatory cycle, our study is centered on establishing SIRT2 as the “eraser”-mediated control of 4E-BP1 acetylation and its downstream impact. We therefore did not pursue “writer” identification here, but we agree it will be of interest in future studies.

Antibody validation: K69 acetyl-specific antibody not validated; may need mass spec confirmation

We did not use a K69 acetyl-specific antibody in this study. We used a pan-acetyllysine antibody, which has been validated by many researchers over the years. MS confirmation is not applicable to the experiments presented.

Discussion Needs: Comprehensive model figure showing full pathway including Rheb, Limitations section: E3 ligase, acetyltransferase, relative contribution vs. Rheb, Biological significance: Disease relevance, why PTM control needed beyond phosphorylation

We have included these discussions now.

More comments from referee 3:

These manuscripts should be published separately, not as integrated companion papers. While both describe SIRT2 regulating translation, they actually represent distinct stress-response pathways rather than a unified mechanism. Zi et al. show amino acid starvation increases SIRT2 protein levels (via ISR-mediated translation), which then stabilizes 4EBP1. Sharma et al. show glucose starvation increases SIRT2 activity (not protein), which degrades Rheb and inhibits mTORC1. These are fundamentally different regulatory contexts engaging SIRT2 through different mechanisms.

More importantly, there's no experimental evidence that the pathways work together.

Despite reviewer requests, neither manuscript measures both substrates under the same conditions, tests substrate competition, or performs epistasis experiments. The "integration" exists only in the Discussion paragraphs, using nearly identical language about PTM enzymes having multiple substrates. The in vivo data from Sharma et al. actually suggest Rheb regulation dominates-SIRT2-KO hearts have high Rheb, low 4EBP1, yet still show increased protein synthesis and hypertrophy. If 4EBP1 stabilization were equally important, low 4EBP1 levels should provide some protection, but they don't.

The one finding that could meaningfully link them-Zi et al. Figure R8 showing K69 acetylation increases 4EBP1's phosphorylation competence-is buried in revision responses rather than integrated into either narrative. This could explain why both upstream (Rheb) and downstream (4EBP1) regulation matter, but neither manuscript develops this insight.

I would recommend publishing these as independent studies, each acknowledging the other exists but not claiming functional integration that isn't experimentally demonstrated. For true back-to-back publication, you would need at minimum: one experiment testing both substrates under the same stress conditions, an epistasis test showing whether 4EBP1 overexpression rescues SIRT2-KO phenotypes, and a unified model explaining when and where each mechanism predominates. Without this, they're two separate papers about SIRT2, not a coordinated study of multi-substrate regulation.

Given the reviewer’s comments and according to the editor’s suggestion, we would like to publish the two manuscripts a related and linked manuscripts, instead of “back-to-back”. We have revised the discussion to increase the integration of the two linked manuscript,

Regarding the reviewer’s comment that “The in vivo data from Sharma et al. actually suggest Rheb regulation dominates-SIRT2-KO hearts have high Rheb, low 4EBP1, yet still show increased protein synthesis and hypertrophy. If 4EBP1 stabilization were equally important, low 4EBP1 levels should provide some protection, but they don't”, we believe the data mentioned by the reviewer precisely support that both RheB and 4EBP1 regulation contribute to the role of SIRT2 in translation regulation: (1) by increasing RheB acetylation, SIRT2 KO activates mTOR and increase 4EBP1 phosphorylation, leading to increase in translation; (2) by increasing 4EBP1 acetylation, SIRT2 KO increases 4EBP1 acetylation, ubiquitination, and degradation, which also leads to increase in translation (4EBP1 is an inhibitor of translation). These two effects are additive or synergistic.

Prof. Hening Lin
The University of Chicago
Department of Medicine and Department of Chemistry
Chicago, IL 60637
United States

Dear Prof. Lin,

I am very pleased to accept your manuscript for publication in the next available issue of EMBO reports. Thank you for your contribution to our journal.

Your manuscript will be processed for publication by EMBO Press. It will be copy edited and you will receive page proofs prior to publication. Please note that you will be contacted by Springer Nature Author Services to complete licensing and payment information.

You may qualify for financial assistance for your publication charges - either via a Springer Nature fully open access agreement or an EMBO initiative. Check your eligibility: https://link.springer.com/journal/44319/how-to-publish-with-us

Should you be planning a Press Release on your article, please get in contact with embo_production@springernature.com as early as possible in order to coordinate publication and release dates.

If you have any questions, please do not hesitate to contact the Editorial Office. Thank you for your contribution to EMBO Reports.

Best regards,
Esther

Esther Schnapp, PhD
Senior Editor
EMBO reports

Referee #1:

The authors have fully addressed my remaining concerns in this version (V3) of the manuscript.

>>> Please note that it is EMBO Reports policy for the transcript of the editorial process (containing referee reports and your response letter) to be published as an online supplement to each paper. If you do NOT want this, you will need to inform the Editorial Office via email immediately. More information is available here: https://link.springer.com/partners/embo-press/editorial-policies#Peer%20review

EMBO Press Author Checklist

Corresponding Author Name: Hening Lin	
Journal Submitted to: EMBO Reports	
Manuscript Number: EMBOR-2025-61642V2	

USEFUL LINKS FOR COMPLETING THIS FORM
The EMBO Journal - Author Guidelines
EMBO Reports - Author Guidelines
Molecular Systems Biology - Author Guidelines
EMBO Molecular Medicine - Author Guidelines

Reporting Checklist for Life Science Articles (updated January
This checklist is adapted from Materials Design Analysis Reporting (MDAR) Checklist for Authors. MDAR establishes a minimum set of requirements in transparent reporting in the life sciences (see Statement of Task: 10.31222/osf.io/9sm4x). Please follow the journal's guidelines in preparing your
Please note that a copy of this checklist will be published alongside your article.

Abridged guidelines for figures
1. Data
The data shown in figures should satisfy the following conditions:
- the data were obtained and processed according to the field's best practice and are presented to reflect the results of the experiments in an accurate and unbiased manner.
- ideally, figure panels should include only measurements that are directly comparable to each other and obtained with the same assay.
- plots include clearly labeled error bars for independent experiments and sample sizes. Unless justified, error bars should not be shown for technical
- if n<5, the individual data points from each experiment should be plotted. Any statistical test employed should be justified.
- Source Data should be included to report the data underlying figures according to the guidelines set out in the authorship guidelines on Data

2. Captions
Each figure caption should contain the following information, for each panel where they are relevant:
- a specification of the experimental system investigated (eg cell line, species name).
- the assay(s) and method(s) used to carry out the reported observations and measurements.
- an explicit mention of the biological and chemical entity(ies) that are being measured.
- an explicit mention of the biological and chemical entity(ies) that are altered/varied/perturbed in a controlled manner.
- the exact sample size (n) for each experimental group/condition, given as a number, not a range;
- a description of the sample collection allowing the reader to understand whether the samples represent technical or biological replicates (including how many animals, litters, cultures, etc.).
- a statement of how many times the experiment shown was independently replicated in the laboratory.
- definitions of statistical methods and measures:
  - common tests, such as t-test (please specify whether paired vs. unpaired), simple χ2 tests, Wilcoxon and Mann-Whitney tests, can be unambiguously identified by name only, but more complex techniques should be described in the methods section;
  - are tests one-sided or two-sided?
  - are there adjustments for multiple comparisons?
  - exact statistical test results, e.g., P values = x but not P values < x;
  - definition of 'center values' as median or average;
  - definition of error bars as s.d. or s.e.m.

Please complete ALL of the questions below.
Select "Not Applicable" only when the requested information is not relevant for your study.

Materials

Newly Created Materials	Information included in the manuscript?	In which section is the information available? (Reagents and Tools Table, Materials and Methods, Figures, Data Availability Section)
New materials and reagents need to be available; do any restrictions apply?	Yes	Reagents and Tools Table

Antibodies	Information included in the manuscript?	In which section is the information available? (Reagents and Tools Table, Materials and Methods, Figures, Data Availability Section)
For **antibodies** provide the following information:<br>- Commercial antibodies: RRID (if possible) or supplier name, catalogue number and or/clone number<br>- Non-commercial: RRID or citation	Yes	Reagents and Tools Table, Materials and Methods

DNA and RNA sequences	Information included in the manuscript?	In which section is the information available? (Reagents and Tools Table, Materials and Methods, Figures, Data Availability Section)
**Short novel DNA or RNA including primers, probes**: provide the sequences.	Yes	Reagents and Tools Table, Materials and Methods

Cell materials	Information included in the manuscript?	In which section is the information available? (Reagents and Tools Table, Materials and Methods, Figures, Data Availability Section)
**Cell lines:** Provide species information, strain. Provide accession number in repository **OR** supplier name, catalog number, clone number, and/**OR** RRID.	Yes	Reagents and Tools Table
**Primary cultures:** Provide species, strain, sex of origin, genetic modification status.	Not Applicable	
Report if the cell lines were recently **authenticated** (e.g., by STR profiling) and tested for mycoplasma contamination.	Not Applicable	

Experimental animals	Information included in the manuscript?	In which section is the information available? (Reagents and Tools Table, Materials and Methods, Figures, Data Availability Section)
**Laboratory animals or Model organisms:** Provide species, strain, sex, age, genetic modification status. Provide accession number in repository **OR** supplier name, catalog number, clone number, **OR** RRID.	Not Applicable	
**Animal observed in or captured from the field:** Provide species, sex, and age where possible.	Not Applicable	
Please detail **housing and husbandry conditions**.	Not Applicable	

Plants and microbes	Information included in the manuscript?	In which section is the information available? (Reagents and Tools Table, Materials and Methods, Figures, Data Availability Section)
**Plants:** provide species and strain, ecotype and cultivar where relevant, unique accession number if available, and source (including location for collected wild specimens).	Not Applicable	
**Microbes:** provide species and strain, unique accession number if available, and source.	Not Applicable	

Human research participants	Information included in the manuscript?	In which section is the information available? (Reagents and Tools Table, Materials and Methods, Figures, Data Availability Section)
If collected and within the bounds of privacy constraints report on age, sex and gender or ethnicity for all study participants.	Not Applicable	

Core facilities	Information included in the manuscript?	In which section is the information available? (Reagents and Tools Table, Materials and Methods, Figures, Data Availability Section)
If your work benefited from core facilities, was their service mentioned in the acknowledgments section?	Not Applicable	

Design

Study protocol	Information included in the manuscript?	In which section is the information available? (Reagents and Tools Table, Materials and Methods, Figures, Data Availability Section)
If study protocol has been **pre-registered, provide DOI in the manuscript**. For clinical trials, provide the trial registration number **OR** cite DOI.	Not Applicable	
Report the **clinical trial registration number** (at ClinicalTrials.gov or equivalent), where applicable.	Not Applicable	

Laboratory protocol	Information included in the manuscript?	In which section is the information available? (Reagents and Tools Table, Materials and Methods, Figures, Data Availability Section)
Provide DOI OR other citation details if **external detailed step-by-step protocols** are available.	Yes	Materials and Methods

Experimental study design and statistics	Information included in the manuscript?	In which section is the information available? (Reagents and Tools Table, Materials and Methods, Figures, Data Availability Section)
Include a statement about **sample size** estimate even if no statistical methods were used.	Yes	Figure legends
Were any steps taken to minimize the effects of subjective bias when allocating animals/samples to treatment (e.g. **randomization procedure**)? If yes, have they been described?	Not Applicable	
Include a statement about **blinding** even if no blinding was done.	Not Applicable	
Describe **inclusion/exclusion criteria** if samples or animals were excluded from the analysis. Were the criteria pre-established?<br><br>If sample or data points were omitted from analysis, report if this was due to attrition or intentional exclusion and provide justification.	Not Applicable	
For every figure, are **statistical tests** justified as appropriate? Do the data meet the assumptions of the tests (e.g., normal distribution)? Describe any methods used to assess it. Is there an estimate of variation within each group of data? Is the variance similar between the groups that are being statistically compared?	Yes	Materials and Methods; Figure legends; Data Availability Section

Sample definition and in-laboratory replication	Information included in the manuscript?	In which section is the information available? (Reagents and Tools Table, Materials and Methods, Figures, Data Availability Section)
In the figure legends: state number of times the experiment was **replicated** in laboratory.	Yes	Figure legends
In the figure legends: define whether data describe **technical or biological replicates**.	Yes	Figure legends

Ethics

Ethics	Information included in the manuscript?	In which section is the information available? (Reagents and Tools Table, Materials and Methods, Figures, Data Availability Section)
Studies involving **human participants**: State details of **authority granting ethics approval** (IRB or equivalent committee(s), provide reference number for approval.	Not Applicable	
Studies involving **human participants**: Include a statement confirming that **informed consent** was obtained from all subjects and that the experiments conformed to the principles set out in the WMA Declaration of Helsinki and the Department of Health and Human Services Belmont Report.	Not Applicable	
Studies involving **human participants:** For publication of **patient photos**, include a statement confirming that consent to publish was obtained.	Not Applicable	
Studies involving experimental **animals**: State details of **authority granting ethics approval** (IRB or equivalent committee(s), provide reference number for approval. Include a statement of compliance with ethical regulations.	Not Applicable	
Studies involving **specimen and field samples:** State if relevant **permits** obtained, provide details of authority approving study; if none were required, explain why.	Not Applicable	

Dual Use Research of Concern (DURC)	Information included in the manuscript?	In which section is the information available? (Reagents and Tools Table, Materials and Methods, Figures, Data Availability Section)
Could your study fall under dual use research restrictions? Please check biosecurity documents and list of **select agents and toxins** (CDC): https://www.selectagents.gov/sat/list.htm	Not Applicable	
If you used a select agent, is the security level of the lab appropriate and reported in the manuscript?	Not Applicable	
If a study is subject to dual use research of concern regulations, is the name of the **authority granting approval and reference number** for the regulatory approval provided in the manuscript?	Not Applicable	

Reporting

The MDAR framework recommends adoption of discipline-specific guidelines, established and endorsed through community initiatives. Journals have their own policy about requiring specific guidelines and recommendations to complement MDAR.

Adherence to community standards	Information included in the manuscript?	In which section is the information available? (Reagents and Tools Table, Materials and Methods, Figures, Data Availability Section)
State if relevant guidelines or checklists (e.g., **ICMJE, MIBBI, ARRIVE, PRISMA**) have been followed or provided.	Not Applicable	
For **tumor marker prognostic studies,** we recommend that you follow the **REMARK** reporting guidelines (see link list at top right). See author guidelines, under 'Reporting Guidelines'. Please confirm you have followed these guidelines.	Not Applicable	
For **phase II and III randomized controlled trials**, please refer to the **CONSORT** flow diagram (see link list at top right) and submit the CONSORT checklist (see link list at top right) with your submission. See author guidelines, under 'Reporting Guidelines'. Please confirm you have submitted this list.	Not Applicable	

Data Availability

Data availability	Information included in the manuscript?	In which section is the information available? (Reagents and Tools Table, Materials and Methods, Figures, Data Availability Section)
Have **primary datasets** been deposited according to the journal's guidelines (see 'Data Deposition' section) and the respective accession numbers provided in the Data Availability Section?	Not Applicable	
Were **human clinical and genomic datasets** deposited in a public access-controlled repository in accordance to ethical obligations to the patients and to the applicable consent agreement?	Not Applicable	
Are **computational models** that are central and integral to a study available without restrictions in a machine-readable form? Were the relevant accession numbers or links provided?	Not Applicable	
If publicly available data were reused, provide the respective **data citations in the reference list**.	Not Applicable	
